# Sex differences in neuromodulatory subcortical systems and their implications for Alzheimer's disease

**DOI:** 10.1002/alz.71291

**Published:** 2026-03-19

**Authors:** Rosaria J. Rae, Jessica Marie Hunter Alberhasky, Marion Baillet, Debra A. Bangasser, Michael E. Belloy, Anne S. Berry, Chiara Berteotti, Hannah Bow, Rachel Buckley, Jessica Z. K. Caldwell, Matteo Carpi, Benjamin J. Clark, Claire J. Ciampa, Alexander C. Conley, Martin J. Dahl, Zoe R. Donaldson, Alexander J. Ehrenberg, Gillian Einstein, Neus Falgàs, Haley A. Fenlon, Megan C. Fitzhugh, Robert C. Froemke, Clara Gallay, Lea Tenenholz Grinberg, Derek A. Hamilton, Zia Hasan, Oihane Uriarte Huarte, Shaista Jabeen, Heidi I. L. Jacobs, Louis John Kolling, Elouise A. Koops, Sabrina Lenzoni, Claudio Liguori, Riccardo Manca, Catherine A. Marcinkiewcz, Tamunotonye Omoluabi, Rademene Oria, Caitlin A. Orsini, Nancy Elizabeth Ortega, Judy Pa, Nathan S. Pentkowski, Joana B. Pereira, Rhudovic Ramos, Derya Sargin, Abhijit Satpati, Maria Clara Selles, Mabel Seto, Shabana M. Shaik, Shireen Sindi, Gowoon Son, Valentine Ucheagwu, Maxime Van Egroo, Qi Yuan, Michael A. Kelberman

**Affiliations:** ^1^ Sex and Gender Differences in Alzheimer's Disease Professional Interest Area International Society to Advance Alzheimer's Research and Treatment Alzheimer's Association Chicago Illinois USA; ^2^ RAEdiant Research LLC Royal Oak Michigan USA; ^3^ Carver College of Medicine University of Iowa Iowa City Iowa USA; ^4^ Athinoula A. Martinos Center for Biomedical Imaging Department of Radiology Massachusetts General Hospital and Harvard Medical School Boston Massachusetts USA; ^5^ Center for Behavioral Neuroscience and Neuroscience Institute Georgia State University Atlanta Georgia USA; ^6^ NeuroGenomics and Informatics Center Washington University School of Medicine St. Louis Missouri USA; ^7^ Department of Neurology Washington University School of Medicine St. Louis Missouri USA; ^8^ Psychology Department Brandeis University Waltham Massachusetts USA; ^9^ Department of Biomedical and NeuroMotor Sciences Alma Mater Studiorum – University of Bologna Bologna Italy; ^10^ Interdisciplinary Neuroscience Program The University of Texas at Austin Austin Texas USA; ^11^ Department of Neurology Massachusetts General Hospital Boston Massachusetts USA; ^12^ Department of Neurology School of Medicine and Public Health University of Wisconsin–Madison Madison Wisconsin USA; ^13^ Department of Human Neuroscience Sapienza University of Rome Rome Italy; ^14^ Department of Psychology The University of New Mexico Albuquerque New Mexico USA; ^15^ Biology Department Brandeis University Waltham Massachusetts USA; ^16^ Center for Cognitive Medicine Department of Psychiatry and Behavioral Sciences Vanderbilt University Medical Center Nashville Tennessee USA; ^17^ Neuromodulatory Subcortical Systems Professional Interest Area International Society to Advance Alzheimer's Research and Treatment Alzheimer's Association Chicago Illinois USA; ^18^ Leonard Davis School of Gerontology University of Southern California Los Angeles California USA; ^19^ Center for Lifespan Psychology Max Planck Institute for Human Development Berlin Germany; ^20^ Max Planck UCL Centre for Computational Psychiatry and Ageing Research Berlin Germany; ^21^ Department of Cellular Molecular and Developmental Biology University of Colorado Boulder Boulder Colorado USA; ^22^ Department of Psychology & Neuroscience University of Colorado Boulder Boulder Colorado USA; ^23^ Memory and Aging Center University of California San Francisco San Francisco California USA; ^24^ Department of Psychology University of Toronto Toronto Ontario Canada; ^25^ Alzheimer's and Other Cognitive Disorders Unit Neurology Service Hospital Clínic de Barcelona, Fundació Recerca Clínic Barcelona‐IDIBAPS Barcelona Spain; ^26^ Department of Neurosciences University of California San Diego La Jolla California USA; ^27^ Departments of Neuroscience and Otolaryngology New York University School of Medicine New York New York USA; ^28^ Barcelonaβeta Brain Research Center Barcelona Spain; ^29^ Universitat Pompeu Fabra Barcelona Spain; ^30^ Department of Laboratory Medicine and Pathology Mayo Clinic Jacksonville Florida USA; ^31^ Biomedical Sciences Faculty of Medicine Memorial University of Newfoundland, Newfoundland St. John's Canada; ^32^ Division of Medical & Scientific Relations Alzheimer's Association Chicago Illinois USA; ^33^ Department of Psychology Hotchkiss Brain Institute Alberta Children's Hospital Research Institute University of Calgary Calgary Alberta Canada; ^34^ Department of Cellular and Systems Pharmacology University of Florida Gainesville Florida USA; ^35^ Department of Psychology University of Innsbruck Innsbruck Austria; ^36^ Department of Systems Medicine University of Rome Tor Vergata Rome Italy; ^37^ Neurology Unit University Hospital of Rome Tor Vergata Rome Italy; ^38^ Department of Medicine and Surgery University of Parma Parma Italy; ^39^ Okuku Campus University of Cross River State Yala Nigeria; ^40^ Department of Psychology and Neurology The University of Texas at Austin Austin Texas USA; ^41^ Clinical Neuroscience Department Karolinska Institute Stockholm Sweden; ^42^ The University of New Mexico School of Medicine University of New Mexico Alburquerque New Mexico USA; ^43^ Department of Neuroscience Mayo Clinic Jacksonville Florida USA; ^44^ Institute for Translational Neuroscience NYU Grossman School of Medicine New York New York USA; ^45^ Department of Pediatrics University of Arizona Tucson Arizona USA; ^46^ Division of Clinical Geriatrics Department of Neurobiology Care Sciences, and Society Karolinska Institute Stockholm Sweden; ^47^ Ageing Epidemiology Research Unit (AGE) School of Public Health Faculty of Medicine Imperial College London London UK; ^48^ Department of Psychology Nnamdi Azikiwe University Awka Nigeria; ^49^ Faculty of Health Medicine and Life Sciences Mental Health and Neuroscience Research Institute Alzheimer Centre Limburg Maastricht University Maastricht the Netherlands

**Keywords:** acetylcholine, brainstem, corticotropin releasing hormone, dopamine, histamine, hypothalamus, neuromodulatory subcortical systems, norepinephrine, orexin/hypocretin, oxytocin, selective vulnerability, serotonin, sex differences, vasopressin

## Abstract

Neuromodulatory subcortical systems (NSSs) are uniquely susceptible to dementia‐related pathology, leading to frequent molecular and behavioral impairments associated with altered function of these nuclei. Some of these systems display clear sex‐specific cytoarchitecture and signaling leading to distinct physiology and behavioral outputs in males and females, while other regions display nominal sex differences. However, the relevance of sex differences in modulating dysfunction of NSSs in Alzheimer's disease (AD) and related dementias is not well understood. This review is a joint effort by the Neuromodulatory Subcortical Systems and Sex and Gender Differences in Alzheimer's Disease Professional Interest Areas of the Alzheimer's Association. We review sex differences in NSSs, both in non‐disease states and in AD models and patients. We highlight the possible role of NSSs in driving sex‐specific AD susceptibility and potential footholds for sex‐based interventions targeting these systems. We conclude by outlining immediate and long‐term actions to address the intersection of NSSs, sex, and AD.

## Highlights

- Neuromodulatory subcortical systems are uniquely vulnerable in Alzheimer's disease.
- Biological sex is an important factor that modulates dementia risk and progression.
- Neuromodulatory subcortical systems show sex differences in structure and function.
- Sex‐dependent neuromodulatory nuclei dysfunction in dementia is understudied.

## 1 INTRODUCTION

Dementias are widely recognized as disorders of severe memory decline, with the most prevalent form being Alzheimer's disease (AD). However, neuropsychiatric symptoms including anxiety, depression, social dysfunction, apathy, and sleep disturbances are highly prevalent, often emerging prior to cognitive deficits, and persist throughout the disease course.^1^, ^2^, ^3^ These early symptoms point to dysfunction in neural systems beyond those canonically used for diagnosis, specifically the involvement of neuromodulatory subcortical systems (NSSs) located in the brainstem and hypothalamus. The neuromodulators produced by these nuclei are critical for regulating molecular processes and behaviors that go awry in AD.^4^ Moreover, AD symptoms associated with dysfunction of NSSs appear coincident with accumulation of disease‐specific pathological hallmarks in these regions.^1^, ^4^, ^5^, ^6^, ^7^, ^8^, ^9^, ^10^, ^11^, ^12^, ^13^

We have previously proposed that understanding the mechanisms underlying selective vulnerability of NSSs is critical for improving outcomes and treatment options targeting these systems.^4^ However, biological sex is one factor that has received little attention in its potential to modulate the susceptibility of NSSs in AD. There are well described sex differences in AD, which occur more frequently in women than men and often follow a more severe clinical trajectory in women.^14^, ^15^, ^16^, ^17^, ^18^, ^19^, ^20^, ^21^, ^22^ While women's increased longevity is frequently cited as a key contributing factor, this explanation remains controversial. For example, while animal models consistently show sex differences in neuromodulator signaling, behavioral outcomes, and vulnerability to amyloid beta (Aβ) and tau, human studies remain limited and inconsistent. This could be due to a failure to adequately account for sex‐specific variables such as hormonal contraceptive use, menstrual cycle phase, or menopausal status. Such factors can significantly alter neuromodulator synthesis, turnover, and receptor expression, ultimately affecting function and dysfunction of neural circuits. Importantly, NSSs exhibit inherent sex differences in their organization, structure, signaling, and biochemical properties even under non‐disease contexts. Therefore, understanding the ways in which sex shapes NSSs form and function may be key to explaining selective vulnerability and divergent clinical outcomes in men and women, as well as to guiding the design of more precise diagnostic and therapeutic strategies.

**FIGURE 1 alz71291-fig-0001:**
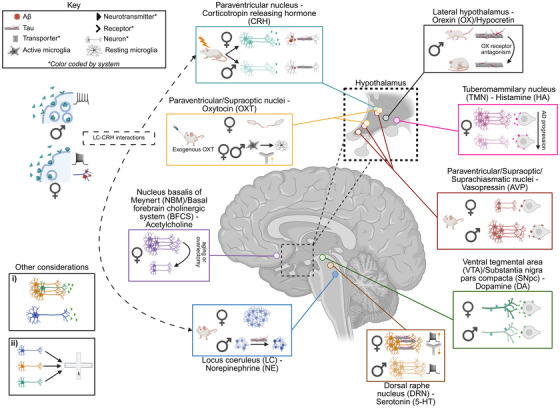
Overview of sex differences in neuromodulatory subcortical systems (NSSs) under baseline conditions and in Alzheimer's disease (AD). Nucleus basalis of Meynert (NBM)/basal forebrain cholinergic system (BFCS) – acetylcholine: Cholinergic decline (lower volume, neuron number, innervation, receptor activity) is observed after ovariectomy and in aging women. Outstanding question(s): What factors influence the efficacy of hormone replacement therapy for alleviating cholinergic decline (age at initiation, treatment duration, hormone formulation)? Ventral tegmental area (VTA)/substantia nigra pars compacta (SNpc) – dopamine (DA): At baseline, females/women display greater DA receptor and transporter density, release, and synthesis capacity (darker shading = greater synthesis capacity). Preclinical AD models show more severe DA‐related behavioral phenotypes and DA plaque burden along with reduced transporter density and synthesis capacity in females. Outstanding question(s): Can we leverage large, publicly available datasets to confirm preclinical sex differences in human populations? Locus coeruleus (LC) – norepinephrine (NE): At baseline, the female LC is larger in volume, has more neurons, and more elaborate dendritic fields. LC tau expression induces upregulation of NE synthesis genes in males only. Outstanding question(s): How do these robust sex differences in animal models translate to the human condition, including vulnerability of and treatment strategies targeting the LC–NE system? Dorsal raphe nucleus (DRN) – serotonin (5‐HT): Most studies report higher 5‐HT synthesis, metabolism, and turnover in females/women, while males display higher neuronal firing at baseline. Tau expression in DRN 5‐HT neurons causes spatial working memory deficits and impairs neuronal excitability that is specific to females. Outstanding question(s): To what extent is AD vulnerability shaped by DRN 5‐HT dysfunction, particularly during periods of hormonal transition? Paraventricular nucleus – corticotropin releasing hormone (CRH): CRH levels are higher in women/females, especially in response to stress. Stress increases AD pathology and inflammation to a greater extent in women/females. Outstanding question(s): Sex differences in CRH levels and CRH_1_ receptor distribution appear to be age dependent, but how do these development differences impact susceptibility to and progression of AD? LC–CRH interactions: Female LC neurons have a higher discharge rate in response to CRH and lower CRH receptor internalization relative to male LC neurons. Only females show colocalization of amyloid beta (Aβ) in LC axons in response to forebrain CRH overexpression. Outstanding question(s): What is the extent of crosstalk between other systems, their dependence on sex, and the implications for AD? Paraventricular/supraoptic nuclei – oxytocin (OXT): Sex differences in OXT structure and function are highly species specific and sometimes contradictory. Preclinical studies show that exogenous OXT (e.g., intranasal OXT) rescues behavioral and molecular AD‐related phenotypes in both sexes, including protection against working and spatial memory decline and a reduction in neuroinflammation. One study demonstrates that OXT can induce social deficits in female AD mice. Outstanding question(s): How do these preclinical findings translate to humans; specifically, how does AD impact OXT‐producing neurons and downstream signaling, and how might these changes affect AD‐related symptoms in a sex‐dependent manner? Paraventricular/supraoptic/suprachiasmatic nuclei – vasopressin (AVP): At baseline, males have more and larger AVP neurons, and greater innervation density and receptor expression. Whether these differences translate to humans is unknown. Diffuse expression and differing susceptibility of AVP nuclei suggest contributions to distinct facets of AD. Outstanding question(s): What is the extent of AVP dysfunction along AD progression and how is this modified by sex? Tuberomammillary nucleus (TMN) – histamine (HA): Androgens methylate HA, reducing baseline circulating levels in males compared to females. Women show greater loss of TMN neurons and HA synthesis enzymes but have greater HA receptor expression in AD. Outstanding question(s): Given HA signals immune responses in the periphery, what are the sex‐dependent effects of peripheral HA signaling on AD progression? Lateral hypothalamus – orexin (OX)/hypocretin: Males appear more responsive to pharmacological OX interventions (reduced hyperphosphorylated tau, normalization of sleep patterns), though responses in females are underexplored. While human evidence for sex differences in OX signaling remains inconsistent, dual OX receptor antagonists improve sleep in AD patients regardless of sex. Outstanding question(s): Does hormonal status and/or other sex‐related factors influence the efficacy of OX‐based therapeutics for sleep, AD, and their interaction? Other considerations: (1) What is the role of non‐primary neurotransmitters released by NSSs in AD (e.g., DA release from the LC or DRN subpopulations)? (2) What are the relative contributions of different NSSs to the same symptoms (e.g., contributions of NE vs. 5‐HT vs. CRH in anxiety‐like behaviors)? How are these phenotypes modified by sex? Figure generated in bioRender.

This joint effort by the Neuromodulatory Subcortical Systems and Sex and Gender Differences in Alzheimer's Disease Professional Interest Areas of the Alzheimer's Association reviews sex differences in NSSs (Table 1). Moving forward, we will use the term “preclinical” to describe studies using cell lines, rodents, non‐human primates, and other non‐human subjects. Similarly, in cases in which preclinical models are discussed, we will use the terms “male/female” to refer to subjects whereas human studies will refer to participants as men/women. Finally, when discussing menopause in women, unless otherwise stated these women were spontaneously menopausal, as opposed to those that had been subject to early ovarian removal. We focus on nine key neuromodulators: acetylcholine, dopamine (DA), norepinephrine (NE), serotonin (5‐HT), corticotropin releasing hormone (CRH), oxytocin (OXT), arginine vasopressin (AVP), histamine (HA), and orexin (OX)/hypocretin. These systems arise from distinct subcortical nuclei but are similar in their widespread projections to cortical and limbic regions. Their broad connectivity and susceptibility to early pathology position these regions as central influencers of molecular and behavioral symptoms of AD, in addition to disease progression. While some of these nuclei have been heavily implicated in the pathophysiology of AD (e.g., acetylcholine, DA, NE), others currently lack mechanistic depth (e.g., AVP, OXT, HA), particularly in the context of sex differences. We have therefore provided a broad overview of notable sex differences under baseline conditions and/or in AD for each NSS, along with outstanding questions to be addressed by future research (Figure 1). By further discussing these outcomes, especially in the context of sex, we may be able to better delineate mechanisms and symptoms that result in precision treatments for dementia.

**TABLE 1:** Summary of studies on sex differences in NSSs.

Acetylcholine			
Citation	Species/model	Sex	Summary
Gibbs, 1998^41^ ^,^ ^$^	Sprague–Dawley rats	Male and female	No differences were seen in medial septum or NBM cholinergic neurons between sexes at any of the ages. Ovariectomized rats had lower choline acetyltransferase and trkA activity in the medial septum and NBM; estradiol partially restored choline acetyltransferase activity.
Veng et al., 2003^42^ ^,^ ^$^	F344 rats	Male and female	Young male rats had larger BFCS neurons and better spatial memory, whereas aged rats showed no differences between sexes on spatial memory or cholinergic neuron density.
Yamamoto et al., 2007^43^ ^,^ ^*^	Wistar rats	Female	Rats receiving estradiol or J 861 had higher levels of choline acetyltransferase positive neurons than ovariectomized rats.
Batallán Burrowes et al., 2022^44^ ^,^ ^*^	Long Evans rats	Female	Rats receiving estradiol had similar M_1_ receptor proteins and vesicular acetylcholine transporter as intact rats, and greater protein levels compared to ovariectomized rats.
Gibbs et al., 1994^49^ ^,^ ^*^	Sprague‐Dawley rats	Female	Estradiol increased levels of choline acetyltransferase in the medial septum and NBM, and reduced nerve growth factor and trkA in the hippocampus, medial septum, and NBM.
Gibbs et al., 1998^64^ ^,^ ^*^	Sprague‐Dawley rats	Female	Rats given estradiol performed better than sham under scopolamine or lorazepam challenge on passive avoidance task.
Rapp et al., 2003^68^ ^,^ ^*^	Rhesus macaques	Female	Monkeys given estradiol performed better on spatial and working memory tasks than monkeys given vehicle.
Gibbs, 2000^48^ ^,^ ^*^	Sprague‐Dawley rats	Female	Rats given estradiol or estradiol and progesterone performed better than vehicle on a spatial memory task. Early initiation of estradiol and progesterone resulted in better performance than later initiation.
Vongher & Frye, 1999^85^ ^,^ ^*^	Long Evans rats	Female	Rats given estradiol and progesterone performed better on water maze compared to rats given vehicle. Estradiol and progesterone improved choline acetyltransferase significantly compared to estradiol alone or vehicle.
Ishunina et al., 2002^46^ ^,^ ^$^	Human *post mortem*	Men and women	In women with AD, androgen receptors were significantly reduced in the NBM and vertical limb of the diagonal band of Broca compared to men with AD.
Shi et al., 2024^45^ ^,^ ^$^	Human	Men and women	Significant early changes in basal forebrain volume were observed between MCI and healthy controls for women only. Longitudinal reduction in volumes were seen only in healthy control women.
Smith et al., 2011^53^ ^,^ ^*^	Human	Women	The group receiving estradiol and progesterone showed increased cholinergic uptake in hippocampus and posterior cingulate versus estradiol alone or placebo.
Norbury et al., 2007^54^ ^,^ ^*^	Human	Women	Compared to premenopausal women, postmenopausal women had lower muscarinic acetylcholine receptor density. Estradiol users had higher muscarinic acetylcholine receptor density compared to non‐users.
Dumas et al., 2006^65^ ^,^ ^*^	Human	Women	Estradiol pretreatment attenuated cognitive impairment under anticholinergic challenge more than placebo.
Dumas et al., 2012^66^ ^,^ ^*^	Human	Women	There was increased cortical activation during working memory task under anticholinergic challenge. Estradiol treatment reduced cortical activation compared to placebo.
Dumas et al., 2008^67^ ^,^ ^*^	Human	Women	Estradiol pretreatment attenuated cognitive impairment under anticholinergic challenge in younger but not older women.
Conley et al., 2022^78^ ^,^ ^*^	Human	Women	Higher endorsement of cognitive complaints was associated with worse performance under anticholinergic blockade.
Espeland et al., 2004^81^ ^,^ ^*^	Human	Women	Women who received conjugated equine estrogen performed worse on the Modified Mini‐Mental State Examination.
Shumaker et al., 2004^82^ ^,^ ^*^	Human	Women	Treatment with conjugated equine estrogen with or without medroxyprogesterone caused higher dementia risk compared to placebo.
Sherwin & Grigorova, 2011^86^ ^,^ ^*^	Human	Women	Combined treatment with conjugated equine estrogen and micronized progesterone resulted in better working memory performance versus other treatments.
Conley et al., 2024^88^ ^,^ ^*^	Human	Women	Micronized progesterone interferes with the ability of estradiol to mitigate anticholinergic blockade
**Dopamine**			
Andén et al., 1964^89^ ^,^ ^*^	Albino and hooded rats	Unspecified	Dopaminergic neurons originating in the substantia nigra terminate in the striatum.
Swanson, 1982^90^ ^,^ ^*^	Albino rats	Male	Independent populations of aminergic and non‐aminergic neurons in the VTA project to nuclei in the telencephalon, diencephalon, and brainstem.
Kritzer & Creutz, 2008^105^ ^,^ ^$^	Sprague–Dawley rats	Male and female	Expression of androgen and ERs in mesocortical projection neurons varied by region, cell, and sex. 30% of mesocortical projection neurons were dopaminergic in males; in females the proportion was 54%.
Walker et al., 2000^106^ ^,^ ^$^	Sprague–Dawley rats	Male and female	Electrical stimulation of medial forebrain bundle in vivo and ex vivo elicited significantly more DA release in the caudate nucleus of females than males. DA reuptake was also faster in females, while DA receptor affinity was no different between males and females.
Xiao & Becker, 1994^109^ ^,^ ^$^	Holtzman rats	Male and female	Female rats had significantly higher extracellular striatal DA concentrations in proestrus and estrus than in diestrus or after gonadectomy. Orchidectomy had no effect on extracellular striatal DA concentrations in male rats.
Locklear et al., 2017^111^ ^,^ ^†^	Sprague–Dawley rats	Male and female	Orchidectomy led to higher rates of burst firing in VTA neurons of male rats in a testosterone‐sensitive, estrogen‐insensitive manner. Orchidectomy also increased burst firing in PFC neurons that project to the VTA in a testosterone‐sensitive manner. Gonadally intact males and females exhibited no differences in burst firing dynamics of VTA neurons.
Kokras et al., 2018^112^ ^,^ ^$^	Wistar rats	Male and female	Sex differences in the open field test and forced swim test were eliminated by gonadectomy but not aromatase inhibition. Aromatase inhibition did, however, decrease NE and DA turnover rates in the hippocampus and PFC of male and female rats. Aromatase inhibition also enhanced serotonergic turnover rates in the hippocampus of males and females, irrespective of gonadectomy.
Aubele & Kritzer, 2011^113^ ^,^ ^*^	Sprague–Dawley rats	Male	PFC extracellular DA levels were significantly lower in male rats 4 days after gonadectomy, but supplementation of both estradiol and testosterone to gonadectomized males maintained PFC DA at control levels. PFC extracellular DA levels were significantly higher in male rats 28 days after gonadectomy compared to intact controls. Testosterone, but not estradiol, supplementation maintained PFC DA concentrations at control levels for long‐term gonadectomized rats. Neither short‐ nor long‐term gonadectomy affected motor cortex DA concentrations.
Aubele & Kritzer, 2012^114^ ^,^ ^*^	Sprague–Dawley rats	Male	Findings showed that VTA neurons that project to the PFC express androgen receptors in male rats. PFC infusion of an AMPA receptor antagonist led to lower levels of PFC DA in intact males and gonadectomized males treated with testosterone propionate, but not in gonadectomized males that received either estradiol or vehicle. PFC infusion of NMDA receptor antagonists led to higher levels of PFC DA in intact males and gonadectomized males treated with testosterone propionate, but decreased PFC DA levels in gonadectomized male rats that received either estradiol or vehicle.
Nobili et al., 2017^122^ ^,^ ^*^	Tg2576 mice	Male	Tg2576 mice exhibited an age‐dependent loss of dopaminergic VTA (but not SNpc) neurons at pre‐plaque stages. VTA neuron loss was associated with lower DA release in the hippocampus and nucleus accumbens shell and impairment in CA1 synaptic plasticity, memory performance, and food reward processing.
Nam et al., 2018^124^ ^,^ ^*^	CD11b‐expressing cells from C57Bl/6 mice	Unspecified	DA was able to modulate metal ions, metal‐free Aβ, metal‐bound Aβ, and reactive oxygen species through its own oxidative transformations. Moreover, DA combatted oxidative stress by reducing induction of inflammatory mediators and upregulating expression of the antioxidant‐producing enzyme heme oxygenase‐1.
Lansdell et al., 2023^126^ ^,^ ^$^	5xFAD mice	Male and female	Female 5xFAD mice had a higher striatal plaque density and exhibited more hyperactivity than males. Hyperactivity was correlated with higher striatal plaque burden and changes in DA signaling in the dorsal striatum.
Habibi et al., 2024^130^ ^,^ ^*^	Wistar rats	Female	Ovariectomy and AD‐like phenotype caused cognitive impairment, altered protein expression, and decreased antioxidant marker levels compared to controls. Estrogen therapy and/or treatment with young plasma therapy restored these outcomes to control levels in ovariectomized rats.
Pacelli et al., 2015^131^ ^,^ ^*^	Neurons from TH‐GFP mice cultured in vitro	Unspecified	Compared to VTA or olfactory bulb dopaminergic neurons, those of the SNpc had a higher basal rate of mitochondrial oxidative phosphorylation, a smaller reserve capacity, and an elevated level of oxidative stress, in part due to the complexity of their axonal arborizations.
Pissadaki & Bolam, 2013^132^ ^,^ ^*^	Digitally simulated neurons modeled after rat dopaminergic neurons	Male	Energy cost of axon potential propagation and recovery of the membrane potential increased with the size and complexity of the axonal arbor. This suggests that the complex arborizations of SNpc neurons heighten energy requirements. Electrophysiological properties of the model were based on findings from adult male rats.
Manza et al., 2022^102^ ^,^ ^$^	Human	Men and women	Women have higher DA release in ventral striatum relative to men. No sex differences in dorsal caudate DA release or in D2/3 receptor density in either ventral striatum or dorsal caudate.
Kaasinen et al., 2001^99^ ^,^ ^$^	Human	Men and women	Women have higher D2 receptor density in frontal cortex compared to men.
Pohjalainen et al., 1998^95^ ^,^ ^$^	Human	Men and women	Women have lower striatal D2 receptor affinity compared to men. There was no sex difference in D2 receptor density.
Laakso et al., 2002^100^ ^,^ ^$^	Human	Men and women	Women had higher DA synthesis capacity in caudate, trending in putamen, compared to men.
Munro et al., 2006^104^ ^,^ ^$^	Human	Men and women	Men had higher DA release compared to women in ventral striatum, caudate, and putamen. No sex differences in baseline D2/3 receptor density. Men also rated the positive effects of amphetamine higher than women.
Jacobs & D'Esposito, 2011^107^ ^,^ ^*^	Human	Women	Effects of estradiol on an N‐back working memory task depend on baseline DA, measured with a catechol‐O‐methyltransferase polymorphism as a proxy for prefrontal DA function.
Pirskanen et al., 2005^127^ ^,^ ^$^	Human	Men and women	Several genotypes at the ERβ gene locus were more frequent in women with AD relative to healthy women controls, while genotype frequency did not differ for men AD patients compared to men controls.
Oveisgharan et al., 2023^128^ ^,^ ^$^	Human *post mortem*	Men and women	ER DNA methylation and RNA expression in dorsolateral PFC related to cognitive decline and AD pathology. Results were most pronounced in women, less robust in men.
Brown et al., 2012^97^ ^,^ ^$^	Human	Men and women	Men smokers had lower striatal D2 receptor availability compared to both women smokers and men non‐smokers, while women smokers and women non‐smokers did not differ in receptor availability.
Iwaki et al., 2021^98^ ^,^ ^$^	Human	Men and women	Across several cross‐sectional and longitudinal cohorts, female Parkinson's disease patients were more likely to develop dyskinesia, whereas men were more likely to have more difficulties in daily living over time and more cognitive impairment.
Lavalaye et al., 2000^101^ ^,^ ^$^	Human	Men and women	Women had higher striatal DA transporter density compared to men.
Mozley et al., 2001^103^ ^,^ ^$^	Human	Men and women	Women had higher DA transporter availability in caudate, which related to better verbal learning performance.
Taylor et al., 2023^108^ ^,^ ^†^	Human	Men and women	Women who use hormonal contraceptives had higher DA synthesis capacity compared to participants who did not use hormonal contraceptives. Higher DA synthesis capacity was related to greater cognitive flexibility across both groups.
Kemppainen et al., 2003^115^ ^,^ ^*^	Human	Men and women	In AD patients, hippocampal D2/3 receptor density was lower compared to controls. Higher receptor density was associated with better memory performance in both groups.
Nagaraja & Jayashree, 2001^116^ ^,^ ^*^	Human	Men and women	The DA agonist piribedil improved cognition in older individuals with MCI.
Ciampa et al., 2024^118^ ^,^ ^†^	Human	Men and women	A genetic polymorphism in the DA transporter gene and a polymorphism in the BDNF gene interacted to predict greater cross‐sectional and longitudinal PET measures of Aβ and tau pathology and hippocampal atrophy.
Roussotte et al., 2015^119^ ^,^ ^*^	Human	Men and women	AD patients were significantly more likely than patients with MCI or healthy controls to carry a DA transporter gene polymorphism associated with greater expression in vitro. This allele was associated with poorer cognitive performance and faster ventricular expansion.
Beach et al., 2012^120^ ^,^ ^*^	Human *post mortem*	Men and women	A higher striatal plaque density score was highly correlated with a Braak neurofibrillary tangle stage of V or VI and presence of dementia.
Stratmann et al., 2016^121^ ^,^ ^*^	Human *post mortem*	Men and women	Tau pathology was present in subcortical dopaminergic nuclei in Braak stage 0 and I AD patients.
**Norepinephrine**			
Evans et al., 2024^168^ ^,^ ^$^	5xFAD mice	Male and female	LC chemogenetic silencing and chronic beta‐blocker treatment increased central nervous system inflammation and impaired memory. Microglia adrb2 knockdown attenuated inflammation in females only.
Braun & Feinstein, 2019^173^ ^,^ ^*^	5xFAD mice	Male	Vindeburnol treatment in male 5xFAD mice reduced amyloid burden, normalized exploratory and anxiety‐like behavior, and likely restored LC–NA function through cAMP‐dependent BDNF signaling.
Kelly et al., 2019^169^ ^,^ ^*^	Tg344‐AD rats	Male and female	LC fiber loss in Tg344‐AD rats led to spatial and working memory deficits, blood–brain barrier disruption, increased Aβ, and vascular remodeling.
Ross et al., 2019^189^ ^,^ ^$^	CRH overexpressing mice	Male and female	CRH overexpression increased Aβ_1‐__42_ accumulation in LC somatodendrites and PFC terminals. In the PFC, Aβ in NE axon terminals was increased in female CRH overexpressing mice. Additionally, swollen microvessels with lipid‐laden vacuoles were detected in female CRH overexpressing mice, a sign of blood–brain barrier dysfunction.
Kummer et al., 2014^170^ ^,^ ^*^	APP/PS1 crossed with Ear2 KO mice	Male and female	Genetic LC neuron loss in APP/PS1 mice caused NE depletion, synaptic plasticity deficits, and spatial memory impairments independent of Aβ pathology. NE supplementation partially reversed deficits.
Hammerschmidt et al., 2013^171^ ^,^ ^*^	APP/PS1 crossed with DBH KO mice	Male and female	Genetic NE depletion in APP/PS1 mice exacerbated spatial memory deficits and long‐term potentiation impairment without increasing Aβ or causing LC neurons loss.
Kalinin et al., 2007^172^ ^,^ ^*^	V717F‐APP mice	Male	LC lesions in male V717F‐APP mice increased Aβ plaque load, glial activation, and amyloid precursor protein C‐terminal fragments. NE deficiency reduced neprilysin activity and microglial Aβ clearance.
O'Neil et al., 2007^161^ ^,^ ^*^	APP/PS1 mice	Female	Aged female APP/PS1 mice showed a 24% loss of LC neurons and fiber loss in cortex and hippocampus, which was absent in younger mice.
Heneka et al., 2006^160^ ^,^ ^*^	APP23 mice	Female	LC degeneration in female APP23 mice elevated glial activation, Aβ plaque load, neuronal death, and cognitive deficits.
Flynn et al., 2025^179^ ^,^ ^$^	LC‐htauE14 in TH‐Cre rats	Male and female	Probiotic supplementation improved spatial learning, reduced inflammation in htauE14 rats, and inhibited GSK‐3β activity in female rats. Female htauE14 rats had greater GFAP expression. Microbiome analyses were conducted in females only.
Omoluabi et al., 2025^181^ ^,^ ^$^	LC‐htauE14 in TH‐Cre rats	Male and female	LC htauE14 caused mitochondrial and memory deficits, rescued by L‐type calcium channel blockade. snRNA‐seq revealed alterations linked to synaptic and ion channel function in males and changes in metabolism and development in females. DBH was upregulated in the male LC.
Kelberman et al., 2023^177^ ^,^ ^*^	TgF344‐AD rats	Male and female	LC neurons showed early hyperactivity in foot‐shock induced firing, followed by hypoactivity with age.
Omoluabi et al., 2021^182^ ^,^ ^*^	LC‐htauE14 in TH‐Cre rats	Male and female	Phasic LC activation prevented tau‐induced behavioral deficits and LC axonal degeneration. Stress‐like tonic patterns worsened LC neuronal health and was associated with depressive behavior.
Ghosh et al., 2019^180^ ^,^ ^*^	LC‐htauE14 in TH‐Cre rats	Male and female	LC hyperphosphorylated tau model mimicking human pretangle origin. LC htau expression caused age‐dependent olfactory learning deficits, axonal loss, and tau spread.
Ahnaou et al., 2019^175^ ^,^ ^*^	P301L mice with LC K18 fibrils	Male	LC tau seeding induced early, progressive disruption of hippocampal CA1 network activity without detectable tau spread.
Rorabaugh et al., 2017^176^ ^,^ ^*^	TgF344‐AD rats	Male and female	LC pretangle tau accumulation occurred before hippocampal or cortical tau pathology, accompanied by axonal loss and NE deficits without frank neuron loss. Chemogenetic LC activation restored reversal learning.
Iba et al., 2015^174^ ^,^ ^*^	PS19 mice with LC tau fibrils	Male and female	LC tau seeding led to early ipsilateral tau pathology and neuron loss by 6–12 months, with contralateral LC showing tau clearance. Tau spread followed LC connectivity but spared hippocampus and entorhinal cortex.
Iversen et al., 1983^190^ ^,^ ^*^	Human *post mortem*	Men and women	Patients with AD dementia displayed ≈ 60% reduction in LC cells compared to controls.
Pearson et al., 1983^191^ ^,^ ^*^	Human *post mortem*	Unspecified	Localization of catecholaminergic neurons in the human brainstem by using tyrosine hydroxylase immunocytochemistry.
Kemper et al., 1987^192^ ^,^ ^*^	Human *post mortem*	Unspecified	Localization of adrenergic and noradrenergic neurons in the human brainstem by using DBH immunocytochemistry.
German et al., 1988^193^ ^,^ ^*^	Human *post mortem*	Unspecified	Computer‐assisted estimation of the number of LC cells and their spatial distribution in the human brainstem.
Chan‐Palay & Asan, 1989^194^ ^,^ ^*^	Human *post mortem*	Men and women	Patients with AD displayed a rostro‐caudal gradient of LC neuronal loss, while patients with Parkinson's disease displayed uniform and more severe loss of LC neurons.
Chan‐Palay & Asan, 1989^195^ ^,^ ^*^	Human *post mortem*	Men and women	Computer‐assisted quantification of the morphology and distribution of LC neurons, with a description of four classes of LC neurons (large multipolar, large elliptical bipolar, small multipolar, and small ovoid bipolar).
Baker et al., 1989^196^ ^,^ ^*^	Human *post mortem*	Unspecified	Computer‐assisted estimation of the number of LC and subcoeruleus cells in the human brainstem.
Chan‐Palay, 1991^197^ ^,^ ^*^	Human *post mortem*	Men and women	Patients with Parkinson's disease displayed severe loss of LC neurons compared to controls.
German et al., 1992^198^ ^,^ ^*^	Human *post mortem*	Men and women	Patients with AD and Down syndrome displayed a rostro‐caudal gradient of LC neuronal loss, while patients with Parkinson's disease displayed uniform loss of LC neurons.
Kubis et al., 2000^199^ ^,^ ^*^	Human *post mortem*	Men and women	Scarce LC neuronal loss is observed across normal aging.
Busch et al., 1997^200^ ^,^ ^*^	Human *post mortem*	Women	LC neurons showed early susceptibility to neurofibrillary tangle formation, but significant neuronal loss appeared at least 25 years later.
Vijayashankar & Brody, 1979^201^ ^,^ ^*^	Human *post mortem*	Men	After age 63, LC neuronal population decreased by 40%.
Mouton et al., 1994^202^ ^,^ ^*^	Human *post mortem*	Men	Older age was not associated with the total number of pigmented LC neurons or their size.
Ohm et al., 1997^203^ ^,^ ^*^	Human *post mortem*	Men	Total number of LC cells was not associated with older age or early neurofibrillary changes. There were no hemisphere differences in the number of LC neurons.
Mann & Yates, 1979^204^ ^,^ ^†^	Human *post mortem*	Men and women	Older age was associated with a 5%–10% decrease in LC nucleolar volume and a decrease in melanin content. No sex differences were observed in LC nucleolar volume or melanin content.
Wree et al., 1980^205^ ^,^ ^$^, ^†^	Human *post mortem*	Men and women	Older age was associated with a loss of LC neurons. The left side of the LC contained more neurons than the right. No sex differences were observed in total number of LC neurons, but loss of LC cells begins earlier in men than women.
Tomlinson et al., 1981^206^ ^,^ ^$^	Human *post mortem*	Men and women	Controls displayed a gradual loss of LC neurons from middle to old age. Patients with AD displayed more severe LC neuronal loss, particularly when Aβ was present in the neocortex. There were slightly higher numbers of LC neurons in control women than control men.
Braak & Del Tredici, 2011^138^ ^,^ ^*^	Human *post mortem*	Men and women	38 out of the 42 cases aged < 30 displayed pretangle tau material in the LC; 19 of those cases displayed pretangle tau material in the LC in the absence of pretangle tau material in the transentorhinal region.
Braak et al., 2011^5^ ^,^ ^*^	Human *post mortem*	Men and women	By age 40, pretangle tau material was present in the LC in all 2332 cases examined.
Buchman et al., 2012^207^ ^,^ ^*^	Human *post mortem*	Men and women	LC neuronal density was associated with the severity of global parkinsonism proximate to death.
Dugger et al., 2012^208^ ^,^ ^*^	Human *post mortem*	Men and women	LC neuronal loss was more pronounced in Lewy body dementia patients than in AD or controls. LC neurons were more vulnerable to α‐synuclein in Lewy body dementia and to tau pathology in AD.
Keren et al., 2015^209^ ^,^ ^*^	Human *post mortem*	Men and women	Cross‐validation of a 7T LC‐sensitive MRI sequence with histological localization of LC neuromelanin cells.
Eser et al., 2018^210^ ^,^ ^*^	Human *post mortem*	Men and women	Progressive supranuclear palsy and corticobasal degeneration patients showed more tau inclusions in LC neurons than AD patients, but AD patients displayed more extreme LC neuronal loss.
Theofilas et al., 2018^211^ ^,^ ^*^	Human *post mortem*	Men and women	Intraneuronal caspase activation and macroautophagy markers in the LC emerge in early Braak stages and increase with stage progression.
Ehrenberg et al., 2018^1^ ^,^ ^*^	Human *post mortem*	Men and women	Neuropsychiatric symptoms of agitation, anxiety, appetite changes, depression, and sleep disturbances emerged as early as in Braak I–II stages and correlated with tau but not Aβ pathology.
Oh et al., 2019^212^ ^,^ ^*^	Human *post mortem*	Men and women	AD patients displayed substantial loss of LC neurons, which was not observed in progressive supranuclear palsy and corticobasal degeneration patients.
Zahola et al., 2019^213^ ^,^ ^*^	Human *post mortem*	Men and women	A reduction in secretagogin expression in the LC was observed starting from Braak stages III–IV and paralleled the loss of tyrosine hydroxylase.
Tong & Chen, 2021^214^ ^,^ ^*^	Human *post mortem*	Men and women	LC neuronal loss was associated with the occurrence of dyskinesia in advanced Parkinson's disease patients.
Oh et al., 2022^10^ ^,^ ^*^	Human *post mortem*	Men and women	AD patients showed greater loss of LC neurons compared to progressive supranuclear palsy. Higher number of LC neurons was associated with shorter total sleep time and greater REM latency.
Murray et al., 2022^215^ ^,^ ^*^	Human *post mortem*	Men and women	Lower LC neuronal density was associated with higher plasma p‐tau_181_ and higher plasma p‐tau_217_.
Gilvesy et al., 2022^6^ ^,^ ^*^	Human *post mortem*	Men and women	Dendritic atrophy was the first sign of degeneration of LC tau‐positive neurons. Tau pathology was more pronounced in the dorsal LC.
Torso et al., 2023^216^ ^,^ ^*^	Human *post mortem*	Men and women	LC hypopigmentation was associated with worse cortical diffusivity metrics.
Beardmore et al., 2024^217^ ^,^ ^*^	Human *post mortem*	Men and women	Increasing Braak stage was associated with LC neuronal loss, increased microglial markers, and reduction in neuromelanin.
Bueichekú et al., 2024^137^ ^,^ ^*^	Human *post mortem*	Men and women	LC tangle density was related to tangles in medial temporal lobe structures and in the inferior temporal cortex.
Fructuoso et al., 2025^218^ ^,^ ^*^	Human *post mortem*	Men and women	LC neuronal loss was most pronounced in AD and Parkinson's disease patients. AD patients further displayed endosomal alterations, while Parkinson's disease and Down syndrome patients exhibited lysosomal changes. Down syndrome patients showed elevated levels of a kinase involved in neurodegenerative processes.
Hary et al., 2025^219^ ^,^ ^*^	Human *post mortem*	Men and women	LC tau accumulation was present from Braak stages I and II. Tau pathology was most severe in the middle portion of the LC.
Theofilas et al., 2017^165^ ^,^ ^†^	Human *post mortem*	Men and women	LC volume decreased by 8.4% for each increase in Braak stages. LC neuronal population started to decrease from Braak stages III to VI. These effects were more pronounced in the middle and rostral LC. No age‐related changes were observed in Braak stages 0–I. No sex differences were observed in LC volume or in number of LC neurons.
Wilson et al., 2013^162^ ^,^ ^†^	Human *post mortem*	Men and women	Higher LC neuronal density was associated with slower rate of cognitive decline, while higher LC tangle density was associated with faster cognitive decline. LC neuronal density further moderated the association between Lewy bodies and cognitive decline. No sex differences were observed in number of LC neurons or in number of hyperphosphorylated tau‐positive LC neurons.
Wilson et al., 2013^220^ ^,^ ^†^	Human *post mortem*	Men and women	Higher density of Lewy bodies in the LC was associated with more depressive symptoms, while neither LC tangle density nor the number of LC tyrosine hydroxylase‐immunoreactive neurons were associated with depressive symptoms. No sex differences were observed in number of LC neurons or in number of hyperphosphorylated tau‐positive LC neurons.
Kelly et al., 2017^163^ ^,^ ^†^	Human *post mortem*	Men and women	MCI and AD patients displayed reductions in genes regulating mitochondrial function and neuritic/structural plasticity compared to controls. No sex differences were observed in number of LC neurons.
Kelly et al., 2021^221^ ^,^ ^†^	Human *post mortem*	Men and women	LC neuronal oxidative stress and pontine arteriolosclerosis differentiated healthy controls with high pathology from MCI patients. No sex differences were observed in number of LC neurons.
Jacobs et al., 2021^222^ ^,^ ^†^	Human *post mortem*	Men and women	MCI and AD patients exhibited greater LC tangle density compared to controls, but no overall group difference was observed for LC neuronal density. LC tangle density was associated with increased cortical Aβ density and Thal phase. No sex differences were observed in number of LC neurons or in LC tangle density. Low LC intensity was related to elevated tau and Aβ burden as well as AD‐related memory decline. No sex differences were observed in LC intensity.
Beckers et al., 2024^223^ ^,^ ^†^	Human *post mortem*	Men and women	Tangle density in the left versus right LC was equivalent across individuals with or without AD pathology, but neuronal density was higher in the left caudal LC among individuals with AD pathology. No sex differences were observed in number of LC neurons or in LC tangle density.
Ehrenberg et al., 2017^11^ ^,^ ^†^	Human *post mortem*	Men and women	Approximately 8% of LC neurons displayed hyperphosphorylated tau intraneuronal cytoplasmic inclusions in Braak stage 0. The proportion of tau‐positive LC neurons was higher in late Braak stages compared to early Braak stages. No sex differences were observed in number of hyperphosphorylated tau‐positive LC neurons.
Freeze et al., 2023^224^ ^,^ ^$^	Human *post mortem*	Men and women	LC hypopigmentation was associated with higher odds of cerebral amyloid angiopathy and arteriolosclerosis, independent of cortical AD pathology. Men had a higher probability of displaying LC hypopigmentation compared to women.
Van Egroo et al., 2024^225^ ^,^ ^$^	Human *post mortem*	Men and women	*Ante mortem* 24‐hour rest–activity rhythm fragmentation was associated with increased odds of LC hypopigmentation, particularly in individuals with cortical AD pathology and independently of comorbid pathologies. Men showed higher probability of LC hypopigmentation compared to women.
Trujillo et al., 2019^227^ ^,^ ^†^	Human	Men and women	Low macromolecular content in the LC contributed to contrast derived from 2D‐T1‐weighted turbo‐spin‐echo sequence with magnetization transfer contrast. No sex differences were observed in LC intensity.
Betts et al., 2017^226^ ^,^ ^†^	Human	Men and women	Description of a novel T1‐weighted fast low angle shot MR sequence at 3T to investigate the LC along its rostrocaudal axis. Older adults showed regional increase in maximum (but not median) LC intensity confined to the rostral third of the LC. No sex differences were observed in LC intensity.
Berger et al., 2023^237^ ^,^ ^†^	Human	Men and women	LC intensity was higher in older than younger adults, whereas LC activity during an oddball task showed no age‐related differences. No sex differences were observed in LC intensity.
Takahashi et al., 2015^238^ ^,^ ^†^	Human	Men and women	LC intensity was reduced in patients with MCI and AD compared to cognitively normal individuals. No sex differences were observed in LC intensity.
Calarco et al., 2022^239^ ^,^ ^†^	Human	Men and women	LC intensity showed no significant association with age and was not different between older adults with and without late‐life depression. LC intensity was correlated with cognitive performance. No sex differences were observed in LC intensity across any group.
Liu et al., 2019^240^ ^,^ ^†^	Human	Men and women	A quadratic relationship between LC intensity and age was described, with a peak occurring at ≈ 60 years. Age‐related decline in LC intensity was restricted to the rostral part of the LC. No sex differences were observed in LC intensity.
Shibata et al., 2006^241^ ^,^ ^†^	Human	Men and women	A quadratic relationship between LC intensity and age was described, with a peak occurring at ≈ 59 years. No sex differences were observed in LC intensity.
Jacobs et al., 2023^166^ ^,^ ^†^	Human	Men and women	LC intensity started to decrease 12 years before clinical onset in presenilin‐1 E280A carriers. High LC intensity was associated with low cortical tau and high memory performance in carriers compared to non‐carriers. No sex differences were observed in LC intensity across any group.
Riley et al., 2025^242^ ^,^ ^$^	Human	Men and women	A quadratic relationship between LC intensity and age was described, with a peak occurring at ≈ 60 years. Greater rostral LC intensity was associated with greater fluid cognition in participants above the 50th percentile for age. Rostral LC intensity was higher in women compared to men and in Black participants.
Bachman et al., 2023^243^ ^,^ ^$^	Human	Men and women	Five weeks of heart rate variability biofeedback training decreased LC intensity in young adults, which was further associated with reduced sympathetic nervous system activity. Higher LC intensity was observed in young women compared to young men.
Bachman et al., 2021^244^ ^,^ ^$^	Human	Men and women	In older adults, lower LC intensity was related to greater cortical thickness in parietal, frontal, and occipital regions. Higher LC intensity was observed in women compared to men.
Galgani et al., 2025^245^ ^,^ ^$^	Human	Men and women	Women showed higher LC intensity than men in cognitively normal and MCI participants. Men exhibited a positive association between LC intensity and volume in frontotemporal area compared to women.
Galgani et al., 2023^246^ ^,^ ^$^	Human	Men and women	Rostral LC intensity was reduced in AD and MCI who converted to dementia during follow‐up. Higher LC intensity was observed in women compared to men in cognitively normal and MCI participants who later converted to dementia.
Clewett et al., 2016^247^ ^,^ ^$^	Human	Men and women	Higher verbal intelligence and higher cognitive reserve were associated with higher LC intensity in older adults. LC intensity was higher in older adults compared to younger adults and lower in women compared to men.
Beckers et al., 2024^248^ ^,^ ^†^	Human	Men and women	At high levels of astrocyte reactivity, lower arborization complexity in LC neurites was associated with lower arborization complexity in frontotemporal cortical and subcortical regions. No sex differences were observed.
Wearn et al., 2024^249^ ^,^ ^†^	Human	Men and women	High isodendritic core microstructural integrity derived from a multiparametric mapping protocol was associated with higher white matter neurite density and arborization complexity in limbic tracts. No sex differences were observed in LC microstructure.
Bennett et al., 2024^250^ ^,^ ^†^	Human	Men and women	Maximum LC intensity was more sensitive to age than mean LC intensity and LC microstructural integrity. Higher LC microstructural integrity was associated with more consistent memory performance. Females showed lower LC microstructural integrity compared to males but no sex differences in LC intensity were observed. Age‐related changes in LC intensity and microstructure were larger in men than women.
Zhang et al., 2016^251^ ^,^ ^$^	Human	Men and women	The LC and VTA/SNpc showed both shared and distinct functional connectivity patterns, with the LC enhancing attentional orienting and sensorimotor responses to salient stimuli. Lower LC functional connectivity was observed with the hippocampus, parahippocampus, and middle temporal gyrus in women compared to men.
Um et al., 2023^252^ ^,^ ^$^	Human	Men and women	At elevated frontal Aβ burden, higher LC functional connectivity was observed with the right postcentral gyrus and right supramarginal gyrus in women compared to men.
Filkowski et al., 2017^253^ ^,^ ^$^	Human	Men and women	Women showed higher functional task activation in the bilateral amygdala, hippocampus, and regions of the dorsal midbrain including the periaqueductal gray/superior colliculus and LC compared to men.
Ludwig et al., 2024^254^ ^,^ ^†^	Human	Men and women	Older adults showed increased LC responses during emotional and task‐related salient events compared to younger adults. No sex differences were observed in LC functional activation in response to emotional salience, task‐related salience, or memory performance.
Kilpatrick et al., 2025^†^	Human	Men and women	Higher left LC functional connectivity with the executive control network was observed in women compared to men, and was mostly driven by differences between men and premenopausal women.
Koops et al., 2025^256^ ^,^ ^†^	Human	Men and women	Higher LC metabolism was observed during preclinical stages, as defined by the presence of elevated Aβ burden, but was lower when elevated tau and cognitive impairment are present. This increase in metabolism was associated with attenuated memory decline, particularly in participants with high Aβ burden. LC metabolism decreased with age and was higher in women compared to men.
**Serotonin**			
Rosecrans, 1970^285^ ^,^ ^$^	CD rats and Swiss albino mice	Male and female	Females from each species have both a more functional 5‐HT system and a higher rate of rearing.
Biegon & McEwen, 1982^306^ ^,^ ^*^	Sprague–Dawley rats	Female	Estradiol may have a direct effect on modifying 5‐HTR availability, while exerting a slow effect on the same receptors through an interaction with intracellular estrogen receptors.
Uphouse et al., 1986^308^ ^,^ ^*^	F344 rats	Female	Differential binding of 5‐HT to cortical membranes during the estrous cycle is most likely the result of 5‐HT_1_Rrather than 5‐HT_2_R. Changes in 5‐HT binding in a brain area nearly devoid of sex steroid receptors suggest that the hormonal fluctuations accompanying the female estrous cycle influence brain areas other than those classically thought to regulate neuroendocrine function.
Carlsson & Carlsson, 1988^284^ ^,^ ^$^	Sprague–Dawley rats	Male and female	5‐HT levels were significantly higher in females than males in the brainstem and limbic forebrain and tended to be so in the cortex. 5‐HIAA levels were significantly higher in females in all five brain regions.
Haleem et al., 1990^283^ ^,^ ^$^	Sprague–Dawley rats	Male and female	5‐HT and/or 5‐HIAA concentrations tended to be higher in female rats than in males with only substantial differences in the hippocampus where female values were 34% and 36% higher, respectively. This is consistent with 5‐HT synthesis being 53% greater in female than male hippocampus.
Sumner & Fink, 1993^309^ ^,^ ^*^	Wistar rats	Female	Results showed a 290% increase in 5‐HT_2_R mRNA in the DRN and a 25% decrease in the medial preoptic area in response to a single injection of estradiol, implying a mechanism for estrogen's positive feedback on luteinizing hormone and prolactin release.
Sumner & Fink, 1995^305^ ^,^ ^*^	Wistar rats	Female	Estradiol significantly increases density of 5‐HT_2__A_R in the cerebral cortex and nucleus accumbens of female rats.
Borisova et al., 1996^299^ ^,^ ^$^	Wistar rats	Male and female	Female rats showed higher 5‐HT content and uptake in the anterior and middle hypothalamus compared to males. Neonatal castration of males eliminated this difference by increasing 5‐HT levels to those seen in females.
Fink et al., 1996^304^ ^,^ ^*^	Wistar rats	Female	Estrogen stimulates an increase in D2 receptors in the striatum and density of 5‐HT_2A_R binding sites in areas of the brain concerned with the control of mood, mental state, and behavior. This may explain the efficacy of estrogen therapy or 5‐HT reuptake blockers in the treating of depressive symptoms of the premenstrual syndrome and indicates sex differences in schizophrenia may be due to estrogen by way of 5‐HT_2A_R.
McQueen et al., 1997^311^ ^,^ ^*^	Wistar rats	Female	Insertion of estradiol benzoate in ovariectomized rats increases the number of cells that expressed *Slc6a4* mRNA in the DRN and increased SERT‐binding sites in lateral septum, basolateral amygdala, ventral nucleus of thalamus, and ventromedial hypothalamic nucleus. SERT‐binding sites in these rats were 15% lower in central periaqueductal gray.
Holschneider et al., 1998^296^ ^,^ ^*^	Sprague–Dawley rats	Female	High‐dose estrogen replacement in ovariectomized rats reduced monoamine oxidase activity, an enzyme responsible for 5‐HT degradation, in a tissue‐specific manner, decreasing monoamine oxidase‐B in peripheral organs and monoamine oxidase‐A in the hypothalamus and amygdala.
Zhang et al., 1999^301^ ^,^ ^$^	Sprague–Dawley rats	Male and female	Male rats showed higher *Htr1a* mRNA in the hypothalamus and amygdala but lower levels in the hippocampus compared to females. *Htr2a* mRNA was mostly similar across sexes, except for lower expression in the female ventromedial hypothalamus, while receptor binding was higher in females’ hippocampus. Gonadectomy in males increased H*tr1a* and decreased *Htr2a* mRNA, effects reversed by testosterone. These findings suggest that testosterone regulates 5‐HTR expression and may contribute to sex differences in mood and stress responses.
Lu et al., 2001^290^ ^,^ ^*^	Sprague–Dawley rats	Female	DRN 5‐HT neurons projecting to the medial preoptic area in female rats express ERβ, but not ERα. Number of ERβ‐expressing neurons do not differ between ovariectomized and estradiol‐treated groups.
Gundlah et al., 2001^291^ ^,^ ^*^	Rhesus macaques	Female	Most 5‐HT (SERT positive) neurons in DRN and median raphe nucleus express ERβ mRNA and protein. ERβ expression remained stable regardless of the ovarian hormone status.
Klink et al., 2002^286^ ^,^ ^$^	Sprague–Dawley rats	Male and female	The DRN 5‐HT systems differ significantly between sexes and undergo changes during pregnancy and the postpartum period.
Sheng et al., 2004^298^ ^,^ ^$^	Wistar rats and C57Bl/6 mice	Male and female	Sex steroids may modulate the affective regulation of the serotonergic system through ERα and/or ERβ in 5‐HT neurons of the mouse rostral DRN, but not through androgen receptors. Such effects may differ by sex and species, as suggested by the prominent sex differences in androgen receptor expression and prominent species differences in ERα and ERβ expression.
Imwalle et al., 2005^294^ ^,^ ^*^	ERβ KO mice	Female	Female mice lacking ERβ showed higher anxiety‐like behavior and reduced 5‐HT levels in the bed nucleus of the stria terminalis, preoptic area, and hippocampus compared to wild‐type females.
Bertrand et al., 2005^310^ ^,^ ^*^	Aromatase KO mice	Female	Low estrogen conditions, induced by ovariectomy or aromatase KO, enhanced SERT function in the hippocampus despite reduced SERT density, leading to heightened sensitivity to fluoxetine.
Donner & Handa, 2009^292^ ^,^ ^*^	Sprague–Dawley rats	Female	Activation of ERβ in DRN increased *Tph2* mRNA expression, enhancing 5‐HT synthesis in female rats. Systemic ERβ agonist treatment led to anxiolytic effects while direct DRN infusion promoted active stress‐coping without changing anxiety levels.
Bethea et al., 2011^316^ ^,^ ^*^	Japanese macaques	Female	Three years after ovariectomy, female macaques showed reductions in 5‐HT neurons, as well as *Fev*, *Tph2*, *SERT*, and *5‐HT_1A_* mRNA expression.
Hiroi & Handa, 2013^293^ ^,^ ^*^	RN46A‐B14 cell line derived from embryonic rat medullary raphe cells and mouse‐derived hippocampal cell line HT‐22	Unspecified	Estradiol and ERβ agonism increased DRN *Tph2* expression through an estrogen response element within its promoter.
Philippe et al., 2022^303^ ^,^ ^†^	Sprague–Dawley rats	Male and female	Based on the hypothermal and corticosteroid responses to 8‐OH‐DPAT, the data suggest that stress habituation is met by an increase in the sensitivity of presynaptic 5‐HT_1A_R in males, and by an increase in the sensitivity of a population of postsynaptic receptors in both sexes.
Goel et al., 2014^302^ ^,^ ^$^	Sprague–Dawley rats	Male and female	Females show a greater hypothalamic‐pituitary‐adrenal axis response compared to males. The 5‐HT_1A_R agonist, WAY, reduced the corticosterone response in males but not females. WAY administration increased total Fos activation in the DRN, but only in males. There was a negative correlation between estrogen and Fos responses within tryptophan hydroxylase‐positive cells in the DRN of WAY‐administered females and a positive correlation found between estrogen and *Htr1a* mRNA expression localized to the region of the zona incerta in close proximity to the paraventricular nucleus of the hypothalamus.
Castañé et al., 2015^329^ ^,^ ^*^	5‐HT_2A_R KO and “wild‐type” mice of unstated lineage	Male	Fluoxetine reduced immobility in the tail suspension test in both wild‐type and 5‐HT_2A_R KO mice, but impaired memory in the novel object recognition test only in wild‐type mice. Both genotypes showed similar increases in extracellular 5‐HT, but fluoxetine enhanced pyramidal neuron activity in 5‐HT_2A_R KO mice only. 5‐HT_2A_R may contribute to fluoxetine‐induced memory deficits by disrupting the excitatory‐inhibitory balance in the PFC.
Cao et al., 2014^297^ ^,^ ^*^	Tph2‐CreER mice crossed with Esr(fl/fl) mice	Female	Estrogen replacement suppressed binge‐like eating in ovariectomized females by activating DRN 5‐HT neurons through ERα and inhibition of small conductance calcium‐activated potassium channel currents.
Breitinger et al., 2001^327^ ^,^ ^*^	NIE‐115 mouse neuroblastoma cells	Unspecified	The mechanism of the channel‐opening process, receptor desensitization, and receptor inhibition by nicotine, cocaine, and fluoxetine were investigated. Two different forms of the 5‐HT_3_R, each with a different desensitization rate, were observed.
He et al., 2023^295^ ^,^ ^$^	Esr2(fl/fl) mice	Male and female	DRN specific ERβ deletion increased anxiety‐like behavior only in females but reduced passive coping in males.
Khan et al., 2023^178^ ^,^ ^†^	htau mice	Male and female	Depressive‐like symptoms were observed at 4 months in both sexes, and hyperlocomotion observed in males. These changes coincided with lower density of 5‐HT neurons, downregulation of 5‐HT markers, reduced excitability of 5‐HT neurons, and hyperphosphorylated tau in the DRN. Inflammatory markers were upregulated in the DRN, which may promote tau phosphorylation and aggregation. Loss of 5‐HT innervation to the entorhinal cortex and dentate gyrus of the hippocampus was observed, and may have contributed to depressive‐like behaviors.
Tian et al., 2023^334^ ^,^ ^*^	5xFAD mice	Male and female	In 5xFAD mice, hippocampal 5‐HT release was markedly reduced despite only modest decreases in serotonergic fiber density or 5‐HT content. Oligomeric Aβ impaired 5‐HT release by inducing mitochondrial dysfunction in 5‐HT neurons.
Wang et al., 2023^340^ ^,^ ^*^	hAPP‐J20 mice crossed with ePet‐Cre mice	Male and female	5‐HT signaling is disrupted in the hippocampus of hAPP‐J20 mice, contributing to early hyperactivity of CA1 neurons. Activation of median raphe nucleus 5‐HT neurons reduced CA1 hyperexcitability and improved memory through 5‐HT_1A_R‐ and 5‐HT_3A_R‐dependent mechanisms.
Torres et al., 2024^129^ ^,^ ^$^	Esr1‐Cre, Esr2‐Cre, Tph2‐iCreER, Rosa26‐LSL‐tdTomato, ER‐EGFP, and ER‐ZsGreen mice	Male and female	In females, estrogen promotes higher baseline activity of DRN 5‐HT neurons, and suppresses their firing to alcohol, contributing to binge‐like drinking. In males, exogenous estrogen blunts alcohol‐induced activation of DRN 5‐HT neurons and alters expression of estrogen and 5‐HT‐related genes in DRN. Activation of ERα‐ or Erβ‐expressing DRN neurons reduces binge drinking in both sexes.
Murakawa et al., 2024^289^ ^,^ ^*^	Erβ‐iCre mice	Female	Excitation of DRN–ERβ neurons is necessary for the decline of lordosis after estrous. Fiber photometry showed DRN–ERβ neuronal activation in response to male intromission was significantly more prolonged on day 2 compared to day 1 of estrus. DRN–ERβ neuronal excitation serves as an inhibitory modulator and is responsible for the decline in receptivity during non‐estrous phases.
Khan et al., 2024^338^ ^,^ ^$^	DRN P301L Tau in ePet‐Cre mice	Male and female	Human P301L tau expression in DRN 5‐HT neurons led to anxiety‐like behavior in both sexes but females showed greater vulnerability exhibiting additional social disinhibition, MCI, and more severe disruption of 5‐HT neuron excitability.
Chen et al., 2024^339^ ^,^ ^*^	5xFAD, CaMKII‐Cre, and GAD2‐Cre mice	Male	In 5XFAD mice, DRN 5‐HT neuron excitability and projections to CA1 hippocampal neurons were reduced. Activating the DRN 5HT‐CA1 pathway and 5‐HT_1B_R or 5‐HT_4_R alleviated depressive‐like and cognitive symptoms by restoring synaptic plasticity.
Young et al., 1980^282^ ^,^ ^$^	Human	Men and women	Cerebrospinal fluid concentrations of 5‐HT metabolite 5‐HIAA and tryptophan were higher in women than men, suggesting greater 5‐HT metabolism in women.
Tejani‐Butt et al., 1995^314^ ^,^ ^*^	Human *post mortem*	Unspecified	SERT sites are decreased in the DRN (especially lateral wings), hippocampal CA2, and entorhinal cortex.
Biver et al., 1996^300^ ^,^ ^$^	Human	Men and women	Men had higher 5‐HT_2_R binding capacity than women, particularly in frontal and cingulate cortices.
Nishizawa et al., 1997^280^ ^,^ ^$^	Humans	Men and women	5‐HT synthesis was ≈ 52% higher in men compared to women.
Kugaya et al., 2003^307^ ^,^ ^*^	Human	Women	Estrogen replacement therapy enhanced 5‐HT_2A_R binding in right PFC and anterior cingulate regions of postmenopausal women. Receptor upregulation in the inferior frontal gyrus correlated with estradiol levels.
Thomas et al., 2006^313^ ^,^ ^*^	Human *post mortem*	Unspecified	SERT density is reduced in the PFC of AD patients compared to healthy elderly and depressed subjects. SERT levels are similar between AD patients with and without major depression or between control and depressed groups.
Ouchi et al., 2009^315^ ^,^ ^*^	Humans	Men and women	In AD patients, SERT binding in the striatum and other projection areas is reduced compared to healthy controls, regardless of depression status. Depressed AD patients showed greater SERT loss and lower SERT binding correlated with higher depression scores but not cognitive impairment. SERT binding potential levels were correlated with reduced glucose metabolism in the right dorsolateral PFC in AD patients.
Cirrito et al., 2011^318^ ^,^ ^*^	APP/PS1 hemizygous mice; humans	Female; men and women	In mice, SSRIs lowered interstitial fluid Aβ by 25% and chronic citalopram treatment halved plaque burden via an ERK‐dependent mechanism. In humans, prior antidepressant use correlated with lower cortical amyloid load.
Sheline et al., 2020^319^ ^,^ ^*^	Human	Men and women	Short‐term escitalopram treatment reduced cerebrospinal fluid Aβ levels by 9.4% compared to placebo in cognitively normal older adults, with greater effects in participants without preexisting amyloid accumulation.
Smith et al., 2017^312^ ^,^ ^*^	Human	Men	Individuals with MCI showed reduction in SERT availability across cortical, limbic, striatal, thalamic, and sensorimotor regions compared to matched controls. This decrease was more prominent than gray matter loss or blood flow reductions and correlated with poorer auditory verbal and visuospatial memory performance.
Pierson et al., 2025^337^ ^,^ ^$^	DRN P301L Tau in C57Bl/6 mice; human *post mortem*	Male and female; men and women	Tau pathology was detected in the DRN of controls (47.37%) and AD patients (90%). In mice, targeted DRN expression of human P301L tau induced depressive‐like behavior, neuronal hyperexcitability, and astrocyte activation in the DRN. Males showed broader affective disruption (reduced social interaction, reduced sucrose preference, hyperlocomotion). Females showed a selective social interaction deficit only.
Terstege et al., 2025^324^ ^,^ ^†^	Human	Men and women	The DRN of AD patients showed reduced metabolic activity. SSRI treatment is associated with lower plasma phosphorylated tau levels and improved DRN metabolism.
Mo et al., 2025^325^ ^,^ ^$^	Human	Men and women	SSRI use was associated with faster cognitive decline. Higher doses were associated with greater risk of dementia and mortality. Men displayed greater cognitive decline than women in multiple dementia variants.
**Corticotropin releasing hormone**			
Bangasser et al., 2017^188^ ^,^ ^$^	Tg2576 mice crossed with CRH overexpressing mice and CaMKII‐tTA mice, CRH overexpressing mice, and Tg2576 mice	Male and Female	Female triple transgenic mice have significantly increased levels of phosphorylated β‐secretase relative to male triple transgenic mice. Female CRH overexpressing mice exhibited significantly higher levels of phosphorylated tau in both soluble and insoluble fractions compared to female wild‐type mice and all male groups. No sex difference in phosphorylated β‐secretase was observed in the Tg2576 mice lacking CRH overexpression. Female triple transgenic mice had a greater number of Aβ plaques and worse short‐term memory compared
Campbell et al., 2015^375^ ^,^ ^*^	PSAPP mice crossed with CRH_1_ KO or heterozygous mice	Female	Partial and complete ablation of CRH_1_ reduced Aβ burden in the hippocampus, insular, rhinal, and retrosplenial cortices via decreased levels of Aβ peptides and Aβ C‐terminal fragments.
Dong et al., 2012^368^ ^,^ ^*^	Tg2576 mice crossed with CRH overexpressing mice and CaMKII‐tTA mice, CRH overexpressing mice crossed with CaMKII‐tTA, and Tg2576 mice	Unspecified	At 3–4 months, mice with hypothalamic‐pituitary‐adrenal axis overactivation developed a Cushingoid phenotype with elevated brain CRH and CRH_1_ levels. By 6 months, triple transgenic mice exhibited higher Aβ_1‐__42_ and plaque burden, the most severe dendritic loss, and severe working and contextual memory deficits compared to Tg2576 mice. Doxycycline treatment, which suppresses CRH transgene expression, prevented the increased Aβ deposition and improved working memory in triple transgenic mice and reversed increased anxiety‐like behaviors in double transgenic mice.
Dong et al., 2014^369^ ^,^ ^*^	Tg2576 mice	Male and female	Administration of the CRH_1_ antagonist antalarmin for 1 week significantly reduced Aβ_1‐42_ levels in isolated stressed mice. Administration of antalarmin for 6 months significantly decreased plasma corticosterone levels, Aβ_1‐42_ levels and Aβ plaque deposition in the cortex and hippocampus, and blocked the effects of isolation stress on anxiety and memory.
Kang et al., 2007^370^ ^,^ ^*^	Tg2576 Mice	Male and female	Acute restraint stress increased interstitial fluid Aβ levels by 32% and led to significantly higher hippocampal CRH levels immediately after stress. Corticosterone administration did not alter Aβ levels, but exogenous CRH increased Aβ levels in a dose‐dependent manner. A CRH_1_ antagonist blocked the acute stress‐induced increase in Aβ.
Hebda‐Bauer et al., 2013^411^ ^,^ ^$^	3xTg‐AD mice	Male and female	During early‐stage pathology, 3xTg‐AD males showed lower CRH mRNA in the paraventricular nucleus of the hypothalamus compared to wild‐type males, with no difference detected between females. Female 3xTg‐AD mice exhibited significantly higher Aβ immunoreactivity in hippocampus and the basolateral amygdala compared to male 3xTg‐AD mice.
Hoorgan et al., 2007^*^	Tg2576 mice	Male	CRH levels in Tg2576 mice were increased in the cingulate, frontal, perirhinal, and entorhinal cortices. In contrast, hippocampal CRH levels were decreased in Tg2576 mice at 18 months, and were lower in the hypothalamus at 18 and 24 months.
Lv et al., 2020^403^ ^,^ ^*^	ICR mice with intracerebroventricular injection of Aβ_1‐42_	Male	Serum corticosterone levels, glucocorticoid receptor, and in the frontal cortex and hippocampus increased in Aβ_1‐42_‐treated mice. Phosphorylation of cAMP response element binding decreased during the same period. These effects returned to normal by 8 months. Aβ_1‐42_ mice showed deficits in the Morris water task. Adrenal gland to body weight ratio also increased at 1 month, peaked at 4 months, and normalized by 8 months.
Zhang et al., 2016^371^ ^,^ ^$^	PSAPP	Male and female	CRH antagonist R121919 reduced Aβ plaques and amyloid precursor protein C‐terminal fragments in both male and female AD mice. β‐secretase activity was decreased in treated females but not in males. In females, the treatment rescued dendritic and synaptic marker loss in the cortex and hippocampus. In males, treatment prevented only a minor loss of a synaptic marker in the cortex. CRH_1_ antagonist treatment also prevented spatial memory deficits only in female AD mice, resulting in performance comparable to control females. Treatment had no effect on cognitive performance of male AD mice, which did not show memory impairment at this age.
Campbell et al., 2015^375^ ^,^ ^*^	CRH overexpressing mice	Female	Overexpression of CRH was associated with increased tau phosphorylation in the hippocampus, which was rescued by the CRH_1_ antagonist R121919. R121919 treatment significantly reduced c‐Jun N‐terminal kinase phosphorylation in CRH overexpressing mice, despite no significant changes observed with activated and inactivated GSK‐3β levels.
Carroll et al., 2011^374^ ^,^ ^*^	Tg2576, PS19, and CRH overexpressing mice	Male	Chronic stress promoted subregion‐specific hippocampal tau hyperphosphorylation, soluble and insoluble tau aggregation, and neurodegeneration. These effects were correlated with impaired learning and memory in a fear conditioning paradigm. Chronic corticosterone administration did not mimic these stress‐induced effects, but these effects were prevented with CRH_1_ antagonist pretreatment.
Rissman et al., 2007^373^ ^,^ ^*^	CHR_1_ and CHR_2_ KO mice	Male	Acute restraint stress increased hippocampal phosphorylated tau. This effect was not prevented by blocking the stress‐induced rise in glucocorticoids but was blocked by genetic or pharmacologic antagonism of CRH_1_.
Rissman et al., 2012^372^ ^,^ ^*^	CHR_1_ CHR_2_ KO mice	Male	Repeated, but not acute, restraint stress increased hippocampal phosphorylated tau in wild‐type and CRH_2_ KO mice. In contrast, CRH_1_ and CRH double KO mice, or wild‐type mice pretreated with a CRH_1_ antagonist, failed to show stress‐induced alterations in phosphorylated tau. stress produced an overlap between hippocampal CRH_1_ expression and cells positive for phosphorylated tau.
De Souza et al., 1986^359^ ^,^ ^*^	Human *post mortem*	Men and women	CRH levels decreased while CRH receptor levels increased in the temporal, frontal, and occipital cortices of AD patients. These changes were correlated with decreased choline acetyltransferase activity.
Whitehouse et al., 1987^360^ ^,^ ^*^	Human *post mortem*	Unspecified	CRH immunoreactivity was decreased in the frontal, temporal, and occipital cortices in AD, Parkinson's disease, and progressive supranuclear palsy patients. Reductions in immunoreactivity were correlated with reduced choline acetyltransferase activity.
Pomara et al., 1989^361^ ^,^ ^*^	Human	Men and women	CRH immunoreactivity in cerebrospinal fluid of AD patients was unchanged compared to controls. However, in AD patients, cognitive deficits were worse in patients with lower CRH immunoreactivity in cerebrospinal fluid.
Frederiksen et al., 1991^378^ ^,^ ^$^	Human *post mortem*	Men and women	CRH concentration positively correlated with age in controls. There were no differences in CRH concentration in patients with schizophrenia. Mean CRH concentration was higher in the hypothalamus of women.
Stroud et al., 2011^391^ ^,^ ^$^	Human	Men and women	Girls over puberty displayed increasing cortisol levels after CRH challenge whereas boys showed no change. The effect in girls was due to prolonged time to reach peak cortisol and delayed return to baseline.
Gallucci et al., 1993^394^ ^,^ ^$^	Human	Men and women	In response to ovine CRH administration, plasma adrenocorticotropin hormone response was greater and more prolonged in women.
**Oxytocin**			
Jackson et al., 2013^467^ ^,^ ^*^	B6.Cg‐Tg(APPswe, PSEN1dE9)85Dbo mice	Female	The OXT gene is downregulated in a mouse model of AD.
El‐Ganainy et al., 2022^487^ ^,^ ^*^	Aluminum chloride‐induced AD in Sprague–Dawley rats	Female	Intranasal OXT treatment improved cognition in the Morris water maze and reduced acetylcholinesterase activity, Aβ deposition, ERK1/2, GSK‐3β kinase, caspase‐3, and tau levels.
Ye et al., 2022^484^ ^,^ ^*^	APP/PS1 mice	Female	Nanogel delivery of OXT prevented cognitive decline and hippocampal atrophy, inhibited inflammatory signaling, and delayed both Aβ deposition and neuronal apoptosis.
Takahashi et al., 2022^486^ ^,^ ^*^	Intracerebroventricular injection of Aβ_25–35_ in ddY mice	Male	Intranasal OXT treatment improved working and spatial memory.
Cheng et al., 2023^483^ ^,^ ^*^	APP/PS1 mice	Female	An engineered macrophage‐biomimetic versatile nanoantidote loaded with OXT improved working and spatial memory.
Selles et al., 2023^465^ ^,^ ^*^	Aβ infusion and APP/PS1 mice	Male	Various AD models displayed reduced OXT levels. Intranasal OXT treatment attenuated microglial activation; altered amyloid plaque morphology; and reversed social, object recognition, and spatial memory deficits.
Koulousakis et al., 2023^485^ ^,^ ^*^	APP/PS1 mice	Female	Intranasal OXT treatment improved spatial and working memory, decreased sociability, and reduced diffuse/dense core plaque ratio.
Usmani et al., 2024^466^ ^,^ ^*^	5xFAD mice	Male	5xFAD mice show reduced OXT expression levels. Intranasal OXT treatment improved performance in cognitive tests and reduced Aβ deposition only when combined with gonadotropin‐releasing hormone.
Ye et al., 2024^488^ ^,^ ^*^	APP/PS1 mice	Male	Intranasal OXT treatment improved cerebral hemodynamics and glymphatic drainage, enhanced intracranial lymphatic clearance of Aβ, ameliorated cognitive impairment, and restored dysregulated meningeal transcriptional profiles.
Zhang et al., 2024^480^ ^,^ ^*^	APP/PS1 mice	Male	APP/PS1 mice showed reduced hippocampal OXT levels.
Sarahian et al., 2025^489^ ^,^ ^*^	Aβ_25–35_ hippocampal injection in Wistar rats	Male	Intranasal OXT treatment improved working and spatial memory and increased the expression of synaptic plasticity‐related genes.
Li et al., 2025^468^ ^,^ ^*^	APP/PS1 mice	Male	APP/PS1 mice displayed no difference in number of OXT neurons but had reduced serum OXT levels. Intranasal OXR treatment during social isolation increased sociability while having no effect on social novelty or memory. Treatment further reduced Aβ deposition and microglia number, and attenuated anxiety‐ and depression‐like behaviors.
Rodeck et al., 1960^549^ ^,^ ^*^	Albino rats (Dusseldorf strain)	Male and female	OXT expression in the hypothalamus was slightly lower in aged rats.
Kania et al., 2020^558^ ^,^ ^$^	Sprague–Dawley rats	Male and female	AVP neurons are more numerous than OXT neurons in the paraventricular nucleus. More OXT neurons were observed in males than females. OXT cells exhibited broader action potential shapes and slower hyperpolarizing after‐potential kinetics. Male OXT neurons displayed higher membrane resistance and slower hyperpolarizing after‐potential kinetics than females.
Kelberman et al., 2024^543^ ^,^ ^*^	Prairie voles	Male and female	Number of OXT neurons in the paraventricular nucleus increase with age.
Fliers et al., 1985^429^ ^,^ ^*^	Brown‐Norway rats	Male	OXT fiber density was similar in young and old rats.
Freda et al., 2022^504^ ^,^ ^$^	C57Bl/6 Mice	Male and female	Tracing of OXT and AVP neurons revealed largely conserved inputs between the two neuronal populations. Outputs of each neuromodulator differed, and sex differences were more pronounced in the AVP system.
Fliers et al., 1985^429^ ^,^ ^†^	Human *post mortem*	Men and women	Mean profile area and size of OXT cells in the paraventricular nucleus and supraoptic nucleus did not change with aging or in patients with senile dementia of the AD type. No sex differences were observed.
Wierda et al., 1991^430^ ^,^ ^†^	Human *post mortem*	Men and women	The number of OXT neurons was unchanged in aging and AD. No sex differences were observed.
Ishunina & Swaab, 1999^431^ ^,^ ^†^	Human *post mortem*	Men and women	No differences were found in any morphometric measure of OXT neurons in the paraventricular nucleus over the course of aging. No sex differences were observed.
Raskind et al., 1986^469^ ^,^ ^*^	Human	Men	Cerebrospinal fluid levels of OXT did not differ between young, elderly, or AD participants.
Mazurek et al., 1987^470^ ^,^ ^*^	Human *post mortem*	Men and women	OXT levels in the hippocampus and temporal cortex of AD patients were significantly higher than controls.
Petekkaya et al., 2021^471^ ^,^ ^*^	Human	Men and women	Patients with mild AD displayed decreased OXT signal and increased OXT concentration that were associated with increased right parahippocampal gyrus volume. OXT signal was lower in controls compared to those with mild AD, but OXT concentration was higher in those with mild AD.
Zou et al., 2022^472^ ^,^ ^*^	Human	Men and women	Changes in OXT signaling pathway genes were common in MCI and cerebral small vessel disease patients that transition to AD.
Santiago et al., 2022^473^ ^,^ ^$^	Human *post mortem*	Men and women	OXT signaling pathway genes were identified as switch genes (key genes in the progression of AD) only in male AD patients.
**Vasopressin**			
Hernández et al., 2016^501^ ^,^ ^*^	Wistar rats	Male	AVP neurons from the hypothalamic neurosecretory pathway connect to GABAergic neurons in the central amygdala. AVP infusion to the amygdala mimicked effects of water‐deprivation stress (increase anxiety‐like behavior). Maternal separation increased the density of AVP innervation to the amygdala.
Mieda et al., 2015^515^ ^,^ ^*^	AVP‐Bmal1 KO mice	Male and female	*Bmal1* deletion in AVP‐producing neurons increased activity time, reduced suprachiasmatic nucleus genes associated with intracellular communication, and disrupted normal oscillatory patterns of cells.
Woodson & Bergan, 2023^503^ ^,^ ^†^	AVP‐ires‐CRE and R26‐LSL‐tdTomato mice	Male and female	Major inputs to AVP‐expressing neurons in the paraventricular nucleus originate from the hypothalamus and thalamus and come from mostly non‐peptidergic cells. Inputs were similar in males and females.
Rodeck et al., 1960^549^ ^,^ ^*^	Albino rats (Dusseldorf strain)	Male and female	AVP expression in the hypothalamus did not change with age.
Kania et al., 2020^558^ ^,^ ^$^	Sprague–Dawley rats	Male and female	AVP neurons are more numerous than OXT neurons in the paraventricular nucleus. More AVP neurons were observed in males than females. AVP cells exhibited narrower action potential shapes and faster hyperpolarizing after‐potential kinetics. Male and female AVP electrophysiological properties were largely similar.
Kelberman et al., 2024^543^ ^,^ ^*^	Prairie voles	Male and female	Number of AVP neurons in the paraventricular nucleus did not change with age.
Wang et al., 1994^510^ ^,^ ^*^	Prairie voles	Male	Injection of AVP to the septum increased paternal behavior in male prairie voles, while antagonism of the AVP 1a receptor reduced paternal behavior.
Dobie et al., 1991^511^ ^,^ ^*^	F344 rats	Male	Fewer AVP labelled cells were observed in the bed nucleus of the stria terminalis in aged animals. The cells of aged animals were also less intensely labelled. Testosterone, which helps synthesize AVP in this brain region, was also lower in old animals.
Rigney et al., 2023^512^ ^,^ ^$^	AVP‐iCre mice	Male and female	Inputs to the AVP neurons in the bed nucleus of the stria terminalis and amygdala mainly originated from the hypothalamus and olfactory bulbs, respectively. Both AVP neuronal populations project mainly to subcortical structures. Output density generally skewed male, while input density was mixed based on location.
Fliers et al., 1985^429^ ^,^ ^*^	Brown‐Norway rats	Male	AVP fiber density across multiple brain regions decreased with age, but was spared in the paraventricular thalamus and solitary tract.
Van Zwieten et al., 1993^514^ ^,^ ^*^	Brown‐Norway rats	Male	AVP immunoreactive cell bodies decreased in the medial amygdala and LC in aged animals. Testosterone levels were reduced beginning at middle age, which correlated with the number of AVP‐immunoreactive cell bodies in both brain regions.
Rood et al., 2013^529^ ^,^ ^$^	C57Bl/6 Mice	Male and female	Gonadectomy and electrolytic lesions were used to eliminate AVP expression in the bed nucleus of the stria terminalis and amygdala, and the suprachiasmatic nucleus, respectively. AVP neurons emanating from the bed nucleus of the stria terminalis and amygdala innervate modulatory nuclei whereas those originating in the suprachiasmatic nucleus project to regions important for hormone regulation. Generally, but not always, males had higher AVP fiber density than females.
Ishunina & Swaab, 1999^431^ ^,^ ^$^	Human *post mortem*	Men and women	AVP cell size was larger in elderly than young women and correlated with age in the paraventricular nucleus. AVP cell size was larger in young men than young women. Sex differences were more pronounced in the left hemisphere paraventricular nucleus.
Raskind et al., 1986^469^ ^,^ ^*^	Human	Men	Cerebrospinal fluid levels of AVP was lower in AD patients compared to young or old participants. Plasma AVP levels did not differ between the three groups but the AD group lacked high values present in young and old participants.
Petekkaya et al., 2021^471^ ^,^ ^*^	Human	Men and women	Patients with mild AD displayed increased AVP concentration associated with increased right parahippocampal gyrus volume. There were no significant differences in AVP signal or concentration between those with mild AD and controls.
Son et al., 2024^59^ ^,^ ^*^	Human *post mortem*	Men and women	AVP neurons in the suprachiasmatic nucleus were selectively vulnerable to developing neurofibrillary tangles and tau fibrils. The paraventricular nucleus and supraoptic nucleus only showed mild tau pathology in late Braak stages. The suprachiasmatic nucleus also showed early immune dysregulation, but was largely spared from amyloid pathology. Number of AVP neurons in the suprachiasmatic nucleus decreased with increasing disease stage.
Goudsmit et al., 1990^547^ ^,^ ^†^	Human *post mortem*	Men and women	There was no loss in total cell number or volume in the supraoptic nucleus or paraventricular nucleus during aging or in AD. There was a decrease in both volume and total cell count in the supraoptic nucleus during both aging and AD. No sex differences were found.
Fliers et al., 1985^429^ ^,^ ^†^	Human *post mortem*	Men and women	Mean profile area of AVP cells in the paraventricular nucleus and supraoptic nucleus decreased up to the sixth decade of life and then gradually increased. Size of AVP cells did not change with aging. There were no deviations from aging found in patients with senile dementia of the AD type and no sex differences were observed.
Lucassen et al., 1997^548^ ^,^ ^*^	Human *post mortem*	Men and women	AVP mRNA in the paraventricular nucleus and supraoptic nucleus were unchanged during aging and in AD. There were negative correlations between the volume of AVP mRNA and age in the supraoptic nucleus of AD patients. There was a positive correlation between paraventricular nucleus AVP mRNA and cerebrospinal fluid pH.
**Histamine**			
Zou et al., 2025^613^ ^,^ ^*^	5xFAD mice	Male and female	The H3R antagonist/inverse agonist pitolisant improved memory and cognitive flexibility, restored cortical slow‐wave coherence and normalized frequency, reduced soluble and insoluble Aβ levels and plaque burden, decreased dystrophic neurite area and density, and increased cathepsin B/D levels and enzyme activity. Beneficial effects were blocked by cathepsin inhibitor treatment. Beneficial effects were similar between sexes but not statistically assessed.
Zhang et al., 2023^618^ ^,^ ^*^	3xTg‐AD mice	Female	Depression‐like behavior and L‐histidine levels were increased in hippocampus of aged female 3xTg‐AD mice. There were no changes in HA levels.
Wang et al., 2022^612^ ^,^ ^*^	APP/PS1 mice	Male	The H3R antagonist/inverse agonist thioperamide reduced neuroinflammation and cognitive deficits via gliosis inhibition and astrocyte phenotype switching through CREB signaling.
Wang et al., 2019^614^ ^,^ ^*^	APP/PS1 mice	Male and female	The H3R antagonist LC1405 improved learning and memory in APP/PS1 mice and upregulated both acetylcholine and HA.
Mani et al., 2017^611^ ^,^ ^*^	B6.129‐Tg(APPSw)40Btla/J mice	Male	The H3R antagonist ciproxifan improved learning and memory, increased acetylcholine levels and decreased acetylcholinesterase activity, reduced oxidative stress and neuroinflammation markers in the absence of reductions in brain Aβ levels.
Delay‐Goyet et al., 2016^610^ ^,^ ^*^	THY‐Tau22 mice	Male	The H3R antagonist/inverse agonist SAR110894 reduced tau hyperphosphorylation, neurofibrillary tangles, and prevented cognitive deficits after 6 months of treatment.
Sundvik et al., 2013^616^ ^,^ ^*^	Presenilin1 KO zebrafish	Male and female	Presenilin knockout embryos displayed decreased HA neurons at 7‐days postfertilization compared to wild‐type. This phenotype normalized by 2 months.
Bardgett et al., 2011^608^ ^,^ ^*^	Tg2576 mice	Male and female	The H3R antagonist ciproxifan improved cognitive deficits and reduced hyperactivity in Tg2576 mice.
Bitner et al., 2011^609^ ^,^ ^*^	Tg2576 & TAPP mice	Male and female	The histamine H3R inverse agonist ABT‐239 normalized the reduced CREB and GSK‐3β phosphorylation Tg2576 mice. The H3R inverse agonist ABT‐239 reversed tau hyperphosphorylation in spinal cord and hippocampus of TAPP mice.
Van Meer et al., 2007^615^ ^,^ ^*^	ApoE KO mice	Male	The H3R antagonist/inverse agonist thioperamide was associated with reduced histamine release in the amygdala of ApoE knockout mice.
Mazurkiewicz‐Kwilecki & Prell, 1986^619^ ^,^ ^*^	Sprague–Dawley rats	Male	12‐month‐old rats displayed higher hypothalamic, midbrain and cortical HA concentrations than 3‐month‐old rats.
Mazurkiewicz‐Kwilecki & Prell, 1984^620^ ^,^ ^*^	Sprague–Dawley rats	Male	12‐month‐old rats displayed higher hypothalamic HA after stress, midbrain and cortical HA concentrations than 3‐month‐old rats.
Medhurst et al., 2009^617^ ^,^ ^*^	TASTPM mice; Human *post mortem*	Unspecified; men and women	There were no differences in H3R expression in TASTPM mice. AD patients displayed normal levels of H3R expression throughout the brain.
Oh et al., 2022^10^ ^,^ ^*^	Human *post mortem*	Men and women	Results showed increased tau burden in TMN HA neurons and demonstrated correlation with total sleep time.
Oh et al., 2019^212^ ^,^ ^*^	Human *post mortem*	Men and women	Unbiased stereological analysis demonstrated 62% decline in histaminergic neurons in the late stage of AD, which was associated with AD‐specific tau toxicity.
Shan et al., 2012^580^ ^,^ ^$^	Human *post mortem*	Men and women	There was significant global and regional HA neuronal loss in the TMN in AD patients. In contrast, L‐histidine decarboxylase mRNA expression levels in TMN did not show any changes. However, medial TMN showed a significant decline in AD. H3R mRNA expression in PFC increased only in females in Braak V–VI over Braak 0–II, while histamine methyltransferase mRNA expression was upregulated in Braak III–IV, and V–VI over Braak 0–II.
Motawaj et al., 2010^585^ ^,^ ^$^	Human	Men and women	Cerebrospinal fluid tele‐methylhistamine levels declined significantly in AD patients. In contrast, cerebrospinal fluid tele‐methylhistamine levels showed an age‐associated increase during normal aging. There was a significant decline in cerebrospinal fluid tele‐methylhistamine levels in male and female AD patients. The extent of decline was greater in females.
Panula et al., 1997^584^ ^,^ ^*^	Human *post mortem*	Men and women	HA concentration significantly declined in hypothalamus, hippocampus, and temporal lobes of AD patients.
Prell et al., 1990^607^ ^,^ ^$^	Human	Men and women	A increase in HA metabolites cerebrospinal fluid levels were seen in normal aging individuals, with females showing higher levels than males.
Cacabelos et al., 1989^583^ ^,^ ^*^	Human *post mortem*	Unspecified	HA levels in patients with senile dementia of the AD type were higher across various brain regions.
Mazurkiewicz‐Kwilecki & Nsonwah, 1989^582^ ^,^ ^*^	Human *post mortem*	Men and women	There was a decline in HA and histidine levels across cortical areas and in the caudate nucleus of AD patients.
**Orexin**			
Keenan et al., 2024^698^ ^,^ ^$^	rTg4510 mice	Male and female	Chronic OX‐B receptor antagonism improved sleep/wake patterns and cognitive function in a manner dependent on tau pathology and sex.
Keenan et al., 2022^697^ ^,^ ^$^	rTg4510 mice	Male and female	GABAergic and OX‐targeting hypnotics enhance sleep during the active phase in tau‐transgenic rTg4510 mice. Sex‐dependent differences are observed in rTg4510 mice in response to OX‐B receptor‐selective antagonists.
Taheri et al., 1999^652^ ^,^ ^$^	Wistar rats	Male and female	Female rats showed higher levels of OX‐A and prepro‐OX mRNA in the lateral and posterior hypothalamus.
Jöhren et al., 2002^653^ ^,^ ^$^	Wistar rats	Male and female	Female rats showed higher levels of OX‐A and prepro‐OX mRNA in the lateral and posterior hypothalamus
Jöhren et al., 2001^654^ ^,^ ^$^	Wistar rats	Male and female	There was sex‐dependent expression of OX‐A and OX‐B receptors in the hypothalamus, pituitary, and adrenal glands.
Silveyra et al., 2007^656^ ^,^ ^$^	Sprague–Dawley rats	Male and female	There were cyclical changes in prepro‐OX and receptor expression in adult females, particularly during proestrus.
Lu et al., 2017^670^ ^,^ ^$^	Human *post mortem*	Men and women	OX‐A immunoreactivity increased only in women with major depressive disorder. Loss of normal diurnal OX variation in patient tissue supported women‐specific OX involvement in major depressive disorder‐related sleep and mood disruption.
Fronczek et al., 2012^683^ ^,^ ^$^	Human *post mortem*	Men and women	Marked loss of hypothalamic OX neurons and reduced cerebrospinal fluid OX‐A levels in AD compared to controls. Cerebrospinal fluid levels of OX‐A were higher in female compared to male controls, which was not present in the AD group.
Schmidt et al., 2013^171^ ^,^ ^$^	Human	Men and women	Cerebrospinal fluid OX‐A levels were higher in women than men across diagnostic groups. This was interpreted as sex‐dependent physiological regulation rather than a result of disease.
Wennström et al., 2012^685^ ^,^ ^$^	Human	Men and women	Women AD patients showed highest cerebrospinal fluid OX‐A levels. Women Lewy body dementia patients had the lowest cerebrospinal fluid OX‐A levels. Women controls had intermediate cerebrospinal fluid OX‐A levels. There were no significant differences among males.
Dauvilliers et al., 2014^686^ ^,^ ^†^	Human	Men and women	Cerebrospinal fluid OX‐A levels were high in AD dementia and MCI groups compared to controls, but no sex differences were observed within or across diagnostic categories.
Liguori et al., 2014^687^ ^,^ ^†^	Human	Men and women	Higher cerebrospinal fluid OX‐A levels were observed in moderate‐to‐severe AD compared to controls with no differences between mild AD and controls. There were no sex differences detected in any group.
Liguori et al., 2016^688^ ^,^ ^*^	Human	Men and women	There was higher cerebrospinal fluid OX‐A levels in MCI patients compared to controls.
Lozano‐Tovar et al., 2025^689^ ^,^ ^$^	Human	Men and women	Cerebrospinal fluid OX‐A levels were higher in moderate AD, non‐fluent primary aphasia, and idiopathic normal pressure hydrocephalus than controls. Within clinical groups there were no sex differences, but men in the control group displayed higher OX‐A levels than women.

*Notes*: Sex differences in the OXT and AVP systems have been extensively reviewed elsewhere.^417^, ^418^, ^419^, ^420^ Therefore, we highlight newer results and those which are pertinent to AD in the above table. Citations with similar authors from the same year are differentiated with superscripts associated with their reference number.

Abbreviations: 5‐HT, serotonin; 5‐HIAA, 5‐Hydroxyindoleacetic acid; Aβ, amyloid beta; AD, Alzheimer's disease; AVP, arginine vasopressin; BACE‐1, beta secretase 1; BDNF, brain‐derived neurotrophic factor; BFCS, basal forebrain cholinergic system; cAMP, cyclic adenosine monophosphate; CRH, corticotropin releasing hormone; DA, dopamine; DBH, dopamine beta‐hydroxylase; DRN, dorsal raphe nucleus; ER, estrogen receptor; ERK, extracellular regulated kinase; GFAP, glial fibrillary acidic protein; GSK‐3β, glycogen synthase kinase 3 beta; HA, histamine; htau, human tau; KO, knock‐out; LC, locus coeruleus; MCI, mild cognitive impairment; MRI, magnetic resonance imaging; NBM, nucleus basalis of Meynert; NE, norepinephrine; NMDA, *N*‐methyl‐d‐aspartic acid; NSSs, neuromodulatory subcortical systems; OX, orexin, OXT, oxytocin; PET, positron emission tomography; PFC, prefrontal cortex; p‐tau, phosphorylated tau; REM, rapid eye movement; SERT, serotonin transporter; SNpc, substantia nigra pars compacta; snRNA‐seq, single nuclei RNA sequencing; SSRI, selective serotonin reuptake inhibitor; TMN, tuberomammillary nucleus; trkA, tropomyosin receptor kinase A; VTA, ventral tegmental area.

* Indicates sex differences were not tested.

^$^ Indicates the presence of sex differences.

^†^ Indicates sex differences were tested for, but no differences were found.

## 2 ACETYLCHOLINE

Acetylcholine is a neurotransmitter involved in higher order cortical processes including memory and attention.^23^, ^24^ The cholinergic system is located in two main subcortical nuclei, the basal forebrain cholinergic system (BFCS), which includes the nucleus basalis of Meynert (NBM), and the brainstem cholinergic nuclei composed of the pedunculo‐pontine and lateral dorsal tegmental nuclei. Both systems possess long‐range projections that innervate the majority of the cortical and subcortical regions.^25^ Multiple studies reported volume loss of the BFCS with AD.^26^, ^27^, ^28^, ^29^, ^30^, ^31^ BFCS loss is particularly associated with accumulation of tau pathology, which has been hypothesized to be the initiating factor in the decline of cholinergic neurons.^8^, ^9^ Cell loss occurs relatively early in disease progression, with changes in BFCS volume predictive of future deterioration of other subcortical structures, particularly the entorhinal and perirhinal cortices.^28^ Using the radiotracer [^18^F]‐FEOBV, studies have shown that cholinergic terminals throughout the cortex also decline with advancing disease stage.^32^ Deterioration of cognitive abilities follows this structural loss, with deficits in ability to exert top‐down control on attentional processes.^33^, ^34^, ^35^ While there is less evidence for brainstem cholinergic decline than for the BCFS, *post mortem* studies of AD patients and [^18^F]‐FEOBV imaging of patients with Lewy body dementia have both shown degeneration of the brainstem cholinergic nuclei with increasing pathological burden.^36^, ^37^

### 2.1 Baseline sex differences in cholinergic system structure and function

The cholinergic system is especially relevant to the topic of sex differences in AD, as acetylcholine is modulated by estrogenic signaling, particularly via estradiol in the cortex.^38^ Estradiol modulates BFCS function mainly through the estrogen receptor ERα and is responsible for increasing levels of choline acetyltransferase,^39^ an enzyme crucial for the synthesis of acetylcholine. Compared to the gradual decrease of sex hormones in men, estradiol levels in women are greatly diminished after menopause, and it is believed that this loss of estradiol directly affects cholinergic integrity, which may increase the risk of developing AD for some women.^38^, ^40^

The BFCS also shows distinctive sex differences with AD progression both in structure and function particularly early in the disease process. Structurally, BFCS atrophy differs across the sexes, both in terms of loss of neuronal density as well as in the reduction of receptor activity. Animal studies tend to focus on changes in cholinergic structure and function after ovariectomy, as it has been shown that cholinergic volumes do not differ between male and intact female animals.^41^, ^42^ In female rats, ovariectomy results in a reduction of cholinergic neurons of the NBM compared to intact female rats or those treated with unopposed estradiol.^43^ Reduction of cholinergic neurons in the BFCS also leads to a reduction of cortical projections, with ovariectomized female rats displaying reductions in cholinergic innervation of the entorhinal cortex.^44^ In humans, BFCS volume changes occur earlier in women compared to men.^45^ Specifically, NBM volume is reduced in women compared to men that are cognitively unimpaired or have mild cognitive impairment (MCI). Notably, there was a greater reduction in NBM volume in healthy older women > 36 years of age relative to men. Furthermore, a *post mortem* study of AD patients showed that androgen receptors are lower in women compared to men, both in the vertical limb of the diagonal band of Broca, and the NBM.^46^ Comparatively, there is less evidence for sex differences in the decline of the brainstem cholinergic system, beyond functional connectivity between brainstem and precuneus being preserved in men but not women with MCI.^47^ However, given the nature of the early involvement of cholinergic dysfunction in the development of AD, and the impact of the hormonal shift at menopause on the cholinergic system, studies have been focused on the ability of exogenous hormones to address this imbalance.

### 2.2 Hormone replacement therapy as a critical modifier of cholinergic function

Hormone therapy through the use of exogenous estrogens and progestins has been proposed as a potential neuroprotective strategy, largely due to its influence on cholinergic tone.^38^, ^48^ Exogenous estradiol modulates cholinergic activity in both animal models^48^, ^49^, ^50^, ^51^ and humans.^43^, ^52^, ^53^, ^54^ Epidemiological studies also link hormone therapy with a reduced risk of developing AD,^55^, ^56^ even in women with early life ovarian removal.^57^ However, findings from studies assessing the impact of hormone therapy on cognitive performance have been mixed.^58^, ^59^ Several factors can affect the ability of hormone therapy to boost cholinergic tone. These factors include the timing of the initiation of hormone therapy relative to the menopause transition, the duration of use, route of administration, and the specific combination of hormonal compounds.[Fig alz71291-fig-0001]

When evaluating the beneficial effects of exogenous estrogens in cognitively unimpaired postmenopausal women, studies often use a stressor or pharmacological challenge to identify underlying cholinergic dysfunction. Cholinergic antagonists, such as mecamylamine (nicotinic receptors) or scopolamine (muscarinic receptors) are often used for this purpose.^60^, ^61^, ^62^, ^63^ A single dose of estradiol attenuates scopolamine‐induced memory impairments in ovariectomized rats.^64^ Similarly, postmenopausal women who receive oral estradiol for 3 months perform better on working memory and attention tasks under an anticholinergic challenge compared to those on placebo.^65^, ^66^ Interestingly, these beneficial effects appear to be age dependent. Estradiol mitigates the anticholinergic challenge only in younger postmenopausal women, while older women showed worsened performance while on estradiol.^67^ This pattern aligns with preclinical data demonstrating that cognitive benefits of exogenous estradiol treatment are greatest when treatment occurs closer to ovariectomy.^68^, ^69^ These studies highlight the importance of the timing of hormone therapy initiation relative to menopause, a fundamental concept of the critical window hypothesis.^67^, ^70^, ^71^ Most studies report improved cognitive outcomes when hormone therapy begins within the first few years of menopause, whereas initiation ≥ 5 years later reduces beneficial effects.^70^, ^72^, ^73^, ^74^ The hypothesis suggests that estradiol's beneficial effects on the cholinergic system are most pronounced while the nuclei are still structurally intact.

The importance of identifying optimal treatment windows is also exemplified by studies examining individuals with subjective cognitive complaints,^75^, ^76^ a transition stage associated with greater risk of progressing to MCI or AD. In these individuals, estradiol appears less effective in counteracting cholinergic disruption. In a study of cortical activation of working memory, postmenopausal women with greater self‐reported cognitive complaints exhibited greater overall cortical activation compared to participants who reported fewer complaints. Such activation suggests increased neuronal effort is required to successfully complete tasks.^77^ Under an anticholinergic challenge, these postmenopausal women performed worse, regardless of whether they received 3 months of estradiol or placebo.^78^

Finally, composition of the hormone therapy regimen also determines its effects on cognition and cholinergic system integrity. Hormone therapy typically combines estrogen and progesterone replacement, with the former compensating for reduced circulating estradiol and the latter preventing endometrial hypoplasia.^79^, ^80^ Both estrogen and progestin types influence cognitive and cholinergic performance. For example, conjugated equine estrogens increased incidence of dementia and MCI in women in the Women's Health Initiative.^81^, ^82^ This effect was most pronounced in older women who were long past menopause,^83^ consistent with the critical window hypothesis. Progestins themselves can have differential effects on cholinergic function and cognitive performance, both alone and in combination with estrogens.^84^ Beneficial effects of progesterone have been seen in ovariectomized mice, in which progesterone in conjunction with estradiol improved choline acetyltransferase activity compared to estradiol or progesterone alone.^85^ A beneficial effect of combined hormone therapy was also seen in postmenopausal women who initiated treatment earlier after menopause. Compared to estrogen therapy alone, women taking estrogens and progestins had greater cholinergic uptake of the radiotracer N‐[^11^C]methylpiperidin‐4‐yl propionate in the hippocampus and posterior cingulate.^53^ However, like estrogens, the type of progestins seem to influence their ability to modulate cholinergic tone. For example, medroxyprogesterone acetate, the most commonly used progestin, impairs cognitive performance in both in animal models and human studies.^86^, ^87^ In contrast, micronized progesterone tends to be less harmful and may even improve some cognitive measures.^86^ However, in the presence of an anticholinergic agent, micronized progesterone interferes with estradiol's ability to improve cognitive performance.^88^ Overall, while preclinical research generally supports the neuroprotective role of hormone therapy through enhanced cholinergic function, evidence from studies in postmenopausal women are mixed. This variability likely reflects differences in age at initiation, treatment duration, hormone formulation, and underlying cholinergic system integrity.

### 2.3 Future directions

Cholinergic system decline is synonymous with AD progression and has been proposed as a factor in the sex disparity of AD, due to its relationship with estrogens and the consequences of the menopause transition.^38^, ^39^ Both animal and human studies show that loss of estradiol increases the vulnerability to cholinergic decline, driven in part by increased tau propagation. Due to the relationship between the cholinergic system and estrogens, a particular focus of past research has been on whether the use of exogenous hormones can mitigate the loss of estradiol after menopause. The benefits of hormone therapy on cholinergic function and cognitive performance are inconclusive, owing to a complex interaction of factors, including the timing of replacement therapy, that impact the ability of hormone therapy to improve performance. Moreover, not all women develop AD, and a better understanding of the underlying vulnerability of some women for cholinergic dysfunction and AD progression should be a focus of future research. Measurements of cholinergic integrity, like cholinergic radiotracers, in conjunction with new fluid biomarkers, may offer a more accurate way of probing the relationships among the cholinergic system, hormone therapy, and AD pathological burden.

## 3 DOPAMINE

Dopaminergic circuits originate from the substantia nigra pars compacta (SNpc) and the ventral tegmental area (VTA).^89^, ^90^ Dopaminergic neurons in the SNpc project to the dorsal striatum, forming the nigrostriatal pathway, which is critical for movement and action selection.^91^ In contrast, dopaminergic neurons in the VTA project to the ventral striatum and nucleus accumbens, forming the mesolimbic pathway, and to the prefrontal cortex (PFC), forming the mesocortical pathway. The former is implicated in reward processing and motivated behavior, while the latter is important for cognition.^92^, ^93^ Additionally, dopaminergic neurons in both the SNpc and VTA project to the hippocampus, where DA release supports synaptic plasticity and memory formation.^94^

### 3.1 Baseline sex differences in dopaminergic circuits and regulation by sex hormones

Sex differences in dopaminergic circuits are evident across the lifespan^95^ and impact trajectories for many DA‐related disorders, such as Parkinson's disease, schizophrenia, and substance use disorder.^96^, ^97^, ^98^ For example, human neuroimaging studies reported higher D2/3 receptor density, transporter density, striatal DA release, and DA synthesis in women than men.^99^, ^100^, ^101^, ^102^, ^103^ However, other studies, although fewer, reported either opposite results, such as higher DA release in men than women,^104^ or null effects (e.g., D2/3 receptor density^102^). Findings from preclinical studies also generally confirm the existence of sex differences in dopaminergic circuits. For instance, there are robust sex differences in the degree of connectivity between the VTA/SNpc and its targets. In female rats, 50% of the projections from the VTA to the PFC are dopaminergic, while in male rats this proportion is only 30%.^105^ Similarly, DA release in the dorsal striatum is greater in females relative to males, suggesting that, like the PFC, the dorsal striatum of females receives denser dopaminergic input than that of males.^106^

Not only does the dopaminergic system vary by sex, but it is also regulated by gonadal hormones. Women with greater baseline DA in the PFC display a negative association between PFC‐dependent cognitive function and levels of estradiol.^107^ An investigation of the neural and cognitive effects of contraceptive use found that hormonal contraceptive users had greater DA synthesis capacity in the dorsal striatum and better cognitive flexibility relative to non‐users.^108^ Importantly, across groups, higher DA synthesis capacity was associated with greater cognitive flexibility. Considered together, these clinical studies provide clear evidence that sex hormones potently influence DA function that is relevant for optimal cognitive function, particularly in women.

Similarly, in preclinical studies, striatal DA release is positively correlated with estradiol levels in intact female rats and the administration of estradiol and progesterone to ovariectomized female rats increases striatal DA release.^109^ In contrast, androgens have no effect on striatal DA properties, but instead appear to regulate cortical DA function in males.^109^, ^110^, ^111^ Androgens tonically suppress DA release in the PFC,^111^ and testosterone inhibits burst firing in PFC‐projecting VTA neurons.^112^, ^113^, ^114^ Consequently, androgen regulation contributes to lower basal PFC DA levels in males, increasing their susceptibility to cortical hypodopaminergia.^110^, ^112^

### 3.2 The dopaminergic system in AD and the moderating influence of sex

Even without considering sex as a modifying factor, there is evidence of DA dysfunction in AD.^115^, ^116^, ^117^ Studies using genetic polymorphisms to infer individual differences in DA function have demonstrated links between the rs6347 DA transporter polymorphism, Aβ, and tau pathology,^118^ as well as an increased risk for developing AD and greater cortical shrinkage with expansion of the ventricles.^119^ In addition, Aβ plaque burden in the striatum is predictive of AD severity,^120^ and dopaminergic midbrain nuclei accumulate tau pathology before it spreads to cortical regions,^121^ suggesting these midbrain areas may be vulnerable in early stages of AD. Similarly, in the Tg576 mouse model of AD, selective neuronal loss in the VTA (but not in the SNpc) was observed prior to Aβ plaque formation.^122^ This dysregulation of DA circuitry not only affects midbrain DA nuclei but also impacts the targets of their projections. In AD patients, hippocampal D2 receptor density is lower than in healthy controls.^115^ Given that higher D2 receptor density is correlated with better memory performance, the reduction in hippocampal D2 receptors in AD patients could account for memory impairments characteristic of AD. Consistent with these clinical findings, VTA neuron loss in Tg576 mice is correlated with lower hippocampal DA levels, lower CA1 synaptic plasticity, worse memory performance, and impaired reward learning.^122^ Based on these findings, degeneration of the VTA in AD may lead to deficits in DA‐dependent hippocampal function and contribute to memory impairments typically associated with AD.

Although it is unclear how DA dysfunction might be mechanistically related to AD, it is possible that AD pathology and aging‐related dysregulated DA may exacerbate each other, leading to worse clinical outcomes. For example, oxidative stress and Aβ aggregation in AD are linked to mitochondrial DNA damage, mitochondrial dysfunction, inflammation, and cytotoxicity.^123^ The administration of DA and its derivatives, however, can combat the effects of oxidative stress and Aβ aggregation.^124^ Hence, AD‐associated hypodopaminergia may weaken the usual protective effects exerted by DA and, as a consequence, make neurons more vulnerable to oxidative damage. Interestingly, a DA receptor agonist improves cognition in individuals with MCI,^116^ thus providing additional support for the supposition that increased DA levels may protect against cognitive decline.

Few studies have considered sex as a moderating factor in the bidirectional interplay between AD and the DA system, despite well‐established sex differences in AD presentation.^125^ For example, women with AD often present with greater severity of depressive symptoms, aberrant motor behavior, and psychotic symptoms, all of which are associated with DA dysfunction. Such sex‐dependent presentation of AD suggests sex‐specific AD‐related changes to this system. This notion is supported by work using the 5xFAD mouse model, in which females exhibited greater striatal Aβ plaque burden, hyperlocomotion, and reduced stereotypies compared to males.^126^ Additionally, female, but not male, 5xFAD mice exhibited reduced DA transporter and tyrosine hydroxylase expression.^126^ Beyond this recent work, however, very little is known about the DA‐linked mechanisms underlying the greater susceptibility and sensitivity of females to AD pathology, underscoring the need for more research in this area.

Although the mechanisms that contribute to sex differences are not well understood, gonadal hormones appear to be involved. Estrogen receptor polymorphisms are associated with increased AD risk in women.^127^ DNA methylation and RNA expression of estrogen receptor genes, particularly *GPER1*, in the PFC is associated with greater cognitive decline and higher *post mortem* indices of AD pathology, with more pronounced effects in women.^128^ Although there is no direct link between hormonal modulation of DA and AD development, it is conceivable that hormone‐related changes in the DA system during menopause, a period when estradiol levels decline, may place women at a higher risk for developing AD. Estradiol protects against Aβ and tau pathology, oxidative stress, and mitochondrial damage while loss of estradiol after menopause reduces antioxidant capacity.^129^ These findings are consistent with evidence from a rodent Aβ_1‐42_ infusion model of AD. While ovariectomy induced hippocampal oxidative stress in aged female rats, estradiol administration ameliorated detrimental effects.^130^ Greater oxidative stress likely impacts cellular function throughout the brain, but dopaminergic neurons may be particularly affected as they have high energy requirements.^131^, ^132^ Hence, the typical age‐related reduction in circulating hormones may increase female risk for AD due to changes in the metabolic function and capacity of DA neurons.

Intriguingly, although men have a lower AD risk than women overall, men, especially of healthy older populations, can present with quicker and more severe cognitive decline.^133^ This sex difference may be attributed to baseline differences in dopaminergic projections from the VTA to the PFC and the influence of androgens on DA release in the PFC from neurons originating in the VTA.^113^, ^114^ Hence, both gonadal hormones and other factors associated with biological sex could be considered risk factors predisposing males to developing cortical hypodopaminergia and AD‐associated cognitive deficits. However, more work is needed to test this hypothesis and examine how AD‐related changes in the DA system may account for sex differences in metrics of disease severity, especially given evidence that women with AD show more pronounced cognitive decline than men.^14^, ^15^, ^16^, ^17^, ^18^, ^19^, ^20^, ^21^, ^22^, ^133^

### 3.3 Future directions

The evidence for sex differences in the dopaminergic system support a link between its sex‐specific dysfunction and AD. Like other NSSs, hormonal regulation of the dopaminergic system may be a critical mediator of sex differences in AD. Relative to other neuromodulators, however, far less is known about how sex‐dependent alterations in the dopaminergic system contribute to the pathophysiology of AD. One limitation of existing clinical studies is that they lack the statistical power necessary to effectively analyze sex differences. Hence, an important future direction in this field is to leverage large, publicly available datasets (e.g., the Alzheimer's Disease Neuroimaging Initiative) to study how both dopaminergic and hormone‐related genetic polymorphisms relate to neural measures of AD pathology and cognitive trajectories. Another critical avenue of research is to identify how interactions between DA and other neurotransmitters, such as 5‐HT and NE, impact AD pathology. Although preclinical studies have demonstrated an association between DA and 5‐HT in AD,^134^ no clinical studies exist that explore such a relationship. Similarly, the locus coeruleus (LC), although known for being the brain's primary source of NE,^135^ also synthesizes DA^94^, ^136^ and is especially vulnerable to tau pathology.^11^ For these reasons, this nucleus is of primary interest for future research on how interactions between DA and NE may influence sex‐dependent vulnerability to AD.

## 4 NOREPINEPHRINE

Extensive evidence implicates the LC, the brain's primary, but not exclusive, source of NE, as a central node in the early stages of AD pathogenesis.^5^, ^137^, ^138^, ^139^ The LC–NE system is a key component of the brain's arousal and stress network, supporting alertness, attention, and cognition.^140^, ^141^, ^142^, ^143^, ^144^, ^145^, ^146^, ^147^ Notably, hyperphosphorylated tau in its pretangle form accumulates in LC neurons decades before clinical symptoms, suggesting a brainstem origin of AD pathology.^5^, ^148^ This observation has spurred increasing interest in the LC's role in shaping AD vulnerability.^149^, ^150^ Given that AD is more prevalent in women than men, LC degeneration, an early AD hallmark, has been hypothesized to be more pronounced in women, partially due to estradiol decline during and after perimenopause. Such hormonal changes may exacerbate NE depletion, thereby accelerating tau propagation and cognitive decline.^149^

### 4.1 Intrinsic vulnerability of the LC–NE system in female rodents

Rodent models have shown that the LC exhibits marked sex differences, with females displaying larger LC volume, greater neuronal counts, and more elaborate dendritic arborization compared to males.^151^, ^152^, ^153^, ^154^, ^155^ While these features may enhance arousal regulation and neuromodulatory capacity, they also increase metabolic demand and sensitivity to oxidative stress, potentially rendering the female LC more vulnerable to degeneration during aging or chronic stress exposure. Recent transcriptomic and proteomic analyses have also shed light on sex‐dependent LC vulnerability. For instance, the female LC in mice expresses elevated levels of stress‐responsive genes such as *Ptger3*, which modulates LC excitability and anxiety‐like behaviors.^156^ Estradiol also enhances NE biosynthesis by upregulating tyrosine hydroxylase, the rate‐limiting enzyme in NE production.^157^, ^158^ On the other hand, proteomic signatures suggest reduced regulation of protein turnover and stress pathways, including eukaryotic initiation factor 2 signaling and glucocorticoid response, in the female rodent LC.^159^ These molecular differences may compromise resilience to neurodegenerative processes, contributing to the greater AD susceptibility observed in women.

Increasingly, animal models of amyloidosis have been used to examine LC degeneration, but few incorporate sex‐stratified analyses. In studies of female APP mice, N‐(2‐chloroethyl)‐N‐ethyl‐2‐bromobenzylamine‐induced LC lesions resulted in increased plaque burden, glial activation, and cognitive deficits.^160^ Aged female APP/PS1 mice exhibited selective LC neuron loss despite preserved dopaminergic systems,^161^ aligning with human evidence of early LC degeneration in AD.^162^, ^163^, ^164^, ^165^, ^166^, ^167^ One study found that LC chemogenetic silencing or adrenoceptor blockade exacerbated central nervous system inflammation,^168^ with Adrb1 knockdown in microglia intensifying inflammatory responses exclusively in females, indicating sex‐specific differences in adrenergic signaling.

Studies using TgF344‐AD rats and APP transgenic mice with LC lesions reported increased Aβ deposition, synaptic deficits, and cognitive impairment but did not analyze sex differences.^169^, ^170^, ^171^ Other investigations in males show that in V717F‐APP, LC noradrenergic depletion increased Aβ plaque burden and reduced neprilysin, a key Aβ‐degrading enzyme, highlighting NE's role in Aβ clearance.^172^ Similarly, in male 5xFAD mice, vindeburnol confers neuroprotection by upregulating tyrosine hydroxylase, reducing Aβ plaque load, and improving behavior via cyclic adenosine monophosphate‐mediated brain‐derived neurotrophic factor upregulation.^173^ While informative for males, these studies underscore the need for comparable data in females to elucidate sex‐specific mechanisms of LC degeneration and AD progression.

Animal models of tau pathology within the LC have provided critical insight into early‐stage AD but, like most amyloid models, often lacked sex‐stratified analyses. Transgenic models carrying the human *MAPT* P301S or P301L mutation linked to frontotemporal dementia^174^, ^175^ are widely used to study tauopathy. In PS19 mice (P301S), tau fibril injection into the LC induced rapid tau aggregation and neuronal loss on the ipsilateral side compared to the contralateral LC, which exhibited tau clearance and minimal cell loss, likely reflecting lateralization of pathological burden and more effective degradation.^174^ In P301L mice, similar injections disrupted hippocampal network activity without overt tau propagation.^175^ Although both sexes were included in the previous two studies, outcomes were not analyzed separately by sex. Similarly, TgF344‐AD rats show early LC tau accumulation accompanied by axonal degeneration and cognitive decline that precedes entorhinal and hippocampal pathology.^176^ A follow‐up study identified age‐ and stage‐dependent dysregulation of LC firing,^177^ though sex differences were not assessed.

Emerging findings suggest that sex significantly modulates tau‐related LC vulnerability. In human tau (htau) overexpressing mice,^178^ males displayed greater hyperactivity and anxiety, whereas both sexes exhibited depressive‐like behaviors and LC pathology. These results indicate shared neuropathology but divergent behavioral outcomes. LC‐targeted models using pseudophosphorylated htauE14 have substantiated foundational studies that pretangle tau pathology originates in the LC and contributes to AD progression.^5^, ^179^, ^180^, ^181^, ^182^ htauE14 induces LC degeneration and tau spread, impairing olfactory learning.^180^ These effects were reversed by driving LC–NE activity in a way that mimics its natural response to novelty.^182^ A more recent report shows that LC htauE14 triggers peripheral and central nervous system inflammation and blood–brain barrier disruption.^179^ Comparing transcriptional signatures of the LC in females and males to htauE14, males showed broad downregulation of synaptic and ion channel genes, while females showed targeted alterations in metabolic and developmental pathways.^181^ Notably, only males displayed an upregulation of NE synthesis genes, suggesting that males, but not females, may mount a compensatory response. On the other hand, probiotic treatment rescued learning and reduced inflammation, with hippocampal glycogen synthase kinase 3 beta suppression observed only in females.^179^ These molecular distinctions likely underlie observed sex‐specific behavioral and neuropathological outcomes in response to tau pathology in the LC.

### 4.2 LC–NE interactions with CRH

The LC–NE system has intricate reciprocal connections with other neuromodulatory systems that, unlike other systems, have been functionally characterized. One such neuromodulator is the stress‐related neuropeptide CRH, which regulates the LC in a sex‐dependent manner. Compared to males, LC neurons in adult female rats are more sensitive to local CRH infusions (causing increased neuronal discharge rate) as indicated by a left shift in the CRH dose–response curve.^151^ Further, after local CRH administration, females show increased cyclic adenosine monophosphate‐mediated cellular signaling as well as reduced swim stress–induced internalization of CRH_1_ compared to males.^183^

While more sensitive to CRH, female LC neurons are less adaptable to high levels of CRH, which can occur during chronic stress. This is due to reduced CRH_1_ receptor internalization compared to males.^184^ Additionally, female LC CRH_1_ receptors are more highly coupled to the Gs‐protein, which can lead to prolonged NE release and heightened arousal during stress.^183^, ^185^, ^186^ These types of baseline LC–CRH sex differences may enhance female risk for stress‐induced cognitive impairments and anxiety by altering signaling in LC terminal regions. Indeed, CRH infusions into the LC produce theta oscillations in the medial PFC selectively in female rats but decrease low‐frequency activity in the medial PFC in males.^187^ These infusions increase and decrease medial PFC–orbitofrontal cortex coherence in females and males, respectively, but only alter orbitofrontal cortex activity in males, resulting in a delayed decrease in delta frequency power.^187^ These sex‐specific effects of CRH signaling in the LC are important to consider when assessing sex differences in the behavioral response to stress. In the cortex, CRH_1_‐Gs signaling is also enhanced in females, and this effect is linked to greater activation of AD pathways (Aβ and tau processing) and more cortical Aβ accumulation in CRH‐overexpressing female mice compared to CRH‐overexpressing male mice.^188^ Last, conditionally overexpressing forebrain CRH in adult male and female mice redistributes Aβ peptides in somatodendritic processes in the LC. However, only females exhibit increased colocalization of Aβ42 in LC axon terminals in the PFC and display more pronounced blood–brain barrier disruption.^189^ Thus, while the sex differences in LC–CRH interactions may lead to adaptations that support resilience in acute stress contexts, chronic CRH overactivation could predispose the female LC to metabolic overload, degeneration, Aβ accumulation, and tau pathology.

### 4.3 Mixed human evidence of sex differences in LC–NE structure and function

While animal models provide crucial mechanistic insights into sex‐specific LC vulnerability, human *post mortem* and neuroimaging studies offer the opportunity to validate and translate these preclinical findings across the lifespan and disease stages. However, compared to preclinical investigations, human work on the LC–NE system rarely explores sex differences because of the limited number of cases examined^190^, ^191^, ^192^, ^193^, ^194^, ^195^, ^196^, ^197^, ^198^, ^199^ or the exclusive use of women^200^ or men.^201^, ^202^, ^203^ The findings from those that do explore sex differences do not always align with results reported in rodents. Some studies report no sex differences in LC neuronal number, nucleolar volume, or melanin content across the lifespan,^204^, ^205^ while others observed more LC neurons and delayed cell loss in women.^205^, ^206^ Recent large‐scale autopsy studies in aging, AD, and other neurodegenerative disorders have also rarely examined sex differences in LC integrity.^1^, ^5^, ^6^, ^10^, ^137^, ^138^, ^207^, ^208^, ^209^, ^210^, ^211^, ^212^, ^213^, ^214^, ^215^, ^216^, ^217^, ^218^, ^219^ Among those that considered the effect of sex, findings varied: while some found no differences in LC volume,^165^ neuronal number,^162^, ^163^, ^165^, ^220^, ^221^, ^222^, ^223^ or hyperphosphorylated tau–positive LC neurons,^11^, ^162^, ^220^, ^222^, ^223^ others have found greater LC hypopigmentation, a proxy for neurodegeneration, in men.^224^, ^225^

Technical advances in imaging the LC with magnetic resonance imaging (MRI) have improved our ability to visualize the LC in vivo in humans, enabling reliable visualization and segmentation of this brainstem nucleus with high precision.^222^, ^226^, ^227^, ^228^, ^229^, ^230^, ^231^ These methodological breakthroughs have facilitated a growing body of research demonstrating the central role of LC structure and function in pathological manifestations of AD in humans, including early tau burden, cognitive decline, and clinical progression.^232^, ^233^, ^234^, ^235^, ^236^ Consequently, MRI‐derived LC integrity has emerged as a critical early biomarker for AD‐related neurodegenerative processes, showing promise for detecting at‐risk individuals.

Most in vivo MRI studies investigating sex differences in LC macrostructural integrity report no differences between men and women. These studies have used a combination of young and old adults,^226^, ^237^ older adults only,^238^, ^239^ cognitively normal individuals assessed across the lifespan,^227^, ^240^, ^241^ cognitively impaired older adults,^222^ and individuals with autosomal dominant AD.^166^ However, in an ethnically and socioeconomically diverse lifespan sample, some studies reported higher LC MRI signal intensity in women compared to men^242^ in both healthy young and old individuals,^243^, ^244^, ^245^, ^246^ and MCI patients^245^ who progressed to AD.^246^ There was one exception reporting lower LC intensity in women compared to men.^247^ Finally, three studies reported mixed findings on sex differences in LC microstructural integrity derived from diffusion‐weighted imaging and quantitative multiparametric mapping, showing either no differences in lifespan and older cohorts^248^, ^249^ or preserved LC microstructural integrity in men compared to women across young and older adults.^250^

Regarding LC function in humans, limited evidence suggests that healthy young and middle‐aged women exhibit lower functional connectivity between the LC and the hippocampus, parahippocampus, and middle temporal gyrus.^251^ One study reported older women with elevated levels of frontal Aβ burden show higher functional connectivity with somatosensory regions, suggesting women mount compensatory mechanisms to maintain optimal salience detection despite increased pathology.^252^ A well‐known role of the LC is in emotional processing^142^ and a meta‐analysis of 56 functional MRI studies using various emotional stimuli revealed higher activations in several brain regions in women, including the amygdala and hippocampus, and most notably in the LC.^253^ However, when specifically examining LC function during an emotional memory task, one recent study found no sex differences in LC activation in response to emotional salience, task‐related salience, and memory performance^254^; we interpret this with caution because the emotional stimuli used might not have been sufficiently intense to elicit sex differences in LC response. In addition, functional connectivity at rest between the left LC and the executive control network was higher in women than men, primarily driven by premenopausal women.^255^ Moreover, LC fluorodeoxyglucose positron emission tomography (PET) signals were higher in women compared to men for both cognitively healthy and impaired individuals,^256^ which suggests that greater LC metabolism may provide increased resilience against the effect of AD‐related processes.^257^

### 4.4 Future directions

Although animal models have established robust sex differences in LC structure and function, evidence for sex‐specific vulnerability to AD pathology remains incomplete, and inconsistent with human studies. Systematic investigation of these differences in both animals and humans remains crucial to advance the field.

Animal studies have shown that sex can modulate LC vulnerability in AD through several mechanisms. Specifically, females exhibit higher inflammatory responses after loss of adrenergic signaling, enhanced CRH_1_‐Gs protein coupling that leads to prolonged stress responses, and greater blood–brain barrier disruption, all of which could influence tau pathology initiation and clearance.^149^, ^179^, ^188^, ^189^ Regarding Aβ pathology, lesion and chemogenetic silencing studies have indicated that LC impairment can exacerbate Aβ deposition and associated neuroinflammation,^160^, ^168^ but the underlying mechanisms and whether they vary between sexes remain unclear and require targeted experimental investigation.

A major limitation across clinical as well as animal model studies is the lack of information about sex hormone–related factors in female participants (e.g., hormone levels, menstrual/estrous cycle phases, contraceptive use, menopausal/reproductive senescence status, history of gynecological surgery, or hormone replacement therapy), despite evidence that sex hormones (especially estradiol) modulate LC structure and function.^149^, ^226^, ^258^ In addition, most human studies have examined specific age ranges or have pooled diverse age groups without isolating the critical menopausal transition at which the risk of health problems rise in women^259^ including higher tau deposition.^260^ Finally, several studies have pooled cognitively normal and impaired participants together, limiting our understanding of sex‐specific patterns of LC changes across disease stages.

The vulnerability of the LC to declining estradiol combined with early tau accumulation might represent a key pathway explaining the well‐documented higher risk for women to develop AD, especially during menopausal transition and early life ovarian removal.^57^, ^261^, ^262^, ^263^, ^264^, ^265^, ^266^, ^267^, ^268^ However, critical gaps remain in our understanding of sex‐specific molecular and cellular mechanisms underlying such LC vulnerability to tau and Aβ pathogenesis, including sex differences in neuroimmune, neuroendocrine, and neurovascular regulation. Understanding how hormonal changes influence LC vulnerability during the lifespan, and how this association is modified by AD pathology, genetic risk factors and other environmental factors such as stress, especially during prodromal phases of the disease, could inform sex‐specific prevention strategies and optimal windows for intervention.

## 5 SEROTONIN

A common feature of early‐stage AD is the loss of 5‐HT neurons and the development of tau pathology in the dorsal raphe nucleus (DRN), suggesting a relative susceptibility of these neurons to the detrimental effects of protein aggregation.^12^, ^13^ The DRN is the largest of the 5‐HT–producing nuclei, but also contains GABAergic, glutamatergic, dopaminergic, and other peptidergic subtypes.^269^ Dysfunction of 5‐HT circuitry results in disruptions to sleep architecture and mood, particularly manifesting as depression.^270^, ^271^ Given that both depression and AD are more prevalent in women than in men,^272^, ^273^, ^274^ and that depression is both a risk factor for and a symptom of AD,^275^, ^276^, ^277^, ^278^ 5‐HT dysfunction may contribute to sex‐dependent vulnerabilities underlying AD pathology and progression.

### 5.1 Baseline sex differences in 5‐HT dynamics and function

5‐HT, one of the brain's most abundant monoamine neurotransmitters, is released in nearly all brain regions.^279^ In humans, PET imaging has shown higher 5‐HT synthesis rates in men^280^ and women,^281^ while cerebrospinal fluid studies indicate greater 5‐HT metabolism in women.^282^ Meanwhile, female rodents tend to exhibit faster rates of 5‐HT synthesis and turnover,^283^ particularly after 5‐HT depletion challenge.^284^, ^285^ These differences may partially be explained by female rodents’ higher expression of tryptophan hydroxylase, the primary rate‐limiting enzyme for 5‐HT production.^284^ Interestingly, in contrast to 5‐HT synthesis and metabolism, the rate of 5‐HT neuronal firing is ≈ 41% higher in male than female rats, possibly reflecting a compensatory biological adaptation.^286^

Sex hormones exert strong regulatory effects on 5‐HT signaling in the DRN. Both ERα and ERβ are expressed in DRN cells,^287^, ^288^ with ≈ 70% to 80% of receptor‐expressing cells being serotonergic.^288^ ERβ in DRN cells contributes to the regulation of estrous in rodents,^289^ and is expressed in a subset of DRN 5‐HT neurons projecting to the medial optic area in rodents, as well as in serotonin transporter (SERT)‐expressing DRN neurons in non‐human primates.^290^, ^291^ Within these neurons, ERβ directly regulates transcription of the tryptophan hydroxylase gene via an estrogen response element present in the gene's promoter region.^292^, ^293^ In female mice containing the ERβ null mutation, 5‐HT levels are significantly lower in postsynaptic brain regions compared to wild types.^294^ Genetic ablation of ERβ specifically in the DRN induces anxiety‐like behaviors in female mice, but not in males.^295^ Estrogens also modulate the expression of the enzymes responsible for 5‐HT degradation, including monoamine oxidase A in the brain and monoamine oxidase B in the periphery, in female rats.^296^ In addition to these effects, estrogen signaling suppresses binge‐like eating through ERα‐dependent activation of 5‐HT neurons in female mice,^297^ and modulates binge‐like alcohol drinking through ERα and ERβ‐dependent mechanisms in both sexes.^288^ Androgen receptors, in contrast, are mainly expressed in the male DRN, and are primarily observed in non‐serotonergic neurons.^298^ Moreover, neonatal gonadectomy in males increases 5‐HT release and reuptake in the hypothalamus,^299^ suggesting that early‐life masculinization contributes to sex‐dependent differences in 5‐HT signaling.

### 5.2 Sex differences in 5‐HT receptor expression and hormonal regulation

Sex differences in 5‐HT receptor (5‐HTR) expression vary across brain regions and physiological state. PET imaging showed reduced 5‐HT_2A_R binding in the frontal, parietal, temporal, and cingulate cortices of women compared to men.^300^ In rodents, females also have region‐specific differences in 5‐HT_2A_R mRNA levels compared to males; however, these transcriptional changes do not correspond to differences in 5‐HT_2A_R binding in these areas.^301^ 5‐HT_1A_R expression is greater in the hypothalamus and amygdala of male and in the hippocampus of female rodents; yet no sex difference in 5‐HT_1A_R binding has been detected in these areas.^301^ These findings are supported by the differential responses of male and female rodents to repeated restraint stress, with increased expression of 5‐HT_1A_R in the DRN of males and in the hippocampus of females.^302^, ^303^

Sex differences in 5‐HT function, via 5‐HTRs and SERT, appear to be strongly influenced by sex hormones. Although estrogens have not yet been shown to interact with every 5‐HTR subtype, experimental manipulations of estradiol levels in rodents affect 5‐HT_2A_R^304^, ^305^ and 5‐HT_1_R.^306^ Estradiol administration increases 5‐HT_2A_R density in the cerebral cortex and nucleus accumbens of female rats,^305^ and in the PFC of postmenopausal women.^307^ However, the effect of estradiol on postsynaptic 5‐HTRs appear to be cyclical, with reduced 5‐HT_1_R and 5‐HT_2_R expression during proestrus when estradiol levels are high, and increased expression during periods of low circulating estradiol.^306^ In parallel, 5‐HT binding to brain tissue is lower during proestrus and higher during estrous, again implying a cyclical nature to the expression of 5‐HTRs.^308^ On the other hand, ovariectomy of rodents decreases overall expression of 5‐HT_1_Rs,^306^ 5‐HT_2A_Rs,^305^, ^309^ and causes SERT dysfunction^310^ which can be rescued by supplementation with exogenous estradiol.^311^ Together, these findings indicate that sex hormones profoundly influence serotonergic function by modulating 5‐HT synthesis, degradation, and release; 5‐HTR expression patterns; and developmental dynamics in neurotransmission, thereby contributing to sex‐dependent differences in 5‐HT signaling.

### 5.3 Selective serotonin reuptake inhibitors and hormonal modulation in AD

The most widely used antidepressants, selective serotonin reuptake inhibitors (SSRIs), bind to SERT leading to reduced 5‐HT reuptake and increased 5‐HT concentration in the presynaptic terminals of 5‐HT neurons. The role of SSRIs in AD treatment has long been debated due to the relation among SERT, depression, and AD. Expression of SERT in cortical and limbic areas is reduced in patients with MCI^312^ and AD,^313^, ^314^ and this loss is more pronounced in AD patients with depression.^315^ Importantly, SERT itself is subject to hormonal regulation, where estradiol can interact with SERT to modulate 5‐HT signaling. For example, ovariectomy in female rodents^310^, ^311^ and macaques^316^ reduces SERT expression. These findings point to a close interaction between serotonergic regulation and sex hormones, which may help explain the observations that women are more likely than men to develop depression during early‐ and mid‐life,^272^, ^273^ and are twice as likely to develop AD.^274^ On the other hand, depression serves as a risk factor for AD.^317^ Moreover, mid‐ and late‐life depression may be secondary to early AD pathology, as AD‐associated depression correlates with tau and Aβ biomarkers,^276^, ^277^ and individuals with high genetic AD risk experience more depression in mid‐life.^278^ Therefore, enhancing 5‐HT transmission with SSRIs may represent a potential therapeutic approach in AD.

Experimental evidence shows SSRI treatment can reduce Aβ levels in rodents^318^ and in healthy older adults.^319^ SSRIs additionally reduced AD‐like pathology and improved cognitive function in AD mouse models (for review, see Mdawar et al.^320^). With respect to cognition, SSRI use has been linked to both positive and negative cognitive outcomes in AD patients.^321^, ^322^, ^323^, ^324^ Escitalopram, a common SSRI, is associated with a higher risk of developing dementia,^322^ and faster cognitive decline in individuals with dementia.^325^ Conversely, fluoxetine has been reported to enhance cognitive performance in AD patients.^326^ Both escitalopram and fluoxetine show high selectivity for SERT over other reuptake transporters, and both induce long‐term downregulation of pre‐synaptic 5‐HT_1A_Rs. However, fluoxetine additionally inhibits 5‐HT_2C_R and 5‐HT_3_Rs,^327^, ^328^ and exerts uncharacterized effects on 5‐HT_2A_R,^329^ suggesting that differential mechanisms could contribute to the efficacy of these medications. While more studies are needed to determine whether sex influences SSRI efficacy in AD, evidence that SSRIs are less effective in postmenopausal women and that estrogen therapy enhances SSRI response suggests that hormonal status may be a critical determinant of treatment efficacy.

The decline in estradiol after menopause accelerates AD progression in women,^330^ providing further evidence that female sex increases vulnerability to AD in advanced age. During normal aging, ER density increases in women and is negatively associated with mood and cognitive performance.^331^ In women with AD, loss of ERβ in the frontal cortex is more severe than in age‐matched controls, with much of this reduction localized to mitochondria.^332^ Reduced ERβ in the DRN has been linked to decreased 5‐HT synthesis,^293^ and ERβ loss impairs mitochondrial polarization,^332^ which is essential for both 5‐HT homeostasis and because 5‐HT itself supports mitochondrial function.^333^, ^334^ Therefore, exacerbated reductions in ERβ may be particularly detrimental, depleting 5‐HT as well as promoting neurodegeneration. Neuroimaging evidence further supports these associations: PET and MRI studies show that estrogen loss correlates with white matter degradation across several brain regions and a 30% greater burden of Aβ plaques compared to age‐matched men.^335^ Notably, exogenous administration of estradiol restores ERβ expression in the hippocampus of aging rodents,^336^ suggesting that estradiol therapy may help preserve serotonergic integrity and mitigate cognitive and affective decline in AD.

### 5.4 Sex differences in serotonergic systems in mouse models of AD pathology

Although the DRN has received comparatively less attention in AD research, growing evidence highlights its vulnerability to early tau pathology. Mouse models have additionally provided critical insight into how DRN dysfunction contributes to AD‐like prodromal symptoms. In mice expressing wild‐type htau, hyperphosphorylated tau appears in the DRN as early as 4 months of age, accompanied by reduced 5‐HT neuron density, decreased neuronal excitability, increased inflammation, and diminished serotonergic innervation of the entorhinal cortex and hippocampus.^178^ These changes coincide with the emergence of depressive‐like behaviors, preceding cognitive decline and underscoring the link between early 5‐HT dysfunction and prodromal symptoms in AD.^178^ In DRN‐targeted hyperphosphorylation‐prone htau expressing mice,^337^ social interaction deficits were observed in both sexes, while reward‐related behaviors were disrupted only in males, again at early time points and in the absence of cognitive impairment. A more selective DRN 5‐HT neuron‐targeted hyperphosphorylation‐prone htau mouse model revealed anxiety‐like behaviors and altered stress coping in both sexes, while social disinhibition and spatial working memory deficits were restricted to females.^338^ Notably, only females exhibited impairments in 5‐HT neuron excitability at an early stage of pathology. Together, these findings demonstrate that DRN tau pathology contributes to the early emergence of AD‐like behavioral symptoms and suggest that women may be particularly vulnerable to 5‐HT dysfunction during prodromal stages.

In several widely used AD mouse models, DRN 5‐HT neuron projections in postsynaptic regions were reported to be disrupted at relatively early ages. In 2‐month‐old 5xFAD mice, DRN 5‐HT positive projections were significantly reduced in the dorsal CA1 of the hippocampus, medial septum and lateral hypothalamus, accompanied by decreased Tph2 expression and lower 5‐HT levels compared to wild‐type mice.^339^ Remarkably, optogenetic activation of DRN 5‐HT projections in the dorsal CA1 was sufficient to reverse depressive‐like behaviors and cognitive impairments. Similarly, in hAPP‐J20 mice overexpressing human amyloid precursor protein with familial AD mutations, 5‐HT fiber density and 5‐HT_1A_R and 5‐HT_3A_R expression were diminished in the CA1 region.^340^ In this model, chemogenetic activation of median raphe 5‐HT neurons, which densely project to the CA1, restored circuit excitability and improved cognitive function independently of Aβ pathology. While these studies provide compelling evidence that 5‐HT dysfunction contributes to early behavioral and cognitive phenotypes through CA1 projections, they did not consider sex as a biological variable, highlighting an important direction for future studies.

### 5.5 Future directions

Despite substantial evidence linking serotonergic dysfunction to AD progression and sex‐specific risk, the underlying mechanisms remain unclear. Recent studies using DRN‐targeted mouse pathology models have started to reveal early vulnerability and sex‐specific effects,^178^, ^337^, ^338^ establishing a valuable platform to investigate how 5‐HT dysfunction contributes to AD progression. Hormone manipulations and sex chromosome analyses will be crucial for identifying the biological substrates of sex‐specific susceptibility to AD in both mouse models and humans. A critical direction is to establish whether hormonal windows of vulnerability act though serotonergic pathways to influence risk factors such as depression and to what extent this accelerates AD progression. Given the early implication of 5‐HT in AD, and the substantial basal sex differences in the serotonergic system, future work should use both preclinical and clinical approaches to define the sex‐dependent therapeutic potential of SSRIs. Clinical trials should stratify participants by sex, menopausal status, and genotype to determine whether any type of estrogen supplementation modifies SSRI effects on serotonergic signaling and cognition during peri and postmenopausal periods. Clarifying the interaction among sex steroids, 5‐HT function, and AD risk will be essential for developing treatment strategies, especially for women who face disproportionately higher AD risk.^341^

## 6 CORTICOTROPIN RELEASING HORMONE

One common biological mechanism conferring susceptibility to neuropsychiatric symptoms, which puts individuals at a higher risk of developing AD, is stress.^342^, ^343^, ^344^ CRH is one of many neuropeptides that mediate the autonomic, behavioral, endocrine, and immune responses to stress via binding at two G protein‐coupled receptors, CRH_1_ and CRH_2_.^345^, ^346^, ^347^, ^348^ CRH_1_ regulates cortisol (corticosterone in rodents) output from the hypothalamic‐pituitary‐adrenal axis, which is pronounced in anxiety, depressive disorders,^349^, ^350^, ^351^, ^352^ and AD.^353^, ^354^, ^355^ Specifically, AD patients exhibit several stress system abnormalities including elevated levels of cortisol,^356^, ^357^, ^358^ reduced CRH‐positive cells, and upregulated CRH_1_ expression in the cortex.^359^, ^360^, ^361^ These increases in cortisol often occur at the MCI stage before AD pathophysiology becomes severe^353^, ^358^, ^362^, ^363^ and are associated with faster rates of cognitive decline.^353^, ^362^ Like humans, evidence from transgenic models suggests that increased corticosterone levels often precede Aβ plaque formation.^364^, ^365^, ^366^ Later stages of AD, characterized by worsening memory and cognitive impairments, involve CRH_1_‐dependent Aβ‐^366^, ^367^, ^368^, ^369^, ^370^, ^371^ and hyperphosphorylated tau‐induced^372^, ^373^, ^374^ hippocampal dysfunction. For instance, chronic stress or intra‐hippocampal CRH infusions increase levels of hippocampal Aβ and hyperphosphorylated tau, effects that are blocked by CRH_1_ antagonists.^370^, ^373^, ^374^ AD mice heterozygous or null for CRH_1_ have reduced hippocampal Aβ levels,^375^ while mice overexpressing CRH have elevated hyperphosphorylated tau^188^, ^376^ and Aβ plaques.^188^, ^369^ Furthermore, AD mice exhibit higher levels of CRH within stress circuitry and an anxiogenic phenotype, which are eliminated in AD mice heterozygous for CRH_1_.^364^ Collectively, these data highlight a critical role for CRH_1_ in modulating both early‐ and late‐stage AD clinical pathology and underscore the need for future research examining sex differences in the role of central CRH_1_ systems in driving AD pathology across various stages of the disease.^377^

### 6.1 Sex differences in CRH levels and interactions with stress

Human^378^ and rodent^379^, ^380^, ^381^, ^382^ adult females generally exhibit higher baseline hypothalamic CRH levels compared to males, which is associated with elevated levels of corticosterone and heightened anxiety in female rodents.^383^, ^384^, ^385^, ^386^, ^387^, ^388^ These sex differences in hypothalamic CRH expression appear to be age dependent. For example, at 6 months, male mice have elevated hypothalamic CRH levels compared to females, whereas at 18 months females exhibit a trend toward higher levels.^389^ Additionally, in female mice, a significant increase in hypothalamic CRH levels occurs during aging, which is absent in males.^389^ Although studies examining sex differences in baseline cortisol levels in humans are mixed,^390^ there are reports of baseline cortisol levels increasing as women transition through puberty, while at the same time, levels decrease in men.^391^ Interestingly, one study reported no differences in baseline cortisol levels in younger adults (22–36 years), but in older adults (67–88 years), women exhibited higher cortisol levels. This finding suggests that increased hypothalamic‐pituitary‐adrenal axis output during aging could be one mechanism underlying the heightened risk for AD in women.^392^

In response to stress, female rats exhibit higher levels of CRH in the paraventricular nucleus of the hypothalamus compared to males,^380^, ^393^ although this sex effect is stressor^393^ and age^389^ dependent. Compared to men, intravenous CRH administration increases adrenocorticotropic hormone and cortisol levels in both adult and adolescent women, respectively.^391^, ^394^ Last, a meta‐analysis examining challenge‐induced cortisol release in humans found that older subjects (69 ± 6 years) exhibited a larger cortisol response than younger subjects (28 ± 5 years), an effect that was significantly larger in women than men.^395^ To clarify the discrepancies in baseline sex differences for cortisol in humans, future studies need to control for the role of sex hormones in regulating hypothalamic‐pituitary‐adrenal axis output, the age of the participants, and the potential influence of circadian patterns on cortisol release.^390^, ^396^

### 6.2 Sex differences in CRH receptors and signaling

Studies examining baseline sex differences in CRH_1_ expression indicate unique distribution patterns in hypothalamic nuclei between males and females. There is a trend for sex differences in total hypothalamic CRH_1_ expression across the lifespan in mice, with females showing an increase compared to males when collapsed across age.^389^ Interestingly, 18‐month‐old males and females exhibit increased hypothalamic CRH_1_ levels compared to 1‐month‐old animals of the same sex, suggesting increases in hypothalamic CRH_1_ may be associated with increased probability of AD progression during aging regardless of sex. Intriguingly, in a series of studies using a CRH_1_ reporter mouse line,^397^ two discrete hypothalamic nuclei displayed sex‐specific patterns of CRH_1_‐expressing cell clusters; in females, higher baseline CRH_1_ levels were found within the anteroventral periventricular nucleus and in males higher CRH_1_ levels were found in the paraventricular nucleus of the hypothalamus.^398^, ^399^ These CRH_1_ cell groups showed sex differences in cellular activation after acute restraint stress, with an increase in CRH_1_ activity in the anteroventral periventricular nucleus in females and an increase in CRH_1_ activity in the paraventricular nucleus of the hypothalamus in males. These effects also occur after 9 days of chronic variable stress.^400^ Interestingly, postpartum female mice have elevated CRH_1_ levels in the anteroventral periventricular nucleus compared to nulliparous females, as well as an increase in restraint stress‐activated anteroventral periventricular nucleus CRH_1_ neurons.^401^ It would be interesting to know if these sex differences in CRH_1_ expression change across the lifespan, as previous reports examining the entire hypothalamus indicate age‐dependent effects with females exhibiting higher CRH_1_ levels compared to males at 12, but not 1, 6, or 18 months of age.^389^ In summary, these data suggest that differences in hypothalamic subnuclei CRH_1_ distribution may contribute to sex differences in various stress‐related disease states including AD.

### 6.3 Sex as a mediator of CRH dysfunction in AD

Although many studies report an impact of corticosterone on pathology and behavioral outcomes in AD in males and females,^402^, ^403^, ^404^ recent research indicates that sex differences also play an important role. Specifically, rodent AD models show that females demonstrate greater sensitivity to stress manipulations, including heightened anxiety,^405^ whereas results from memory tests are mixed.^405^, ^406^, ^407^ In addition to behavioral outcomes, studies report sex‐dependent changes in AD pathology after stress, with female mice exhibiting greater Aβ, tau, and inflammation.^405^, ^408^ Cortical phosphoproteomic responses to chronic stress are also largely sex specific.^405^ However, in humans, men with amnestic MCI were more likely to show deficits in episodic memory after acute psychosocial stress and express higher cortisol levels compared to normal aging men and women.^409^ In sum, these findings are supportive of a role for sex in determining behavioral and neurobiological outcomes after stress. However, studies are yet to identify specific neural systems mediating these outcomes.

In AD, stress also has a sex‐specific impact on CRH systems. Several studies in AD models report that females exhibit a significantly greater corticosterone response to stress, which may ultimately influence the expression of AD‐related pathology.^405^, ^407^, ^410^ Additionally, elevated corticosterone levels are observed in female but not male AD mice in the absence of an explicit stressor.^411^ This may occur due to lower basal CRH levels in the paraventricular nucleus of the hypothalamus in males.^402^, ^411^ In human subjects, elevated cortisol during midlife is associated with the highest Aβ burden in cortical regions 15 years later.^412^ Critically, this association was significant only in women, particularly those who were postmenopausal. Recently, several studies identified sex differences in CRH_1_ receptor pathways that may mediate the increased vulnerability in females to AD pathology after stress.^183^, ^188^ Administering a CRH_1_ antagonist prior to stress blocked the subsequent increase in expression of hippocampal Aβ only in female AD mice.^408^ Administration of inhibitors of protein kinase A or extracellular regulated kinase, which are activated by CRH_1_, also block the expression of Aβ in the hippocampus, suggesting that a CRH_1_/protein kinase A/extracellular regulated kinase signaling pathway may mediate female‐specific effects of stress on AD pathology.^408^ In males, the expression of β‐arrestin is thought to reduce CRH_1_ signaling and thereby offer protection from the effects of stress on AD pathology.^183^ Consistent with this hypothesis, β‐arrestin knockout male mice showed elevated Aβ expression in response to stress.^408^ Taken together, these studies provide important insight into components of CRH signaling pathways that mediate sex‐specific effects of stress on AD pathology.

### 6.4 Future directions

CRH systems are implicated in neuropsychiatric symptoms that increase AD risk as well as AD pathology. To further elucidate the role of hypothalamic CRH in AD progression, levels of corticosterone, CRH, and CRH_1_ expression should be examined in rodent AD models at timepoints prior to disease onset and during the earliest stages of disease. Ideally, these studies will help clarify discrepancies in CRH expression reported between rodent AD models^402^ and *post mortem* human brains.^359^ Additionally, while numerous studies have examined the causal role of hippocampal CRH in regulating AD pathophysiology, a mechanistic understanding of sex differences in CRH_1_ signaling pathways, neural circuits implicated, and the downstream consequences of such sex differences are understudied and thus, poorly understood. Studies manipulating the hypothalamic CRH systems in AD models are needed to further define the role of CRH in AD progression. Sex differences in hypothalamic nuclei CRH_1_ distribution likely contribute to sex differences in stress‐related disease states, but studies are needed to determine whether this effect extends to AD models and how this may affect AD progression. Last, future research examining sex differences in CRH–LC signaling in AD models is needed, particularly across the lifespan and reproductive stages, to determine whether this stress system represents causal mechanism of heightened AD risk in women.

## 7 OXYTOCIN

OXT is a small neuropeptide produced in the paraventricular, supraoptic, and accessory nuclei of the mammalian hypothalamus.^413^ OXT is stored in large dense‐core vesicles located in the soma, dendrites, and along the axon. OXT was initially believed to be released as a hormonal factor from the somatic and dendritic regions and largely had effects in the central nervous system via volume transmission to reach presynaptic neurons at a distance. More recently, axon fibers projecting throughout the brain have been identified which, upon stimulation, can release OXT in target regions.^414^ Upon release, OXT binds to the G‐protein coupled OXT receptor, which canonically stimulates the Gq pathway. However, it has also been shown to lead to Gi and Go activation.^415^ OXT neurons project to brain areas implicated in AD pathogenesis, including the hippocampus, cerebral cortex, and amygdala, all of which express OXT receptors to varying degrees.^413^, ^416^ While OXT is well known for its peripheral physiological effects (e.g., milk let‐down response and fetal ejection), there is strong evidence highlighting its central effects.^413^ OXT's role in modulating social behavior is particularly relevant in the context of AD.^413^ Social withdrawal is an early symptom of AD,^3^ and social isolation increases the risk and progression of dementia.^417^

### 7.1 Baseline sex differences in the OXT system

Sex differences in the OXT system across species have been recently reviewed elsewhere.^418^, ^419^, ^420^, ^421^ A host of studies indicate a lack of sex differences in the number of OXT neurons and innervation density.^422^, ^423^, ^424^, ^425^, ^426^, ^427^, ^428^, ^429^, ^430^, ^431^, ^432^, ^433^ When sex differences are present, females tend to show greater numbers of OXT neurons and innervation than males.^424^, ^425^, ^426^, ^434^ Meanwhile, OXT receptor expression shows largely the opposite pattern, with males displaying higher levels of OXT receptors than females.^433^, ^435^, ^436^, ^437^, ^438^, ^439^ Thus, there is a potential sex distinction in the structure of the OXT system at the level of the cell bodies versus target regions. However, such sex differences, or lack thereof, are highly brain region and species specific.^418^, ^419^, ^422^, ^423^, ^424^, ^425^, ^426^, ^427^, ^428^, ^429^, ^430^, ^431^, ^432^, ^433^, ^436^, ^438^, ^440^, ^441^, ^442^, ^443^, ^444^ OXT receptor expression is further modulated by gonadal hormones, likely through ERα. Testosterone administration to neonatal female rats leads to higher OXT receptor densities whereas gonadectomy of adult male and female rats decreases OXT receptor density.^438^, ^444^ OXT receptor expression is also higher in estrous compared to non‐estrous females, but still significantly lower than males.^433^ Of note, sex differences are most commonly reported in rodent species whereas a vast majority of human and non‐human primate studies indicate no sex differences in characteristics of the OXT system.^418^ Such species differences are important to keep in mind when considering sex as a variable in AD‐related OXT dysfunction, in addition to the translational value of any preclinical findings.

However, OXT itself promotes sex‐specific behaviors, including promoting sexual behavior in males and parturition and post‐partum behavior in females.^421^, ^445^, ^446^, ^447^ However, a major focus of OXT research in AD should be on the potential impact it has on social behavior,^448^ especially given its sex‐specific effects. OXT has been most well studied in its facilitatory role in forming partner preferences in monogamous species. This includes prairie voles and humans, with effects typically being stronger in females.^449^, ^450^ However, such effects are not exclusive to females, as antagonism of OXT receptors in the lateral septum blocks pair bonding and brain‐wide knockout of the OXT receptor reduces consolation behavior in male prairie voles.^451^, ^452^ In non‐monogamous species, such as rats, OXT administration can improve social recognition and reverse social avoidance in males, but not females.^453^, ^454^, ^455^, ^456^ Interestingly, early‐life manipulations of the OXT system can have effects well into adulthood. For example, neonatally applied OXT induces aggressive mate‐guarding behavior in adult female prairie voles.^457^ Neonatal antagonism of OXT receptors also decreases alloparental care in male prairie voles and social approach behavior in female CD‐1 mice.^458^, ^459^ Comparing sex differences in the effects of OXT across species indicates the presence of some species‐specific behavioral effects that are seemingly in opposition (e.g., greater behavioral effects in female prairie voles and male rats, but relatively similar effects in both sexes in humans).^418^, ^419^ Together, these results again underscore the need to consider species in the sex‐specific effects of OXT. In humans, OXT can also induce sex‐specific behavioral effects.^418^, ^419^, ^420^, ^460^, ^461^, ^462^, ^463^ Underlying sex differences in the neurobiological effects of OXT may lead to these behavioral differences but, in some situations, may also culminate in similar behavioral output in males and females.^418^

Effects of early‐life OXT manipulation on late‐life phenotypes and in the pathophysiology of AD are interesting to consider but have yet to be explored. Given the high prevalence of social deficits in AD,^3^ understanding the contributing role of OXT dysfunction is of great importance. Further delineating the moderating influence of sex is also critical given the sex differences in AD symptomology. While women typically suffer greater incidence of neuropsychiatric symptoms,^19^, ^125^, ^464^ men exhibit more severe symptoms in the social domain such as aggression, apathy, and agitation.^125^ Neural underpinnings of such differences have yet to be identified, but they might be partially due to single nucleotide polymorphisms in the OXT receptor gene which have sex‐specific effects on social behavior.^418^ OXT is also known to reduce anxiety in both sexes, and appears to influence memory, with clear relevance to AD, but more work needs to be done to solidify its precise effects and any dependence on sex.^421^

### 7.2 OXT dysfunction in AD patients and models

Preclinical studies of OXT dysregulation in AD rodent models reveal downregulation of OXT levels in both male and female APP/PS1^465^, ^466^ and female B6.APBTg mice^467^ and reduced serum OXT levels in male APP/PS1 mice.^468^ However, these studies did not compare results between females and males and therefore do not account for any potential impact of sex in these AD models.

Human studies on the impact of AD on the OXT system present conflicting evidence. One of the first studies of OXT neurons in tissue from human AD patients used cell number and size as proxies for peptide production, and no significant differences were observed for OXT neurons compared to normal aging.^429^ In this study, men and women were pooled due to a lack of significant morphological differences between sexes. These results were replicated in later studies analyzing normally aging populations and AD patients.^430^, ^431^ There were similarly no sex differences in morphological parameters of OXT neurons in the supraoptic or paraventricular nucleus,^431^ though sample sizes were low. Another study in male patients showed no significant difference in cerebrospinal fluid OXT levels.^469^ In another study including samples from men and women,^470^ AD patients displayed increased levels of OXT in the hippocampus and temporal cortex. In contrast, recent work that included men and women reported lower serum OXT levels in AD patients.^471^ Two other recent studies show OXT signaling pathway dysregulation in the blood and entorhinal cortex of AD patients.^472^, ^473^ In the case of the entorhinal cortex, this phenotype was only observed in men. These studies highlight how a failure to evaluate sex‐specific changes in the OXT system can lead to conflicting findings and hinder our understanding of AD‐related dysfunction. If there are sex differences in the effects of AD on the OXT system, future work could focus on evaluating the use of OXT‐based therapeutics. Moreover, human studies investigating OXT system dysfunction in AD patients are limited and variable, likely due to the complexity of the system, the heterogeneity of the populations studied, the lack of sex‐stratified analyses, and the limitations of the available methods. Furthermore, circuit‐specific defects that emerge in a disease stage–specific manner may not be captured by the approaches described above. Given these limitations, the involvement of the system as a pathological hallmark in human patients remains to be fully determined.

### 7.3 OXT as a potential treatment for AD

While OXT is well tolerated and improves social symptoms in the context of frontotemporal dementia,^474^, ^475^, ^476^ there is only one study with a small number of AD participants revealing limited but positive outcomes on social cognition after intranasal OXT treatment.^477^ However, low sample sizes have precluded analyzing the potential modifying effect of sex on patient outcomes. Meanwhile, the number of preclinical studies evaluating OXT as a therapeutic approach in AD has increased in the last few years. Such studies demonstrate the protective effects of exogenous OXT treatment at the molecular, cellular, network and behavioral levels. In vitro, the use of primary cultures or cell lines allow for the study of the effect of OXT after exposure to Aβ or other factors related to AD pathology.^465^, ^478^ Work on network‐ and synaptic‐level responses has been performed in brain slices ^465^, ^479^ and organoids.^480^, ^481^ These studies suggest that the protective effects of OXT in AD models are mediated by changes in synaptic plasticity,^479^ inflammation,^465^, ^480^ and cell death.^478^ OXT protects against Aβ‐induced toxicity through the extracellular regulated kinase pathway, both in the PC12 cell line^478^ and in hippocampal slices.^479^ OXT is also anti‐inflammatory^482^ and can reduce microglial activation in AD models^465^, ^483^, ^484^ through inhibition of Toll‐like receptor 4–mediated pro‐inflammatory signaling^483^ and extracellular regulated kinase/p38 mitogen‐activated protein kinase and cyclooxygenase‐2 /inducible nitric oxide synthase nuclear factor kappa beta signaling pathways.^484^ Using human induced pluripotent stem cell–derived cerebral organoids revealed that OXT further enables Aβ clearance by upregulating triggering receptor expressed on myeloid cells 2, a key modulator of microglial phagocytosis.^481^ Together, evidence collected in vitro supports OXT as a therapeutic approach to target AD‐associated pathways.

In preclinical AD models, multiple groups have shown that exogenous OXT can rescue behavioral and molecular AD‐related phenotypes.^465^, ^468^, ^483^, ^484^, ^485^, ^486^, ^487^, ^488^, ^489^ The protective effects of OXT observed in mouse models have been proposed to be mediated by reducing cell death^484^, ^487^ or inflammation,^468^, ^484^, ^487^ and by increasing Aβ clearance.^466^, ^468^, ^484^, ^485^, ^487^, ^488^ At the behavioral level, cognitive benefits of OXT have been the primary focus in AD models.^465^, ^484^, ^485^, ^486^, ^487^ Comparatively, there are few studies looking at the effects of OXT on social behaviors in these models. Intranasal OXT rescued the reduced sociability in APP/PS1 male mice.^465^ Other work has assessed the consequences of OXT treatment on social memory using the 5‐trial social task and found that OXT protects against social memory loss in APP/PS1 male mice.^465^ In contrast, other studies have not observed altered sociability in APP/PS1 females, but rather showed reduced sociability in this model *after* OXT treatment.^485^ Whether these differences in OXT effects are due to sex‐specific mechanisms or variations in experimental protocols is unknown, and should be investigated in future studies.^490^

### 7.4 Future directions

Research into sex differences in dysfunction of the OXT system in AD and the potential therapeutic benefits of exogenous OXT is in its infancy. More studies are needed to understand whether and to what extent the OXT system is impacted in AD, and the moderating effects of sex. However, one of the main limitations in probing this system is the lack of reliable methods to measure brain levels of OXT. OXT is usually measured using enzyme‐linked immunosorbent assay and these measurements are mostly done in blood or saliva. Unfortunately, there is some evidence showing that such samples are not direct readouts of OXT levels in the brain.^491^ Alternatively, *post mortem* histological analysis of human brain tissue using validated and reliable OXT antibodies could reveal changes in cell number, innervation, receptor distribution, and levels throughout the brain.

Exogenous OXT has been proposed as a treatment for AD, due in part to the early constellation of social dysfunction that persists throughout disease course. Yet, much of this work is based on relatively limited preclinical evidence showing benefits of OXT in AD models, both at the neuropathological and behavioral levels. Given the stark species differences in the OXT system, more work needs to be done, and caution should be taken when interpreting and translating findings from preclinical models to humans. Considering these factors highlights the need for more work at the human and *post mortem* levels. Human studies can be further expanded to include robust clinical trials testing the therapeutic effects of OXT in AD patients. When designing such trials, dose and administration protocols need to be carefully defined, including route of administration, treatment duration, and the inclusion of behavioral therapy as part of the treatment. Such considerations are due to the fact that OXT has been shown to increase the salience of social behaviors,^413^, ^492^, ^493^ opening the possibility of achieving greater therapeutic benefits in positive social contexts. Although human and non‐human primate research shows few sex differences in the OXT system, controlling and analyzing results by sex will help clarify any potential modifying effects of sex on the therapeutic benefits of OXT treatment.

## 8 ARGININE VASOPRESSIN

AVP is a hormone with wide‐ranging effects on physiology and behavior. AVP‐expressing neurons are primarily located in the hypothalamus, with projections extending to the basal forebrain, midbrain, and brainstem nuclei. Research across species has extensively characterized the AVP neuronal system, highlighting its conserved biological roles across species to regulate neurosecretion, sleep/wake cycles, circadian rhythms, social behaviors, and the stress responses.^494^, ^495^ There are also extra‐hypothalamic AVP neuronal populations that display intrinsic differences based on sex.^496^ Given that disturbances in homeostatic regulation are common in AD, studying the AVP system function/dysfunction and its associated dependence on sex may provide insights into disease mechanisms.

### 8.1 Dispersed organization of the AVP system and underlying sex differences

In the hypothalamus, AVP neurons are divided into three major nuclei, each with distinct organization and specialized functions. AVP neurons in the supraoptic nucleus are magnocellular and regulate water balance and blood pressure.^494^, ^497^, ^498^ Suprachiasmatic nucleus AVP neurons are parvocellular and orchestrate circadian rhythms.^499^, ^500^ The paraventricular nucleus includes both magnocellular and parvocellular AVP neurons that regulate social and emotional behaviors, modulate autonomic activity, and stimulate the release of adrenocorticotropic hormone, which is essential for stress responses.^494^, ^497^, ^501^, ^502^ Collectively, these hypothalamic AVP neurons are both neuroendocrine and neuromodulatory, with axonal projections to extra‐hypothalamic regions such as the basal forebrain, midbrain, and brainstem. They are conserved in form and function across species.^418^, ^421^, ^496^ AVP axonal projections emanating from hypothalamic areas are typically denser in males than females.^503^, ^504^

Immunohistochemistry, RNAscope, and retrograde tracing studies have also revealed AVP‐expressing neurons in extra‐hypothalamic areas such as basal forebrain and amygdala. AVP neurons in these regions modulate social, emotional, and anxiety‐related behaviors. The bed nucleus of the stria terminalis in the basal forebrain coordinates acute stress responses by inhibiting CRH secretion and influencing paraventricular AVP activity.^505^ The amygdala plays a central role in fear and anxiety regulation.^506^ Within the amygdala, the central nucleus serves as the primary output region orchestrating behavioral and physiological fear responses,^501^ while the basal, lateral, and medial subdivisions process and integrate incoming information. AVP signaling reduces innate fear responses through local GABAergic neurons and provides feedback to the hypothalamic–pituitary–adrenal axis, thereby linking emotional regulation with neuroendocrine responses.^501^, ^507^, ^508^, ^509^ AVP neurons in these regions contribute directly to sex differences in behavior. In prairie voles, AVP injected into the lateral septum increased paternal responsiveness.^510^ Similarly, the number of AVP neurons in the amygdala is greater in male mice in response to testosterone.^507^ Structural investigations corroborate these findings; quantification of AVP immunoreactivity revealed that AVP neurons and fibers are more abundant in males than females in the septal nucleus, bed nucleus of the stria terminalis, and amygdala.^511^, ^512^, ^513^, ^514^

AVP neurons are also present in other hypothalamic regions, such as the zona incerta and median eminence. Although these nuclei do not contain large populations of AVP‐synthesizing neurons, AVP immunoreactivity is detected in synaptic terminals projecting from the hypothalamus. While sex differences in these areas have not been thoroughly studied, both the zona incerta and median eminence play important roles in regulating the hypothalamic–pituitary–adrenal axis in response to stress,^495^, ^503^, ^515^ which is a sex‐dependent process (see section 6).

AVP release exerts its effects via binding to two G‐protein coupled receptor subtypes, AVPR1A and AVPR1B.^516^ AVPR1A receptors are widely expressed across multiple brain regions, whereas AVPR2B receptors exhibit more restricted distribution within the hippocampus, amygdala, olfactory bulb, and hypothalamic–pituitary–adrenal axis.^418^, ^517^, ^518^, ^519^ AVP receptors expressed in subcortical regions exhibit marked sex differences with direct implications for behavior. Autoradiographic quantification reveals higher densities of AVP binding in the ventromedial hypothalamus and premammillary nuclei of male hamsters compared to female hamsters.^520^ In addition, AVPR1A binding densities are also higher in male Wistar rats, showing distinctive subregional differences in the hypothalamus and basal forebrain such as the medial posterior bed nucleus of the stria terminalis, anteroventral thalamus, tuberal lateral hypothalamus, and stigmoid hypothalamus.^418^ Behaviorally, blocking AVPR1A receptors in the DRN and lateral habenula reduces social behaviors, particularly urine marking, ultrasonic vocalization, and territorial aggression in male mice, but has no effect in females.^521^ More recently, a study reported sex‐specific distributions of AVPR1A receptors across mouse subcortical regions, reinforcing the previous findings that AVP receptor localization shapes AVP neuronal function. These findings provide targets for investigating the mechanisms underlying sex differences in AVP function.^517^

Overall, most studies^418^, ^421^ indicate that, compared to females, the male AVP system comprises larger neurons, greater amounts of AVP mRNA, and higher fiber density, receptor binding, and levels in plasma and urine.^423^, ^425^, ^428^, ^431^, ^432^, ^436^, ^520^, ^522^, ^523^, ^524^, ^525^, ^526^, ^527^, ^528^, ^529^, ^530^, ^531^, ^532^, ^533^, ^534^, ^535^, ^536^, ^537^, ^538^, ^539^, ^540^, ^541^, ^542^ There are some reports of the opposite or null effects, but species differences are much less pronounced compared to the OXT system.^543^, ^544^, ^545^

### 8.2 AVP neurons show differing susceptibility in AD patients

The cytoarchitecture of AVP neuronal hubs demonstrate region‐dependent susceptibility in healthy aging and AD, with some modulating influence of sex. In the two major AVP magnocellular hubs, the paraventricular and supraoptic nuclei, human studies across normal aging and in AD found no significant sex differences in total cell number or volume.^546^, ^547^, ^548^ However, in old rodents (> 24 months) and humans (> 80 years) cellular hypertrophy is observed in the paraventricular and supraoptic nuclei that is absent during midlife.^429^, ^549^ Several human studies further suggest that AVP neurons remain activated in old age, displaying enlarged individual cell size in both normal aging and AD without neuronal loss.^550^, ^551^ This finding is rather atypical compared to other subcortical nuclei, which usually display severe loss of cell bodies and subsequent neurotransmission. However, these conclusions are limited by the small number of human studies and pooled analyses, underscoring the need for larger scale stereological studies that would be able to clearly identify any sex differences. In fact, evidence from quantitative stereology in rhesus monkeys suggests possible sex differences. There was a significant increase in neuron and glia counts in the male paraventricular nucleus with age, particularly > 20 years (roughly equivalent to > 60 years in humans).^552^ Cellular hypertrophy also correlated with age, though the effect did not reach significance.

Suprachiasmatic nucleus AVP neurons play a critical role in regulating circadian rhythms and are selectively vulnerable to AD‐associated pathology. Suprachiasmatic nucleus neuron number declines in individuals > 80 years, in contrast to the stable, or even increased number, in animal models.^547^, ^553^ In AD, suprachiasmatic nucleus AVP neurons are also significantly reduced.^547^, ^553^, ^554^ However, evidence for sex differences in suprachiasmatic nucleus degeneration is limited. Although some data suggest a possible male‐biased decline, this was not statistically significant.^547^, ^553^ Recent analyses combining quantitative histology and proteomics in AD brains reported neither sex‐specific neuronal loss nor tau accumulation in suprachiasmatic nucleus AVP neurons.^554^ This study also found no significant age‐related changes, though data from individuals > 80 years remain sparse. Although circadian rhythms themselves differ between sexes^555^, ^556^ and circadian disruption in AD often presents with sex‐specific features,^557^ the neuronal influences, including that of AVP, underlying these sex differences in both baseline circadian rhythms and disease‐related changes in AD remain unresolved. Addressing this gap will require studies that integrate clinical phenotypes with neuropathological findings, while accounting for AD heterogeneity, demographic variables, and intrinsic biological factors, including well controlled cohort studies that include sex as a variable.

Age‐related AVP neuronal loss outside these brain regions has been documented.^511^, ^512^, ^513^, ^514^ However, studies directly quantifying AVP neuronal loss in AD, and whether this differs between sexes, remain limited. For regions like the basal forebrain nuclei, which regulate social behaviors, fear, and anxiety, understanding sex‐specific AVP phenotypes in AD is of particular importance.

### 8.3 Future directions

The AVP system, composed of multiple dispersed brain regions, is a critical modulator of physiology and behavior often disrupted in AD. The function of AVP neurons in the brain depends on their receptors and the target brain regions receiving efferent projections. Such effects are further modulated by sex differences, with healthy males across species typically displaying greater AVP function and sensitivity to AVP interventions. The diffuse nature and distinct functional outputs of each AVP region raises the interesting possibility that different AVP‐expressing regions contribute to specific facets of AD. Yet, the overall extent and nature of these changes remain poorly characterized, highlighting the need for comprehensive preclinical and clinical studies that include sex as a factor. Furthermore, AVP‐synthesizing regions appear to be separable into either selectively vulnerable or resistant to AD, but the mechanism for these differences is not well understood. Because hypothalamic AVP‐synthesizing neurons act as central regulators of extra‐hypothalamic AVP circuits, investigating sex differences within this system is of high importance. Future research should move beyond localized characterization and toward a more integrated, intra‐network understanding. This includes interactions with other subcortical neuromodulatory systems, such as CRH and OXT, the latter of which frequently colocalizes with AVP circuits and also exhibits some sex differences.^457^, ^458^, ^558^, ^559^

## 9 HISTAMINE

HA, also known as 1H‐imidazole‐4‐ethanamine or ergamine, is a low molecular weight endogenous alkylamino compound that plays a critical role in wakefulness, cognition, and immune regulation.^560^, ^561^ In the brain, the sole histaminergic hub is the posterior hypothalamic tuberomammillary nucleus (TMN). The histaminergic neurons of TMN synthesize HA through oxidative decarboxylation of L‐histidine by a rate‐limiting enzyme, L‐histidine decarboxylase, in the presence of co‐factor pyridoxal‐5′‐phosphate.^562^ In the human brain, the TMN constitutes a diffusely organized population of large HA neurons (diameter 25–40 microns), located at the intersection of the caudal tuberal and rostral mammillary regions. These multipolar neurons have three to six primary dendrites and contain darkly stained peripheral endoplasmic reticulum, with typical irregularities in the cell membrane. These cells are also characterized by substantial lipofuscin aggregation.^563^, ^564^ The number of HA neurons in humans varies between 64,000 and 150,000 neurons.^212^, ^563^, ^565^, ^566^ Although the TMN forms the core of the medial hypothalamic zone, it extends substantially into the lateral hypothalamic zone.^567^ In addition to TMN HA neurons, mast and endothelial cells also produce traces of HA in the brain.^568^

As a part of the monoaminergic extra‐thalamic pathways, TMN HA neurons maintain reciprocal connections with the OX/hypocretin neurons of the lateral hypothalamus and LC–NE neurons to promote wakefulness.^569^, ^570^ Besides these reciprocal connections, the unmyelinated axons of the TMN HA neurons send widespread dense innervations to hypothalamic sleep‐promoting nuclei and the basal forebrain, as well as diffuse innervations to the neocortex.^567^, ^571^ HA neurons exert their neuromodulatory function through the four G‐protein‐coupled metabotropic histamine receptors. The postsynaptic excitatory H1Rs are primarily localized in cortical astrocytes, hippocampal, hypothalamic, and striatal neurons, whereas H2Rs are widely distributed in the basal ganglia, hippocampus, and amygdala. The presynaptic H3Rs are predominantly distributed in the cerebral cortex and subcortex, where they can function either as inhibitory auto‐receptors or heteroreceptors. The immune regulatory H4Rs are expressed in the spinal cord, hippocampus, and cerebral cortex in humans and rats.^572^, ^573^ These receptor subtypes differ markedly in their ligand binding affinity, with H3R and H4R displaying stronger binding than H1R and H2R.^574^ Based on the HA availability, microenvironment, and activation state, HA receptors can mediate inflammatory signals, hippocampal neurogenesis, and modulate anxiety‐related behavior, fear, and recognition memory.^575^, ^576^, ^577^, ^578^

*Post mortem* human studies and rodent models have shown loss of cortical and hypothalamic HA regulation in AD.^10^, ^579^, ^580^, ^581^, ^582^ There is profound loss of TMN HA neurons associated with AD‐specific phosphorylated tau aggregation.^579^ Subsequent analysis revealed a significant negative correlation between TMN neuron counts and clinical sleep measures, including sleep maintenance and proportion of time spent in N2 and rapid eye movement sleep stages, while a positive correlation was noted with wake after sleep onset.^10^ There is also a region‐specific loss of HA neurons in the TMN, with the most severe loss in the rostral TMN and the least in the caudal TMN. This region‐specific neuronal loss was accompanied by a significant downregulation of L‐histidine decarboxylase mRNA only in the medial TMN.^580^ Although TMN HA neurons declined significantly in AD, there is also a substantial increase in HA levels in the posterior hypothalamus in AD patients,^583^ in spite of overall cortical HA levels declining significantly.^584^ These region‐specific alterations within the HA system suggest a compensatory response to the substantial loss of TMN HA neurons. Furthermore, reduction in HA metabolic products in cerebrospinal fluid^585^ indicates altered HA levels and metabolism, which can potentially affect the brain's immune environment through microglial activation.^586^ Together, these studies demonstrate that the HA system is severely affected in AD, but the pattern, precise mechanism, and any sex differences of neuronal loss in AD are unknown.

### 9.1 Sex differences in the histaminergic system and interactions with sex hormones

The histaminergic system exhibits sex differences in tone, receptor expression patterns, and function, with compelling evidence that gonadal hormones play a key role in this relationship. TMN neurons express estrogen receptors ERα and ERβ in both sexes,^587^, ^588^ and a large percentage of HA‐synthesizing neurons in the TMN express nuclear ERα.^589^ Female rats have higher levels of HA in the brain.^590^ This is partially a result of the influence of androgens on HA methylation, which reduces HA levels in males.^591^ Female rats also display higher levels of cortical H1Rs and H2Rs than males.^592^, ^593^ Further, HA receptors are colocalized with estrogen receptors in the ventromedial nucleus of the hypothalamus.^594^ HA binding sites in rat cortex are denser in adult female rats compared to males and prepubertal animals of both sexes.^595^

Considerable evidence highlights the critical role of ovarian and androgenic steroids in sex‐specific expression of HA receptors and histaminergic function. HA levels and functional interactions with other neurotransmitter systems vary across the estrous cycle.^596^, ^597^ Prepubertal ovariectomized females show reduced HA binding sites at levels comparable to males, which is reversed by estradiol replacement.^595^ Ovariectomy also reduces H1R binding and expression of H1R mRNA in the hypothalamus,^594^, ^598^ both of which are reversed by estradiol. The effects of ovariectomy and estradiol appear to be mediated primarily by ERα.^594^ Evidence of the expression of progesterone and androgen receptors on TMN neurons is limited. However, adjacent hypothalamic regions, including the posterior hypothalamic nucleus, dorsomedial nucleus, ventromedial nucleus, infundibular nucleus, and bed nucleus of the stria terminalis, do express ERβs and influence histaminergic tone and function.^599^, ^600^ Progesterone specifically reduces the increased expression of H1Rs caused by estradiol, possibly through direct or indirect modulation of TMN neurons.^594^

Several recent studies have linked sex differences in the histaminergic system to sex differences in brain function and behavioral/cognitive functions. In female mice, exogenous HA increases striatal DA release via H3Rs in animals with high estrogen levels, whereas in males, HA reduces striatal dopamine release mediated by H2Rs.^597^ After neuroinflammatory responses or exposure to drugs that alter H3R function, female mice display greater regulation of HA release compared to males, which may be hormonally mediated and confer a neuroprotective advantage.^601^ Female mice also display greater sensitivity to the arousing effects of H1R antagonism.^602^ Longer retention of object memory has been observed in female rats, potentially linked to greater H1R and H2R expression in females.^592^ Similar dose‐dependent improvements in memory performance in both sexes were seen after acute administration of the H3R antagonist thioperamide.^592^ Acute chemogenetic activation of TMN HA neurons improved object recognition memory in female but not male mice.^603^ H1R and corticosterone bioperiodicity are tightly linked, with females showing lower H1R bioperiodicity and greater food consumption than males during dietary restriction.^593^, ^604^ Collectively, these observations illustrate a clear influence of sex on histaminergic function that could contribute to sex differences in how this neurotransmitter affects AD symptoms, pathology, and progression.

### 9.2 Sex differences in histaminergic systems in human and animal models of AD

Changes observed in AD and models of AD include receptor binding, expression, and composition of functional domains, all of which are highly predictive of cognitive deficits as observed in AD patients.^605^ Such findings support the potential for histaminergic drugs as effective AD treatments. However, while there are compelling sex differences in the HA system there is a paucity of data on how the alterations in histaminergic function in AD vary with sex, mainly owing to the inclusion of only one sex or the absence of rigorous assessments of sex differences in available studies.

A wealth of data has identified alterations in the histaminergic system in AD which are linked to cognitive decline and blood–brain barrier disruption.^606^ A few *post mortem* human studies have examined HA neuronal changes in AD patients, but the effect of sex in the progression of AD remains unknown. A substantial (57%) loss of TMN neurons occurs in AD patients.^580^ There are sex‐dependent changes in TMN neurons in AD, which, although not statistically significant, were substantially more pronounced in women (67%) than in men (34%) relative to controls. TMN L‐histidine decarboxylase mRNA expression levels showed non‐significant decreases in AD patients compared to controls. This decline parallels the changes in TMN neuron number, with women showing a steeper decline than men, specifically in AD patients relative to controls. Finally, sex‐dependent changes in HA projections across AD stages were assessed in the PFC. Both H3R and histamine‐N‐methyltransferase mRNA expression in the PFC was significantly increased in women at Braak stage V to VI compared to 0 to II. Additionally, in women, HA metabolism increased starting at Braak stage III to IV.^580^

To characterize the biological profiles of AD, various neurotransmitter metabolites have been studied in cerebrospinal fluid. Comparing tele‐methylhistamine levels in the aging brain to that of AD patients revealed a contrasting trend in HA metabolism. Whereas HA metabolism tends to increase in normal aging, it declines in AD patients. This reduction in cerebrospinal fluid levels of tele‐methylhistamine was sex dependent, with a greater decline in women AD patients than men AD patients.^585^ The age‐associated increase in tele‐methylhistamine also depends on sex,  with women having higher tele‐methylhistamine levels than men.^585^, ^607^ In addition, tele‐methylimidazoleacetic acid levels increased ≈ 30% in the cerebrospinal fluid during aging, and middle‐aged women also displayed higher levels than men.^607^ This contrast in cerebrospinal fluid levels of HA metabolites in normal aging and AD indicates a reduction in HA function, potentially due to TMN neuronal degeneration. Based on this work, modulating H3Rs with an inverse agonist may be able to normalize HA tone and improve sleep/wake dysfunction and cognition in AD patients.

To this end, rodent AD models have been leveraged to study the effects of pharmacological manipulation of the HA system. Improvements in cognitive and learning/memory deficits have been consistently observed after treatment with H3R antagonists or H3R inverse agonists in several transgenic models, including 5xFAD, APP_Tg2576_, B6.129‐Tg(APPSw)40Btla/J, THY‐Tau22, and BL/6‐Tg APP/PS1 mice.^608^, ^609^, ^610^, ^611^, ^612^, ^613^, ^614^ H3R antagonists and inverse agonists reduced pathological protein accumulation, normalized cellular signaling pathways, reduced neuroinflammation and gliosis, decreased oxidative stress markers, increased acetylcholine levels, enhanced protein clearance mechanisms, reduced dystrophic neurite pathology, and restored cortical slow‐wave coherence and frequency patterns. One study found that HA release was reduced in the amygdala of ApoE(−/−) mice.^615^ Just over half of these studies with pharmacological interventions were conducted exclusively in males, with only one study using females alone to examine the effects of ABT‐239 in TAPP mice for tau pathology.^609^ Three studies included both sexes but pooled or segregated the data for analysis without a statistical assessment of sex differences.^608^, ^613^, ^614^ However, beneficial effects appeared similar between males and females.^613^ Animal studies without pharmacological intervention that pooled sexes for analysis report either transient decreases in HA neuron number early in embryonic development that normalizes in adulthood or no difference in H3Rs expression in TASTPM mice, similar to observations in humans.^616^, ^617^ One study including sex differences observed that 3xTg‐AD mice displayed decreased L‐histidine expression in females and not males, without commensurate changes in HA levels.^618^

Male Sprague–Dawley rats display increased hypothalamic, midbrain, and cortical HA levels over the course of aging, which is increased under conditions of stress.^619^, ^620^ HA receptor preservation in transgenic models and advanced aging suggest that providing HA or an agonist may be a promising treatment strategy. H3R inverse agonists, such as ABT‐239 and SAR152954, have been effective in improving brain plasticity, learning, and memory in rodent models of fetal alcohol spectrum disorders, even into adulthood and well after the neurodevelopmental insult.^621^, ^622^, ^623^, ^624^ Future work should aim to conduct rigorous evaluations of sex differences in responses to histaminergic interventions to better understand underlying mechanisms of AD pathology and potential variation in response to treatment with histaminergic drugs.

### 9.3 Peripheral HA and AD pathology as mediated by sex

Sex differences in peripheral histaminergic function, particularly related to immune responses, potentially contribute to divergent AD susceptibility and response to histaminergic treatments. In female rats, mast cells display greater susceptibility to sex steroid modulation of HA release and perinatal androgens contribute to organizing lifelong sex differences in mast cell function, with males exhibiting reduced HA content and attenuated degranulation responses.^625^, ^626^, ^627^ Castration reduces peritoneal HA concentrations in males,^628^ and testosterone exerts selective anti‐inflammatory effects on mast cells sourced from women donors, but not men.^629^

H4Rs are predominantly expressed on immune cells and orchestrate mast cell recruitment and activation,^630^, ^631^ potentially underlying the relationship between peripheral sex differences and central neuroinflammatory responses. Emerging evidence suggests that peripheral histaminergic dysfunction influences AD pathogenesis through multiple mechanisms that could be sex dependent. Aβ peptides trigger mast cell degranulation through pannexin1‐dependent mechanisms,^632^ while mast cell proteases can generate Aβ N‐termini,^633^ creating positive feedback loops between AD pathology and neuroinflammation caused by the peripheral immune response.^634^ HA promotes astrocyte neuroprotection and microglial neurotoxicity,^635^, ^636^, ^637^ while simultaneously disrupting blood–brain barrier integrity and altering neurotransmitter function.^637^, ^638^, ^639^ Notably, females display greater vulnerability to HA‐mediated disruption of blood–brain barrier integrity.^606^, ^640^, ^641^ Mast cell deficiency improved cognition in an AD mouse model;^642^ however, the vast majority of studies have either excluded females or failed to assess sex differences statistically.^643^, ^644^ This represents a critical knowledge gap in understanding how the mechanisms discussed here may contribute to the increased prevalence of AD in females.

### 9.4 Future directions

Substantial sex differences in the HA system are well documented across neurobiological and behavioral domains that hold considerable relevance for understanding sex differences in AD. Higher HA receptor expression, enhancements in histaminergic modulation, decline in HA metabolism, and distinct patterns of HA–neurotransmitter interactions are evident in healthy women. Such differences have been linked to sex differences in cognition, learning and memory, and arousal in which the histaminergic system plays a key role. Despite robust evidence of sex differences in histaminergic function and general alterations to this system in AD, there is limited research examining the role of sex on histaminergic function in the context of AD.

There are several other important future steps that should be performed to appropriately understand sex as a modifier of histaminergic dysregulation in AD. For example, histaminergic anatomical organization and connectivity have not been characterized in AD. Although H3R antagonists and inverse agonists have shown considerable therapeutic promise in preclinical models of AD, the efficacy and potential synergy with other AD treatments have not been rigorously investigated or examined across sexes. Potential alterations in the peripheral histaminergic system, particularly mast cell dysfunction and disruptions in blood–brain barrier integrity, represent another understudied domain in which sex differences could contribute to AD pathogenesis. Finally, whether and how the histaminergic system compensates at different stages of AD progression and how such effects vary with sex need to be examined.

## 10 OREXIN/HYPOCRETIN

Neuropeptides OX‐A and ‐B (OX‐A/B, also known as hypocretin 1 and 2) are released by a specific group of neurons localized to a limited area in the tuberal region of the hypothalamus behind the paraventricular nucleus. The OX system plays a key role in regulating the transition between wakefulness and sleep, orchestrates thermoregulation and blood pressure, participates in motivation/reward and feeding, and interacts with the neuroendocrine system.^645^

Disruption or degeneration of the OX system leads to symptoms of narcolepsy in both humans and animal models.^646^ OX circuitry exhibits distinct synaptic architecture, characterized by excitatory glutamatergic input and a specialized glutamatergic receptor profile. This synaptic architecture supports the system's rapid activation in response to salient stimuli and underlies its role in regulating arousal, motivation, and survival‐relevant behaviors. Thus, the role of OX in regulating the sleep–wake cycle has been largely documented in narcolepsy type 1 (or in OX knock‐out animal models), which has expanded our understanding of the relevant role of this neurotransmitter in other brain functions and disease states.^646^, ^647^, ^648^

### 10.1 OX system and sex hormone interactions

After the discovery of two OX types, OX‐A and ‐B, and their receptors, OX1R and OX2R,^649^, ^650^ a separate report demonstrated that OX modulates luteinizing hormone secretion in an estrogen‐dependent manner.^651^ In particular, the effects of OX‐A and ‐B on luteinizing hormone secretion were investigated in ovariectomized rats with or without supplementation of ovarian hormones. Intracerebroventricular administration of OX‐A and ‐B rapidly stimulated luteinizing hormone secretion in a dose‐ and time‐dependent manner in ovariectomized rats pretreated with estradiol and progesterone. Ten minutes after injection, peak plasma luteinizing hormone levels were significantly higher in OX‐A‐treated rats compared to those treated with OX‐B. Conversely, in ovariectomized rats without steroid priming, both OX‐A and ‐B suppressed luteinizing hormone secretion. Thus, OXs are part of a group of hypothalamic signaling molecules that neurochemically link reproductive function with energy homeostasis.^651^

### 10.2 OX expression and function depend on sex hormones

In rodents, sex differences have been reported in OX peptide expression, function, and receptor distribution across the hypothalamus, pituitary, adrenal glands, and gonads. Female rats show higher levels of OX‐A and prepro‐OX mRNA in the lateral and posterior hypothalamus,^652^, ^653^ and greater OX1R expression in the hypothalamus compared to males. In contrast, males exhibit higher OX1R expression in the pituitary and OX2R in the adrenal glands compared to females.^654^ These expression patterns are hormonally regulated: gonadectomy increases pituitary OX1R in male rats (reversed by testosterone) and estradiol replacement in ovariectomized female rats produces a similar but stronger effect.^655^ Despite these dynamic effects, long‐term hormonal manipulations do not appear to significantly alter hypothalamic OX expression.^653^, ^655^ In contrast, rapid, cyclical changes in prepro‐OX and receptor expression have been observed in adult females, particularly during proestrus.^656^

OX terminals innervate gonadotropin‐releasing hormone neurons, which express OX1R and respond directly to OX by increasing gonadotropin‐releasing hormone release.^657^, ^658^, ^659^, ^660^ This supports a dual mechanism of luteinizing hormone regulation: indirectly via hypothalamic gonadotropin‐releasing hormone release and directly at the pituitary level,^660^ particularly in females. Estradiol modulates these interactions: OXs suppress luteinizing hormone in ovariectomized rats while enhancing luteinizing hormone release in estradiol‐treated animals.^651^, ^661^, ^662^ Estradiol has also been reported to suppress OX‐A activity directly,^663^ although most OX neurons do not co‐express estrogen receptor ERα or androgen receptor, suggesting that hormonal control may occur via afferent inputs.^664^

These sex‐specific expression patterns are developmentally programmed as proestrus‐associated upregulation of OX genes is abolished in neonatally androgenized females.^665^ Moreover, combined estradiol and progesterone treatment in perinatally demasculinized males mimicked the female‐like pattern, indicating that perinatal testosterone imprints the sex‐specific regulation of both OX and gonadotropin‐releasing hormone/luteinizing hormone systems, possibly through epigenetic mechanisms.

Beyond reproductive control, OXs are involved in other sex‐specific behaviors and pathologies, such as male sexual motivation,^664^, ^666^ sex‐specific obesity patterns,^667^, ^668^ and differential stress responses, depression susceptibility, and related disorders.^669^, ^670^ Nonetheless, much of what is known about OX function is based on male data, leaving the female phenotype underexplored.

### 10.3 Clinical evidence of sex differences in orexinergic systems

Understanding the complex interplay among female sex hormones, orexinergic signaling, sleep disruption, and tau pathology may yield novel insights into the sex‐specific progression of AD and support the development of tailored therapeutic strategies. However, clinical data on sex differences in human OX expression remain limited, with a notable lack of mechanistic studies across the lifespan. Most available evidence derives from research on sleep disorders (with narcolepsy being a prototypical model of OX deficiency^671^), including neuropsychiatric and neurodegenerative conditions.^672^

Such sleep and mental health disorders show marked sex differences in prevalence.^673^, ^674^, ^675^ Specifically, the higher incidence of insomnia, circadian sleep–wake rhythm disorders, internalizing mental health conditions, and AD in women, particularly during hormonal transitions such as puberty and menopause,^676^, ^677^ aligns with preclinical findings of enhanced OX expression and reactivity in females. Along with growing evidence suggesting a possible link to hyperactivation of the OX system,^678^, ^679^ insomnia is consistently more prevalent in women,^680^ whereas narcolepsy appears to be more common in men.^681^

Studies directly measuring OX‐A levels in humans, whether via *post mortem* brain analysis or cerebrospinal fluid sampling, have yielded inconsistent findings. Complicating matters, plasma OX‐A levels, despite being easier to access, do not correspond to the cerebrospinal fluid OX‐A concentrations.^682^ One *post mortem* study of patients with major depressive disorder found increased hypothalamic and cortical OX‐A immunoreactivity in women, but not in men, as well as an absence of diurnal OX‐A regulation in cerebrospinal fluid samples collected from patients,^670^ suggesting sex‐specific involvement of OX‐A in major depressive disorder‐related sleep and mood disruptions.

Another *post mortem* study revealed a loss of hypothalamic OX neurons and reduced cerebrospinal fluid OX‐A levels in late‐stage AD, with no significant sex effects reported.^683^ On the other hand, when AD patients were compared to cognitively normal controls, higher cerebrospinal fluid OX‐A levels were observed in women compared to men, regardless of diagnosis.^684^ A similar trend was reported across AD, dementia with Lewy bodies, and healthy controls, where differences in cerebrospinal fluid OX‐A levels were primarily driven by sex. Specifically, women exhibit higher and lower OX‐A cerebrospinal fluid levels in AD and in dementia with Lewy bodies, respectively.^685^ Notably, this study suggests that sex‐specific dysfunction of the OX system may be disease dependent. Still, these interpretations should be considered with the fact that anti‐dementia treatments prescribed to patients may have affected sleep and OX neurotransmission. Conversely, some have found no sex differences in cerebrospinal fluid OX‐A levels across diagnostic groups, although OX‐A cerebrospinal fluid levels were higher in AD groups.^686^ Likewise, higher cerebrospinal fluid OX‐A concentrations have been reported in patients with moderate‐to‐severe AD versus controls, but not in patients with mild AD, and no sex‐related differences were found.^687^ However, a subsequent investigation revealed higher cerebrospinal fluid levels of OX‐A in patients with MCI compared to controls, again with no reported sex differences.^688^

A more recent multicenter study involving patients with a range of neurocognitive disorders (including mild to severe AD, behavioral variant frontotemporal dementia, non‐fluent primary aphasia, and idiopathic normal pressure hydrocephalus) and elderly controls, reported higher cerebrospinal fluid OX‐A levels across most disorder groups compared to controls.^689^ While no sex differences were detected, men in the control group exhibited higher cerebrospinal fluid OX‐A levels than women. This finding diverges from preclinical models but may reflect age‐related hormonal shifts that were not accounted for, given the mean age of > 60 years in the human control group which may diminish estradiol‐mediated modulation of OX‐A signaling.

### 10.4 Considering sex in the therapeutic potential of OXs in AD

Interest in OX as a therapeutic target for neurodegenerative diseases, especially AD, is growing.^673^ Enhanced OX activation in females—linked to greater stress vulnerability—may contribute to sex‐specific susceptibility to AD.^669^ Moreover, estrogen receptors have been localized in neurons containing neurofibrillary tangles,^690^, ^691^ although hormone therapies have shown limited benefit in clinical trials.^692^ Given the role of estradiol in sleep regulation^693^, ^694^, ^695^, ^696^ and the central importance of OX in the sleep–wake cycle, the intersection among estrogens, sleep, and OX signaling in AD pathophysiology, particularly in women, deserves greater attention.

Two recent studies using rTg4510 tauopathy mice highlight key sex differences in OX responses to pharmacological intervention.^697^, ^698^ Acute OX2R antagonism improves non–rapid eye movement sleep and reduces hyperarousal in male mice, but these effects are transient or absent in females despite equivalent drug exposure. Chronic treatment in males also reduces hyperphosphorylated tau levels and improves glymphatic clearance, effects that were similarly absent in females. In animal model studies, suvorexant, a dual OX receptor antagonist, increases rapid eye movement sleep in both sexes but fails to resolve hyperarousal, whereas zolpidem, a positive allosteric modulator of the GABA_A_ receptor, shows limited impact.^697^, ^698^ Parallel evidence in humans shows that degeneration of subcortical wake‑promoting neurons correlates strongly with disrupted sleep phenotypes in AD and progressive supranuclear palsy patients, suggesting a mechanistic substrate for arousal dysregulation in tauopathies.^10^ These results suggest that females may have intrinsic resistance to OX2R‐targeted therapies, potentially due to altered receptor function or divergent tau‐related circuitry. In agreement with this possibility, chronic administration of lemborexant, another dual OX receptor antagonist, improves sleep–wake cycle, reduces reactive microgliosis, and mitigates brain atrophy in male tauopathy mice.^699^ This further underscores the therapeutic relevance of modulating OX signaling in AD models and its potential sex‐dependent effects.

Finally, with increasing clinical interest in dual OX receptor antagonists for insomnia disorder^700^ and their potential utility in AD,^701^ preliminary findings suggest good tolerability in both sexes. In a large trial, suvorexant showed comparable efficacy in women and men with insomnia disorder, though adverse events were more frequently reported in women.^702^ In a separate placebo‐controlled study in patients with mild‐to‐moderate AD and comorbid insomnia disorder, suvorexant significantly improved objective sleep measures without sex‐related differences in efficacy.^703^ However, neither trial accounted for hormonal status or menopause/menopausal transition. Furthermore, dual OX receptor antagonists, by targeting both OX1R and OX2R, may obscure potential sex differences in receptor‐specific OX regulation and pharmacodynamics.^682^

Overall, human evidence remains inconclusive. Existing studies are predominantly cross‐sectional, often with small sample sizes that do not incorporate hormonal profiling or stratified analyses. Longitudinal research is required to clarify whether sex modulates OX signaling across aging and neurodegeneration, and whether OX‐targeting therapies warrant sex‐specific dosing or timing.

### 10.5 Future directions

OX plays a major role in sleep–wake cycles, disruption of which is one of the most common occurrences throughout the course of AD. Although human studies have shown inconsistent results, it is clear that the OX system is sensitive to sex hormones and pharmacological effects of OX interventions are sex dependent. Thus, future research should establish longitudinal cohorts with serial cerebrospinal fluid OX level assessments, alongside core biomarkers of neurodegeneration (Aβ/tau), objective sleep–wake metrics (e.g., actigraphy, polysomnography), and cognitive evaluations, with adequate power for sex and hormonal subgroup analyses. Therapeutic investigations should conduct sex‐balanced randomized controlled trials of dual OX receptor antagonists, especially in older adults with cognitive complaints or early‐stage AD, including pharmacokinetic/pharmacodynamic profiling and biomarker or imaging endpoints. Measuring the potential differences in OX neurotransmission during the various physiological phases of men's and women's lives, in light of the continuous modifications of sex hormone levels, is also warranted. Finally, further emphasis should be placed on exploring the marked sex differences in the effectiveness of OX2R antagonism, specifically focusing on the sex‐dependent interactions between tau pathology and the OX system. These insights may inform the development of hypnotic treatments for tauopathy‐related neurodegenerative diseases. Notably, enhancing sleep and reducing hyperarousal after disease onset has been shown to restore cognitive function in male tau transgenic mice, even without reducing phosphorylated tau levels.^39^ Although the mechanisms remain unclear, these findings reinforce the therapeutic potential of sleep modulation against neurodegeneration via the OX system.

## 11 DISCUSSION

NSSs are increasingly being recognized as key players in the early stages of AD, and their dysfunction persists throughout disease progression. These systems are responsible for regulating mood, stress, social behaviors, sleep, and cognition, accumulating early, disease‐specific pathology that ultimately leads to neuronal degeneration. NSSs display varying degrees of sex differences in structure, function, and response to sex hormones. Such sex‐specific differences are generally less well explored in humans and AD but could contribute to the well‐documented sex disparities in incidence, symptom progression, and neuropathology.

In this review, we summarize evidence of sex differences across nine NSSs. Variability in reported sex differences across human and model systems stems, in part, from methodological gaps, including the lack of sex‐disaggregated data, underpowered samples, and failure to account for hormonal status across the lifespan. This has resulted in mixed findings, with some failing to examine sex altogether. Without consistent, sex‐informed experimental designs, the field risks overlooking critical mechanisms that shape AD vulnerability, particularly at the earliest stages of disease when mitigation strategies would be most effective. Moving forward, research on NSSs, and dementias more broadly, must systematically include sex as a biological variable, incorporate hormonal context, and report findings by sex in both human studies and animal models to resolve discrepancies in the literature. Doing so will strengthen and clarify our understanding of NSSs involvement in dementia, supporting the development of more targeted and effective interventions for both women and men. Some considerations for future studies are to determine the extent to which each one of these NSSs contribute to overlapping symptoms (e.g., AVP and OXT in social deficits, LC–NE and DRN 5‐HT in depression), the amount of cross‐talk occurring between each system (e.g., LC–NE and CRH), and the extent to which non‐primary neurotransmitters (e.g., DA release from DRN subpopulations, galanin release from the LC) play a role in disease processes.

## CONFLICT OF INTEREST STATEMENT

Oihane Uriarte Huarte is a full‐time employee of the Alzheimer's Association. Claudio Liguori has served as a consultant and received research support from Idorsia and EISAI. The following authors serve or have served as executive committee members of the Neuromodulatory Subcortical Systems Professional Interest Area of ISTAART: Martin J. Dahl, Alexander J. Ehrenberg, Neus Falgàs, Lea Tenenholz Grinberg, Heidi I. L. Jacobs, Elouise A. Koops, Sabrina Lenzoni, Gowoon Son, and Michael A. Kelberman. The following authors serve or have served as executive committee members of the Sex and Gender Differences in Alzheimer's Disease Professional Interest Area of ISTAART: Rosaria J. Rae, Michael E. Belloy, Rachel Buckley, Jessica Z. K. Caldwell, Gillian Einstein, Megan C. Fitzhugh, Clara Gallay, Judy Pa, Mabel Seto, Shabana M. Shaik, and Shireen Sindi. All other authors have no conflicts of interest to declare. Rosaria J. Rae began employment at NeuroNexus while this manuscript was under review and during the revision process. Author disclosures are available in the supporting information.

## Supporting information



Supporting Information

## References

[1] Ehrenberg AJ , Suemoto CK , de P França Resende E , et al. Neuropathologic correlates of psychiatric symptoms in Alzheimer's disease. J Alzheimers Dis. 2018;66:115‐126. doi:10.3233/JAD-180688 30223398 PMC6381997

[2] Johansson M , Stomrud E , Insel PS , et al. Mild behavioral impairment and its relation to tau pathology in preclinical Alzheimer's disease. Transl Psychiatry. 2021;11:1‐8. doi:10.1038/s41398-021-01206-z 33500386 PMC7838407

[3] Jost BC , Grossberg GT . The evolution of psychiatric symptoms in Alzheimer's disease: a natural history study. J Am Geriatr Soc. 1996;44:1078‐1081. doi:10.1111/j.1532-5415.1996.tb02942.x 8790235

[4] Ehrenberg AJ , Kelberman MA , Liu KY , et al. Priorities for research on neuromodulatory subcortical systems in Alzheimer's disease: position paper from the NSS PIA of ISTAART. Alzheimer's Dementia. 2023;19:2182‐2196. doi:10.1002/alz.12937 PMC10182252 36642985

[5] Braak H , Thal DR , Ghebremedhin E , Del Tredici K . Stages of the Pathologic Process in Alzheimer Disease: age Categories From 1 to 100 Years. Journal of Neuropathology & Experimental Neurology. 2011;70:960‐969. doi:10.1097/NEN.0b013e318232a379 22002422

[6] Gilvesy A , Husen E , Magloczky Z , et al. Spatiotemporal characterization of cellular tau pathology in the human locus coeruleus‐pericoerulear complex by three‐dimensional imaging. Acta Neuropathol. 2022;144:651‐676. doi:10.1007/s00401-022-02477-6 36040521 PMC9468059

[7] Son G , Mladinov M , Pereira FL , et al. Anterior hypothalamic pathology in Alzheimer's disease: a human postmortem study using spatial in‐situ proteomics. Alzheimer's & Dementia. 2022;18:e060951. doi:10.1002/alz.060951

[8] Yoo HS , Kim H‐K , Lee J‐H , et al. Association of basal forebrain volume with amyloid, tau, and cognition in Alzheimer's disease. J Alzheimers Dis. 2024;99:145‐159. doi:10.3233/JAD-230975 38640150

[9] Cantero JL , Atienza M , Lage C , et al. Atrophy of basal forebrain initiates with tau pathology in individuals at risk for Alzheimer's disease. Cereb Cortex. 2020;30:2083‐2098. doi:10.1093/cercor/bhz224 31799623 PMC8493660

[10] Oh JY , Walsh CM , Ranasinghe K , et al. Subcortical neuronal correlates of sleep in neurodegenerative diseases. JAMA Neurol. 2022;79:498‐508. doi:10.1001/jamaneurol.2022.0429 35377391 PMC8981071

[11] Ehrenberg AJ , Nguy AK , Theofilas P , et al. Quantifying the accretion of hyperphosphorylated tau in the locus coeruleus and dorsal raphe nucleus: the pathological building blocks of early Alzheimer's disease. Neuropathol Appl Neurobiol. 2017;43:393‐408. doi:10.1111/nan.12387 28117917 PMC5642282

[12] Grinberg LT , Rüb U , Ferretti REL , et al. The dorsal raphe nucleus shows phospho‐tau neurofibrillary changes before the transentorhinal region in Alzheimer's disease. A precocious onset?. Neuropathol Appl Neurobiol. 2009;35:406‐416. doi:10.1111/j.1365-2990.2009.00997.x 19508444

[13] Chen CP , Eastwood SL , Hope T , McDonald B , Francis PT , Esiri MM . Immunocytochemical study of the dorsal and median raphe nuclei in patients with Alzheimer's disease prospectively assessed for behavioural changes. Neuropathol Appl Neurobiol. 2000;26:347‐355. doi:10.1046/j.1365-2990.2000.00254.x 10931368

[14] Caldwell JZK , Berg J‐L , Cummings JL , Banks SJ , Alzheimer's Disease Neuroimaging Initiative . Moderating effects of sex on the impact of diagnosis and amyloid positivity on verbal memory and hippocampal volume. Alzheimers Res Ther. 2017;9:72. doi:10.1186/s13195-017-0300-8 28899422 PMC5596932

[15] Mazure CM , Swendsen J . Sex differences in Alzheimer's disease and other dementias. Lancet Neurol. 2016;15:451‐452. doi:10.1016/S1474-4422(16)00067-3 26987699 PMC4864429

[16] Aggarwal NT , Mielke MM . Sex differences in Alzheimer's disease. Neurol Clin. 2023;41:343‐358. doi:10.1016/j.ncl.2023.01.001 37030962 PMC10321561

[17] Beam CR , Kaneshiro C , Jang JY , Reynolds CA , Pedersen NL , Gatz M . Differences between women and men in incidence rates of dementia and Alzheimer's disease. J Alzheimers Dis. 2018;64:1077‐1083. doi:10.3233/JAD-180141 30010124 PMC6226313

[18] Subramaniapillai S , Almey A , Natasha Rajah M , Einstein G . Sex and gender differences in cognitive and brain reserve: implications for Alzheimer's disease in women. Front Neuroendocrinol. 2021;60:100879. doi:10.1016/j.yfrne.2020.100879 33137359

[19] Mielke MM . Sex and gender differences in Alzheimer's disease dementia. Psychiatr Times. 2018;35:14‐17. 30820070 PMC6390276

[20] Nichols E , Steinmetz JD , Vollset SE , et al. Estimation of the global prevalence of dementia in 2019 and forecasted prevalence in 2050: an analysis for the Global Burden of Disease Study 2019. Lancet Public Health. 2022;7:e105‐25. doi:10.1016/S2468-2667(21)00249-8 34998485 PMC8810394

[21] O'Neal MA . Women and the risk of Alzheimer's disease. Front Glob Women's Health. 2024;4:1324522. doi:10.3389/fgwh.2023.1324522 38250748 PMC10796575

[22] Buckley RF , Mormino EC , Amariglio RE , et al. Sex, amyloid, and APOE ε4 and risk of cognitive decline in preclinical Alzheimer's disease: findings from three well‐characterized cohorts. Alzheimers Dement. 2018;14:1193‐1203. doi:10.1016/j.jalz.2018.04.010 29803541 PMC6131023

[23] Ballinger EC , Ananth M , Talmage DA , Role LW . Basal forebrain cholinergic circuits and signaling in cognition and cognitive decline. Neuron. 2016;91:1199‐1218. doi:10.1016/j.neuron.2016.09.006 27657448 PMC5036520

[24] Hasselmo ME , Sarter M . Modes and models of forebrain cholinergic neuromodulation of cognition. Neuropsychopharmacol. 2011;36:52‐73. doi:10.1038/npp.2010.104 PMC2992803 20668433

[25] Bohnen NI , Grothe MJ , Ray NJ , Müller MLTM , Teipel SJ . Recent advances in cholinergic imaging and cognitive decline‐Revisiting the cholinergic hypothesis of dementia. Curr Geriatr Rep. 2018;7:1‐11. doi:10.1007/s13670-018-0234-4 29503795 PMC5831510

[26] Teipel S , Heinsen H , Amaro E , et al. Cholinergic basal forebrain atrophy predicts amyloid burden in Alzheimer's disease. Neurobiol Aging. 2014;35:482‐491. doi:10.1016/j.neurobiolaging.2013.09.029 24176625 PMC4120959

[27] Kilimann I , Grothe M , Heinsen H , et al. Subregional basal forebrain atrophy in Alzheimer's disease: a multicenter study. J Alzheimers Dis. 2014;40:687‐700. doi:10.3233/JAD-132345 24503619 PMC4120953

[28] Fernández‐Cabello S , Kronbichler M , Van Dijk KRA , et al. Basal forebrain volume reliably predicts the cortical spread of Alzheimer's degeneration. Brain. 2020;143:993‐1009. doi:10.1093/brain/awaa012 32203580 PMC7092749

[29] Kerbler GM , Fripp J , Rowe CC , et al. Basal forebrain atrophy correlates with amyloid β burden in Alzheimer's disease. Neuroimage Clin. 2015;7:105‐113. doi:10.1016/j.nicl.2014.11.015 25610772 PMC4299972

[30] Whitehouse PJ , Price DL , Struble RG , Clark AW , Coyle JT , Delon MR . Alzheimer's disease and senile dementia: loss of neurons in the basal forebrain. Science. 1982;215:1237‐1239. doi:10.1126/science.7058341 7058341

[31] Mieling M , Meier H , Bunzeck N . Structural degeneration of the nucleus basalis of Meynert in mild cognitive impairment and Alzheimer's disease – Evidence from an MRI‐based meta‐analysis. Neurosci Biobehav Rev. 2023;154:105393. doi:10.1016/j.neubiorev.2023.105393 37717861

[32] Aghourian M , Legault‐Denis C , Soucy J‐P , et al. Quantification of brain cholinergic denervation in Alzheimer's disease using PET imaging with [18F]‐FEOBV. Mol Psychiatry. 2017;22:1531‐1538. doi:10.1038/mp.2017.183 28894304

[33] Dumas JA , Newhouse PA . The cholinergic hypothesis of cognitive aging revisited again: cholinergic functional compensation. Pharmacol Biochem Behav. 2011;99:254‐261. doi:10.1016/j.pbb.2011.02.022 21382398 PMC3114182

[34] Parikh V , Sarter M . Cholinergic mediation of attention: contributions of phasic and tonic increases in prefrontal cholinergic activity. Ann N Y Acad Sci. 2008;1129:225‐235. doi:10.1196/annals.1417.021 18591483

[35] Sarter M , Givens B , Bruno JP . The cognitive neuroscience of sustained attention: where top‐down meets bottom‐up. Brain Res Rev. 2001;35:146‐160. doi:10.1016/S0165-0173(01)00044-3 11336780

[36] Parvizi J , Van Hoesen GW , Damasio A . The selective vulnerability of brainstem nuclei to Alzheimer's disease. Ann Neurol. 2001;49:53‐66. doi:10.1002/1531-8249(200101)49:1<53::aid-ana30>3.0.co;2-q 11198297

[37] Okkels N , Horsager J , Labrador‐Espinosa M , et al. Severe cholinergic terminal loss in newly diagnosed dementia with Lewy bodies. Brain. 2023;146:3690‐3704. doi:10.1093/brain/awad192 37279796

[38] Newhouse P , Dumas J . Estrogen‐cholinergic interactions: implications for cognitive aging. Horm Behav. 2015;74:173‐185. doi:10.1016/j.yhbeh.2015.06.022 26187712 PMC4573353

[39] Russell JK , Jones CK , Newhouse PA . The role of estrogen in brain and cognitive aging. Neurotherapeutics. 2019;16:649‐665. doi:10.1007/s13311-019-00766-9 31364065 PMC6694379

[40] Gibbs RB . Estrogen therapy and cognition: a review of the cholinergic hypothesis. Endocr Rev. 2010;31:224‐253. doi:10.1210/er.2009-0036 20019127 PMC2852210

[41] Gibbs RB . Impairment of basal forebrain cholinergic neurons associated with aging and long‐term loss of ovarian function. Exp Neurol. 1998;151:289‐302. doi:10.1006/exnr.1998.6789 9628764

[42] Veng LM , Granholm A‐C , Rose GM . Age‐related sex differences in spatial learning and basal forebrain cholinergic neurons in F344 rats. Physiol Behav. 2003;80:27‐36. doi:10.1016/s0031-9384(03)00219-1 14568305

[43] Yamamoto H , Kitawaki J , Kikuchi N , et al. Effects of estrogens on cholinergic neurons in the rat basal nucleus. J Steroid Biochem Mol Biol. 2007;107:70‐79. doi:10.1016/j.jsbmb.2007.03.035 17651965

[44] Batallán Burrowes AA , Olajide OJ , Iasenza IA , Shams WM , Carter F , Chapman CA . Ovariectomy reduces cholinergic modulation of excitatory synaptic transmission in the rat entorhinal cortex. PLoS One. 2022;17:e0271131. doi:10.1371/journal.pone.0271131 35939438 PMC9359571

[45] Shi Y , Cui D , Sun F , et al. Exploring sexual dimorphism in basal forebrain volume changes during aging and neurodegenerative diseases. iScience. 2024;27:109041. doi:10.1016/j.isci.2024.109041 38361626 PMC10867643

[46] Ishunina TA , Fisser B , Swaab DF . Sex differences in androgen receptor immunoreactivity in basal forebrain nuclei of elderly and Alzheimer patients. Exp Neurol. 2002;176:122‐132. doi:10.1006/exnr.2002.7907 12093089

[47] Williamson J , Yabluchanskiy A , Mukli P , et al. Sex differences in brain functional connectivity of hippocampus in mild cognitive impairment. Front Aging Neurosci. 2022;14:959394. doi:10.3389/fnagi.2022.959394 36034134 PMC9399646

[48] Gibbs RB . Effects of gonadal hormone replacement on measures of basal forebrain cholinergic function. Neuroscience. 2000;101:931‐938. doi:10.1016/s0306-4522(00)00433-4 11113342

[49] Gibbs RB , Wu D , Hersh LB , Pfaff DW . Effects of estrogen replacement on the relative levels of choline acetyltransferase, trkA, and nerve growth factor messenger RNAs in the basal forebrain and hippocampal formation of adult rats. Exp Neurol. 1994;129:70‐80. doi:10.1006/exnr.1994.1148 7925844

[50] Tinkler GP , Voytko ML . Estrogen modulates cognitive and cholinergic processes in surgically menopausal monkeys. Prog Neuropsychopharmacol Biol Psychiatry. 2005;29:423‐431. doi:10.1016/j.pnpbp.2004.12.016 15795051

[51] Mennenga SE , Gerson JE , Koebele SV , Kingston ML , et al. Understanding the cognitive impact of the contraceptive estrogen Ethinyl Estradiol: tonic and cyclic administration impairs memory, and performance correlates with basal forebrain cholinergic system integrity. Psychoneuroendocrinology. 2015;54:1‐13. doi:10.1016/j.psyneuen.2015.01.002 25679306 PMC4433884

[52] Bartholomeusz CF , Wesnes KA , Kulkarni J , Vitetta L , Croft RJ , Nathan PJ . Estradiol treatment and its interaction with the cholinergic system: effects on cognitive function in healthy young women. Horm Behav. 2008;54:684‐693. doi:10.1016/j.yhbeh.2008.07.007 18706905

[53] Smith YR , Bowen L , Love TM , et al. Early initiation of hormone therapy in menopausal women is associated with increased hippocampal and posterior cingulate cholinergic activity. J Clin Endocrinol Metab. 2011;96:E1761‐1770. doi:10.1210/jc.2011-0351 21865354 PMC3205894

[54] Norbury R , Travis MJ , Erlandsson K , Waddington W , Ell PJ , Murphy DGM . Estrogen therapy and brain muscarinic receptor density in healthy females: a SPET study. Horm Behav. 2007;51:249‐257. doi:10.1016/j.yhbeh.2006.10.007 17173920

[55] Zandi PP , Carlson MC , Plassman BL , et al. Hormone replacement therapy and incidence of Alzheimer disease in older women: the Cache County Study. JAMA. 2002;288:2123‐2129. doi:10.1001/jama.288.17.2123 12413371

[56] Saleh RNM , Hornberger M , Ritchie CW , Minihane AM . Hormone replacement therapy is associated with improved cognition and larger brain volumes in at‐risk APOE4 women: results from the European Prevention of Alzheimer's Disease (EPAD) cohort. Alzheimers Res Ther. 2023;15:10. doi:10.1186/s13195-022-01121-5 36624497 PMC9830747

[57] Calvo N , McFall GP , Ramana S , et al. Associated risk and resilience factors of Alzheimer's disease in women with early bilateral oophorectomy: data from the UK Biobank. Journal of Alzheimer's Disease. 2024;102:119‐128. doi:10.3233/JAD-240646 39497303

[58] Puri TA , Gravelsins LL , Alexander MW , et al. Association between menopause age and estradiol‐based hormone therapy with cognitive performance in cognitively normal women in the CLSA. Neurology. 2025;105:e213995. doi:10.1212/WNL.0000000000213995 40865026 PMC12380479

[59] Gleason CE , Dowling NM , Kara F , et al. Long‐term cognitive effects of menopausal hormone therapy: findings from the KEEPS continuation study. PLOS Med. 2024;21:e1004435. doi:10.1371/journal.pmed.1004435 39570992 PMC11581397

[60] Baakman AC , Alvarez‐Jimenez R , Rissmann R , et al. An anti‐nicotinic cognitive challenge model using mecamylamine in comparison with the anti‐muscarinic cognitive challenge using scopolamine. Br J Clin Pharmacol. 2017;83:1676‐1687. doi:10.1111/bcp.13268 28217868 PMC5510063

[61] Snyder PJ , Bednar MM , Cromer JR , Maruff P . Reversal of scopolamine‐induced deficits with a single dose of donepezil, an acetylcholinesterase inhibitor. Alzheimers Dement. 2005;1:126‐135. doi:10.1016/j.jalz.2005.09.004 19595845

[62] Snyder PJ , Lim YY , Schindler R , et al. Microdosing of scopolamine as a “cognitive stress test”: rationale and test of a very low dose in an at‐risk cohort of older adults. Alzheimers Dement. 2014;10:262‐267. doi:10.1016/j.jalz.2014.01.009 24698030

[63] Drachman DA , Noffsinger D , Sahakian BJ , Kurdziel S , Fleming P . Aging, memory, and the cholinergic system: a study of dichotic listening. Neurobiol Aging. 1980;1:39‐43. doi:10.1016/0197-4580(80)90022-6 7266733

[64] Gibbs RB , Burke AM , Johnson DA . Estrogen replacement attenuates effects of scopolamine and lorazepam on memory acquisition and retention. Horm Behav. 1998;34:112‐125. doi:10.1006/hbeh.1998.1452 9799622

[65] Dumas J , Hancur‐Bucci C , Naylor M , Sites C , Newhouse P . Estrogen treatment effects on anticholinergic‐induced cognitive dysfunction in normal postmenopausal women. Neuropsychopharmacol. 2006;31:2065‐2078. doi:10.1038/sj.npp.1301042 16482084

[66] Dumas JA , Kutz AM , Naylor MR , Johnson JV , Newhouse PA . Estradiol treatment altered anticholinergic‐related brain activation during working memory in postmenopausal women. Neuroimage. 2012;60:1394‐1403. doi:10.1016/j.neuroimage.2012.01.043 22266175 PMC3303937

[67] Dumas J , Hancur‐Bucci C , Naylor M , Sites C , Newhouse P . Estradiol interacts with the cholinergic system to affect verbal memory in postmenopausal women: evidence for the critical period hypothesis. Horm Behav. 2008;53:159‐169. doi:10.1016/j.yhbeh.2007.09.011 17964576 PMC2435492

[68] Rapp PR , Morrison JH , Roberts JA . Cyclic estrogen replacement improves cognitive function in aged ovariectomized rhesus monkeys. J Neurosci. 2003;23:5708‐5714. doi:10.1523/JNEUROSCI.23-13-05708.2003 12843274 PMC6741262

[69] Gibbs RB . Long‐term treatment with estrogen and progesterone enhances acquisition of a spatial memory task by ovariectomized aged rats. Neurobiol Aging. 2000;21:107‐116. doi:10.1016/s0197-4580(00)00103-2 10794855

[70] Maki PM . Critical window hypothesis of hormone therapy and cognition: a scientific update on clinical studies. Menopause. 2013;20:695‐709. doi:10.1097/GME.0b013e3182960cf8 23715379 PMC3780981

[71] Gibbs RB , Mauk R , Nelson D , Johnson DA . Donepezil treatment restores the ability of estradiol to enhance cognitive performance in aged rats: evidence for the cholinergic basis of the critical period hypothesis. Horm Behav. 2009;56:73‐83. doi:10.1016/j.yhbeh.2009.03.003 19303882 PMC2737520

[72] Whitmer RA , Quesenberry CP , Zhou J , Yaffe K . Timing of hormone therapy and dementia: the critical window theory revisited. Ann Neurol. 2011;69:163‐169. doi:10.1002/ana.22239 21280086 PMC3058824

[73] Jamshed N , Ozair FF , Aggarwal P , Ekka M . Alzheimer disease in post‐menopausal women: intervene in the critical window period. J Midlife Health. 2014;5:38‐40. doi:10.4103/0976-7800.127791 24672205 PMC3955045

[74] Kantarci K , Tosakulwong N , Lesnick TG , et al. Cardiometabolic outcomes in Kronos Early Estrogen Prevention Study continuation: 14‐year follow‐up of a hormone therapy trial. Menopause. 2024;31:10‐17. doi:10.1097/GME.0000000000002278 37989141 PMC10756493

[75] Jessen F . Subjective and objective cognitive decline at the pre‐dementia stage of Alzheimer's disease. Eur Arch Psychiatry Clin Neurosci. 2014;264(Suppl 1):S3‐S7. doi:10.1007/s00406-014-0539-z 25238934

[76] Jessen F , Wiese B , Bachmann C , et al. Prediction of dementia by subjective memory impairment: effects of severity and temporal association with cognitive impairment. Arch Gen Psychiatry. 2010;67:414‐422. doi:10.1001/archgenpsychiatry.2010.30 20368517

[77] Dumas JA , Kutz AM , McDonald BC , et al. Increased working memory‐related brain activity in middle‐aged women with cognitive complaints. Neurobiol Aging. 2013;34:1145‐1147. doi:10.1016/j.neurobiolaging.2012.08.013 23036586 PMC3540200

[78] Conley AC , Albert KM , McDonald BC , Saykin AJ , Dumas JA , Newhouse PA . Estradiol treatment in young postmenopausal women with self‐reported cognitive complaints: effects on cholinergic‐mediated cognitive performance. Hum Psychopharmacol. 2022;37:e2838. doi:10.1002/hup.2838 35212023 PMC9399322

[79] Crandall CJ , Mehta JM , Manson JE . Management of menopausal symptoms: a review. JAMA. 2023;329:405‐420. doi:10.1001/jama.2022.24140 36749328

[80] Stuenkel CA , Davis SR , Gompel A , et al. Treatment of symptoms of the menopause: an endocrine society clinical practice guideline. J Clin Endocrinol Metab. 2015;100:3975‐4011. doi:10.1210/jc.2015-2236 26444994

[81] Espeland MA , Rapp SR , Shumaker SA , et al. Conjugated equine estrogens and global cognitive function in postmenopausal women: women's Health Initiative Memory Study. JAMA. 2004;291:2959‐2968. doi:10.1001/jama.291.24.2959 15213207

[82] Shumaker SA , Legault C , Kuller L , et al. Conjugated equine estrogens and incidence of probable dementia and mild cognitive impairment in postmenopausal women: women's Health Initiative Memory Study. JAMA. 2004;291:2947‐2958. doi:10.1001/jama.291.24.2947 15213206

[83] Espeland MA , Shumaker SA , Leng I , et al. Long‐term effects on cognitive function of postmenopausal hormone therapy prescribed to women aged 50 to 55 years. JAMA Intern Med. 2013;173:1429‐1436. doi:10.1001/jamainternmed.2013.7727 23797469 PMC3844547

[84] Henderson VW . Progesterone and human cognition. Climacteric. 2018;21:333‐340. doi:10.1080/13697137.2018.1476484 29852783 PMC6309195

[85] Vongher JM , Frye CA . Progesterone in conjunction with estradiol has neuroprotective effects in an animal model of neurodegeneration. Pharmacol Biochem Behav. 1999;64:777‐785. doi:10.1016/S0091-3057(99)00140-9 10593201

[86] Sherwin BB , Grigorova M . Differential effects of estrogen and micronized progesterone or medroxyprogesterone acetate on cognition in postmenopausal women. Fertil Steril. 2011;96:399‐403. doi:10.1016/j.fertnstert.2011.05.079 21703613 PMC4838455

[87] Maki PM , Rubin LH , Fornelli D , et al. Effects of botanicals and combined hormone therapy on cognition in postmenopausal women. Menopause. 2009;16:1167‐1177. doi:10.1097/gme.0b013e3181ace484 19590458 PMC2783198

[88] Conley AC , Vega JN , Johnson JV , Dumas JA , Newhouse PA . Effect of estradiol with or without micronized progesterone on cholinergic‐related cognitive performance in postmenopausal women. Front Neurosci. 2024;18:1428675. doi:10.3389/fnins.2024.1428675 39184322 PMC11342399

[89] Andén N‐E , Carlsson A , Dahlström A , Fuxe K , Hillarp N‐Å , Larsson K . Demonstration and mapping out of nigro‐neostriatal dopamine neurons. Life Sciences. 1964;3:523‐530. doi:10.1016/0024-3205(64)90161-4 14187491

[90] Swanson LW . The projections of the ventral tegmental area and adjacent regions: a combined fluorescent retrograde tracer and immunofluorescence study in the rat. Brain Res Bull. 1982;9:321‐353. doi:10.1016/0361-9230(82)90145-9 6816390

[91] Haber SN . The place of dopamine in the cortico‐basal ganglia circuit. Neuroscience. 2014;282:248‐257. doi:10.1016/j.neuroscience.2014.10.008 25445194 PMC5484174

[92] Cools R , Arnsten AFT . Neuromodulation of prefrontal cortex cognitive function in primates: the powerful roles of monoamines and acetylcholine. Neuropsychopharmacol. 2022;47:309‐328. doi:10.1038/s41386-021-01100-8 PMC8617291 34312496

[93] Bech P , Crochet S , Dard R , et al. Striatal dopamine signals and reward learning. Function. 2023;4:zqad056. doi:10.1093/function/zqad056 37841525 PMC10572094

[94] Dahl MJ , Kulesza A , Werkle‐Bergner M , Mather M . Declining locus coeruleus–dopaminergic and noradrenergic modulation of long‐term memory in aging and Alzheimer's disease. Neurosci Biobehav Rev. 2023;153:105358. doi:10.1016/j.neubiorev.2023.105358 37597700 PMC10591841

[95] Pohjalainen T , Rinne JO , Någren K , SyvÄlahti E , Hietala J . Sex differences in the striatal dopamine D2 receptor binding characteristics in vivo. AJP. 1998;155:768‐773. doi:10.1176/ajp.155.6.768 9619148

[96] Aleman A , Kahn RS , Selten J‐P . Sex Differences in the risk of schizophrenia: evidence from meta‐analysis. Archiv General Psychiatry. 2003;60:565‐571. doi:10.1001/archpsyc.60.6.565 12796219

[97] Brown AK , Mandelkern MA , Farahi J , et al. Sex differences in striatal dopamine D2/D3 receptor availability in smokers and non‐smokers. Int J Neuropsychopharmacol. 2012;15:989‐994. doi:10.1017/S1461145711001957 22243762 PMC4113216

[98] Iwaki H , Blauwendraat C , Leonard HL , et al. Differences in the presentation and progression of Parkinson's disease by sex. Movement Disorders. 2021;36:106‐117. doi:10.1002/mds.28312 33002231 PMC7883324

[99] Kaasinen V , Någren K , Hietala J , Farde L , Rinne JO . Sex differences in extrastriatal dopamine D2‐like receptors in the human brain. AJP. 2001;158:308‐311. doi:10.1176/appi.ajp.158.2.308 11156817

[100] Laakso A , Vilkman H , Bergman J , et al. Sex differences in striatal presynaptic dopamine synthesis capacity in healthy subjects. Biol Psychiatry. 2002;52:759‐763. doi:10.1016/S0006-3223(02)01369-0 12372667

[101] Lavalaye J , Booij J , Reneman L , Habraken JBA , Van Royen EA . Effect of age and gender on dopamine transporter imaging with [ 123 I]FP‐CIT SPET in healthy volunteers. Eur J Nucl Med Mol Imaging. 2000;27:867‐869. doi:10.1007/s002590000279 10952500

[102] Manza P , Shokri‐Kojori E , Wiers CE , et al. Sex differences in methylphenidate‐induced dopamine increases in ventral striatum. Mol Psychiatry. 2022;27:939‐946. doi:10.1038/s41380-021-01294-9 34707237 PMC9043036

[103] Mozley LH , Gur RC , Mozley PD , Gur RE . Striatal dopamine transporters and cognitive functioning in healthy men and women. AJP. 2001;158:1492‐1499. doi:10.1176/appi.ajp.158.9.1492 11532737

[104] Munro CA , McCaul ME , Wong DF , et al. Sex differences in striatal dopamine release in healthy adults. Biol Psychiatry. 2006;59:966‐974. doi:10.1016/j.biopsych.2006.01.008 16616726

[105] Kritzer MF , Creutz LM . Region and sex differences in constituent dopamine neurons and immunoreactivity for intracellular estrogen and androgen receptors in mesocortical projections in rats. J Neurosci. 2008;28:9525‐9535. doi:10.1523/JNEUROSCI.2637-08.2008 18799684 PMC2613180

[106] Walker QD , Rooney MB , Wightman RM , Kuhn CM . Dopamine release and uptake are greater in female than male rat striatum as measured by fast cyclic voltammetry. Neuroscience. 2000;95:1061‐1070. doi:10.1016/S0306-4522(99)00500-X 10682713

[107] Jacobs E , D'Esposito M . Estrogen shapes dopamine‐dependent cognitive processes: implications for women's health. J Neurosci. 2011;31:5286‐5293. doi:10.1523/JNEUROSCI.6394-10.2011 21471363 PMC3089976

[108] Taylor CM , Furman DJ , Berry AS , et al. Striatal dopamine synthesis and cognitive flexibility differ between hormonal contraceptive users and nonusers. Cerebral Cortex. 2023;33:8485‐8495. doi:10.1093/cercor/bhad134 37160338 PMC10321119

[109] Xiao L , Becker JB . Quantitative microdialysis determination of extracellular striatal dopamine concentration in male and female rats: effects of estrous cycle and gonadectomy. Neurosci Lett. 1994;180:155‐158. doi:10.1016/0304-3940(94)90510-X 7700570

[110] Kritzer MF , Adler A , Locklear M . Androgen effects on mesoprefrontal dopamine systems in the adult male brain. Neuroscience. 2025;568:519‐534. doi:10.1016/j.neuroscience.2024.07.001 38977069

[111] Locklear MN , Michaelos M , Collins WF , Kritzer MF . Gonadectomy but not biological sex affects burst‐firing in dopamine neurons of the ventral tegmental area and in prefrontal cortical neurons projecting to the ventral tegmentum in adult rats. Eur J Neurosci. 2017;45:106‐120. doi:10.1111/ejn.13380 27564091

[112] Kokras N , Pastromas N , Papasava D , de Bournonville C , Cornil CA , Dalla C . Sex differences in behavioral and neurochemical effects of gonadectomy and aromatase inhibition in rats. Psychoneuroendocrinology. 2018;87:93‐107. doi:10.1016/j.psyneuen.2017.10.007 29054014

[113] Aubele T , Kritzer MF . Gonadectomy and hormone replacement affects in vivo basal extracellular dopamine levels in the prefrontal cortex but not motor cortex of adult male rats. Cereb Cortex. 2011;21:222‐232. doi:10.1093/cercor/bhq083 20466748 PMC3025724

[114] Aubele T , Kritzer MF . Androgen influence on prefrontal dopamine systems in adult male rats: localization of cognate intracellular receptors in medial prefrontal projections to the ventral tegmental area and effects of gonadectomy and hormone replacement on glutamate‐stimulated extracellular dopamine level. Cereb Cortex. 2012;22:1799‐1812. doi:10.1093/cercor/bhr258 21940701 PMC3500858

[115] Kemppainen N , Laine M , Laakso MP , et al. Hippocampal dopamine D2 receptors correlate with memory functions in Alzheimer's disease. Eur J Neurosci. 2003;18:149‐154. doi:10.1046/j.1460-9568.2003.02716.x 12859348

[116] Nagaraja D , Jayashree S . Randomized study of the dopamine receptor agonist piribedil in the treatment of mild cognitive impairment. AJP. 2001;158:1517‐1519. doi:10.1176/appi.ajp.158.9.1517 11532743

[117] Pan X , Kaminga AC , Wen SW , Wu X , Acheampong K , Liu A . Dopamine and dopamine receptors in Alzheimer's Disease: a systematic review and network meta‐analysis. Frontiers in Aging Neuroscience. 2019;11:175. 31354471 10.3389/fnagi.2019.00175 PMC6637734

[118] Ciampa CJ , Morin TM , Murphy A , Joie RL , Landau SM , Berry AS . *DAT1* and *BDNF* polymorphisms interact to predict Aβ and tau pathology. Neurobiol Aging. 2024;133:115‐124. doi:10.1016/j.neurobiolaging.2023.10.009 37948982 PMC10872994

[119] Roussotte FF , Gutman BA , Hibar DP , Madsen SK , Narr KL , Thompson PM . Carriers of a common variant in the dopamine transporter gene have greater dementia risk, cognitive decline, and faster ventricular expansion. Alzheimer's Dementia. 2015;11:1153‐1162. doi:10.1016/j.jalz.2014.10.011 PMC4465053 25496873

[120] Beach TG , Sue LI , Walker DG , et al. Striatal amyloid plaque density predicts braak neurofibrillary stage and clinicopathological alzheimer's disease: implications for amyloid imaging. J Alzheimer's Disease. 2012;28:869‐876. doi:10.3233/JAD-2011-111340 22112552 PMC3760731

[121] Stratmann K , Heinsen H , Korf H‐W , et al. Precortical phase of Alzheimer's disease (AD)‐related Tau cytoskeletal pathology. Brain Pathol. 2016;26:371‐386. doi:10.1111/bpa.12289 26193084 PMC4720581

[122] Nobili A , Latagliata EC , Viscomi MT , et al. Dopamine neuronal loss contributes to memory and reward dysfunction in a model of Alzheimer's disease. Nat Commun. 2017;8:14727. doi:10.1038/ncomms14727 28367951 PMC5382255

[123] Ionescu‐Tucker A , Cotman CW . Emerging roles of oxidative stress in brain aging and Alzheimer's disease. Neurobiol Aging. 2021;107:86‐95. doi:10.1016/j.neurobiolaging.2021.07.014 34416493

[124] Nam E , Derrick JS , Lee S , et al. Regulatory activities of dopamine and its derivatives toward metal‐free and metal‐induced amyloid‐β aggregation, oxidative stress, and inflammation in Alzheimer's disease. ACS Chem Neurosci. 2018;9:2655‐2666. doi:10.1021/acschemneuro.8b00122 29782798

[125] Eikelboom WS , Pan M , Ossenkoppele R , et al. Sex differences in neuropsychiatric symptoms in Alzheimer's disease dementia: a meta‐analysis. Alzheimer's Res Therapy. 2022;14:48. doi:10.1186/s13195-022-00991-z PMC8978393 35379344

[126] Lansdell TA , Xu H , Galligan JJ , Dorrance AM . Effects of striatal amyloidosis on the dopaminergic system and behavior: a comparative study in male and female 5XFAD mice. J Alzheimer's Dis. 2023;94:1361‐1375. doi:10.3233/JAD-220905 37424461 PMC12543006

[127] Pirskanen M , Hiltunen M , Mannermaa A , et al. Estrogen receptor beta gene variants are associated with increased risk of Alzheimer's disease in women. Eur J Hum Genet. 2005;13:1000‐1006. doi:10.1038/sj.ejhg.5201447 15944651

[128] Oveisgharan S , Yang J , Yu L , et al. Estrogen receptor genes, cognitive decline, and Alzheimer disease. Neurology. 2023;100:e1474‐87. doi:10.1212/WNL.0000000000206833 36697247 PMC10104608

[129] Lopez‐Lee C , Torres ERS , Carling G , Gan L . Mechanisms of sex differences in Alzheimer's disease. Neuron. 2024;112:1208‐1221. doi:10.1016/j.neuron.2024.01.024 38402606 PMC11076015

[130] Habibi P , Shahidi S , Khajvand‐Abedini M , et al. Effect of young plasma therapy on cognition, oxidative stress, miRNA‐134, BDNF, CREB, and SIRT‐1 expressions and neuronal survey in the hippocampus of aged ovariectomized rats with Alzheimer's. Brain Sci. 2024;14:656. doi:10.3390/brainsci14070656 39061398 PMC11274886

[131] Pacelli C , Giguère N , Bourque M‐J , Lévesque M , Slack RS , Trudeau L‐É . Elevated mitochondrial bioenergetics and axonal arborization size are key contributors to the vulnerability of dopamine neurons. Current Biology. 2015;25:2349‐2360. doi:10.1016/j.cub.2015.07.050 26320949

[132] Pissadaki EK , Bolam JP . The energy cost of action potential propagation in dopamine neurons: clues to susceptibility in Parkinson's disease. Front Comput Neurosci. 2013;7:13. doi:10.3389/fncom.2013.00013 23515615 PMC3600574

[133] Laws KR , Irvine K , Gale TM . Sex differences in cognitive impairment in Alzheimer's disease. World J Psychiatry. 2016;6:54‐65. doi:10.5498/wjp.v6.i1.54 27014598 PMC4804268

[134] Ceyzériat K , Gloria Y , Tsartsalis S , et al. Alterations in dopamine system and in its connectivity with serotonin in a rat model of Alzheimer's disease. Brain Communications. 2021;3:fcab029. doi:10.1093/braincomms/fcab029 34286270 PMC8287930

[135] Schwarz LA , Luo L . Organization of the locus coeruleus‐norepinephrine system. Current Biology. 2015;25:R1051‐6. doi:10.1016/j.cub.2015.09.039 26528750

[136] Kempadoo KA , Mosharov EV , Choi SJ , Sulzer D , Kandel ER . Dopamine release from the locus coeruleus to the dorsal hippocampus promotes spatial learning and memory. Proc National Acad Sci. 2016;113:14835‐14840. doi:10.1073/pnas.1616515114 PMC5187750 27930324

[137] Bueichekú E , Diez I , Kim C‐M , et al. Spatiotemporal patterns of locus coeruleus integrity predict cortical tau and cognition. Nat Aging. 2024;4:625‐637. doi:10.1038/s43587-024-00626-y 38664576 PMC11108787

[138] Braak H , Del Tredici K . The pathological process underlying Alzheimer's disease in individuals under thirty. Acta Neuropathologica. 2011;121:171‐181. doi:10.1007/s00401-010-0789-4 21170538

[139] Braak H , Del Tredici K . Neuroanatomy and pathology of sporadic Alzheimer's disease. Adv Anat Embryol Cell Biol. 2015;215:1‐162. 25920101

[140] Poe GR , Foote S , Eschenko O , et al. Locus coeruleus: a new look at the blue spot. Nat Rev Neurosci. 2020;21:644‐659. doi:10.1038/s41583-020-0360-9 32943779 PMC8991985

[141] Aston‐Jones G , Cohen JD . Adaptive gain and the role of the locus coeruleus–norepinephrine system in optimal performance. J Comparative Neurol. 2005;493:99‐110. doi:10.1002/cne.20723 16254995

[142] Sara SJ . The locus coeruleus and noradrenergic modulation of cognition. Nat Rev Neurosci. 2009;10:211‐223. doi:10.1038/nrn2573 19190638

[143] Mather M , Harley CW . The locus coeruleus: essential for maintaining cognitive function and the aging brain. Trends Cogn Sci. 2016;20:214‐226. doi:10.1016/j.tics.2016.01.001 26895736 PMC4761411

[144] Abercrombie ED , Keller RW , Zigmond MJ . Characterization of hippocampal norepinephrine release as measured by microdialysis perfusion: pharmacological and behavioral studies. Neuroscience. 1988;27:897‐904. doi:10.1016/0306-4522(88)90192-3 3252176

[145] Morrison JH , Grzanna R , Molliver ME , Coyle JT . The distribution and orientation of noradrenergic fibers in neocortex of the rat: an immunofluorescence study. J Comp Neurol. 1978;181:17‐39. doi:10.1002/cne.901810103 355267

[146] Pentkowski NS , Rogge‐Obando KK , Donaldson TN , Bouquin SJ , Clark BJ . Anxiety and Alzheimer's disease: behavioral analysis and neural basis in rodent models of Alzheimer's‐related neuropathology. Neurosci Biobehav Rev. 2021;127:647‐658. doi:10.1016/j.neubiorev.2021.05.005 33979573 PMC8292229

[147] James T , Kula B , Choi S , Khan SS , Bekar LK , Smith NA . Locus coeruleus in memory formation and Alzheimer's disease. Eur J Neurosci. 2021;54:6948‐6959. doi:10.1111/ejn.15045 33190318 PMC8121900

[148] Braak H , Del Tredici K . Where, when, and in what form does sporadic Alzheimer's disease begin?. Curr Opin Neurol. 2012;25:708‐714. doi:10.1097/WCO.0b013e32835a3432 23160422

[149] Luckey AM , Robertson IH , Lawlor B , Mohan A , Vanneste S . Sex differences in locus coeruleus: a heuristic approach that may explain the increased risk of Alzheimer's disease in females. J Alzheimer's Dis. 2021;83:505‐522. doi:10.3233/JAD-210404 34334399

[150] Joshi N , Chandler D . Sex and the noradrenergic system. Handb Clin Neurol. 2020;175:167‐176. doi:10.1016/B978-0-444-64123-6.00012-6 33008523

[151] Curtis AL , Bethea T , Valentino RJ . Sexually dimorphic responses of the brain norepinephrine system to stress and corticotropin‐releasing factor. Neuropsychopharmacology. 2006;31:544‐554. doi:10.1038/sj.npp.1300875 16123744

[152] Bangasser D , Zhang X , Garachh V , Hanhauser E , Valentino R . Sexual dimorphism in locus coeruleus dendritic morphology: a structural basis for sex differences in emotional arousal. Physiol Behav. 2011;103:342‐351. doi:10.1016/j.physbeh.2011.02.037 21362438 PMC3081983

[153] Luque JM , de Blas MR , Segovia S , Guillamón A . Sexual dimorphism of the dopamine‐beta‐hydroxylase‐immunoreactive neurons in the rat locus ceruleus. Brain Res Dev Brain Res. 1992;67:211‐215. doi:10.1016/0165-3806(92)90221-h 1511516

[154] Pinos H , Collado P , Rodríguez‐Zafra M , Rodríguez C , Segovia S , Guillamón A . The development of sex differences in the locus coeruleus of the rat. Brain Res Bull. 2001;56:73‐78. doi:10.1016/s0361-9230(01)00540-8 11604252

[155] Babstock D , Malsbury CW , Harley CW . The dorsal locus coeruleus is larger in male than in female Sprague–Dawley rats. Neurosci Lett. 1997;224:157‐160. doi:10.1016/S0304-3940(97)13462-0 9131660

[156] Mulvey B , Bhatti DL , Gyawali S , et al. Molecular and functional sex differences of noradrenergic neurons in the mouse locus coeruleus. Cell Rep. 2018;23:2225‐2235. doi:10.1016/j.celrep.2018.04.054 29791834 PMC6070358

[157] Serova L , Rivkin M , Nakashima A , Sabban EL . Estradiol stimulates gene expression of norepinephrine biosynthetic enzymes in rat locus coeruleus. Neuroendocrinology. 2002;75:193‐200. doi:10.1159/000048237 11914591

[158] Thanky NR , Son JH , Herbison AE . Sex differences in the regulation of tyrosine hydroxylase gene transcription by estrogen in the locus coeruleus of TH9‐LacZ transgenic mice. Brain Res Mol Brain Res. 2002;104:220‐226. doi:10.1016/s0169-328x(02)00383-2 12225877

[159] Lee J , Wang Z‐M , Messi ML , Milligan C , Furdui CM , Delbono O . Sex differences in single neuron function and proteomics profiles examined by patch‐clamp and mass spectrometry in the locus coeruleus of the adult mouse. Acta Physiol (Oxf). 2024;240:e14123. doi:10.1111/apha.14123 38459766 PMC11021178

[160] Heneka MT , Ramanathan M , Jacobs AH , et al. Locus ceruleus degeneration promotes Alzheimer pathogenesis in amyloid precursor protein 23 transgenic mice. J Neurosci. 2006;26:1343‐1354. doi:10.1523/JNEUROSCI.4236-05.2006 16452658 PMC6675491

[161] O'Neil JN , Mouton PR , Tizabi Y , et al. Catecholaminergic neuronal loss in locus coeruleus of aged female dtg APP/PS1 mice. J Chem Neuroanat. 2007;34:102‐107. doi:10.1016/j.jchemneu.2007.05.008 17658239 PMC5483173

[162] Wilson RS , Nag S , Boyle PA , et al. Neural reserve, neuronal density in the locus ceruleus, and cognitive decline. Neurology. 2013;80:1202‐1208. doi:10.1212/WNL.0b013e3182897103 23486878 PMC3691778

[163] Kelly SC , He B , Perez SE , Ginsberg SD , Mufson EJ , Counts SE . Locus coeruleus cellular and molecular pathology during the progression of Alzheimer's disease. Acta Neuropathol Commun. 2017;5:8. doi:10.1186/s40478-017-0411-2 28109312 PMC5251221

[164] Prokopiou PC , Engels‐Domínguez N , Papp KV , et al. Lower novelty‐related locus coeruleus function is associated with Aβ‐related cognitive decline in clinically healthy individuals. Nat Commun. 2022;13:1571. doi:10.1038/s41467-022-28986-2 35322012 PMC8943159

[165] Theofilas P , Ehrenberg AJ , Dunlop S , et al. Locus coeruleus volume and cell population changes during Alzheimer's disease progression: a stereological study in human postmortem brains with potential implication for early‐stage biomarker discovery. Alzheimers Dement. 2017;13:236‐246. doi:10.1016/j.jalz.2016.06.2362 27513978 PMC5298942

[166] Jacobs HIL , Becker JA , Kwong K , Munera D , et al. Waning locus coeruleus integrity precedes cortical tau accrual in preclinical autosomal dominant Alzheimer's disease. Alzheimers Dement. 2023;19:169‐180. doi:10.1002/alz.12656 35298083 PMC9481982

[167] Dahl MJ , Mather M , Werkle‐Bergner M , et al. Locus coeruleus integrity is related to tau burden and memory loss in autosomal‐dominant Alzheimer's disease. Neurobiol Aging. 2022;112:39‐54. doi:10.1016/j.neurobiolaging.2021.11.006 35045380 PMC8976827

[168] Evans AK , Park HH , Woods CE , et al. Impact of noradrenergic inhibition on neuroinflammation and pathophysiology in mouse models of Alzheimer's disease. J Neuroinflammation. 2024;21:322. doi:10.1186/s12974-024-03306-1 39696597 PMC11657531

[169] Kelly SC , McKay EC , Beck JS , Collier TJ , Dorrance AM , Counts SE . locus coeruleus degeneration induces forebrain vascular pathology in a transgenic rat model of Alzheimer's disease. J Alzheimers Dis. 2019;70:371‐388. doi:10.3233/JAD-190090 31177220 PMC6929678

[170] Kummer MP , Hammerschmidt T , Martinez A , et al. Ear2 deletion causes early memory and learning deficits in APP/PS1 mice. J Neurosci. 2014;34:8845‐8854. doi:10.1523/JNEUROSCI.4027-13.2014 24966384 PMC4147626

[171] Hammerschmidt T , Kummer MP , Terwel D , et al. Selective loss of noradrenaline exacerbates early cognitive dysfunction and synaptic deficits in APP/PS1 mice. Biol Psychiatry. 2013;73:454‐463. doi:10.1016/j.biopsych.2012.06.013 22883210 PMC4712953

[172] Kalinin S , Gavrilyuk V , Polak PE , et al. Noradrenaline deficiency in brain increases beta‐amyloid plaque burden in an animal model of Alzheimer's disease. Neurobiol Aging. 2007;28:1206‐1214. doi:10.1016/j.neurobiolaging.2006.06.003 16837104

[173] Braun D , Feinstein DL . The locus coeruleus neuroprotective drug vindeburnol normalizes behavior in the 5xFAD transgenic mouse model of Alzheimer's disease. Brain Res. 2019;1702:29‐37. doi:10.1016/j.brainres.2017.12.028 29274883

[174] Iba M , McBride JD , Guo JL , Zhang B , Trojanowski JQ , Lee VM‐Y . Tau pathology spread in PS19 tau transgenic mice following locus coeruleus (LC) injections of synthetic tau fibrils is determined by the LC's afferent and efferent connections. Acta Neuropathol. 2015;130:349‐362. doi:10.1007/s00401-015-1458-4 26150341 PMC4545685

[175] Ahnaou A , Walsh C , Manyakov NV , Youssef SA , Drinkenburg WH . Early electrophysiological disintegration of hippocampal neural networks in a novel locus coeruleus tau‐seeding mouse model of Alzheimer's disease. Neural Plast. 2019;2019:6981268. doi:10.1155/2019/6981268 31285742 PMC6594257

[176] Rorabaugh JM , Chalermpalanupap T , Botz‐Zapp CA , et al. Chemogenetic locus coeruleus activation restores reversal learning in a rat model of Alzheimer's disease. Brain. 2017;140:3023‐3038. doi:10.1093/brain/awx232 29053824 PMC5841201

[177] Kelberman MA , Rorabaugh JM , Anderson CR , et al. Age‐dependent dysregulation of locus coeruleus firing in a transgenic rat model of Alzheimer's disease. Neurobiology of Aging. 2023;125:98‐108. doi:10.1016/j.neurobiolaging.2023.01.016 36889122 PMC10038926

[178] Khan KM , Balasubramanian N , Gaudencio G , et al. Human tau‐overexpressing mice recapitulate brainstem involvement and neuropsychiatric features of early Alzheimer's disease. Acta Neuropathol Commun. 2023;11:57. doi:10.1186/s40478-023-01546-5 37009893 PMC10069039

[179] Flynn CM , Omoluabi T , Janes AM , et al. Targeting early tau pathology: probiotic diet enhances cognitive function and reduces inflammation in a preclinical Alzheimer's model. Alzheimers Res Ther. 2025;17:24. doi:10.1186/s13195-025-01674-1 39827356 PMC11742226

[180] Ghosh A , Torraville SE , Mukherjee B , et al. An experimental model of Braak's pretangle proposal for the origin of Alzheimer's disease: the role of locus coeruleus in early symptom development. Alzheimers Res Ther. 2019;11:59. doi:10.1186/s13195-019-0511-2 31266535 PMC6607586

[181] Omoluabi T , Hasan Z , Piche JE , et al. Locus coeruleus vulnerability to tau hyperphosphorylation in a rat model. Aging Cell. 2025;24:e14405. doi:10.1111/acel.14405 39520141 PMC11896524

[182] Omoluabi T , Torraville SE , Maziar A , et al. Novelty‐like activation of locus coeruleus protects against deleterious human pretangle tau effects while stress‐inducing activation worsens its effects. Alzheimers Dement (N Y). 2021;7:e12231. doi:10.1002/trc2.12231 35005208 PMC8719346

[183] Bangasser DA , Curtis A , Reyes BAS , et al. Sex differences in corticotropin‐releasing factor receptor signaling and trafficking: potential role in female vulnerability to stress‐related psychopathology. Mol Psychiatry. 2010;15:877, 896‐904. doi:10.1038/mp.2010.66 20548297 PMC2935505

[184] Valentino RJ , Bangasser DA . Sex‐biased cellular signaling: molecular basis for sex differences in neuropsychiatric diseases. Dialogues Clin Neurosci. 2016;18:385‐393. doi:10.31887/DCNS.2016.18.4/rvalentino 28179810 PMC5286724

[185] Bangasser DA . Sex differences in stress‐related receptors: ″micro″ differences with ″macro″ implications for mood and anxiety disorders. Biol Sex Differ. 2013;4:2. doi:10.1186/2042-6410-4-2 23336736 PMC3556142

[186] Enman NM , Reyes BAS , Shi Y , Valentino RJ , Van Bockstaele EJ . Sex differences in morphine‐induced trafficking of mu‐opioid and corticotropin‐releasing factor receptors in locus coeruleus neurons. Brain Res. 2019;1706:75‐85. doi:10.1016/j.brainres.2018.11.001 30391476

[187] Bates MLS , Arner JR , Curtis AL , Valentino R , Bhatnagar S . Sex‐specific alterations in corticotropin‐releasing factor regulation of coerulear‐cortical network activity. Neuropharmacology. 2023;223:109317. doi:10.1016/j.neuropharm.2022.109317 36334761

[188] Bangasser DA , Dong H , Carroll J , et al. Corticotropin‐releasing factor overexpression gives rise to sex differences in Alzheimer's disease‐related signaling. Mol Psychiatry. 2017;22:1126‐1133. doi:10.1038/mp.2016.185 27752081 PMC5395355

[189] Ross JA , Alexis R , Reyes BAS , Risbrough V , Van Bockstaele EJ . Localization of amyloid beta peptides to locus coeruleus and medial prefrontal cortex in corticotropin releasing factor overexpressing male and female mice. Brain Struct Funct. 2019;224:2385‐2405. doi:10.1007/s00429-019-01915-8 31250157 PMC7371412

[190] Iversen LL , Rossor MN , Reynolds GP , et al. Loss of pigmented dopamine‐beta‐hydroxylase positive cells from locus coeruleus in senile dementia of Alzheimer's type. Neurosci Lett. 1983;39:95‐100. doi:10.1016/0304-3940(83)90171-4 6633940

[191] Pearson J , Goldstein M , Markey K , Brandeis L . Human brainstem catecholamine neuronal anatomy as indicated by immunocytochemistry with antibodies to tyrosine hydroxylase. Neuroscience. 1983;8:3‐32. doi:10.1016/0306-4522(83)90023-4 6132348

[192] Kemper CM , O'Connor DT , Westlund KN . Immunocytochemical localization of dopamine‐beta‐hydroxylase in neurons of the human brain stem. Neuroscience. 1987;23:981‐989. doi:10.1016/0306-4522(87)90173-4 3437997

[193] German DC , Walker BS , Manaye K , Smith WK , Woodward DJ , North AJ . The human locus coeruleus: computer reconstruction of cellular distribution. J Neurosci. 1988;8:1776‐1788. doi:10.1523/JNEUROSCI.08-05-01776.1988 3367220 PMC6569207

[194] Chan‐Palay V , Asan E . Alterations in catecholamine neurons of the locus coeruleus in senile dementia of the Alzheimer type and in Parkinson's disease with and without dementia and depression. J Comp Neurol. 1989;287:373‐392. doi:10.1002/cne.902870308 2570794

[195] Chan‐Palay V , Asan E . Quantitation of catecholamine neurons in the locus coeruleus in human brains of normal young and older adults and in depression. J Comp Neurol. 1989;287:357‐372. doi:10.1002/cne.902870307 2570793

[196] Baker KG , Törk I , Hornung JP , Halasz P . The human locus coeruleus complex: an immunohistochemical and three dimensional reconstruction study. Exp Brain Res. 1989;77:257‐270. doi:10.1007/BF00274983 2571514

[197] Chan‐Palay V . Locus coeruleus and norepinephrine in Parkinson's disease. Jpn J Psychiatry Neurol. 1991;45:519‐521. doi:10.1111/j.1440-1819.1991.tb02540.x 1722263

[198] German DC , Manaye KF , White CL , et al. Disease‐specific patterns of locus coeruleus cell loss. Ann Neurol. 1992;32:667‐676. doi:10.1002/ana.410320510 1449247

[199] Kubis N , Faucheux BA , Ransmayr G , et al. Preservation of midbrain catecholaminergic neurons in very old human subjects. Brain. 2000;123(Pt 2):366‐373. doi:10.1093/brain/123.2.366 10648443

[200] Busch C , Bohl J , Ohm TG . Spatial, temporal and numeric analysis of Alzheimer changes in the nucleus coeruleus. Neurobiol Aging. 1997;18:401‐406. doi:10.1016/s0197-4580(97)00035-3 9330971

[201] Vijayashankar N , Brody H . A quantitative study of the pigmented neurons in the nuclei locus coeruleus and subcoeruleus in man as related to aging. J Neuropathol Exp Neurol. 1979;38:490‐497. doi:10.1097/00005072-197909000-00004 469568

[202] Mouton PR , Pakkenberg B , Gundersen HJ , Price DL . Absolute number and size of pigmented locus coeruleus neurons in young and aged individuals. J Chem Neuroanat. 1994;7:185‐190. doi:10.1016/0891-0618(94)90028-0 7848573

[203] Ohm TG , Busch C , Bohl J . Unbiased estimation of neuronal numbers in the human nucleus coeruleus during aging. Neurobiol Aging. 1997;18:393‐399. doi:10.1016/s0197-4580(97)00034-1 9330970

[204] Mann DM , Yates PO . The effects of ageing on the pigmented nerve cells of the human locus caeruleous and substantia nigra. Acta Neuropathol. 1979;47:93‐97. doi:10.1007/BF00717030 474078

[205] Wree A , Braak H , Schleicher A , Zilles K . Biomathematical analysis of the neuronal loss in the aging human brain of both sexes, demonstrated in pigment preparations of the pars cerebellaris loci coerulei. Anat Embryol (Berl). 1980;160:105‐119. doi:10.1007/BF00315653 7469032

[206] Tomlinson BE , Irving D , Blessed G . Cell loss in the locus coeruleus in senile dementia of Alzheimer type. J Neurol Sci. 1981;49:419‐428. doi:10.1016/0022-510x(81)90031-9 7217992

[207] Buchman AS , Nag S , Shulman JM , et al. Locus coeruleus neuron density and parkinsonism in older adults without Parkinson's disease. Mov Disord. 2012;27:1625‐1631. doi:10.1002/mds.25142 23038629 PMC3628555

[208] Dugger BN , Murray ME , Boeve BF , et al. Neuropathological analysis of brainstem cholinergic and catecholaminergic nuclei in relation to rapid eye movement (REM) sleep behaviour disorder. Neuropathol Appl Neurobiol. 2012;38:142‐152. doi:10.1111/j.1365-2990.2011.01203.x 21696423 PMC3218297

[209] Keren NI , Taheri S , Vazey EM , et al. Histologic validation of locus coeruleus MRI contrast in post‐mortem tissue. Neuroimage. 2015;113:235‐245. doi:10.1016/j.neuroimage.2015.03.020 25791783 PMC4649944

[210] Eser RA , Ehrenberg AJ , Petersen C , et al. Selective vulnerability of brainstem nuclei in distinct tauopathies: a postmortem study. J Neuropathol Exp Neurol. 2018;77:149‐161. doi:10.1093/jnen/nlx113 29304218 PMC6251636

[211] Theofilas P , Ehrenberg AJ , Nguy A , et al. Probing the correlation of neuronal loss, neurofibrillary tangles, and cell death markers across the Alzheimer's disease Braak stages: a quantitative study in humans. Neurobiol Aging. 2018;61:1‐12. doi:10.1016/j.neurobiolaging.2017.09.007 29031088 PMC5705284

[212] Oh J , Eser RA , Ehrenberg AJ , et al. Profound degeneration of wake‐promoting neurons in Alzheimer's disease. Alzheimers Dement. 2019;15:1253‐1263. doi:10.1016/j.jalz.2019.06.3916 31416793 PMC6801040

[213] Zahola P , Hanics J , Pintér A , et al. Secretagogin expression in the vertebrate brainstem with focus on the noradrenergic system and implications for Alzheimer's disease. Brain Struct Funct. 2019;224:2061‐2078. doi:10.1007/s00429-019-01886-w 31144035 PMC6591208

[214] Tong Q , Chen L . Associations of Alzheimer's disease neuropathologic changes with clinical presentations of Parkinson's disease. J Alzheimers Dis. 2021;81:201‐207. doi:10.3233/JAD-210114 33720903

[215] Murray ME , Moloney CM , Kouri N , et al. Global neuropathologic severity of Alzheimer's disease and locus coeruleus vulnerability influences plasma phosphorylated tau levels. Molecular Neurodegeneration. 2022;17:85. doi:10.1186/s13024-022-00578-0 36575455 PMC9795667

[216] Torso M , Ridgway GR , Valotti M , et al. In vivo cortical diffusion imaging relates to Alzheimer's disease neuropathology. Alzheimers Res Ther. 2023;15:165. doi:10.1186/s13195-023-01309-3 37794477 PMC10548768

[217] Beardmore R , Durkin M , Zayee‐Mellick F , et al. Changes in the locus coeruleus during the course of Alzheimer's disease and their relationship to cortical pathology. Neuropathol Appl Neurobiol. 2024;50:e12965. doi:10.1111/nan.12965 38374720

[218] Fructuoso M , Vermeiren Y , Boluda S , et al. Disease‐specific neuropathological alterations of the locus coeruleus in Alzheimer's disease, Down syndrome, and Parkinson's disease. Alzheimers Dement. 2025;21:e70262. doi:10.1002/alz.70262 40501099 PMC12159339

[219] Hary AT , Chadha S , Mercaldo N , et al. Locus coeruleus tau validates and informs high‐resolution MRI in aging and at earliest Alzheimer's pathology stages. Acta Neuropathol Commun. 2025;13:44. doi:10.1186/s40478-025-01957-6 40022196 PMC11871710

[220] Wilson RS , Nag S , Boyle PA , et al. Brainstem aminergic nuclei and late‐life depressive symptoms. JAMA Psychiatry. 2013;70:1320‐1328. doi:10.1001/jamapsychiatry.2013.2224 24132763 PMC3856195

[221] Kelly SC , Nelson PT , Counts SE . Pontine arteriolosclerosis and locus coeruleus oxidative stress differentiate resilience from mild cognitive impairment in a clinical pathologic cohort. J Neuropathol Exp Neurol. 2021;80:325‐335. doi:10.1093/jnen/nlab017 33709107 PMC7985827

[222] Jacobs HIL , Becker JA , Kwong K , et al. In vivo and neuropathology data support locus coeruleus integrity as indicator of Alzheimer's disease pathology and cognitive decline. Sci Transl Med. 2021;13:eabj2511. doi:10.1126/scitranslmed.abj2511 34550726 PMC8641759

[223] Beckers E , Riphagen JM , Van Egroo M , Bennett DA , Jacobs HIL . Sparse asymmetry in locus coeruleus pathology in Alzheimer's disease. J Alzheimer's Disease. 2024;99:105‐111. doi:10.3233/JAD-231328 38607758 PMC11091606

[224] Freeze WM , Van Veluw SJ , Jansen WJ , Bennett DA , Jacobs HIL . Locus coeruleus pathology is associated with cerebral microangiopathy at autopsy. Alzheimers Dement. 2023;19:5023‐5035. doi:10.1002/alz.13096 37095709 PMC10593911

[225] Van Egroo M , van Someren EJW , Grinberg LT , Bennett DA , Jacobs HIL . Associations of 24‐hour rest‐activity rhythm fragmentation, cognitive decline, and postmortem locus coeruleus hypopigmentation in Alzheimer's disease. Ann Neurol. 2024;95:653‐664. doi:10.1002/ana.26880 38407546 PMC11875531

[226] Betts MJ , Cardenas‐Blanco A , Kanowski M , Jessen F , Düzel E . In vivo MRI assessment of the human locus coeruleus along its rostrocaudal extent in young and older adults. Neuroimage. 2017;163:150‐159. doi:10.1016/j.neuroimage.2017.09.042 28943414

[227] Trujillo P , Petersen KJ , Cronin MJ , et al. Quantitative magnetization transfer imaging of the human locus coeruleus. Neuroimage. 2019;200:191‐198. doi:10.1016/j.neuroimage.2019.06.049 31233908 PMC6934172

[228] Priovoulos N , Jacobs HIL , Ivanov D , Uludağ K , Verhey FRJ , Poser BA . High‐resolution in vivo imaging of human locus coeruleus by magnetization transfer MRI at 3T and 7T. Neuroimage. 2018;168:427‐436. doi:10.1016/j.neuroimage.2017.07.045 28743460

[229] Keren NI , Lozar CT , Harris KC , Morgan PS , Eckert MA . In‐vivo mapping of the human locus coeruleus. Neuroimage. 2009;47:1261‐1267. doi:10.1016/j.neuroimage.2009.06.012 19524044 PMC3671394

[230] Chen X , Huddleston DE , Langley J , et al. Simultaneous imaging of locus coeruleus and substantia nigra with a quantitative neuromelanin MRI approach. Magn Reson Imaging. 2014;32:1301‐1306. doi:10.1016/j.mri.2014.07.003 25086330

[231] Dahl MJ , Mather M , Düzel S , et al. Higher rostral locus coeruleus integrity is associated with better memory performance in older adults. Nat Hum Behav. 2019;3:1203‐1214. doi:10.1038/s41562-019-0715-2 31501542 PMC7203800

[232] Galgani A , Giorgi FS . Exploring the role of locus coeruleus in Alzheimer's disease: a comprehensive update on MRI studies and implications. Curr Neurol Neurosci Rep. 2023;23:925‐936. doi:10.1007/s11910-023-01324-9 38064152 PMC10724305

[233] Engels‐Domínguez N , Koops EA , Prokopiou PC , et al. State‐of‐the‐art imaging of neuromodulatory subcortical systems in aging and Alzheimer's disease: challenges and opportunities. Neurosci Biobehav Rev. 2023;144:104998. doi:10.1016/j.neubiorev.2022.104998 36526031 PMC9805533

[234] Kelberman M , Keilholz S , Weinshenker D . What's that (blue) spot on my MRI? Multimodal neuroimaging of the locus coeruleus in neurodegenerative disease. Front Neurosci. 2020;14:583421. 33122996 10.3389/fnins.2020.583421 PMC7573566

[235] Betts MJ , Kirilina E , Otaduy MCG , et al. Locus coeruleus imaging as a biomarker for noradrenergic dysfunction in neurodegenerative diseases. Brain. 2019;142:2558‐2571. doi:10.1093/brain/awz193 31327002 PMC6736046

[236] Krohn F , Lancini E , Ludwig M , et al. Noradrenergic neuromodulation in ageing and disease. Neurosci Biobehav Rev. 2023;152:105311. doi:10.1016/j.neubiorev.2023.105311 37437752

[237] Berger A , Koshmanova E , Beckers E , et al. Structural and functional characterization of the locus coeruleus in young and late middle‐aged individuals. Front Neuroimaging. 2023;2:1207844. doi:10.3389/fnimg.2023.1207844 37554637 PMC10406214

[238] Takahashi J , Shibata T , Sasaki M , et al. Detection of changes in the locus coeruleus in patients with mild cognitive impairment and Alzheimer's disease: high‐resolution fast spin‐echo T1‐weighted imaging. Geriatr Gerontol Int. 2015;15:334‐340. doi:10.1111/ggi.12280 24661561 PMC4405055

[239] Calarco N , Cassidy CM , Selby B , et al. Associations between locus coeruleus integrity and diagnosis, age, and cognitive performance in older adults with and without late‐life depression: an exploratory study. Neuroimage Clin. 2022;36:103182. doi:10.1016/j.nicl.2022.103182 36088841 PMC9474922

[240] Liu KY , Acosta‐Cabronero J , Cardenas‐Blanco A , et al. In vivo visualization of age‐related differences in the locus coeruleus. Neurobiol Aging. 2019;74:101‐111. doi:10.1016/j.neurobiolaging.2018.10.014 30447418 PMC6338679

[241] Shibata E , Sasaki M , Tohyama K , et al. Age‐related changes in locus ceruleus on neuromelanin magnetic resonance imaging at 3 Tesla. Magn Reson Med Sci. 2006;5:197‐200. doi:10.2463/mrms.5.197 17332710

[242] Riley E , Cicero N , Mabry SA , Swallow KM , Anderson AK , De Rosa E . Age‐related differences in locus coeruleus intensity across a demographically diverse sample. Neurobiol Aging. 2025;150:122‐131. doi:10.1016/j.neurobiolaging.2025.03.005 40101307 PMC11981832

[243] Bachman SL , Cole S , Yoo HJ , et al. Daily heart rate variability biofeedback training decreases locus coeruleus MRI contrast in younger adults in a randomized clinical trial. Int J Psychophysiol. 2023;193:112241. doi:10.1016/j.ijpsycho.2023.08.014 37647944 PMC10591988

[244] Bachman SL , Dahl MJ , Werkle‐Bergner M , et al. Locus coeruleus MRI contrast is associated with cortical thickness in older adults. Neurobiol Aging. 2021;100:72‐82. doi:10.1016/j.neurobiolaging.2020.12.019 33508564 PMC7920995

[245] Galgani A , Lombardo F , Frijia F , et al. Locus coeruleus sexual dimorphism and its impact on cognitive impairment and cortical atrophy in Alzheimer's disease. Neurodegener Dis. 2025:1‐14. doi:10.1159/000544882 40010329

[246] Galgani A , Lombardo F , Martini N , et al. Magnetic resonance imaging Locus Coeruleus abnormality in amnestic mild cognitive impairment is associated with future progression to dementia. Eur J Neurol. 2023;30:32‐46. doi:10.1111/ene.15556 36086917 PMC10092028

[247] Clewett DV , Lee T‐H , Greening S , Ponzio A , Margalit E , Mather M . Neuromelanin marks the spot: identifying a locus coeruleus biomarker of cognitive reserve in healthy aging. Neurobiol Aging. 2016;37:117‐126. doi:10.1016/j.neurobiolaging.2015.09.019 26521135 PMC5134892

[248] Beckers E , Van Egroo M , Ashton NJ , et al. Microstructural associations between locus coeruleus, cortical, and subcortical regions are modulated by astrocyte reactivity: a 7T MRI adult lifespan study. Cereb Cortex. 2024;34:bhae261. doi:10.1093/cercor/bhae261 38904081 PMC11190376

[249] Wearn A , Tremblay SA , Tardif CL , et al. Neuromodulatory subcortical nucleus integrity is associated with white matter microstructure, tauopathy and APOE status. Nat Commun. 2024;15:4706. doi:10.1038/s41467-024-48490-z 38830849 PMC11148077

[250] Bennett IJ , Langley J , Sun A , Solis K , Seitz AR , Hu XP . Locus coeruleus contrast and diffusivity metrics differentially relate to age and memory performance. Sci Rep. 2024;14:15372. doi:10.1038/s41598-024-66238-z 38965363 PMC11224383

[251] Zhang S , Hu S , Chao HH , Li C‐SR . Resting‐state functional connectivity of the locus coeruleus in humans: in comparison with the ventral tegmental area/substantia nigra pars compacta and the effects of age. Cereb Cortex. 2016;26:3413‐3427. doi:10.1093/cercor/bhv172 26223261 PMC4961017

[252] Um YH , Wang S‐M , Kang DW , et al. Sex‐related disparities in the resting state functional connectivity of the locus coeruelus and salience network in preclinical Alzheimer's disease. Int J Mol Sci. 2023;24:15092. doi:10.3390/ijms242015092 37894772 PMC10606651

[253] Filkowski MM , Olsen RM , Duda B , Wanger TJ , Sabatinelli D . Sex differences in emotional perception: meta analysis of divergent activation. Neuroimage. 2017;147:925‐933. doi:10.1016/j.neuroimage.2016.12.016 27988321

[254] Ludwig M , Yi Y‐J , Lüsebrink F , et al. Functional locus coeruleus imaging to investigate an ageing noradrenergic system. Commun Biol. 2024;7:777. doi:10.1038/s42003-024-06446-5 38937535 PMC11211439

[255] Kilpatrick LA , Gupta A , Meriwether D , et al. Differential brainstem connectivity according to sex and menopausal status in healthy men and women. Res Sq. 2024:rs.3.rs‐4875269. doi:10.21203/rs.3.rs-4875269/v1 PMC12007138 40251694

[256] Koops EA , Dutta J , Hanseeuw BJ , et al. Elevated locus coeruleus metabolism provides resilience against cognitive decline in preclinical Alzheimer's disease. Alzheimers Dement. 2025;21:e14385. doi:10.1002/alz.14385 39588792 PMC11772725

[257] Sundermann EE , Maki PM , Reddy S , Bondi MW , Biegon A . Women's higher brain metabolic rate compensates for early Alzheimer's pathology. Alzheimers Dement (Amst). 2020;12:e12121. doi:10.1002/dad2.12121 33251322 PMC7678742

[258] Helena C , Gustafsson J‐A , Korach K , Pfaff D , Anselmo‐Franci JA , Ogawa S . Effects of estrogen receptor alpha and beta gene deletion on estrogenic induction of progesterone receptors in the locus coeruleus in female mice. Endocrine. 2009;36:169‐177. doi:10.1007/s12020-009-9207-x 19551522 PMC4775101

[259] Brinton RD , Yao J , Yin F , Mack WJ , Cadenas E . Perimenopause as a neurological transition state. Nat Rev Endocrinol. 2015;11:393‐405. doi:10.1038/nrendo.2015.82 26007613 PMC9934205

[260] Buckley RF , O'Donnell A , McGrath ER , et al. menopause status moderates sex differences in Tau burden: a framingham PET study. Ann Neurol. 2022;92:11‐22. doi:10.1002/ana.26382 35471588 PMC9233144

[261] Brown A , Gervais NJ , Rieck J , et al. Women's brain health: midlife ovarian removal affects associative memory. Mol Neurobiol. 2023;60:6145‐6159. doi:10.1007/s12035-023-03424-6 37423941 PMC10533588

[262] Brown A , Gravelsins L , Gervais NJ , et al. Early midlife ovarian removal is associated with lower posterior hippocampal function. Alzheimer's Dementia. 2025;21:e14447. doi:10.1002/alz.14447 PMC11851323 39732509

[263] Brown A , Gervais NJ , Gravelsins L , et al. Effects of early midlife ovarian removal on medial temporal lobe gray matter volume and recognition memory. Hippocampus. 2025;35:e70012. doi:10.1002/hipo.70012 40156318 PMC11953763

[264] Calvo N , Gravelsins L , Brown A , et al. Cognitive and brain health in women with early bilateral salpingo‐oophorectomy: implications for risk, resilience, and subjective cognitive decline. Alzheimer's Dementia. 2025;21:e70454. doi:10.1002/alz.70454 PMC12333622 40779417

[265] Gervais NJ , Au A , Almey A , et al. Cognitive markers of dementia risk in middle‐aged women with bilateral salpingo‐oophorectomy prior to menopause. Neurobiol Aging. 2020;94:1‐6. doi:10.1016/j.neurobiolaging.2020.04.019 32497876

[266] Gervais NJ , Gravelsins L , Brown A , et al. Scene memory and hippocampal volume in middle‐aged women with early hormone loss. Neurobiol Aging. 2022;117:97‐106. doi:10.1016/j.neurobiolaging.2022.05.003 35696793

[267] Gervais NJ , Gravelsins L , Brown A , et al. Disturbed sleep is associated with reduced verbal episodic memory and entorhinal cortex volume in younger middle‐aged women with risk‐reducing early ovarian removal. Front Endocrinol. 2023;14:1265470. doi:10.3389/fendo.2023.1265470 PMC10584319 37859979

[268] Witt ST , Brown A , Gravelsins L , et al. Gray matter volume in women with the BRCA mutation with and without ovarian removal: evidence for increased risk of late‐life Alzheimer's disease or dementia. Menopause. 2024;31:608. doi:10.1097/GME.0000000000002361 38688467

[269] Huang KW , Ochandarena NE , Philson AC , et al. Molecular and anatomical organization of the dorsal raphe nucleus. eLife. 2019;8:e46464. doi:10.7554/eLife.46464 31411560 PMC6726424

[270] Jauhar S , Cowen PJ , Browning M . Fifty years on: serotonin and depression. J Psychopharmacol. 2023;37:237‐241. doi:10.1177/02698811231161813 36938996 PMC10076339

[271] Bremshey S , Groß J , Renken K , Masseck OA . The role of serotonin in depression—A historical roundup and future directions. J Neurochem. 2024;168:1751‐1779. doi:10.1111/jnc.16097 38477031

[272] Weissman MM , Leaf PJ , Tischler GL , et al. Affective disorders in five United States communities. Psychol Med. 1988;18:141‐153. doi:10.1017/S0033291700001975 3363034

[273] Kessler RC , McGonagle KA , Swartz M , Blazer DG , Nelson CB . Sex and depression in the National Comorbidity Survey I: lifetime prevalence, chronicity and recurrence. J Affective Disord. 1993;29:85‐96. doi:10.1016/0165-0327(93)90026-G 8300981

[274] Moutinho S . Women twice as likely to develop Alzheimer's disease as men — but scientists do not know why. Nat Med. 2025;31:704‐707. doi:10.1038/s41591-025-03564-3 40087515

[275] Ownby RL , Crocco E , Acevedo A , John V , Loewenstein D . Depression and risk for Alzheimer disease: systematic review, meta‐analysis, and metaregression analysis. Arch Gen Psychiatry. 2006;63:530‐538. doi:10.1001/archpsyc.63.5.530 16651510 PMC3530614

[276] Tateno A , Nogami T , Sakayori T , Yamamoto K , Okubo Y . Depression as a prodromal symptom of neurodegenerative diseases. J Nippon Med Sch. 2023;90:157‐164. doi:10.1272/jnms.JNMS.2023_90-216 37258256

[277] Sun X , Steffens DC , Au R , et al. Amyloid‐associated depression: a prodromal depression of Alzheimer disease?. Arch Gen Psychiatry. 2008;65:542‐550. doi:10.1001/archpsyc.65.5.542 18458206 PMC3042807

[278] Wingo TS , Gerasimov ES , Canon SM , Lah JJ , Levey AI , Wingo AP . Alzheimer's disease genetic burden is associated with mid‐life depression among persons with normal cognition. Alzheimer's Dementia. 2023;19:868‐874. doi:10.1002/alz.12716 PMC9768095 35727298

[279] Berger M , Gray JA , Roth BL . The expanded biology of serotonin. Annu Rev Med. 2009;60:355‐366. doi:10.1146/annurev.med.60.042307.110802 19630576 PMC5864293

[280] Nishizawa S , Benkelfat C , Young SN , et al. Differences between males and females in rates of serotonin synthesis in human brain. Proc Natl Acad Sci. 1997;94:5308‐5313. doi:10.1073/pnas.94.10.5308 9144233 PMC24674

[281] Markova TZ , Ciampa CJ , Parent JH , et al. Poorer aging trajectories are associated with elevated serotonin synthesis capacity. Mol Psychiatry. 2023;28:4390‐4398. doi:10.1038/s41380-023-02177-x 37460847 PMC10792105

[282] Young SN , Gauthier S , Anderson GM , Purdy WC . Tryptophan, 5‐hydroxyindoleacetic acid and indoleacetic acid in human cerebrospinal fluid: interrelationships and the influence of age, sex, epilepsy and anticonvulsant drugs. J Neurol Neurosurg Psychiatry. 1980;43:438‐445. doi:10.1136/jnnp.43.5.438 6158559 PMC490572

[283] Haleem DJ , Kennett GA , Curzon G . Hippocampal 5‐hydroxytryptamine synthesis is greater in female rats than in males and more decreased by the 5‐HT1A agonist 8‐OH‐DPAT. J Neural Transmission. 1990;79:93‐101. doi:10.1007/BF01251004 1688708

[284] Carlsson M , Carlsson A . A regional study of sex differences in rat brain serotonin. Prog Neuro‐Psychopharmacol Biol Psychiatry. 1988;12:53‐61. doi:10.1016/0278-5846(88)90061-9 2452455

[285] Rosecrans JA . Differences in brain area 5‐hydroxytryptamine turnover and rearing behavior in rats and mice of both sexes. Eur J Pharmacol. 1970;9:379‐382. doi:10.1016/0014-2999(70)90239-6 5440309

[286] Klink R , Robichaud M , Debonnel G . Gender and gonadal status modulation of dorsal raphe nucleus serotonergic neurons. Part I: effects of gender and pregnancy. Neuropharmacology. 2002;43:1119‐1128. doi:10.1016/S0028-3908(02)00219-8 12504918

[287] Merchenthaler I , Lane MV , Numan S , Dellovade TL . Distribution of estrogen receptor alpha and beta in the mouse central nervous system: in vivo autoradiographic and immunocytochemical analyses. J Comp Neurol. 2004;473:270‐291. doi:10.1002/cne.20128 15101093

[288] Torres Irizarry VC , Feng B , Yang X , et al. Estrogen signaling in the dorsal raphe regulates binge‐like drinking in mice. Transl Psychiatry. 2024;14:122. doi:10.1038/s41398-024-02821-2 38413577 PMC10899193

[289] Murakawa T , Kogure L , Hata K , et al. Estrous cycle‐dependent modulation of sexual receptivity in female mice by estrogen receptor beta‐expressing cells in the dorsal raphe nucleus. J Neurosci. 2024;44:e1137242024. doi:10.1523/JNEUROSCI.1137-24.2024 39299803 PMC11604141

[290] Lu H , Ozawa H , Nishi M , Ito T , Kawata M . Serotonergic neurones in the dorsal raphe nucleus that project into the medial preoptic area contain oestrogen receptor β. J Neuroendocrinol. 2001;13:839‐845. doi:10.1046/j.1365-2826.2001.00695.x 11679052

[291] Gundlah C , Lu NZ , Mirkes SJ , Bethea CL . Estrogen receptor beta (ERbeta) mRNA and protein in serotonin neurons of macaques. Brain Res Mol Brain Res. 2001;91:14‐22. doi:10.1016/s0169-328x(01)00108-5 11457488

[292] Donner N , Handa RJ . Estrogen receptor beta regulates the expression of tryptophan‐hydroxylase 2 mRNA within serotonergic neurons of the rat dorsal raphe nuclei. Neuroscience. 2009;163:705‐718. doi:10.1016/j.neuroscience.2009.06.046 19559077 PMC2740745

[293] Hiroi R , Handa RJ . Estrogen receptor‐β regulates human tryptophan hydroxylase‐2 through an estrogen response element in the 5’ untranslated region. J Neurochem. 2013;127:487‐495. doi:10.1111/jnc.12401 24033289 PMC5825233

[294] Imwalle DB , Gustafsson J‐A , Rissman EF . Lack of functional estrogen receptor beta influences anxiety behavior and serotonin content in female mice. Physiol Behav. 2005;84:157‐163. doi:10.1016/j.physbeh.2004.11.002 15642619

[295] He J , Yan J‐J , Zha X , et al. Sexually dimorphic effects of estrogen receptor 2 deletion in the dorsal raphe nucleus on emotional behaviors. J Neuroendocrinol. 2023;35:e13195. doi:10.1111/jne.13195 36072992

[296] Holschneider DP , Kumazawa T , Chen K , Shih JC . Tissue‐specific effects of estrogen on monoamine oxidase A and B in the rat. Life Sci. 1998;63:155‐160. doi:10.1016/s0024-3205(98)00255-0 9698044

[297] Cao X , Xu P , Oyola MG , et al. Estrogens stimulate serotonin neurons to inhibit binge‐like eating in mice. J Clin Invest. 2014;124:4351‐4362. doi:10.1172/JCI74726 25157819 PMC4191033

[298] Sheng Z , Kawano J , Yanai A , et al. Expression of estrogen receptors (alpha, beta) and androgen receptor in serotonin neurons of the rat and mouse dorsal raphe nuclei; sex and species differences. Neurosci Res. 2004;49:185‐196. doi:10.1016/j.neures.2004.02.011 15140561

[299] Borisova NA , Proshlyakova EV , Sapronova AY , Ugrumov MV . Androgen‐dependent sex differences in the hypothalamic serotoninergic system. Eur J Endocrinol. 1996;134:232‐235. doi:10.1530/eje.0.1340232 8630525

[300] Biver F , Lotstra F , Monclus M , et al. Sex difference in 5HT2 receptor in the living human brain. Neurosci Lett. 1996;204:25‐28. doi:10.1016/0304-3940(96)12307-7 8929969

[301] Zhang L , Ma W , Barker JL , Rubinow DR . Sex differences in expression of serotonin receptors (subtypes 1A and 2A) in rat brain: a possible role of testosterone. Neuroscience. 1999;94:251‐259. doi:10.1016/S0306-4522(99)00234-1 10613515

[302] Goel N , Innala L , Viau V . Sex differences in serotonin (5‐HT) 1A receptor regulation of HPA axis and dorsal raphe responses to acute restraint. Psychoneuroendocrinology. 2014;40:232‐241. doi:10.1016/j.psyneuen.2013.11.020 24485495

[303] Philippe TJ , Bao L , Koblanski ME , Viau V . Sex differences in serotonin 5‐HT 1A receptor responses to repeated restraint stress in adult male and female rats. Int J Neuropsychopharmacol. 2022;25:863‐876. doi:10.1093/ijnp/pyac046 35904324 PMC9593217

[304] Fink G , Sumner BEH , Rosie R , Grace O , Quinn JP . Estrogen control of central neurotransmission: effect on mood, mental state, and memory. Cell Mol Neurobiol. 1996;16:325‐344. doi:10.1007/BF02088099 8818400 PMC11563142

[305] Sumner BEH , Fink G . Estrogen increases the density of 5‐Hydroxytryptamine2A receptors in cerebral cortex and nucleus accumbens in the female rat. J Steroid Biochem Mol Biol. 1995;54:15‐20. doi:10.1016/0960-0760(95)00075-B 7632610

[306] Biegon A , McEwen BS . Modulation by estradiol of serotonin receptors in brain. J Neurosci. 1982;2:199‐205. doi:10.1523/JNEUROSCI.02-02-00199.1982 7199565 PMC6564299

[307] Kugaya A , Epperson CN , Zoghbi S , et al. Increase in prefrontal cortex serotonin 2A receptors following estrogen treatment in postmenopausal women. Am J Psychiatry. 2003;160:1522‐1524. doi:10.1176/appi.ajp.160.8.1522 12900319

[308] Uphouse L , Williams J , Eckols K , Sierra V . Variations in binding of [3H]5‐HT to cortical membranes during the female rat estrous cycle. Brain Res. 1986;381:376‐381. doi:10.1016/0006-8993(86)90093-4 3756512

[309] Sumner BEH , Fink G . Effects of acute estradiol on 5‐hydroxytryptamine and dopamine receptor subtype mRNA expression in female rat brain. Mol Cellular Neurosci. 1993;4:83‐92. doi:10.1006/mcne.1993.1010 19912911

[310] Bertrand PP , Paranavitane UT , Chavez C , Gogos A , Jones M , van den Buuse M . The effect of low estrogen state on serotonin transporter function in mouse hippocampus: a behavioral and electrochemical study. Brain Res. 2005;1064:10‐20. doi:10.1016/j.brainres.2005.10.018 16298349

[311] McQueen JK , Wilson H , Fink G . Estradiol‐17β increase serotonin transporter (SERT) mRNA levels and the density of SERT‐binding sites in female rat brain. Mol Brain Res. 1997;45:13‐23. doi:10.1016/S0169-328X(96)00233-1 9105666

[312] Smith GS , Barrett FS , Joo JH , et al. Molecular imaging of serotonin degeneration in mild cognitive impairment. Neurobiol Dis. 2017;105:33‐41. doi:10.1016/j.nbd.2017.05.007 28511918 PMC5663212

[313] Thomas AJ , Hendriksen M , Piggott M , et al. A study of the serotonin transporter in the prefrontal cortex in late‐life depression and Alzheimer's disease with and without depression. Neuropathol Appl Neurobiol. 2006;32:296‐303. doi:10.1111/j.1365-2990.2006.00728.x 16640648

[314] Tejani‐Butt SM , Yang J , Pawlyk AC . Altered serotonin transporter sites in Alzheimer's disease raphe and hippocampus. Neuroreport. 1995;6:1207‐1210. doi:10.1097/00001756-199505300-00033 7662909

[315] Ouchi Y , Yoshikawa E , Futatsubashi M , Yagi S , Ueki T , Nakamura K . Altered brain serotonin transporter and associated glucose metabolism in Alzheimer disease. J Nucl Med. 2009;50:1260‐1266. doi:10.2967/jnumed.109.063008 19617327

[316] Bethea CL , Smith AW , Centeno ML , Reddy AP . Long‐term ovariectomy decreases serotonin neuron number and gene expression in free ranging macaques. Neuroscience. 2011;192:675‐688. doi:10.1016/j.neuroscience.2011.06.003 21763405 PMC3166449

[317] Huang Y‐Y , Gan Y‐H , Yang L , Cheng W , Yu J‐T . Depression in Alzheimer's disease: epidemiology, mechanisms, and treatment. Biol Psychiatry. 2024;95:992‐1005. doi:10.1016/j.biopsych.2023.10.008 37866486

[318] Cirrito JR , Disabato BM , Restivo JL , et al. Serotonin signaling is associated with lower amyloid‐β levels and plaques in transgenic mice and humans. Proc Natl Acad Sci. 2011;108:14968‐14973. doi:10.1073/pnas.1107411108 21873225 PMC3169155

[319] Sheline YI , Snider BJ , Beer JC , et al. Effect of escitalopram dose and treatment duration on CSF Aβ levels in healthy older adults: a controlled clinical trial. Neurology. 2020;95:e2658‐65. doi:10.1212/WNL.0000000000010725 32913021 PMC7713735

[320] Mdawar B , Ghossoub E , Khoury R . Selective serotonin reuptake inhibitors and Alzheimer's disease. Neural Regen Res. 2020;15:41‐46. doi:10.4103/1673-5374.264445 31535641 PMC6862425

[321] Sepehry AA , Lee PE , Hsiung GYR , Beattie BL , Jacova C . Effect of selective serotonin reuptake inhibitors in Alzheimer's disease with comorbid depression: a meta‐analysis of depression and cognitive outcomes. Drugs Aging. 2012;29:793‐806. doi:10.1007/s40266-012-0012-5 23079957

[322] Wang Y‐C , Tai P‐A , Poly TN , et al. Increased risk of dementia in patients with antidepressants: a meta‐analysis of observational studies. Behav Neurol. 2018;2018:5315098. doi:10.1155/2018/5315098 30123386 PMC6079596

[323] Jones HE , Joshi A , Shenkin S , Mead GE . The effect of treatment with selective serotonin reuptake inhibitors in comparison to placebo in the progression of dementia: a systematic review and meta‐analysis. Age Ageing. 2016;45:448‐456. doi:10.1093/ageing/afw053 27055878

[324] Terstege DJ , Jabeen S , Alzheimer's Disease Neuroimaging Initiative , Galea LAM , Epp JR , Sargin D , Alzheimer's Disease Neuroimaging Initiative . SSRIs reduce plasma tau and restore dorsal raphe metabolism in Alzheimer's disease. Alzheimers Dement. 2025;21:e14579. doi:10.1002/alz.14579 39935329 PMC11814539

[325] Mo M , Abzhandadze T , Hoang MT , et al. Antidepressant use and cognitive decline in patients with dementia: a national cohort study. BMC Med. 2025;23:82. doi:10.1186/s12916-025-03851-3 39994788 PMC11854023

[326] Xie Y , Liu P‐P , Lian Y‐J , Liu H‐B , Kang J‐S . The effect of selective serotonin reuptake inhibitors on cognitive function in patients with Alzheimer's disease and vascular dementia: focusing on fluoxetine with long follow‐up periods. Sig Transduct Target Ther. 2019;4:30. doi:10.1038/s41392-019-0064-7 PMC6799811 31637010

[327] Breitinger H‐GA , Geetha N , Hess GP . Inhibition of the serotonin 5‐HT3 receptor by nicotine, cocaine, and fluoxetine investigated by rapid chemical kinetic techniques. Biochemistry. 2001;40:8419‐8429. doi:10.1021/bi0106890 11444989

[328] Pälvimäki E‐P , Majasuo H , Laakso A , et al. Interactions of selective serotonin reuptake inhibitors with the serotonin 5‐HT2C receptor. Psychopharmacology. 1996;126:234‐240. doi:10.1007/BF02246453 8876023

[329] Castañé A , Kargieman L , Celada P , Bortolozzi A , Artigas F . 5‐HT2A receptors are involved in cognitive but not antidepressant effects of fluoxetine. Eur Neuropsychopharmacol. 2015;25:1353‐1361. doi:10.1016/j.euroneuro.2015.04.006 25914158

[330] Mosconi L , Berti V , Guyara‐Quinn C , et al. Perimenopause and emergence of an Alzheimer's bioenergetic phenotype in brain and periphery. PLOS ONE. 2017;12:e0185926. doi:10.1371/journal.pone.0185926 29016679 PMC5634623

[331] Mosconi L , Nerattini M , Matthews DC , et al. In vivo brain estrogen receptor density by neuroendocrine aging and relationships with cognition and symptomatology. Sci Rep. 2024;14:12680. doi:10.1038/s41598-024-62820-7 38902275 PMC11190148

[332] Long J , He P , Shen Y , Li R . New evidence of mitochondria dysfunction in the female Alzheimer's disease brain: deficiency of estrogen receptor‐β. J Alzheimers Dis. 2012;30:545‐558. doi:10.3233/JAD-2012-120283 22451324 PMC3506431

[333] Fanibunda SE , Deb S , Maniyadath B , et al. Serotonin regulates mitochondrial biogenesis and function in rodent cortical neurons via the 5‐HT2A receptor and SIRT1‐PGC‐1α axis. Proc Natl Acad Sci U S A. 2019;116:11028‐11037. doi:10.1073/pnas.1821332116 31072928 PMC6561197

[334] Tian J , Stucky CS , Wang T , Muma NA , Johnson M , Du H . Mitochondrial dysfunction links to impaired hippocampal serotonin release in a mouse model of Alzheimer's disease. J Alzheimers Dis. 2023;93:605‐619. doi:10.3233/JAD-230072 37066917 PMC10416312

[335] Mosconi L , Berti V , Dyke J , et al. Menopause impacts human brain structure, connectivity, energy metabolism, and amyloid‐beta deposition. Sci Rep. 2021;11:10867. doi:10.1038/s41598-021-90084-y 34108509 PMC8190071

[336] Waters EM , Yildirim M , Janssen WGM , et al. Estrogen and aging affect the synaptic distribution of estrogen receptor beta‐immunoreactivity in the CA1 region of female rat hippocampus. Brain Research. 2011;1379:86‐97. doi:10.1016/j.brainres.2010.09.069 20875808 PMC3046233

[337] Pierson SR , Fiock KL , Wang R , et al. Tau pathology in the dorsal raphe may be a prodromal indicator of Alzheimer's disease. Mol Psychiatry. 2025;30:532‐546. doi:10.1038/s41380-024-02664-9 39143322 PMC12010729

[338] Khan NS , Uribe Isaza J , Rouhi N , et al. Behavioral and neurophysiological implications of pathological human tau expression in serotonin neurons. ACS Chem Neurosci. 2024;15:932‐943. doi:10.1021/acschemneuro.3c00626 38377680 PMC10921395

[339] Chen M , Wang C , Lin Y , et al. Dorsal raphe nucleus‐hippocampus serotonergic circuit underlies the depressive and cognitive impairments in 5×FAD male mice. Transl Neurodegener. 2024;13:34. doi:10.1186/s40035-024-00425-w 39044270 PMC11267773

[340] Wang J , Mei Y , Zhang X , et al. Aberrant serotonergic signaling contributes to the hyperexcitability of CA1 pyramidal neurons in a mouse model of Alzheimer's disease. Cell Rep. 2023;42:112152. doi:10.1016/j.celrep.2023.112152 36821438

[341] Barth C , Crestol A , de Lange A‐MG , Galea LAM . Sex steroids and the female brain across the lifespan: insights into risk of depression and Alzheimer's disease. Lancet Diabetes Endocrinol. 2023;11:926‐941. doi:10.1016/S2213-8587(23)00224-3 37865102

[342] Canet G , Hernandez C , Zussy C , Chevallier N , Desrumaux C , Givalois L . Is AD a stress‐related disorder? Focus on the HPA axis and its promising therapeutic targets. Front Aging Neurosci. 2019;11:269. doi:10.3389/fnagi.2019.00269 31611783 PMC6776918

[343] Zawia NH , Basha MR . Environmental risk factors and the developmental basis for Alzheimer's disease. Rev Neurosci. 2005;16:325‐337. doi:10.1515/revneuro.2005.16.4.325 16519009

[344] Caruso A , Nicoletti F , Gaetano A , Scaccianoce S . Risk Factors for Alzheimer's Disease: focus on Stress. Front Pharmacol. 2019;10:976. doi:10.3389/fphar.2019.00976 31551781 PMC6746823

[345] Justice NJ . The relationship between stress and Alzheimer's disease. Neurobiol Stress. 2018;8:127‐133. doi:10.1016/j.ynstr.2018.04.002 29888308 PMC5991350

[346] Reyna NC , Clark BJ , Hamilton DA , Pentkowski NS . Anxiety and Alzheimer's disease pathogenesis: focus on 5‐HT and CRF systems in 3xTg‐AD and TgF344‐AD animal models. Front Aging Neurosci. 2023;15:1251075. doi:10.3389/fnagi.2023.1251075 38076543 PMC10699143

[347] Vandael D , Gounko NV . Corticotropin releasing factor‐binding protein (CRF‐BP) as a potential new therapeutic target in Alzheimer's disease and stress disorders. Transl Psychiatry. 2019;9:272. doi:10.1038/s41398-019-0581-8 31641098 PMC6805916

[348] Vale W , Spiess J , Rivier C , Rivier J . Characterization of a 41‐residue ovine hypothalamic peptide that stimulates secretion of corticotropin and beta‐endorphin. Science. 1981;213:1394‐1397. doi:10.1126/science.6267699 6267699

[349] Flandreau EI , Ressler KJ , Owens MJ , Nemeroff CB . Chronic overexpression of corticotropin‐releasing factor from the central amygdala produces HPA axis hyperactivity and behavioral anxiety associated with gene‐expression changes in the hippocampus and paraventricular nucleus of the hypothalamus. Psychoneuroendocrinology. 2012;37:27‐38. doi:10.1016/j.psyneuen.2011.04.014 21616602 PMC3164918

[350] Kennis M , Gerritsen L , van Dalen M , Williams A , Cuijpers P , Bockting C . Prospective biomarkers of major depressive disorder: a systematic review and meta‐analysis. Mol Psychiatry. 2020;25:321‐338. doi:10.1038/s41380-019-0585-z 31745238 PMC6974432

[351] Piccirillo G , Fimognari FL , Infantino V , et al. High plasma concentrations of cortisol and thromboxane B2 in patients with depression. Am J Med Sci. 1994;307:228‐232. doi:10.1097/00000441-199403000-00011 8160715

[352] Łoś K , Waszkiewicz N . Biological markers in anxiety disorders. J Clin Med. 2021;10:1744. doi:10.3390/jcm10081744 33920547 PMC8073190

[353] Csernansky JG , Dong H , Fagan AM , et al. Plasma cortisol and progression of dementia in subjects with Alzheimer‐type dementia. Am J Psychiatry. 2006;163:2164‐2169. doi:10.1176/ajp.2006.163.12.2164 17151169 PMC1780275

[354] Popp J , Wolfsgruber S , Heuser I , et al. Cerebrospinal fluid cortisol and clinical disease progression in MCI and dementia of Alzheimer's type. Neurobiol Aging. 2015;36:601‐607. doi:10.1016/j.neurobiolaging.2014.10.031 25435336

[355] Hatzinger M , Z'Brun A , Hemmeter U , et al. Hypothalamic‐pituitary‐adrenal system function in patients with Alzheimer's disease. Neurobiol Aging. 1995;16:205‐209. doi:10.1016/0197-4580(94)00159-6 7777138

[356] Davis KL , Davis BM , Greenwald BS , et al. Cortisol and Alzheimer's disease, I: basal studies. Am J Psychiatry. 1986;143:300‐305. doi:10.1176/ajp.143.3.300 3953862

[357] Hartmann A , Veldhuis JD , Deuschle M , Standhardt H , Heuser I . Twenty‐four hour cortisol release profiles in patients with Alzheimer's and Parkinson's disease compared to normal controls: ultradian secretory pulsatility and diurnal variation. Neurobiol Aging. 1997;18:285‐289. doi:10.1016/s0197-4580(97)80309-0 9263193

[358] Huang C‐W , Lui C‐C , Chang W‐N , Lu C‐H , Wang Y‐L , Chang C‐C . Elevated basal cortisol level predicts lower hippocampal volume and cognitive decline in Alzheimer's disease. J Clin Neurosci. 2009;16:1283‐1286. doi:10.1016/j.jocn.2008.12.026 19570680

[359] De Souza EB , Whitehouse PJ , Kuhar MJ , Price DL , Vale WW . Reciprocal changes in corticotropin‐releasing factor (CRF)‐like immunoreactivity and CRF receptors in cerebral cortex of Alzheimer's disease. Nature. 1986;319:593‐595. doi:10.1038/319593a0 3003585

[360] Whitehouse PJ , Vale WW , Zweig RM , et al. Reductions in corticotropin releasing factor‐like immunoreactivity in cerebral cortex in Alzheimer's disease, Parkinson's disease, and progressive supranuclear palsy. Neurology. 1987;37:905‐909. doi:10.1212/wnl.37.6.905 3495748

[361] Pomara N , Singh RR , Deptula D , et al. CSF corticotropin‐releasing factor (CRF) in Alzheimer's disease: its relationship to severity of dementia and monoamine metabolites. Biol Psychiatry. 1989;26:500‐504. doi:10.1016/0006-3223(89)90071-1 2477071

[362] Lupien SJ , de Leon M , de Santi S , et al. Cortisol levels during human aging predict hippocampal atrophy and memory deficits. Nat Neurosci. 1998;1:69‐73. doi:10.1038/271 10195112

[363] Lind K , Edman A , Nordlund A , Olsson T , Wallin A . Increased saliva cortisol awakening response in patients with mild cognitive impairment. Dement Geriatr Cogn Disord. 2007;24:389‐395. doi:10.1159/000109938 17943022

[364] Guo Q , Zheng H , Justice NJ . Central CRF system perturbation in an Alzheimer's disease knockin mouse model. Neurobiol Aging. 2012;33:2678‐2691. doi:10.1016/j.neurobiolaging.2012.01.002 22336193 PMC3361634

[365] Brureau A , Zussy C , Delair B , et al. Deregulation of hypothalamic‐pituitary‐adrenal axis functions in an Alzheimer's disease rat model. Neurobiol Aging. 2013;34:1426‐1439. doi:10.1016/j.neurobiolaging.2012.11.015 23273603

[366] Justice NJ , Huang L , Tian J‐B , et al. Posttraumatic stress disorder‐like induction elevates β‐amyloid levels, which directly activates corticotropin‐releasing factor neurons to exacerbate stress responses. J Neurosci. 2015;35:2612‐2623. doi:10.1523/JNEUROSCI.3333-14.2015 25673853 PMC4323535

[367] Dong H , Goico B , Martin M , Csernansky CA , Bertchume A , Csernansky JG . Modulation of hippocampal cell proliferation, memory, and amyloid plaque deposition in APPsw (Tg2576) mutant mice by isolation stress. Neuroscience. 2004;127:601‐609. doi:10.1016/j.neuroscience.2004.05.040 15283960

[368] Dong H , Murphy KM , Meng L , et al. Corticotrophin releasing factor accelerates neuropathology and cognitive decline in a mouse model of Alzheimer's disease. J Alzheimers Dis. 2012;28:579‐592. doi:10.3233/JAD-2011-111328 22045495 PMC3494090

[369] Dong H , Wang S , Zeng Z , et al. Effects of corticotrophin‐releasing factor receptor 1 antagonists on amyloid‐β and behavior in Tg2576 mice. Psychopharmacology (Berl). 2014;231:4711‐4722. doi:10.1007/s00213-014-3629-8 24862368 PMC4233002

[370] Kang J‐E , Cirrito JR , Dong H , Csernansky JG , Holtzman DM . Acute stress increases interstitial fluid amyloid‐beta via corticotropin‐releasing factor and neuronal activity. Proc Natl Acad Sci USA. 2007;104:10673‐10678. doi:10.1073/pnas.0700148104 17551018 PMC1965571

[371] Zhang C , Kuo C‐C , Moghadam SH , et al. Corticotropin‐releasing factor receptor‐1 antagonism mitigates beta amyloid pathology and cognitive and synaptic deficits in a mouse model of Alzheimer's disease. Alzheimers Dement. 2016;12:527‐537. doi:10.1016/j.jalz.2015.09.007 26555315 PMC4860182

[372] Rissman RA , Staup MA , Lee AR , et al. Corticotropin‐releasing factor receptor‐dependent effects of repeated stress on tau phosphorylation, solubility, and aggregation. Proc Natl Acad Sci USA. 2012;109:6277‐6282. doi:10.1073/pnas.1203140109 22451915 PMC3341026

[373] Rissman RA , Lee K‐F , Vale W , Sawchenko PE . Corticotropin‐releasing factor receptors differentially regulate stress‐induced tau phosphorylation. J Neurosci. 2007;27:6552‐6562. doi:10.1523/JNEUROSCI.5173-06.2007 17567816 PMC6672442

[374] Carroll JC , Iba M , Bangasser DA , et al. Chronic stress exacerbates tau pathology, neurodegeneration, and cognitive performance through a corticotropin‐releasing factor receptor‐dependent mechanism in a transgenic mouse model of tauopathy. J Neurosci. 2011;31:14436‐14449. doi:10.1523/JNEUROSCI.3836-11.2011 21976528 PMC3230070

[375] Campbell SN , Zhang C , Roe AD , et al. Impact of CRFR1 ablation on amyloid‐β production and accumulation in a mouse model of Alzheimer's disease. J Alzheimers Dis. 2015;45:1175‐1184. doi:10.3233/JAD-142844 25697705 PMC4459491

[376] Campbell SN , Zhang C , Monte L , et al. Increased tau phosphorylation and aggregation in the hippocampus of mice overexpressing corticotropin‐releasing factor. J Alzheimers Dis. 2015;43:967‐976. doi:10.3233/JAD-141281 25125464 PMC4258165

[377] Cohen RM , Rezai‐Zadeh K , Weitz TM , et al. A transgenic Alzheimer rat with plaques, tau pathology, behavioral impairment, oligomeric aβ, and frank neuronal loss. J Neurosci. 2013;33:6245‐6256. doi:10.1523/JNEUROSCI.3672-12.2013 23575824 PMC3720142

[378] Frederiksen SO , Ekman R , Gottfries CG , Widerlöv E , Jonsson S . Reduced concentrations of galanin, arginine vasopressin, neuropeptide Y and peptide YY in the temporal cortex but not in the hypothalamus of brains from schizophrenics. Acta Psychiatr Scand. 1991;83:273‐277. doi:10.1111/j.1600-0447.1991.tb05539.x 1709331

[379] Viau V , Bingham B , Davis J , Lee P , Wong M . Gender and puberty interact on the stress‐induced activation of parvocellular neurosecretory neurons and corticotropin‐releasing hormone messenger ribonucleic acid expression in the rat. Endocrinology. 2005;146:137‐146. doi:10.1210/en.2004-0846 15375029

[380] Iwasaki‐Sekino A , Mano‐Otagiri A , Ohata H , Yamauchi N , Shibasaki T . Gender differences in corticotropin and corticosterone secretion and corticotropin‐releasing factor mRNA expression in the paraventricular nucleus of the hypothalamus and the central nucleus of the amygdala in response to footshock stress or psychological stress in rats. Psychoneuroendocrinology. 2009;34:226‐237. doi:10.1016/j.psyneuen.2008.09.003 18849120

[381] Corticotropin‐releasing hormone mRNA levels in response to chronic mild stress rise in male but not in female rats while tyrosine hydroxylase mRNA levels decrease in both sexes. Portal de Periódicos da CAPES n.d. Accessed September 1, 2025. https://www.periodicos.capes.gov.br/index.php/acervo/buscador.html?task=detalhes&id=W2101334425 10.1016/s0306-4530(00)00040-8 11070336

[382] Seale JV , Wood SA , Atkinson HC , et al. Gonadectomy reverses the sexually diergic patterns of circadian and stress‐induced hypothalamic‐pituitary‐adrenal axis activity in male and female rats. J Neuroendocrinol. 2004;16:516‐524. doi:10.1111/j.1365-2826.2004.01195.x 15189326

[383] Li K , Nakajima M , Ibañez‐Tallon I , Heintz N . A cortical circuit for sexually dimorphic oxytocin‐dependent anxiety behaviors. Cell. 2016;167:60‐72.e11. doi:10.1016/j.cell.2016.08.067 27641503 PMC5220951

[384] Handa RJ , Burgess LH , Kerr JE , O'Keefe JA . Gonadal steroid hormone receptors and sex differences in the hypothalamo‐pituitary‐adrenal axis. Horm Behav. 1994;28:464‐476. doi:10.1006/hbeh.1994.1044 7729815

[385] Kitay JI . Sex differences in adrenal cortical secretion in the rat. Endocrinology. 1961;68:818‐824. doi:10.1210/endo-68-5-818 13756461

[386] Bangasser DA , Wicks B . Sex‐specific mechanisms for responding to stress. J Neurosci Res. 2017;95:75‐82. doi:10.1002/jnr.23812 27870416 PMC5120612

[387] Chisari A , Carino M , Perone M , Gaillard RC , Spinedi E . Sex and strain variability in the rat hypothalamo‐pituitary‐adrenal (HPA) axis function. J Endocrinol Invest. 1995;18:25‐33. doi:10.1007/BF03349692 7759781

[388] Atkinson HC , Waddell BJ . Circadian variation in basal plasma corticosterone and adrenocorticotropin in the rat: sexual dimorphism and changes across the estrous cycle. Endocrinology. 1997;138:3842‐3848. doi:10.1210/endo.138.9.5395 9275073

[389] Locci A , Yan Y , Rodriguez G , Dong H . Sex differences in CRF1, CRF, and CRFBP expression in C57BL/6J mouse brain across the lifespan and in response to acute stress. J Neurochem. 2021;158:943‐959. doi:10.1111/jnc.15157 32813270 PMC9811412

[390] Yan Y , Dominguez S , Fisher DW , Dong H . Sex differences in chronic stress responses and Alzheimer's disease. Neurobiol Stress. 2018;8:120‐126. doi:10.1016/j.ynstr.2018.03.002 29888307 PMC5991323

[391] Stroud LR , Papandonatos GD , Williamson DE , Dahl RE . Sex differences in cortisol response to corticotropin releasing hormone challenge over puberty: Pittsburgh pediatric neurobehavioral studies. Psychoneuroendocrinology. 2011;36:1226‐1238. doi:10.1016/j.psyneuen.2011.02.017 21489699 PMC3270708

[392] Seeman TE , Singer B , Wilkinson CW , McEwen B . Gender differences in age‐related changes in HPA axis reactivity. Psychoneuroendocrinology. 2001;26:225‐240. doi:10.1016/s0306-4530(00)00043-3 11166486

[393] Babb JA , Masini CV , Day HEW , Campeau S . Sex differences in activated corticotropin‐releasing factor neurons within stress‐related neurocircuitry and hypothalamic‐pituitary‐adrenocortical axis hormones following restraint in rats. Neuroscience. 2013;234:40‐52. doi:10.1016/j.neuroscience.2012.12.051 23305762 PMC3594441

[394] Gallucci WT , Baum A , Laue L , et al. Sex differences in sensitivity of the hypothalamic‐pituitary‐adrenal axis. Health Psychol. 1993;12:420‐425. doi:10.1037//0278-6133.12.5.420 8223368

[395] Otte C , Hart S , Neylan TC , Marmar CR , Yaffe K , Mohr DC . A meta‐analysis of cortisol response to challenge in human aging: importance of gender. Psychoneuroendocrinology. 2005;30:80‐91. doi:10.1016/j.psyneuen.2004.06.002 15358445

[396] Kudielka BM , Kirschbaum C . Sex differences in HPA axis responses to stress: a review. Biol Psychol. 2005;69:113‐132. doi:10.1016/j.biopsycho.2004.11.009 15740829

[397] Justice NJ , Yuan ZF , Sawchenko PE , Vale W . Type 1 corticotropin‐releasing factor receptor expression reported in BAC transgenic mice: implications for reconciling ligand‐receptor mismatch in the central corticotropin‐releasing factor system. J Comp Neurol. 2008;511:479‐496. doi:10.1002/cne.21848 18853426 PMC2597626

[398] Rosinger ZJ , Jacobskind JS , De Guzman RM , Justice NJ , Zuloaga DG . A sexually dimorphic distribution of corticotropin‐releasing factor receptor 1 in the paraventricular hypothalamus. Neuroscience. 2019;409:195‐203. doi:10.1016/j.neuroscience.2019.04.045 31055007 PMC6897333

[399] Rosinger ZJ , Jacobskind JS , Bulanchuk N , et al. Characterization and gonadal hormone regulation of a sexually dimorphic corticotropin‐releasing factor receptor 1 cell group. J Comp Neurol. 2019;527:1056‐1069. doi:10.1002/cne.24588 30499109 PMC6857540

[400] Rosinger ZJ , De Guzman RM , Jacobskind JS , et al. Sex‐dependent effects of chronic variable stress on discrete corticotropin‐releasing factor receptor 1 cell populations. Physiol Behav. 2020;219:112847. doi:10.1016/j.physbeh.2020.112847 32081812 PMC7540729

[401] De Guzman RM , Rosinger ZJ , Parra KE , Jacobskind JS , Justice NJ , Zuloaga DG . Alterations in corticotropin‐releasing factor receptor type 1 in the preoptic area and hypothalamus in mice during the postpartum period. Horm Behav. 2021;135:105044. doi:10.1016/j.yhbeh.2021.105044 34507241 PMC8653990

[402] Horgan J , Miguel‐Hidalgo JJ , Thrasher M , Bissette G . Longitudinal brain corticotropin releasing factor and somatostatin in a transgenic mouse (TG2576) model of Alzheimer's disease. J Alzheimers Dis. 2007;12:115‐127. doi:10.3233/jad-2007-12201 17917156 PMC2919580

[403] Lv J , Chen L , Zhu N , et al. Beta amyloid‐induced time‐dependent learning and memory impairment: involvement of HPA axis dysfunction. Metab Brain Dis. 2020;35:1385‐1394. doi:10.1007/s11011-020-00613-3 32860609

[404] Steinmetz D , Ramos E , Campbell SN , Morales T , Rissman RA . Reproductive stage and modulation of stress‐induced tau phosphorylation in female rats. J Neuroendocrinol. 2015;27:827‐834. doi:10.1111/jne.12323 26510116 PMC4625411

[405] Dominguez S , Rodriguez G , Fazelinia H , et al. Sex differences in the phosphoproteomic profiles of APP/PS1 mice after chronic unpredictable mild stress. J Alzheimers Dis. 2020;74:1131‐1142. doi:10.3233/JAD-191009 32144982 PMC9843707

[406] Sil A , Erfani A , Lamb N , Copland R , Riedel G , Platt B . Sex differences in behavior and molecular pathology in the 5XFAD model. J Alzheimers Dis. 2022;85:755‐778. doi:10.3233/JAD-210523 34864660

[407] Nguyen ET , Selmanovic D , Maltry M , et al. Endocrine stress responsivity and social memory in 3xTg‐AD female and male mice: a tale of two experiments. Horm Behav. 2020;126:104852. doi:10.1016/j.yhbeh.2020.104852 32949555

[408] Edwards HM , Wallace CE , Gardiner WD , et al. Sex‐dependent effects of acute stress on amyloid‐β in male and female mice. Brain. 2023;146:2268‐2274. doi:10.1093/brain/awad052 37127299 PMC10232275

[409] Murphy KJ , Hodges TE , Sheppard PAS , Troyer AK , Hampson E , Galea LAM . Sex differences in cortisol and memory following acute social stress in amnestic mild cognitive impairment. J Clin Exp Neuropsychol. 2020;42:881‐901. doi:10.1080/13803395.2020.1825633 33023371

[410] Dong H , Keegan JM , Hong E , et al. Corticotrophin releasing factor receptor 1 antagonists prevent chronic stress‐induced behavioral changes and synapse loss in aged rats. Psychoneuroendocrinology. 2018;90:92‐101. doi:10.1016/j.psyneuen.2018.02.013 29477954 PMC5864558

[411] Hebda‐Bauer EK , Simmons TA , Sugg A , et al. 3xTg‐AD mice exhibit an activated central stress axis during early‐stage pathology. J Alzheimers Dis. 2013;33:407‐422. doi:10.3233/JAD-2012-121438 22976078 PMC3525735

[412] Salardini A , Himali JJ , Abdullah MS , et al. Elevated serum cortisol associated with early‐detected increase of brain amyloid deposition in Alzheimer's disease imaging biomarkers among menopausal women: the Framingham Heart Study. Alzheimers Dement. 2025;21:e70179. doi:10.1002/alz.70179 40271551 PMC12019305

[413] Froemke RC , Young LJ . Oxytocin, Neural Plasticity, and Social Behavior. Annu Rev Neurosci. 2021;44:359‐381. doi:10.1146/annurev-neuro-102320-102847 33823654 PMC8604207

[414] Stoop R . Neuromodulation by oxytocin and vasopressin. Neuron. 2012;76:142‐159. doi:10.1016/j.neuron.2012.09.025 23040812

[415] Busnelli M , Chini B . Molecular basis of oxytocin receptor signalling in the brain: what we know and what we need to know. Curr Top Behav Neurosci. 2018;35:3‐29. doi:10.1007/7854_2017_6 28812263

[416] Quintana DS , Rokicki J , van der Meer D , et al. Oxytocin pathway gene networks in the human brain. Nat Commun. 2019;10:668. doi:10.1038/s41467-019-08503-8 30737392 PMC6368605

[417] Drinkwater E , Davies C , Spires‐Jones TL . Potential neurobiological links between social isolation and Alzheimer's disease risk. Eur J Neurosci. 2022;56:5397‐5412. doi:10.1111/ejn.15373 34184343

[418] Dumais KM , Veenema AH . Vasopressin and oxytocin receptor systems in the brain: sex differences and sex‐specific regulation of social behavior. Front Neuroendocrinol. 2016;40:1‐23. doi:10.1016/j.yfrne.2015.04.003 25951955 PMC4633405

[419] Caldwell HK . Oxytocin and sex differences in behavior. Curr Opin Behav Sci. 2018;23:13‐20. doi:10.1016/j.cobeha.2018.02.002

[420] Procyshyn TL , Dupertuys J , Bartz JA . Neuroimaging and behavioral evidence of sex‐specific effects of oxytocin on human sociality. Trends Cogn Sci. 2024;28:948‐961. doi:10.1016/j.tics.2024.06.010 39054193

[421] Lu Q , Hu S . Chapter 5 ‐ Sex differences of oxytocin and vasopressin in social behaviors. In: Swaab DF , Kreier F , Lucassen PJ , Salehi A , Buijs RM , eds. Handbook of Clinical Neurology. Elsevier; 2021:65‐88. doi:10.1016/B978-0-12-820107-7.00005-7 34225950

[422] Rosen GJ , de Vries GJ , Goldman SL , Goldman BD , Forger NG . Distribution of oxytocin in the brain of a eusocial rodent. Neuroscience. 2008;155:809‐817. doi:10.1016/j.neuroscience.2008.05.039 18582538 PMC2614305

[423] Wang Z , Zhou L , Hulihan TJ , Insel TR . Immunoreactivity of central vasopressin and oxytocin pathways in microtine rodents: a quantitative comparative study. J Comp Neurol. 1996;366:726‐737. doi:10.1002/(SICI)1096-9861(19960318)366:4<726::AID-CNE11>3.0.CO;2-D 8833119

[424] Xu L , Pan Y , Young KA , Wang Z , Zhang Z . Oxytocin and vasopressin immunoreactive staining in the brains of Brandt's voles (Lasiopodomys brandtii) and greater long‐tailed hamsters (Tscherskia triton). Neuroscience. 2010;169:1235‐1247. doi:10.1016/j.neuroscience.2010.05.064 20573572 PMC3680116

[425] Qiao X , Yan Y , Wu R , et al. Sociality and oxytocin and vasopressin in the brain of male and female dominant and subordinate mandarin voles. J Comp Physiol A Neuroethol Sens Neural Behav Physiol. 2014;200:149‐159. doi:10.1007/s00359-013-0870-2 24292210

[426] Wang Y , Xu L , Pan Y , Wang Z , Zhang Z . Species differences in the immunoreactive expression of oxytocin, vasopressin, tyrosine hydroxylase and estrogen receptor alpha in the brain of Mongolian Gerbils (Meriones unguiculatus) and Chinese striped hamsters (Cricetulus barabensis). PLOS ONE. 2013;8:e65807. doi:10.1371/journal.pone.0065807 23762431 PMC3676338

[427] Caffé AR , Van Ryen PC , Van der Woude TP , Van Leeuwen FW . Vasopressin and oxytocin systems in the brain and upper spinal cord of Macaca fascicularis. J Comp Neurol. 1989;287:302‐325. doi:10.1002/cne.902870304 2778107

[428] Wang Z , Moody K , Newman JD , Insel TR . Vasopressin and oxytocin immunoreactive neurons and fibers in the forebrain of male and female common marmosets (Callithrix jacchus). Synapse. 1997;27:14‐25. doi:10.1002/(SICI)1098-2396(199709)27:1<14::AID-SYN2>3.0.CO;2-G 9268061

[429] Fliers E , Swaab DF , Pool CW , Verwer RWH . The vasopressin and oxytocin neurons in the human supraoptic and paraventricular nucleus; changes with aging and in senile dementia. Brain Res. 1985;342:45‐53. doi:10.1016/0006-8993(85)91351-4 4041817

[430] Wierda M , Goudsmit E , Van Der Woude PF , et al. Oxytocin cell number in the human paraventricular nucleus remains constant with aging and in Alzheimer's disease. Neurobiol Aging. 1991;12:511‐516. doi:10.1016/0197-4580(91)90081-T 1770986

[431] Ishunina TA , Swaab DF . Vasopressin and oxytocin neurons of the human supraoptic and paraventricular nucleus; size changes in relation to age and sex. J Clin Endocrinol Metab. 1999;84:4637‐4644. doi:10.1210/jcem.84.12.6187 10599731

[432] Lim MM , Murphy AZ , Young LJ . Ventral striatopallidal oxytocin and vasopressin V1a receptors in the monogamous prairie vole (Microtus ochrogaster). J Comp Neurol. 2004;468:555‐570. doi:10.1002/cne.10973 14689486

[433] Dumais KM , Bredewold R , Mayer TE , Veenema AH . Sex differences in oxytocin receptor binding in forebrain regions: correlations with social interest in brain region‐ and sex‐ specific ways. Horm Behav. 2013;64:693‐701. doi:10.1016/j.yhbeh.2013.08.012 24055336

[434] Häussler HU , Jirikowski GF , Caldwell JD . Sex differences among oxytocin‐immunoreactive neuronal systems in the mouse hypothalamus. J Chem Neuroanat. 1990;3:271‐276 2204355

[435] Bale TL , Dorsa DM . Sex differences in and effects of estrogen on oxytocin receptor messenger ribonucleic acid expression in the ventromedial hypothalamus. Endocrinology. 1995;136:27‐32. doi:10.1210/endo.136.1.7828541 7828541

[436] Smeltzer MD , Curtis JT , Aragona BJ , Wang Z . Dopamine, oxytocin, and vasopressin receptor binding in the medial prefrontal cortex of monogamous and promiscuous voles. Neurosci Lett. 2006;394:146‐151. doi:10.1016/j.neulet.2005.10.019 16289323

[437] Campbell P , Ophir AG , Phelps SM . Central vasopressin and oxytocin receptor distributions in two species of singing mice. J Comp Neurol. 2009;516:321‐333. doi:10.1002/cne.22116 19637308

[438] Uhl‐Bronner S , Waltisperger E , Martínez‐Lorenzana G , Condes Lara M , Freund‐Mercier MJ . Sexually dimorphic expression of oxytocin binding sites in forebrain and spinal cord of the rat. Neuroscience. 2005;135:147‐154. doi:10.1016/j.neuroscience.2005.05.025 16084653

[439] Beery AK , Lacey EA , Francis DD . Oxytocin and vasopressin receptor distributions in a solitary and a social species of tuco‐tuco (Ctenomys haigi and Ctenomys sociabilis). J Comp Neurol. 2008;507:1847‐1859. doi:10.1002/cne.21638 18271022

[440] Bales KL , Plotsky PM , Young LJ , et al. Neonatal oxytocin manipulations have long‐lasting, sexually dimorphic effects on vasopressin receptors. Neuroscience. 2007;144:38‐45. doi:10.1016/j.neuroscience.2006.09.009 17055176 PMC1774559

[441] Hammock E , Levitt P . Oxytocin receptor ligand binding in embryonic tissue and postnatal brain development of the C57BL/6J mouse. Front Behav Neurosci. 2013;7:195. doi:10.3389/fnbeh.2013.00195 24376405 PMC3858721

[442] Dubois‐Dauphin M , Pévet P , Barberis C , Tribollet E , Dreifuss JJ . Localization of binding sites for oxytocin in the brain of the golden hamster. Neuroreport. 1992;3:797‐800. doi:10.1097/00001756-199209000-00019 1330065

[443] Loup F , Tribollet E , Dubois‐Dauphin M , Dreifuss JJ . Localization of high‐affinity binding sites for oxytocin and vasopressin in the human brain. An autoradiographic study. Brain Res. 1991;555:220‐232. doi:10.1016/0006-8993(91)90345-v 1657300

[444] Tribollet E , Audigier S , Dubois‐Dauphin M , Dreifuss JJ . Gonadal steroids regulate oxytocin receptors but not vasopressin receptors in the brain of male and female rats. An autoradiographical study. Brain Res. 1990;511:129‐140. doi:10.1016/0006-8993(90)90232-z 2158853

[445] Fuchs AR , Poblete VF . Oxytocin and uterine function in pregnant and parturient rats. Biol Reprod. 1970;2:387‐400. doi:10.1095/biolreprod2.3.387 5527837

[446] Oti T , Satoh K , Uta D , et al. Oxytocin influences male sexual activity via non‐synaptic axonal release in the spinal cord. Curr Biol. 2021;31:103‐114.e5. doi:10.1016/j.cub.2020.09.089 33125871 PMC7855431

[447] Belin V , Moos F , Richard P . Synchronization of oxytocin cells in the hypothalamic paraventricular and supraoptic nuclei in suckled rats: direct proof with paired extracellular recordings. Exp Brain Res. 1984;57:201‐203. doi:10.1007/BF00231147 6542868

[448] Donaldson ZR , Young LJ . Oxytocin, vasopressin, and the neurogenetics of sociality. Science. 2008;322:900‐904. doi:10.1126/science.1158668 18988842

[449] Williams JR , Insel TR , Harbaugh CR , Carter CS . Oxytocin administered centrally facilitates formation of a partner preference in female prairie voles (Microtus ochrogaster). J Neuroendocrinol. 1994;6:247‐250. doi:10.1111/j.1365-2826.1994.tb00579.x 7920590

[450] Insel TR , Hulihan TJ . A gender‐specific mechanism for pair bonding: oxytocin and partner preference formation in monogamous voles. Behav Neurosci. 1995;109:782‐789. doi:10.1037//0735-7044.109.4.782 7576222

[451] Liu Y , Curtis JT , Wang Z . Vasopressin in the lateral septum regulates pair bond formation in male prairie voles (Microtus ochrogaster). Behav Neurosci. 2001;115:910‐919. doi:10.1037//0735-7044.115.4.910 11508730

[452] Horie K , Blumenthal SA , Inoue K , Yada S , Nishimori K , Young LJ . Male, but not female, oxytocin receptor knockout prairie voles (*Microtus ochrogaster*) show impaired consolation behavior. Hormones and Behavior. 2025;169:105708. doi:10.1016/j.yhbeh.2025.105708 39965529

[453] Lukas M , Neumann ID . Social preference and maternal defeat‐induced social avoidance in virgin female rats: sex differences in involvement of brain oxytocin and vasopressin. Journal of Neuroscience Methods. 2014;234:101‐107. doi:10.1016/j.jneumeth.2014.03.013 24709115

[454] Lukas M , Toth I , Reber SO , Slattery DA , Veenema AH , Neumann ID . The neuropeptide oxytocin facilitates pro‐social behavior and prevents social avoidance in rats and mice. Neuropsychopharmacology. 2011;36:2159‐2168. doi:10.1038/npp.2011.95 21677650 PMC3176581

[455] Benelli A , Bertolini A , Poggioli R , Menozzi B , Basaglia R , Arletti R . Polymodal dose‐response curve for oxytocin in the social recognition test. Neuropeptides. 1995;28:251‐255. doi:10.1016/0143-4179(95)90029-2 7596490

[456] Engelmann M , Ebner K , Wotjak CT , Landgraf R . Endogenous oxytocin is involved in short‐term olfactory memory in female rats. Behav Brain Res. 1998;90:89‐94. doi:10.1016/s0166-4328(97)00084-3 9520216

[457] Bales KL , Carter CS . Sex differences and developmental effects of oxytocin on aggression and social behavior in prairie voles (Microtus ochrogaster). Horm Behav. 2003;44:178‐184. doi:10.1016/s0018-506x(03)00154-5 14609540

[458] Bales KL , Pfeifer LA , Carter CS . Sex differences and developmental effects of manipulations of oxytocin on alloparenting and anxiety in prairie voles. Dev Psychobiol. 2004;44:123‐131. doi:10.1002/dev.10165 14994263

[459] Mogi K , Ooyama R , Nagasawa M , Kikusui T . Effects of neonatal oxytocin manipulation on development of social behaviors in mice. Physiol Behav. 2014;133:68‐75. doi:10.1016/j.physbeh.2014.05.010 24857720

[460] Schiller B , Brustkern J , Walker M , Hamm A , Heinrichs M . Oxytocin has sex‐specific effects on trust and underlying neurophysiological processes. Psychoneuroendocrinology. 2023;151:106076. doi:10.1016/j.psyneuen.2023.106076 36931056

[461] Ditzen B , Nater UM , Schaer M , et al. Sex‐specific effects of intranasal oxytocin on autonomic nervous system and emotional responses to couple conflict. Soc Cogn Affect Neurosci. 2013;8:897‐902. doi:10.1093/scan/nss083 22842905 PMC3831552

[462] Kubzansky LD , Mendes WB , Appleton AA , Block J , Adler GK . A heartfelt response: oxytocin effects on response to social stress in men and women. Biol Psychol. 2012;90:1‐9. doi:10.1016/j.biopsycho.2012.02.010 22387929 PMC3327158

[463] Rilling JK , DeMarco AC , Hackett PD , et al. Sex differences in the neural and behavioral response to intranasal oxytocin and vasopressin during human social interaction. Psychoneuroendocrinology. 2014;39:237‐248. doi:10.1016/j.psyneuen.2013.09.022 24157401 PMC3842401

[464] Silvestri C , Almici V , Libri I , et al. Sex differences in the severity and progression of neuropsychiatric symptoms across different dementia types. Neurol Clin Pract. 2024;14:e200299. doi:10.1212/CPJ.0000000000200299 38720954 PMC11073872

[465] Selles MC , Fortuna JTS , de Faria YPR , et al. Oxytocin attenuates microglial activation and restores social and non‐social memory in APP/PS1 Alzheimer model mice. iScience. 2023;26:106545. doi:10.1016/j.isci.2023.106545 37128547 PMC10148027

[466] Usmani SS , Jung H‐G , Zhang Q , et al. Targeting the hypothalamus for modeling age‐related DNA methylation and developing OXT‐GnRH combinational therapy against Alzheimer's disease‐like pathologies in male mouse model. Nat Commun. 2024;15:9419. doi:10.1038/s41467-024-53507-8 39482312 PMC11528003

[467] Jackson HM , Soto I , Graham LC , Carter GW , Howell GR . Clustering of transcriptional profiles identifies changes to insulin signaling as an early event in a mouse model of Alzheimer's disease. BMC Genom. 2013;14:831. doi:10.1186/1471-2164-14-831 PMC3907022 24274089

[468] Li J , Li Y , Wang Z , et al. Oxytocin intervention mitigates pathological and behavioral impairments in APP/PS1 mice subjected to early social isolation. CNS Neurosci Ther. 2025;31:e70511. doi:10.1111/cns.70511 40635450 PMC12241820

[469] Raskind MA , Peskind ER , Lampe TH , Risse SC , Taborsky GJ , Dorsa D . Cerebrospinal fluid vasopressin, oxytocin, somatostatin, and beta‐endorphin in Alzheimer's disease. Arch Gen Psychiatry. 1986;43:382‐388. doi:10.1001/archpsyc.1986.01800040092013 2869744

[470] Mazurek MF , Beal MF , Bird ED , Martin JB . Oxytocin in Alzheimer's disease: postmortem brain levels. Neurology. 1987;37:1001‐1003. doi:10.1212/wnl.37.6.1001 3587615

[471] Petekkaya E , Burakgazi G , Kuş B , Melek İM , Arpacı A . Comparative study of the volume of the temporal lobe sections and neuropeptide effect in Alzheimer's patients and healthy persons. Int J Neurosci. 2021;131:725‐734. doi:10.1080/00207454.2020.1831490 33064056

[472] Zou C , Huang X , Zhang Y , et al. Potential biomarkers of Alzheimer's disease and cerebral small vessel disease. Front Mol Neurosci. 2022;15:996107. doi:10.3389/fnmol.2022.996107 36299860 PMC9588985

[473] Santiago JA , Quinn JP , Potashkin JA . Sex‐specific transcriptional rewiring in the brain of Alzheimer's disease patients. Front Aging Neurosci. 2022;14:1009368. doi:10.3389/fnagi.2022.1009368 36389068 PMC9659968

[474] Oliver LD , Stewart C , Coleman K , et al. Neural effects of oxytocin and mimicry in frontotemporal dementia: a randomized crossover study. Neurology. 2020;95:e2635‐47. doi:10.1212/WNL.0000000000010933 32963103 PMC7713736

[475] Finger EC . New potential therapeutic approaches in frontotemporal dementia: oxytocin, vasopressin, and social cognition. J Mol Neurosci. 2011;45:696‐701. doi:10.1007/s12031-011-9550-2 21618004

[476] Coleman KKL , Berry S , Cummings J , et al. Intranasal oxytocin for apathy in people with frontotemporal dementia (FOXY): a multicentre, randomised, double‐blind, placebo‐controlled, adaptive, crossover, phase 2a/2b superiority trial. Lancet Neurol. 2025;24:128‐139. doi:10.1016/S1474-4422(24)00456-3 39862881 PMC13557230

[477] Michaelian JC , McCade D , Hoyos CM , et al. Pilot randomized, double‐blind, placebo‐controlled crossover trial evaluating the feasibility of an intranasal oxytocin in improving social cognition in individuals living with Alzheimer's disease. J Alzheimers Dis Rep. 2023;7:715‐729. doi:10.3233/ADR-230013 37483320 PMC10357119

[478] Alanazi MM , Albaker AB , Alzaagi LA , et al. Oxytocin protects PC12 cells against β‐amyloid‐induced cell injury. Pharmaceuticals. 2025;18:390. doi:10.3390/ph18030390 40143166 PMC11944556

[479] Takahashi J , Yamada D , Ueta Y , et al. Oxytocin reverses Aβ‐induced impairment of hippocampal synaptic plasticity in mice. Biochem Biophys Res Commun. 2020;528:174‐178. doi:10.1016/j.bbrc.2020.04.046 32482389

[480] Zhang Y , Tang C , He Y , et al. Semaglutide ameliorates Alzheimer's disease and restores oxytocin in APP/PS1 mice and human brain organoid models. Biomed Pharmacother. 2024;180:117540. doi:10.1016/j.biopha.2024.117540 39405916

[481] Asaba T , Hamano S , Nanmo A , Seo J , Kageyama T , Fukuda J . Human iPSC‐derived cerebral organoids reveal oxytocin‐mediated protection against amyloid‐β pathology. Regenerative Therapy. 2025;30:259‐267. doi:10.1016/j.reth.2025.06.013 40654516 PMC12246583

[482] Yuan L , Liu S , Bai X , et al. Oxytocin inhibits lipopolysaccharide‐induced inflammation in microglial cells and attenuates microglial activation in lipopolysaccharide‐treated mice. J Neuroinflammation. 2016;13:77. doi:10.1186/s12974-016-0541-7 27075756 PMC4831099

[483] Cheng M , Ye C , Tian C , et al. Engineered macrophage‐biomimetic versatile nanoantidotes for inflammation‐targeted therapy against Alzheimer's disease by neurotoxin neutralization and immune recognition suppression. Bioact Mater. 2023;26:337‐352. doi:10.1016/j.bioactmat.2023.03.004 36950153 PMC10027514

[484] Ye C , Cheng M , Ma L , et al. Oxytocin nanogels inhibit innate inflammatory response for early intervention in Alzheimer's disease. ACS Appl Mater Interfaces. 2022;14:21822‐21835. doi:10.1021/acsami.2c00007 35510352

[485] Koulousakis P , Willems E , Schepers M , et al. Exogenous oxytocin administration restores memory in female APP/PS1 mice. J Alzheimers Dis. 2023;96:1207‐1219. doi:10.3233/JAD-230657 37927260 PMC10741313

[486] Takahashi J , Ueta Y , Yamada D , et al. Intracerebroventricular administration of oxytocin and intranasal administration of the oxytocin derivative improve β‐amyloid peptide (25‐35)‐induced memory impairment in mice. Neuropsychopharmacology Reports. 2022;42:492‐501. doi:10.1002/npr2.12292 36117475 PMC9773650

[487] El‐Ganainy SO , Soliman OA , Ghazy AA , et al. Intranasal oxytocin attenuates cognitive impairment, β‐amyloid burden and tau deposition in female rats with Alzheimer's disease: interplay of ERK1/2/GSK3β/Caspase‐3. Neurochem Res. 2022;47:2345‐2356. doi:10.1007/s11064-022-03624-x 35596040 PMC9352611

[488] Ye C , Wang S , Niu L , et al. Unlocking potential of oxytocin: improving intracranial lymphatic drainage for Alzheimer's disease treatment. Theranostics. 2024;14:4331‐4351. doi:10.7150/thno.98587 39113801 PMC11303076

[489] Sarahian N , Khodagholi F , Valian N , Ahmadiani A . Interplay of MeCP2/REST/Synaptophysin‐BDNF and intranasal oxytocin influence on Aβ‐induced memory and cognitive impairments. Behav Brain Res. 2025;476:115235. doi:10.1016/j.bbr.2024.115235 39236931

[490] Selles MC , Oliveira MM . The oxytocin puzzle: unlocking Alzheimer's disease. J Alzheimers Dis. 2024;97:1101‐1104. doi:10.3233/JAD-231127 38189754

[491] Caicedo Mera JC , Cárdenas Molano MA , García López CC , Acevedo Triana C , Martínez Cotrina J . Discussions and perspectives regarding oxytocin as a biomarker in human investigations. Heliyon. 2021;7:e08289. doi:10.1016/j.heliyon.2021.e08289 34805562 PMC8581272

[492] Shamay‐Tsoory SG , Abu‐Akel A . The social salience hypothesis of oxytocin. Biol Psychiatry. 2016;79:194‐202. doi:10.1016/j.biopsych.2015.07.020 26321019

[493] Ford CL , Young LJ . Refining oxytocin therapy for autism: context is key. Nat Rev Neurol. 2022;18:67‐68. doi:10.1038/s41582-021-00602-9 34880473 PMC8816821

[494] Zimmerman EA , Robinson AG . Hypothalamic neurons secreting vasopressin and neurophysin. Kidney Int. 1976;10:12‐24. doi:10.1038/ki.1976.75 59834

[495] Holmes MC , Antoni FA , Aguilera G , Catt KJ . Magnocellular axons in passage through the median eminence release vasopressin. Nature. 1986;319:326‐329. doi:10.1038/319326a0 3001538

[496] De Vries GJ , Panzica GC . Sexual differentiation of central vasopressin and vasotocin systems in vertebrates: different mechanisms, similar endpoints. Neuroscience. 2006;138:947‐955. doi:10.1016/j.neuroscience.2005.07.050 16310321 PMC1457099

[497] Swanson LW , Sawchenko PE . Hypothalamic integration: organization of the paraventricular and supraoptic nuclei. Annu Rev Neurosci. 1983;6:269‐324. doi:10.1146/annurev.ne.06.030183.001413 6132586

[498] Arnauld E , Vincent JD , Dreifuss JJ . Firing patterns of hypothalamic supraoptic neurons during water deprivation in monkeys. Science. 1974;185:535‐537. doi:10.1126/science.185.4150.535 4210247

[499] Watanabe K , Koibuchi N , Ohtake H , Yamaoka S . Circadian rhythms of vasopressin release in primary cultures of rat suprachiasmatic nucleus. Brain Res. 1993;624:115‐120. doi:10.1016/0006-8993(93)90067-W 8252382

[500] de Vries GJ , Buijs RM , Swaab DF . Ontogeny of the vasopressinergic neurons of the suprachiasmatic nucleus and their extrahypothalamic projections in the rat brain–presence of a sex difference in the lateral septum. Brain Res. 1981;218:67‐78. doi:10.1016/0006-8993(81)90989-6 7023607

[501] Hernández VS , Hernández OR , Perez de la Mora M , et al. Hypothalamic vasopressinergic projections innervate central amygdala GABAergic neurons: implications for anxiety and stress coping. Front Neural Circuits. 2016;10:92. doi:10.3389/fncir.2016.00092 27932956 PMC5122712

[502] Rigney N , Whylings J , de Vries GJ , Petrulis A . Sex differences in the control of social investigation and anxiety by vasopressin cells of the paraventricular nucleus of the hypothalamus. Neuroendocrinology. 2021;111:521‐535. doi:10.1159/000509421 32541145 PMC7736187

[503] Woodson J , Bergan JF . Uncovering the brain‐wide pattern of synaptic input to vasopressin‐expressing neurons in the paraventricular nucleus of the hypothalamus. J Comp Neurol. 2023;531:1017‐1031. doi:10.1002/cne.25476 37121600 PMC10566340

[504] Freda SN , Priest MF , Badong D , Xiao L , Liu Y , Kozorovitskiy Y . Brainwide input‐output architecture of paraventricular oxytocin and vasopressin neurons 2022:2022.01.17.476652. doi:10.1101/2022.01.17.476652

[505] Choi DC , Furay AR , Evanson NK , et al. The role of the posterior medial bed nucleus of the stria terminalis in modulating hypothalamic‐pituitary‐adrenocortical axis responsiveness to acute and chronic stress. Psychoneuroendocrinology. 2008;33:659‐669. doi:10.1016/j.psyneuen.2008.02.006 18378095 PMC3641575

[506] Buijs RM . Intra‐ and extrahypothalamic vasopressin and oxytocin pathways in the rat. Pathways to the limbic system, medulla oblongata and spinal cord. Cell Tissue Res. 1978;192:423‐435. doi:10.1007/BF00212323 699026

[507] Tong WH , Abdulai‐Saiku S , Vyas A . Arginine vasopressin in the medial amygdala causes greater post‐stress recruitment of hypothalamic vasopressin neurons. Mol Brain. 2021;14:141. doi:10.1186/s13041-021-00850-2 34526037 PMC8442369

[508] Tong WH , Abdulai‐Saiku S , Vyas A . Medial amygdala arginine vasopressin neurons regulate innate aversion to cat odors in male mice. Neuroendocrinology. 2021;111:505‐520. doi:10.1159/000508862 32447337

[509] Ressler KJ . Amygdala activity, fear, and anxiety: modulation by stress. Biol Psychiatry. 2010;67:1117‐1119. doi:10.1016/j.biopsych.2010.04.027 20525501 PMC2882379

[510] Wang Z , Ferris CF , De Vries GJ . Role of septal vasopressin innervation in paternal behavior in prairie voles (Microtus ochrogaster). Proc Natl Acad Sci U S A. 1994;91:400‐404. doi:10.1073/pnas.91.1.400 8278401 PMC42955

[511] Dobie DJ , Miller MA , Urban JH , Raskind MA , Dorsa DM . Age‐related decline of vasopressin mRNA in the bed nucleus of the stria terminalis. Neurobiol Aging. 1991;12:419‐423. doi:10.1016/0197-4580(91)90067-t 1770975

[512] Rigney N , de Vries GJ , Petrulis A . Sex differences in afferents and efferents of vasopressin neurons of the bed nucleus of the stria terminalis and medial amygdala in mice. Horm Behav. 2023;154:105407. doi:10.1016/j.yhbeh.2023.105407 37523807 PMC10529859

[513] Fliers E , De Vries GJ , Swaab DF . Changes with aging in the vasopressin and oxytocin innervation of the rat brain. Brain Res. 1985;348:1‐8. doi:10.1016/0006-8993(85)90351-8 3904923

[514] Van Zwieten EJ , Kos WT , Ravid R , Swaab DF . Decreased number of vasopressin immunoreactive neurons in the medial amygdala and locus coeruleus of the aged rat. Neurobiol Aging. 1993;14:245‐248. doi:10.1016/0197-4580(93)90008-y 8321392

[515] Mieda M , Ono D , Hasegawa E , et al. Cellular clocks in AVP neurons of the SCN are critical for interneuronal coupling regulating circadian behavior rhythm. Neuron. 2015;85:1103‐1116. doi:10.1016/j.neuron.2015.02.005 25741730

[516] de Wied D , Diamant M , Fodor M . Central nervous system effects of the neurohypophyseal hormones and related peptides. Frontiers in Neuroendocrinology. 1993;14:251‐302. doi:10.1006/frne.1993.1009 8258377

[517] Gumerova A , Pevnev G , Korkmaz F , et al. Sex–specific single transcript level atlas of vasopressin and its receptor (AVPR1a) in the mouse brain. eLife. 2026;14:RP105355. doi:10.7554/eLife.105355.3 41837842 PMC12991652

[518] Tribollet E , Barberis C , Jard S , Dubois‐Dauphin M , Dreifuss JJ . Localization and pharmacological characterization of high affinity binding sites for vasopressin and oxytocin in the rat brain by light microscopic autoradiography. Brain Res. 1988;442:105‐118. doi:10.1016/0006-8993(88)91437-0 2834008

[519] Roper J , O'Carroll A‐M , Young W , Lolait S . The vasopressin Avprlb receptor: molecular and pharmacological studies. Stress. 2011;14:98‐115. doi:10.3109/10253890.2010.512376 20828336 PMC3016603

[520] Dubois‐Dauphin M , Theler JM , Zaganidis N , et al. Expression of vasopressin receptors in hamster hypothalamus is sexually dimorphic and dependent upon photoperiod. Proc Natl Acad Sci U S A. 1991;88:11163‐11167. doi:10.1073/pnas.88.24.11163 1837144 PMC53094

[521] Rigney N , Beaumont R , Petrulis A . Sex differences in vasopressin 1a receptor regulation of social communication within the lateral habenula and dorsal raphe of mice. Horm Behav. 2020;121:104715. doi:10.1016/j.yhbeh.2020.104715 32067962 PMC7249673

[522] van Leeuwen FW , Caffe AR , De Vries GJ . Vasopressin cells in the bed nucleus of the stria terminalis of the rat: sex differences and the influence of androgens. Brain Res. 1985;325:391‐394. doi:10.1016/0006-8993(85)90348-8 3978433

[523] Wang Z . Species differences in the vasopressin‐immunoreactive pathways in the bed nucleus of the stria terminalis and medial amygdaloid nucleus in prairie voles (Microtus ochrogaster) and meadow voles (Microtus pennsylvanicus). Behav Neurosci. 1995;109:305‐311. doi:10.1037//0735-7044.109.2.305 7619320

[524] Delville Y , Koh ET , Ferris CF . Sexual differences in the magnocellular vasopressinergic system in golden hamsters. Brain Res Bull. 1994;33:535‐540. doi:10.1016/0361-9230(94)90080-9 8186999

[525] De Vries GJ , Buds RM , Swaab DF . Ontogeny of the vasopressinergic neurons of the suprachiasmatic nucleus and their extrahypothalamic projections in the rat brain—presence of a sex difference in the lateral septum. Brain Res. 1981;218:67‐78. doi:10.1016/0006-8993(81)90989-6 7023607

[526] De Vries GJ , Rissman EF , Simerly RB , et al. A model system for study of sex chromosome effects on sexually dimorphic neural and behavioral traits. J Neurosci. 2002;22:9005‐9014. doi:10.1523/JNEUROSCI.22-20-09005.2002 12388607 PMC6757680

[527] Bakker J , De Mees C , Douhard Q , et al. Alpha‐fetoprotein protects the developing female mouse brain from masculinization and defeminization by estrogens. Nat Neurosci. 2006;9:220‐226. doi:10.1038/nn1624 16388309

[528] Gatewood JD , Wills A , Shetty S , et al. Sex chromosome complement and gonadal sex influence aggressive and parental behaviors in mice. J Neurosci. 2006;26:2335‐2342. doi:10.1523/JNEUROSCI.3743-05.2006 16495461 PMC6674813

[529] Rood BD , Stott RT , You S , Smith CJW , Woodbury ME , De Vries GJ . Site of origin of and sex differences in the vasopressin innervation of the mouse (Mus musculus) brain. J Comp Neurol. 2013;521:2321‐2358. doi:10.1002/cne.23288 23239101

[530] Lonstein JS , De Vries GJ . Sex differences in the parental behaviour of adult virgin prairie voles: independence from gonadal hormones and vasopressin. J Neuroendocrinol. 1999;11:441‐449. doi:10.1046/j.1365-2826.1999.00361.x 10336725

[531] Crenshaw BJ , De Vries GJ , Yahr P . Vasopressin innervation of sexually dimorphic structures of the gerbil forebrain under various hormonal conditions. J Comp Neurol. 1992;322:589‐598. doi:10.1002/cne.903220412 1401252

[532] Buijs RM , Pévet P , Masson‐Pévet M , et al. Seasonal variation in vasopressin innervation in the brain of the European hamster (*Cricetus cricetus*). Brain Research. 1986;371:193‐196. doi:10.1016/0006-8993(86)90829-2 3708343

[533] Hermes ML , Buijs RM , Masson‐Pévet M , Pévet P . Seasonal changes in vasopressin in the brain of the garden dormouse (Eliomys quercinus L.). J Comp Neurol. 1990;293:340‐346. doi:10.1002/cne.902930303 2324321

[534] Wang Z , De Vries GJ . Androgen and estrogen effects on vasopressin messenger RNA expression in the medial amygdaloid nucleus in male and female rats. J Neuroendocrinol. 1995;7:827‐831. doi:10.1111/j.1365-2826.1995.tb00722.x 8748118

[535] Miller MA , Vician L , Clifton DK , Dorsa DM . Sex differences in vasopressin neurons in the bed nucleus of the stria terminalis by in situ hybridization. Peptides. 1989;10:615‐619. doi:10.1016/0196-9781(89)90152-6 2780420

[536] Taylor PV , Veenema AH , Paul MJ , Bredewold R , Isaacs S , de Vries GJ . Sexually dimorphic effects of a prenatal immune challenge on social play and vasopressin expression in juvenile rats. Biol Sex Differ. 2012;3:15. doi:10.1186/2042-6410-3-15 22697211 PMC3420237

[537] Delville Y , Ferris CF . Sexual differences in vasopressin receptor binding within the ventrolateral hypothalamus in golden hamsters. Brain Research. 1995;681:91‐96. doi:10.1016/0006-8993(95)00291-W 7552297

[538] Share L , Crofton JT , Ouchi Y . Vasopressin: sexual dimorphism in secretion, cardiovascular actions and hypertension. Am J Med Sci. 1988;295:314‐319. doi:10.1097/00000441-198804000-00017 3364463

[539] Asplund R , Aberg H . Diurnal variation in the levels of antidiuretic hormone in the elderly. J Intern Med. 1991;229:131‐134. doi:10.1111/j.1365-2796.1991.tb00320.x 1997638

[540] van Londen L , Goekoop JG , van Kempen GM , et al. Plasma levels of arginine vasopressin elevated in patients with major depression. Neuropsychopharmacology. 1997;17:284‐292. doi:10.1016/S0893-133X(97)00054-7 9326754

[541] Miller M , Bales KL , Taylor SL , et al. Oxytocin and vasopressin in children and adolescents with autism spectrum disorders: sex differences and associations with symptoms. Autism Res. 2013;6:91‐102. doi:10.1002/aur.1270 23413037 PMC3657571

[542] Graugaard‐Jensen C , Hvistendahl GM , Frøkiaer J , Bie P , Djurhuus JC . Urinary concentration does not exclusively rely on plasma vasopressin. A study between genders. Gender and diurnal urine regulation. Acta Physiol (Oxf). 2014;212:97‐105. doi:10.1111/apha.12337 24965868

[543] Kelberman MA , Winther KE , Medvedeva YM , Donaldson ZR . Aging leads to sex‐dependent effects on pair bonding and increased number of oxytocin‐producing neurons in monogamous prairie voles. Horm Behav. 2024;166:105647. doi:10.1016/j.yhbeh.2024.105647 39342749 PMC11602381

[544] Dubois‐Dauphin M , Barberis C , de Bilbao F . Vasopressin receptors in the mouse *(Mus musculus)* brain: sex‐related expression in the medial preoptic area and hypothalamus. Brain Res. 1996;743:32‐39. doi:10.1016/S0006-8993(96)01019-0 9017227

[545] Insel TR , Gelhard R , Shapiro LE . The comparative distribution of forebrain receptors for neurohypophyseal peptides in monogamous and polygamous mice. Neuroscience. 1991;43:623‐630. doi:10.1016/0306-4522(91)90321-e 1656322

[546] Van der Woude PF , Goudsmit E , Wierda M , et al. No vasopressin cell loss in the human hypothalamus in aging and Alzheimer's disease. Neurobiol Aging. 1995;16:11‐18. doi:10.1016/0197-4580(95)80003-a 7723930

[547] Goudsmit E , Hofman MA , Fliers E , Swaab DF . The supraoptic and paraventricular nuclei of the human hypothalamus in relation to sex, age and Alzheimer's disease. Neurobiol Aging. 1990;11:529‐536. doi:10.1016/0197-4580(90)90114-f 2234284

[548] Lucassen PJ , Van Heerikhuize JJ , Guldenaar SE , Pool CW , Hofman MA , Swaab DF . Unchanged amounts of vasopressin mRNA in the supraoptic and paraventricular nucleus during aging and in Alzheimer's disease. J Neuroendocrinol. 1997;9:297‐305. doi:10.1046/j.1365-2826.1997.t01-1-00583.x 9147293

[549] Rodeck H , Lederis K , Heller H . The hypothalamoneurohypophysial system in old rats. J Endocrinol. 1960;21:225‐228. doi:10.1677/joe.0.0210225 13742514

[550] Vogels OJ , Broere CA , Nieuwenhuys R . Neuronal hypertrophy in the human supraoptic and paraventricular nucleus in aging and Alzheimer's disease. Neurosci Lett. 1990;109:62‐67. doi:10.1016/0304-3940(90)90538-k 2314642

[551] Lucassen PJ , Ravid R , Gonatas NK , Swaab DF . Activation of the human supraptic and paraventricular nucleus neurons with aging and in Alzheimer's disease as judged from increasing size of the Golgi apparatus. Brain Research. 1993;632:105‐113. doi:10.1016/0006-8993(93)91144-H 8149218

[552] Roberts DE , Killiany RJ , Rosene DL . Neuron numbers in the hypothalamus of the normal aging rhesus monkey: stability across the adult lifespan and between the sexes. J Comp Neurol. 2012;520:1181‐1197. doi:10.1002/cne.22761 21935936 PMC4278435

[553] Swaab DF , Fliers E , Partiman TS . The suprachiasmatic nucleus of the human brain in relation to sex, age and senile dementia. Brain Res. 1985;342:37‐44. doi:10.1016/0006-8993(85)91350-2 4041816

[554] Son G , Mladinov M , Pereira FL , et al. Spatially conserved pathoprotein profiling in the human suprachiasmatic nucleus in progressive Alzheimer disease stages 2024:2024.03.07.584000. doi:10.1101/2024.03.07.584000

[555] Duffy JF , Cain SW , Chang A‐M , et al. Sex difference in the near‐24‐hour intrinsic period of the human circadian timing system. Proc Natl Acad Sci U S A. 2011;108(Suppl 3):15602‐15608. doi:10.1073/pnas.1010666108 21536890 PMC3176605

[556] Thirouin ZS , Gizowski C , Murtaz A , Bourque CW . Sex‐specific differences in the circadian pattern of action potential firing by rat suprachiasmatic nucleus vasopressin neurons. J Neuroendocrinol. 2023;35:e13273. doi:10.1111/jne.13273 37132408

[557] Guarnieri B , Maestri M , Cucchiara F , et al. Multicenter study on sleep and circadian alterations as objective markers of mild cognitive impairment and Alzheimer's disease reveals sex differences. J Alzheimers Dis. 2020;78:1707‐1719. doi:10.3233/JAD-200632 33185597

[558] Kania A , Sambak P , Gugula A , et al. Electrophysiology and distribution of oxytocin and vasopressin neurons in the hypothalamic paraventricular nucleus: a study in male and female rats. Brain Struct Funct. 2020;225:285‐304. doi:10.1007/s00429-019-01989-4 31820102

[559] Otero‐García M , Agustín‐Pavón C , Lanuza E , Martínez‐García F . Distribution of oxytocin and co‐localization with arginine vasopressin in the brain of mice. Brain Struct Funct. 2016;221:3445‐3473. doi:10.1007/s00429-015-1111-y 26388166

[560] Dale HH , Laidlaw PP . The physiological action of beta‐iminazolylethylamine. J Physiol. 1910;41:318‐344. doi:10.1113/jphysiol.1910.sp001406 16993030 PMC1512903

[561] PubChem . Histamine n.d. https://pubchem.ncbi.nlm.nih.gov/compound/774 (accessed August 30, 2025)

[562] Vaaler GL , Snell EE . Pyridoxal 5’‐phosphate dependent histidine decarboxylase: overproduction, purification, biosynthesis of soluble site‐directed mutant proteins and replacement of conserved residues. Biochemistry. 1989;28:7306‐7313. doi:10.1021/bi00444a024 2684275 10.1021/bi00444a024

[563] Airaksinen MS , Paetau A , Paljärvi L , et al. Histamine neurons in human hypothalamus: anatomy in normal and Alzheimer diseased brains. Neuroscience. 1991;44:465‐481. doi:10.1016/0306‐4522(91)90070‐5 1719449 10.1016/0306-4522(91)90070-5

[564] Shan L , Dauvilliers Y , Siegel JM . Interactions of the histamine and hypocretin systems in CNS disorders. Nat Rev Neurol. 2015;11:401‐413. doi:10.1038/nrneurol.2015.99 26100750 10.1038/nrneurol.2015.99 PMC8744538

[565] John J , Thannickal TC , McGregor R , et al. Greatly increased numbers of histamine cells in human narcolepsy with cataplexy. Ann Neurol. 2013;74:786‐793. doi:10.1002/ana.23968 23821583 10.1002/ana.23968 PMC8211429

[566] Valko PO , Gavrilov YV , Yamamoto M , et al. Increase of histaminergic tuberomammillary neurons in narcolepsy. Ann Neurol. 2013;74:794‐804. doi:10.1002/ana.24019 24006291 10.1002/ana.24019

[567] Nieuwenhuys R , Voogd J , Van Huijzen C . The Human Central Nervous System. Springer; 2008. doi:10.1007/978‐3‐540‐34686‐9

[568] Silver R , Silverman AJ , Vitković L , Lederhendler II . Mast cells in the brain: evidence and functional significance. Trends Neurosci. 1996;19:25‐31. doi:10.1016/0166‐2236(96)81863‐7 8787137 10.1016/0166-2236(96)81863-7

[569] Giorgi FS , Galgani A , Puglisi‐Allegra S , Busceti CL , Fornai F . The connections of Locus Coeruleus with hypothalamus: potential involvement in Alzheimer's disease. J Neural Transm (Vienna). 2021;128:589‐613. doi:10.1007/s00702‐021‐02338‐8 33942174 10.1007/s00702-021-02338-8 PMC8105225

[570] Eriksson KS , Sergeeva O , Brown RE , Haas HL . Orexin/hypocretin excites the histaminergic neurons of the tuberomammillary nucleus. J Neurosci. 2001;21:9273‐9279. doi:10.1523/JNEUROSCI.21‐23‐09273.2001 11717361 10.1523/JNEUROSCI.21-23-09273.2001 PMC6763926

[571] Cheng J , Wu F , Zhang M , et al. the interaction between the ventrolateral preoptic nucleus and the tuberomammillary nucleus in regulating the sleep‐wakefulness cycle. Front Neurosci. 2020;14:615854. doi:10.3389/fnins.2020.615854 33381012 10.3389/fnins.2020.615854 PMC7767984

[572] Brown RE , Stevens DR , Haas HL . The physiology of brain histamine. Prog Neurobiol. 2001;63:637‐672. doi:10.1016/s0301‐0082(00)00039‐3 11164999 10.1016/s0301-0082(00)00039-3

[573] Connelly WM , Shenton FC , Lethbridge N , et al. The histamine H4 receptor is functionally expressed on neurons in the mammalian CNS. Br J Pharmacol. 2009;157:55‐63. doi:10.1111/j.1476‐5381.2009.00227.x 19413571 10.1111/j.1476-5381.2009.00227.x PMC2697783

[574] Panula P , Chazot PL , Cowart M , et al. International union of basic and clinical pharmacology. XCVIII. Histamine receptors. Pharmacol Rev. 2015;67:601‐655. doi:10.1124/pr.114.010249 26084539 10.1124/pr.114.010249 PMC4485016

[575] Fang Z , Chen J , Zheng Y , Chen Z . Targeting histamine and histamine receptors for memory regulation: an emotional perspective. Curr Neuropharmacol. 2024;22:1846‐1869. doi:10.2174/1570159×22666240128003108 38288837 10.2174/1570159X22666240128003108 PMC11284729

[576] Saraiva C , Barata‐Antunes S , Santos T , et al. Histamine modulates hippocampal inflammation and neurogenesis in adult mice. Sci Rep. 2019;9:8384. doi:10.1038/s41598‐019‐44816‐w 31182747 10.1038/s41598-019-44816-w PMC6558030

[577] Kárpáti A , Yoshikawa T , Naganuma F , et al. Histamine H1 receptor on astrocytes and neurons controls distinct aspects of mouse behaviour. Sci Rep. 2019;9:16451. doi:10.1038/s41598‐019‐52623‐6 31712580 10.1038/s41598-019-52623-6 PMC6848115

[578] Daher F , Mattioli R . Impairment in the aversive memory of mice in the inhibitory avoidance task but not in the elevated plus maze through intra‐amygdala injections of histamine. Pharmacol Biochem Behav. 2015;135:237‐245. doi:10.1016/j.pbb.2015.05.023 26079070 10.1016/j.pbb.2015.05.023

[579] Oh J , Petersen C , Walsh CM , Bittencourt JC , Neylan TC , Grinberg LT . The role of co‐neurotransmitters in sleep and wake regulation. Mol Psychiatry. 2019;24:1284‐1295. doi:10.1038/s41380‐018‐0291‐2 30377299 10.1038/s41380-018-0291-2 PMC6491268

[580] Shan L , Bossers K , Unmehopa U , Bao A‐M , Swaab DF . Alterations in the histaminergic system in Alzheimer's disease: a postmortem study. Neurobiol Aging. 2012;33:2585‐2598. doi:10.1016/j.neurobiolaging.2011.12.026 22284987 10.1016/j.neurobiolaging.2011.12.026

[581] Nakamura S , Takemura M , Ohnishi K , et al. Loss of large neurons and occurrence of neurofibrillary tangles in the tuberomammillary nucleus of patients with Alzheimer's disease. Neurosci Lett. 1993;151:196‐199. doi:10.1016/0304‐3940(93)90019‐h 8506080 10.1016/0304-3940(93)90019-h

[582] Mazurkiewicz‐Kwilecki IM , Nsonwah S . Changes in the regional brain histamine and histidine levels in postmortem brains of Alzheimer patients. Can J Physiol Pharmacol. 1989;67:75‐78. doi:10.1139/y89‐013 2713757 10.1139/y89-013

[583] Cacabelos R , Yamatodani A , Niigawa H , et al. Brain histamine in Alzheimer's disease. Methods Find Exp Clin Pharmacol. 1989;11:353‐360. 2755282

[584] Panula P , Rinne J , Kuokkanen K , et al. Neuronal histamine deficit in Alzheimer's disease. Neuroscience. 1997;82:993‐997. doi:10.1016/S0306‐4522(97)00353‐9 10.1016/s0306-4522(97)00353-9 9466423

[585] Motawaj M , Peoc'h K , Callebert J , Arrang J‐M . CSF levels of the histamine metabolite tele‐methylhistamine are only slightly decreased in Alzheimer's disease. J Alzheimers Dis. 2010;22:861‐871. doi:10.3233/JAD‐2010‐100381 20858978 10.3233/JAD-2010-100381

[586] Zhang W , Zhang X , Zhang Y , Qu C , Zhou X , Zhang S . Histamine induces microglia activation and the release of proinflammatory mediators in rat brain via H1R or H4R. J Neuroimmune Pharmacol. 2020;15:280‐291. doi:10.1007/s11481‐019‐09887‐6 31863333 10.1007/s11481-019-09887-6

[587] Haas HL , Sergeeva OA , Selbach O . Histamine in the nervous system. Physiol Rev. 2008;88:1183‐1241. doi:10.1152/physrev.00043.2007 18626069 10.1152/physrev.00043.2007

[588] Ishunina TA , van Heerikhuize JJ , Ravid R , Swaab DF . Estrogen receptors and metabolic activity in the human tuberomamillary nucleus: changes in relation to sex, aging and Alzheimer's disease. Brain Research. 2003;988:84‐96. doi:10.1016/S0006‐8993(03)03347‐X 14519529 10.1016/s0006-8993(03)03347-x

[589] Cs Fekete , Strutton PH , Cagampang FRA , et al. Estrogen receptor immunoreactivity is present in the majority of central histaminergic neurons: evidence for a new neuroendocrine pathway associated with luteinizing hormone‐releasing hormone‐synthesizing neurons in rats and Humans1. Endocrinology. 1999;140:4335‐4341. doi:10.1210/endo.140.9.6968 10465307 10.1210/endo.140.9.6968

[590] Netter KJ , Cohn VH , Shore PA . Sex difference in histamine metabolism in the rat. Am J Physiol‐Legacy Content. 1961;201:224‐226. doi:10.1152/ajplegacy.1961.201.2.224 10.1152/ajplegacy.1961.201.2.224 13728458

[591] Westling H , Wetterqvist H . Further observations on the difference in the metabolism of histamine in male and female rats. Br J Pharmacol Chemother. 1962;19:64‐73. doi:10.1111/j.1476‐5381.1962.tb01427.x 14006354 10.1111/j.1476-5381.1962.tb01427.x PMC1482241

[592] Ghi P , Orsetti M , Gamalero SR , Ferretti C . Sex differences in memory performance in the object recognition test. Possible role of histamine receptors. Pharmacol Biochem Behav. 1999;64:761‐766. doi:10.1016/s0091‐3057(99)00143‐4 10593199 10.1016/s0091-3057(99)00143-4

[593] Mercer LP , Kelley DS , Bundrant HM , Haq AU , Humphries LL . Gender affects rats’ central nervous system histaminergic responses to dietary manipulation. J Nutr. 1996;126:3128‐3135. doi:10.1093/jn/126.12.3128 9001383 10.1093/jn/126.12.3128

[594] Mori H , Matsuda K‐I , Yamawaki M , Kawata M . Estrogenic regulation of histamine receptor subtype H1 expression in the ventromedial nucleus of the hypothalamus in female rats. PLOS ONE. 2014;9:e96232. doi:10.1371/journal.pone.0096232 24805361 10.1371/journal.pone.0096232 PMC4013143

[595] Ghi P , Ferretti C , Lupi ML , Blengio M , Portaleone P . Sexual dimorphism in [3H]histamine binding sites of rat cerebral cortex. Pharmacol Res. 1991;23:187‐193. doi:10.1016/S1043‐6618(05)80121‐5 2062793 10.1016/s1043-6618(05)80121-5

[596] Jonassen F , Granerus G , Wetterqvist H . Histamine metabolism during the menstrual cycle. Acta Obstet Gynecol Scand. 1976;55:297‐304. doi:10.3109/00016347609158501 973560 10.3109/00016347609158501

[597] Zandt MV , Pittenger C . Sex differences in histamine regulation of striatal dopamine. J Neurosci. 2025;45:e2182242025. doi:10.1523/JNEUROSCI.2182‐24.2025 40355265 10.1523/JNEUROSCI.2182-24.2025 PMC12160404

[598] Seltzer AM , Donoso AO . Effects of ovariectomy and ovarian steroids on binding of 3H‐mepyramine, an H1‐histamine antagonist, in rat hypothalamus. Brain Res Bull. 1989;23:183‐186. doi:10.1016/0361‐9230(89)90145‐7 2819476 10.1016/0361-9230(89)90145-7

[599] Fernández‐Guasti A , Kruijver FP , Fodor M , Swaab DF . Sex differences in the distribution of androgen receptors in the human hypothalamus. J Comp Neurol. 2000;425:422‐435. doi:10.1002/1096‐9861(20000925)425:3%253C422::aid‐cne7%253E3.0.co;2‐h 10972942 10.1002/1096-9861(20000925)425:3<422::aid-cne7>3.0.co;2-h

[600] Zhang L , Verwer RWH , van Heerikhuize J , et al. Progesterone receptor distribution in the human hypothalamus and its association with suicide. Acta Neuropathol Commun. 2024;12:16. doi:10.1186/s40478‐024‐01733‐y 38263257 10.1186/s40478-024-01733-y PMC10807127

[601] Berger SN , Baumberger B , Samaranayake S , et al. an in vivo definition of brain histamine dynamics reveals critical neuromodulatory roles for this elusive messenger. Int J Mol Sci. 2022;23:14862. doi:10.3390/ijms232314862 36499189 10.3390/ijms232314862 PMC9738190

[602] Easton A , Norton J , Goodwillie A , Pfaff DW . Sex differences in mouse behavior following pyrilamine treatment: role of histamine 1 receptors in arousal. Pharmacol Biochem Behav. 2004;79:563‐572. doi:10.1016/j.pbb.2004.09.014 15582029 10.1016/j.pbb.2004.09.014

[603] Costa A , Ducourneau E , Curti L , et al. Chemogenetic activation or inhibition of histaminergic neurons bidirectionally modulates recognition memory formation and retrieval in male and female mice. Sci Rep. 2024;14:11283. doi:10.1038/s41598‐024‐61998‐0 38760416 10.1038/s41598-024-61998-0 PMC11101472

[604] Robinson EN , Saxon‐Kelley D , Tiu A , Haq A‐U , Mercer LP . The effect of sex on central histaminergic responses and corticosterone bioperiodicity in Sprague‐Dawley rats. J Nutr Biochem. 2005;16:38‐43. doi:10.1016/j.jnutbio.2004.09.001 15629239 10.1016/j.jnutbio.2004.09.001

[605] Zlomuzica A , Dere D , Binder S , De Souza Silva MA , Huston JP , Dere E . Neuronal histamine and cognitive symptoms in Alzheimer's disease. Neuropharmacology. 2016;106:135‐145. doi:10.1016/j.neuropharm.2015.05.007 26025658 10.1016/j.neuropharm.2015.05.007

[606] Sedeyn JC , Wu H , Hobbs RD , Levin EC , Nagele RG , Venkataraman V . Histamine induces Alzheimer's disease‐like blood brain barrier breach and local cellular responses in mouse brain organotypic cultures. Biomed Res Int. 2015;2015:937148. doi:10.1155/2015/937148 26697497 10.1155/2015/937148 PMC4677161

[607] Prell GD , Khandelwal JK , Burns RS , LeWitt PA , Green JP . Influence of age and gender on the levels of histamine metabolites and *pros*‐methylimidazoleacetic acid in human cerebrospinal fluid. Archives of Gerontology and Geriatrics. 1990;11:85‐95. doi:10.1016/0167‐4943(90)90059‐F 15374496 10.1016/0167-4943(90)90059-f

[608] Bardgett ME , Davis NN , Schultheis PJ , Griffith MS . Ciproxifan, an H3 receptor antagonist, alleviates hyperactivity and cognitive deficits in the APP Tg2576 mouse model of Alzheimer's disease. Neurobiol Learn Mem. 2011;95:64‐72. doi:10.1016/j.nlm.2010.10.008 21073971 10.1016/j.nlm.2010.10.008 PMC3034295

[609] Bitner RS , Markosyan S , Nikkel AL , Brioni JD . In‐vivo histamine H3 receptor antagonism activates cellular signaling suggestive of symptomatic and disease modifying efficacy in Alzheimer's disease. Neuropharmacology. 2011;60:460‐466. doi:10.1016/j.neuropharm.2010.10.026 21044639 10.1016/j.neuropharm.2010.10.026

[610] Delay‐Goyet P , Blanchard V , Schussler N , et al. SAR110894, a potent histamine H3‐receptor antagonist, displays disease‐modifying activity in a transgenic mouse model of tauopathy. Alzheimers Dement (N Y). 2016;2:267‐280. doi:10.1016/j.trci.2016.10.002 29067314 10.1016/j.trci.2016.10.002 PMC5651361

[611] Mani V , Jaafar SM , Azahan NSM , et al. Ciproxifan improves cholinergic transmission, attenuates neuroinflammation and oxidative stress but does not reduce amyloid level in transgenic mice. Life Sci. 2017;180:23‐35. doi:10.1016/j.lfs.2017.05.013 28501482 10.1016/j.lfs.2017.05.013

[612] Wang J , Liu B , Xu Y , et al. Thioperamide attenuates neuroinflammation and cognitive impairments in Alzheimer's disease via inhibiting gliosis. Exp Neurol. 2022;347:113870. doi:10.1016/j.expneurol.2021.113870 34563511 10.1016/j.expneurol.2021.113870

[613] Zou Y , Yang L , Zhu J , et al. Pitolisant alleviates brain network dysfunction and cognitive deficits in a mouse model of Alzheimer's disease. Transl Psychiatry. 2025;15:126. doi:10.1038/s41398‐025‐03358‐8 40185739 10.1038/s41398-025-03358-8 PMC11971262

[614] Wang L , Fang J , Jiang H , et al. 7‐Pyrrolidinethoxy‐4′‐methoxyisoflavone prevents amyloid β–induced injury by regulating histamine H3 receptor‐mediated cAMP/CREB and AKT/GSK3β pathways. Front Neurosci. 2019;13:334. doi:10.3389/fnins.2019.00334 31024245 10.3389/fnins.2019.00334 PMC6468582

[615] van Meer P , Pfankuch T , Raber J . Reduced histamine levels and H3 receptor antagonist‐induced histamine release in the amygdala of Apoe‐/‐ mice. J Neurochem. 2007;103:124‐130. doi:10.1111/j.1471‐4159.2007.04705.x 17573822 10.1111/j.1471-4159.2007.04705.x

[616] Sundvik M , Chen Y‐C , Panula P . Presenilin1 regulates histamine neuron development and behavior in zebrafish, danio rerio. J Neurosci. 2013;33:1589‐1597. doi:10.1523/JNEUROSCI.1802‐12.2013 23345232 10.1523/JNEUROSCI.1802-12.2013 PMC6618731

[617] Medhurst AD , Roberts JC , Lee J , et al. Characterization of histamine H3 receptors in Alzheimer's disease brain and amyloid over‐expressing TASTPM mice. Br J Pharmacol. 2009;157:130‐138. doi:10.1111/j.1476‐5381.2008.00075.x 19222483 10.1111/j.1476-5381.2008.00075.x PMC2697792

[618] Zhang M , Liu L‐Y , Xu Y , et al. Imbalance of multiple neurotransmitter pathways leading to depression‐like behavior and cognitive dysfunction in the triple transgenic mouse model of Alzheimer disease. Metab Brain Dis. 2023;38:2465‐2476. doi:10.1007/s11011‐023‐01242‐2 37256468 10.1007/s11011-023-01242-2

[619] Mazurkiewicz‐Kwilecki IM , Prell GD . Brain histamine response to stress in 12 month old rats. Life Sci. 1986;38:2339‐2345. doi:10.1016/0024‐3205(86)90641‐7 2425206 10.1016/0024-3205(86)90641-7

[620] Mazurkiewicz‐Kwilecki IM , Prell GD . Age‐related changes in brain histamine. Agents and Actions. 1984;14:554‐557. doi:10.1007/BF01973870 6731185 10.1007/BF01973870

[621] Goncalves‐Garcia M , Davies S , Savage DD , Hamilton DA . The histamine H3 receptor inverse agonist SAR‐152954 reverses deficits in long‐term potentiation associated with moderate prenatal alcohol exposure. Alcohol. 2024;118:45‐55. doi:10.1016/j.alcohol.2024.04.005 38705312 10.1016/j.alcohol.2024.04.005 PMC11409852

[622] Savage DD , Rosenberg MJ , Wolff CR , et al. Effects of a novel cognition‐enhancing agent on fetal ethanol‐induced learning deficits. Alcohol Clin Exp Res. 2010;34:1793‐1802. doi:10.1111/j.1530‐0277.2010.01266.x 20626729 10.1111/j.1530-0277.2010.01266.x PMC3654805

[623] Varaschin RK , Rosenberg MJ , Hamilton DA , Savage DD . Differential effects of the histamine H3 receptor agonist methimepip on dentate granule cell excitability, paired‐pulse plasticity and long‐term potentiation in prenatal alcohol‐exposed rats. Alcoholism: Clin Experim Res. 2014;38:1902‐1911. doi:10.1111/acer.12430 10.1111/acer.12430 PMC5094461 24818819

[624] Varaschin RK , Akers KG , Rosenberg MJ , Hamilton DA , Savage DD . Effects of the cognition‐enhancing agent ABT‐239 on fetal ethanol‐induced deficits in dentate gyrus synaptic plasticity. J Pharmacol Experim Therap. 2010;334:191‐198. doi:10.1124/jpet.109.165027 10.1124/jpet.109.165027 PMC2912053 20308329

[625] Lenz KM , Pickett LA , Wright CL , Davis KT , Joshi A , McCarthy MM . Mast cells in the developing brain determine adult sexual behavior. J Neurosci. 2018;38:8044‐8059. doi:10.1523/JNEUROSCI.1176‐18.2018 30093566 10.1523/JNEUROSCI.1176-18.2018 PMC6136154

[626] Mackey E , Thelen KM , Bali V , et al. Perinatal androgens organize sex differences in mast cells and attenuate anaphylaxis severity into adulthood. Proc Natl Acad Sci. 2020;117:23751‐23761. doi:10.1073/pnas.1915075117 32917815 10.1073/pnas.1915075117 PMC7519313

[627] Muñoz‐Cruz S , Mendoza‐Rodríguez Y , Nava‐Castro KE , Yepez‐Mulia L , Morales‐Montor J . Gender‐related effects of sex steroids on histamine release and FcεRI expression in rat peritoneal mast cells. J Immunol Res. 2015;2015:351829. doi:10.1155/2015/351829 25973435 10.1155/2015/351829 PMC4417946

[628] Lima AP , Lunardi LO , Rosa E Silva AA . Effects of castration and testosterone replacement on peritoneal histamine concentration and lung histamine concentration in pubertal male rats. J Endocrinol. 2000;167:71‐75. doi:10.1677/joe.0.1670071 11018754 10.1677/joe.0.1670071

[629] Guhl S , Artuc M , Zuberbier T , Babina M . Testosterone exerts selective anti‐inflammatory effects on human skin mast cells in a cell subset dependent manner. Experim Dermatol. 2012;21:878‐880. doi:10.1111/exd.12006 10.1111/exd.12006 23163656

[630] Thurmond RL , Gelfand EW , Dunford PJ . The role of histamine H1 and H4 receptors in allergic inflammation: the search for new antihistamines. Nat Rev Drug Discov. 2008;7:41‐53. doi:10.1038/nrd2465 18172439 10.1038/nrd2465

[631] Zampeli E , Tiligada E . The role of histamine H4 receptor in immune and inflammatory disorders. Br J Pharmacol. 2009;157:24‐33. doi:10.1111/j.1476‐5381.2009.00151.x 19309354 10.1111/j.1476-5381.2009.00151.x PMC2697784

[632] Harcha PA , Vargas A , Yi C , Koulakoff AA , Giaume C , Sáez JC . Hemichannels are required for amyloid β‐peptide‐induced degranulation and are activated in brain mast cells of APPswe/PS1dE9 mice. J Neurosci. 2015;35:9526‐9538. doi:10.1523/JNEUROSCI.3686‐14.2015 26109673 10.1523/JNEUROSCI.3686-14.2015 PMC6605189

[633] Nelson RB , Siman R , Iqbal MA , Potter H . Identification of a chymotrypsin‐like mast cell protease in rat brain capable of generating the N‐terminus of the Alzheimer amyloid beta‐protein. J Neurochem. 1993;61:567‐577. doi:10.1111/j.1471‐4159.1993.tb02160.x 8336143 10.1111/j.1471-4159.1993.tb02160.x

[634] Niederhoffer N , Levy R , Sick E , et al. Amyloid beta peptides trigger CD47‐dependent mast cell secretory and phagocytic responses. Int J Immunopathol Pharmacol. 2009;22:473‐483. doi:10.1177/039463200902200224 19505377 10.1177/039463200902200224

[635] Rocha SM , Saraiva T , Cristóvão AC , et al. Histamine induces microglia activation and dopaminergic neuronal toxicity via H1 receptor activation. J Neuroinflam. 2016;13:137. doi:10.1186/s12974‐016‐0600‐0 10.1186/s12974-016-0600-0 PMC4893260 27260166

[636] Rocha SM , Pires J , Esteves M , Graça B , Bernardino L . Histamine: a new immunomodulatory player in the neuron‐glia crosstalk. Front Cell Neurosci. 2014;8:120. doi:10.3389/fncel.2014.00120 24817841 10.3389/fncel.2014.00120 PMC4012198

[637] Yue J , Tan Y , Huan R , et al. Mast cell activation mediates blood‐brain barrier impairment and cognitive dysfunction in septic mice in a histamine‐dependent pathway. Front Immunol. 2023;14:1090288. doi:10.3389/fimmu.2023.1090288 36817492 10.3389/fimmu.2023.1090288 PMC9929573

[638] Hersey M , Samaranayake S , Berger SN , et al. Inflammation‐induced histamine impairs the capacity of escitalopram to increase hippocampal extracellular serotonin. J Neurosci. 2021;41:6564‐6577. doi:10.1523/JNEUROSCI.2618‐20.2021 34083254 10.1523/JNEUROSCI.2618-20.2021 PMC8318079

[639] Abbott NJ . Inflammatory mediators and modulation of blood‐brain barrier permeability. Cell Mol Neurobiol. 2000;20:131‐147. doi:10.1023/a:1007074420772 10696506 10.1023/A:1007074420772 PMC11537513

[640] Dalla C , Pavlidi P , Sakelliadou D‐G , Grammatikopoulou T , Kokras N . Sex differences in blood–brain barrier transport of psychotropic drugs. Front Behav Neurosci. 2022;16:844916. doi:10.3389/fnbeh.2022.844916 35677576 10.3389/fnbeh.2022.844916 PMC9169874

[641] Weber CM , Clyne AM . Sex differences in the blood‐brain barrier and neurodegenerative diseases. APL Bioeng. 2021;5:011509. doi:10.1063/5.0035610 33758788 10.1063/5.0035610 PMC7968933

[642] Lin C‐CJ , Herisson F , Le H , et al. Mast cell deficiency improves cognition and enhances disease‐associated microglia in 5XFAD mice. Cell Rep. 2023;42:113141. doi:10.1016/j.celrep.2023.113141 37713312 10.1016/j.celrep.2023.113141 PMC10634538

[643] Jones MK , Nair A , Gupta M . Mast cells in neurodegenerative disease. Front Cell Neurosci. 2019;13:171. doi:10.3389/fncel.2019.00171 31133804 10.3389/fncel.2019.00171 PMC6524694

[644] Shaik‐Dasthagirisaheb YB , Conti P . The role of mast cells in Alzheimer's disease. Adv Clin Exp Med. 2016;25:781‐787. doi:10.17219/acem/61914 27629855 10.17219/acem/61914

[645] Mieda M , Sakurai T . Overview of orexin/hypocretin system. Prog Brain Res. 2012;198:5‐14. doi:10.1016/B978‐0‐444‐59489‐1.00002‐1 22813966 10.1016/B978-0-444-59489-1.00002-1

[646] Berteotti C , Liguori C , Pace M . Dysregulation of the orexin/hypocretin system is not limited to narcolepsy but has far‐reaching implications for neurological disorders. Eur J Neurosci. 2021;53:1136‐1154. doi:10.1111/ejn.15077 33290595 10.1111/ejn.15077

[647] Sakurai T . The role of orexin in motivated behaviours. Nat Rev Neurosci. 2014;15:719‐731. doi:10.1038/nrn3837 25301357 10.1038/nrn3837

[648] Soya S , Sakurai T . Evolution of orexin neuropeptide system: structure and function. Front Neurosci. 2020;14:691. doi:10.3389/fnins.2020.00691 32754010 10.3389/fnins.2020.00691 PMC7365868

[649] Sakurai T , Amemiya A , Ishii M , et al. Orexins and orexin receptors: a family of hypothalamic neuropeptides and G protein‐coupled receptors that regulate feeding behavior. Cell. 1998;92:573‐585. doi:10.1016/s0092‐8674(00)80949‐6 9491897 10.1016/s0092-8674(00)80949-6

[650] de Lecea L , Kilduff TS , Peyron C , et al. The hypocretins: hypothalamus‐specific peptides with neuroexcitatory activity. Proc Natl Acad Sci U S A. 1998;95:322‐327. doi:10.1073/pnas.95.1.322 9419374 10.1073/pnas.95.1.322 PMC18213

[651] Pu S , Jain MR , Kalra PS , Kalra SP . Orexins, a novel family of hypothalamic neuropeptides, modulate pituitary luteinizing hormone secretion in an ovarian steroid‐dependent manner. Regul Pept. 1998;78:133‐136. doi:10.1016/s0167‐0115(98)00128‐1 9879756 10.1016/s0167-0115(98)00128-1

[652] Taheri S , Mahmoodi M , Opacka‐Juffry J , Ghatei MA , Bloom SR . Distribution and quantification of immunoreactive orexin A in rat tissues. FEBS Lett. 1999;457:157‐161. doi:10.1016/s0014‐5793(99)01030‐3 10486585 10.1016/s0014-5793(99)01030-3

[653] Jöhren O , Neidert SJ , Kummer M , Dominiak P . Sexually dimorphic expression of prepro‐orexin mRNA in the rat hypothalamus. Peptides. 2002;23:1177‐1180. doi:10.1016/s0196‐9781(02)00052‐9 12126748 10.1016/s0196-9781(02)00052-9

[654] Jöhren O , Neidert SJ , Kummer M , Dendorfer A , Dominiak P . Prepro‐orexin and orexin receptor mRNAs are differentially expressed in peripheral tissues of male and female rats. Endocrinology. 2001;142:3324‐3331. doi:10.1210/endo.142.8.8299 11459774 10.1210/endo.142.8.8299

[655] Jöhren O , Brüggemann N , Dendorfer A , Dominiak P . Gonadal steroids differentially regulate the messenger ribonucleic acid expression of pituitary orexin type 1 receptors and adrenal orexin type 2 receptors. Endocrinology. 2003;144:1219‐1225. doi:10.1210/en.2002‐0030 12639903 10.1210/en.2002-0030

[656] Silveyra P , Catalano PN , Lux‐Lantos V , Libertun C . Impact of proestrous milieu on expression of orexin receptors and prepro‐orexin in rat hypothalamus and hypophysis: actions of Cetrorelix and Nembutal. Am J Physiol Endocrinol Metab. 2007;292:E820‐828. doi:10.1152/ajpendo.00467.2006 17122088 10.1152/ajpendo.00467.2006

[657] Small CJ , Goubillon M‐L , Murray JF , et al. Central orexin a has site‐specific effects on luteinizing hormone release in female rats. Endocrinology. 2003;144:3225‐3236. doi:10.1210/en.2002‐0041 12810579 10.1210/en.2002-0041

[658] Campbell RE , Grove KL , Smith MS . Gonadotropin‐releasing hormone neurons coexpress orexin 1 receptor immunoreactivity and receive direct contacts by orexin fibers. Endocrinology. 2003;144:1542‐1548. doi:10.1210/en.2002‐220958 12639939 10.1210/en.2002-220958

[659] Iqbal J , Pompolo S , Sakurai T , Clarke IJ . Evidence that orexin‐containing neurones provide direct input to gonadotropin‐releasing hormone neurones in the ovine hypothalamus. J Neuroendocrinol. 2001;13:1033‐1041. doi:10.1046/j.1365‐2826.2001.00719.x 11722699 10.1046/j.1365-2826.2001.00719.x

[660] Russell SH , Small CJ , Kennedy AR , et al. Orexin A interactions in the hypothalamo‐pituitary gonadal axis. Endocrinology. 2001;142:5294‐5302. doi:10.1210/endo.142.12.8558 11713229 10.1210/endo.142.12.8558

[661] Tamura T , Irahara M , Tezuka M , Kiyokawa M , Aono T . Orexins, orexigenic hypothalamic neuropeptides, suppress the pulsatile secretion of luteinizing hormone in ovariectomized female rats. Biochem Biophys Res Commun. 1999;264:759‐762. doi:10.1006/bbrc.1999.1573 10544004 10.1006/bbrc.1999.1573

[662] Furuta M , Funabashi T , Kimura F . Suppressive action of orexin A on pulsatile luteinizing hormone secretion is potentiated by a low dose of estrogen in ovariectomized rats. Neuroendocrinology. 2002;75:151‐157. doi:10.1159/000048232 11914586 10.1159/000048232

[663] Tenorio‐Lopes L , Fournier S , Henry MS , Bretzner F , Kinkead R . Disruption of estradiol regulation of orexin neurons: a novel mechanism in excessive ventilatory response to CO2 inhalation in a female rat model of panic disorder. Transl Psychiatry. 2020;10:394. doi:10.1038/s41398‐020‐01076‐x 33173029 10.1038/s41398-020-01076-x PMC7656265

[664] Muschamp JW , Dominguez JM , Sato SM , Shen R‐Y , Hull EM . A Role for Hypocretin (Orexin) in Male Sexual Behavior. J Neurosci. 2007;27:2837‐2845. doi:10.1523/JNEUROSCI.4121‐06.2007 17360905 10.1523/JNEUROSCI.4121-06.2007 PMC6672590

[665] Cataldi NI , Lux‐Lantos VA , Libertun C . Perinatal programming of the orexinergic (hypocretinergic) system in hypothalamus and anterior pituitary by testosterone. Peptides. 2018;99:117‐127. doi:10.1016/j.peptides.2017.04.006 28442349 10.1016/j.peptides.2017.04.006

[666] Di Sebastiano AR , Wilson‐Pérez HE , Lehman MN , Coolen LM . Lesions of orexin neurons block conditioned place preference for sexual behavior in male rats. Horm Behav. 2011;59:1‐8. doi:10.1016/j.yhbeh.2010.09.006 20851122 10.1016/j.yhbeh.2010.09.006

[667] Fujiki N , Yoshida Y , Zhang S , Sakurai T , Yanagisawa M , Nishino S . Sex difference in body weight gain and leptin signaling in hypocretin/orexin deficient mouse models. Peptides. 2006;27:2326‐2331. doi:10.1016/j.peptides.2006.03.011 16626839 10.1016/j.peptides.2006.03.011 PMC1616410

[668] Ramanathan L , Siegel JM . Gender differences between hypocretin/orexin knockout and wild type mice: age, body weight, body composition, metabolic markers, leptin and insulin resistance. J Neurochem. 2014;131:615‐624. doi:10.1111/jnc.12840 25066943 10.1111/jnc.12840 PMC8734533

[669] Grafe LA , Cornfeld A , Luz S , Valentino R , Bhatnagar S . Orexins mediate sex differences in the stress response and in cognitive flexibility. Biol Psychiatry. 2017;81:683‐692. doi:10.1016/j.biopsych.2016.10.013 27955897 10.1016/j.biopsych.2016.10.013 PMC5359079

[670] Lu J , Zhao J , Balesar R , et al. Sexually dimorphic changes of hypocretin (orexin) in depression. eBioMedicine. 2017;18:311‐319. doi:10.1016/j.ebiom.2017.03.043 28377228 10.1016/j.ebiom.2017.03.043 PMC5405188

[671] Hungs M , Mignot E . Hypocretin/orexin, sleep and narcolepsy. Bioessays. 2001;23:397‐408. doi:10.1002/bies.1058 11340621 10.1002/bies.1058

[672] Carpi M , Palagini L , Fernandes M , et al. Clinical usefulness of dual orexin receptor antagonism beyond insomnia: neurological and psychiatric comorbidities. Neuropharmacology. 2024;245:109815. doi:10.1016/j.neuropharm.2023.109815 38114045 10.1016/j.neuropharm.2023.109815

[673] Ten‐Blanco M , Flores Á , Cristino L , Pereda‐Pérez I , Berrendero F . Targeting the orexin/hypocretin system for the treatment of neuropsychiatric and neurodegenerative diseases: from animal to clinical studies. Front Neuroendocrinol. 2023;69:101066. doi:10.1016/j.yfrne.2023.101066 37015302 10.1016/j.yfrne.2023.101066

[674] Krishnan V , Collop NA . Gender differences in sleep disorders. Curr Opin Pulm Med. 2006;12:383‐389. doi:10.1097/01.mcp.0000245705.69440.6a 17053485 10.1097/01.mcp.0000245705.69440.6a

[675] Tesic A , Rodgers S , Müller M , et al. Sex differences in neurodevelopmental and common mental disorders examined from three epidemiological perspectives. Psychiatry Res. 2019;278:213‐217. doi:10.1016/j.psychres.2019.06.019 31226547 10.1016/j.psychres.2019.06.019

[676] Mong JA , Cusmano DM . Sex differences in sleep: impact of biological sex and sex steroids. Philos Trans R Soc Lond B Biol Sci. 2016;371:20150110. doi:10.1098/rstb.2015.0110 26833831 10.1098/rstb.2015.0110 PMC4785896

[677] Meers J , Stout‐Aguilar J , Nowakowski S . Chapter 3 ‐ Sex differences in sleep health. In: Grandner MA , ed. Sleep and Health. Academic Press; 2019:21‐29. doi:10.1016/B978‐0‐12‐815373‐4.00003‐4

[678] Prober DA , Rihel J , Onah AA , Sung R‐J , Schier AF . Hypocretin/orexin overexpression induces an insomnia‐like phenotype in zebrafish. J Neurosci. 2006;26:13400‐13410. doi:10.1523/JNEUROSCI.4332‐06.2006 17182791 10.1523/JNEUROSCI.4332-06.2006 PMC6675014

[679] Palagini L , Geoffroy PA , Balestrieri M , et al. Current models of insomnia disorder: a theoretical review on the potential role of the orexinergic pathway with implications for insomnia treatment. J Sleep Res. 2023;32:e13825. doi:10.1111/jsr.13825 36786121 10.1111/jsr.13825

[680] Zhang B , Wing Y‐K . Sex differences in insomnia: a meta‐analysis. Sleep. 2006;29:85‐93. doi:10.1093/sleep/29.1.85 16453985 10.1093/sleep/29.1.85

[681] Mallampalli MP , Carter CL . Exploring sex and gender differences in sleep health: a Society for Women's Health Research Report. J Womens Health (Larchmt). 2014;23:553‐562. doi:10.1089/jwh.2014.4816 24956068 10.1089/jwh.2014.4816 PMC4089020

[682] Grafe LA , Bhatnagar S . The contribution of orexins to sex differences in the stress response. Brain Res. 2020;1731:145893. doi:10.1016/j.brainres.2018.07.026 30081036 10.1016/j.brainres.2018.07.026 PMC6360123

[683] Fronczek R , van Geest S , Frölich M , et al. Hypocretin (orexin) loss in Alzheimer's disease. Neurobiol Aging. 2012;33:1642‐1650. doi:10.1016/j.neurobiolaging.2011.03.014 21546124 10.1016/j.neurobiolaging.2011.03.014

[684] Schmidt FM , Kratzsch J , Gertz H‐J , et al. Cerebrospinal fluid melanin‐concentrating hormone (MCH) and hypocretin‐1 (HCRT‐1, orexin‐A) in Alzheimer's disease. PLoS One. 2013;8:e63136. doi:10.1371/journal.pone.0063136 23667582 10.1371/journal.pone.0063136 PMC3646736

[685] Wennström M , Londos E , Minthon L , Nielsen HM . Altered CSF orexin and α‐synuclein levels in dementia patients. J Alzheimers Dis. 2012;29:125‐132. doi:10.3233/JAD‐2012‐111655 22207004 10.3233/JAD-2012-111655

[686] Dauvilliers YA , Lehmann S , Jaussent I , Gabelle A . Hypocretin and brain β‐amyloid peptide interactions in cognitive disorders and narcolepsy. Front Aging Neurosci. 2014;6:119. doi:10.3389/fnagi.2014.00119 24966833 10.3389/fnagi.2014.00119 PMC4052448

[687] Liguori C , Romigi A , Nuccetelli M , et al. Orexinergic system dysregulation, sleep impairment, and cognitive decline in Alzheimer disease. JAMA Neurol. 2014;71:1498‐1505. doi:10.1001/jamaneurol.2014.2510 25322206 10.1001/jamaneurol.2014.2510

[688] Liguori C , Nuccetelli M , Izzi F , et al. Rapid eye movement sleep disruption and sleep fragmentation are associated with increased orexin‐A cerebrospinal‐fluid levels in mild cognitive impairment due to Alzheimer's disease. Neurobiol Aging. 2016;40:120‐126. doi:10.1016/j.neurobiolaging.2016.01.007 26973111 10.1016/j.neurobiolaging.2016.01.007

[689] Lozano‐Tovar S , Cremascoli R , Nuccetelli M , et al. Cerebrospinal‐fluid Orexin‐A levels in different neurocognitive disorders: a comparison study. Neurol Sci. 2025;46:3631‐3638. doi:10.1007/s10072‐025‐08148‐0 40198471 10.1007/s10072-025-08148-0 PMC12267319

[690] Pike CJ . Sex and the development of Alzheimer's disease. J Neurosci Res. 2017;95:671‐680. doi:10.1002/jnr.23827 27870425 10.1002/jnr.23827 PMC5120614

[691] Wang C , Zhang F , Jiang S , et al. Estrogen receptor‐α is localized to neurofibrillary tangles in Alzheimer's disease. Sci Rep. 2016;6:20352. doi:10.1038/srep20352 26837465 10.1038/srep20352 PMC4738266

[692] Merlo S , Spampinato SF , Sortino MA . Estrogen and Alzheimer's disease: still an attractive topic despite disappointment from early clinical results. European Journal of Pharmacology. 2017;817:51‐58. doi:10.1016/j.ejphar.2017.05.059 28577965 10.1016/j.ejphar.2017.05.059

[693] McHill AW , Klerman EB , Slater B , Kangarloo T , Mankowski PW , Shaw ND . The relationship between estrogen and the decline in delta power during adolescence. Sleep. 2017;40:zsx008. doi:10.1093/sleep/zsx008 28364433 10.1093/sleep/zsx008 PMC5806564

[694] Tansupswatdikul P , Chaikittisilpa S , Jaimchariyatam N , Panyakhamlerd K , Jaisamrarn U , Taechakraichana N . Effects of estrogen therapy on postmenopausal sleep quality regardless of vasomotor symptoms: a randomized trial. Climacteric. 2015;18:198‐204. doi:10.3109/13697137.2014.964670 25242569 10.3109/13697137.2014.964670

[695] Joffe H , Crawford SL , Freeman MP , et al. Independent contributions of nocturnal hot flashes and sleep disturbance to depression in estrogen‐deprived women. J Clin Endocrinol Metab. 2016;101:3847‐3855. doi:10.1210/jc.2016‐2348 27680875 10.1210/jc.2016-2348 PMC5052351

[696] Gervais NJ , Mong JA , Lacreuse A . Ovarian hormones, sleep and cognition across the adult female lifespan: an integrated perspective. Front Neuroendocrinol. 2017;47:134‐153. doi:10.1016/j.yfrne.2017.08.002 28803147 10.1016/j.yfrne.2017.08.002 PMC7597864

[697] Keenan RJ , Daykin H , Chu J , et al. Differential sleep/wake response and sex differences following acute suvorexant, MK‐1064 and zolpidem administration in the rTg4510 mouse model of tauopathy. Br J Pharmacol. 2022;179:3403‐3417. doi:10.1111/bph.15813 35112344 10.1111/bph.15813 PMC9302982

[698] Keenan RJ , Daykin H , Metha J , et al. Orexin 2 receptor antagonism sex‐dependently improves sleep/wakefulness and cognitive performance in tau transgenic mice. Br J Pharmacol. 2024;181:87‐106. doi:10.1111/bph.16212 37553894 10.1111/bph.16212

[699] Parhizkar S , Bao X , Chen W , et al. Lemborexant ameliorates tau‐mediated sleep loss and neurodegeneration in males in a mouse model of tauopathy. Nat Neurosci. 2025;28:1460‐1472. doi:10.1038/s41593‐025‐01966‐7 40425791 10.1038/s41593-025-01966-7 PMC12277058

[700] Xue T , Wu X , Chen S , et al. The efficacy and safety of dual orexin receptor antagonists in primary insomnia: a systematic review and network meta‐analysis. Sleep Med Rev. 2022;61:101573. doi:10.1016/j.smrv.2021.101573 34902823 10.1016/j.smrv.2021.101573

[701] Carpi M , Mercuri NB , Liguori C . Orexin receptor antagonists for the prevention and treatment of Alzheimer's disease and associated sleep disorders. Drugs. 2024;84:1365‐1378. doi:10.1007/s40265‐024‐02096‐3 39365407 10.1007/s40265-024-02096-3 PMC11602839

[702] Herring WJ , Connor KM , Snyder E , et al. Clinical profile of suvorexant for the treatment of insomnia over 3 months in women and men: subgroup analysis of pooled phase‐3 data. Psychopharmacology (Berl). 2017;234:1703‐1711. doi:10.1007/s00213‐017‐4573‐1 28265715 10.1007/s00213-017-4573-1

[703] Herring WJ , Ceesay P , Snyder E , et al. Polysomnographic assessment of suvorexant in patients with probable Alzheimer's disease dementia and insomnia: a randomized trial. Alzheimers Dement. 2020;16:541‐551. doi:10.1002/alz.12035 31944580 10.1002/alz.12035 PMC7984350

